# Direct decarboxylative Giese amidations: photocatalytic *vs.* metal- and light-free†

**DOI:** 10.1039/d3sc03143h

**Published:** 2023-08-24

**Authors:** David M. Kitcatt, Katie A. Scott, Elena Rongione, Simon Nicolle, Ai-Lan Lee

**Affiliations:** a Institute of Chemical Sciences, School of Engineering and Physical Sciences, Heriot-Watt University Edinburgh EH14 4AS UK A.Lee@hw.ac.uk; b GlaxoSmithKline Gunnels Wood Rd Stevenage SG1 2NY UK

## Abstract

A direct intermolecular decarboxylative Giese amidation reaction from bench stable, non-toxic and environmentally benign oxamic acids has been developed, which allows for easy access to 1,4-difunctionalised compounds which are not otherwise readily accessible. Crucially, a more general acceptor substrate scope is now possible, which renders the Giese amidation applicable to more complex substrates such as natural products and chiral building blocks. Two different photocatalytic methods (one *via* oxidative and the other *via* reductive quenching cycles) and one metal- and light-free method were developed and the flexibility provided by different conditions proved to be crucial for enabling a more general substrate scope.

## Abstract 2

A direct decarboxylative Giese amidation reaction from oxamic acids has been achieved, allowing easy access to previously inaccessible 1,4-difunctionalised compounds.

## Graphical abstract

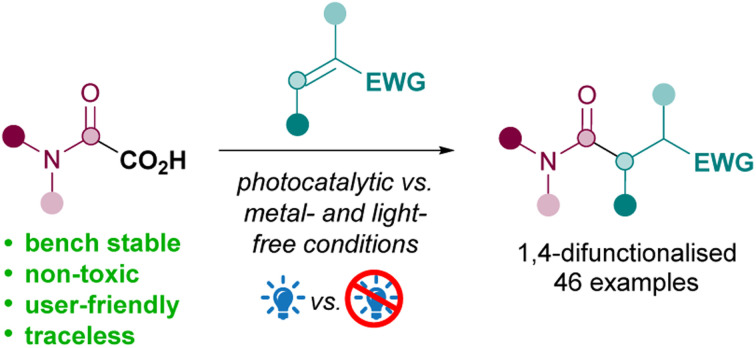

## Introduction

Giese radical conjugate addition reactions have re-emerged at the forefront of radical chemistry as a powerful method for forming C–C bonds which are not otherwise attainable *via* conventional nucleophilic protocols.^1,2^ The current popularity of the Giese reaction is largely due to the recent emergence of mild photocatalytic methodologies.^2^ The Giese alkylation, for example, has been exploited in a myriad of applications, including chemoselective bioconjugation of peptides,^3^ synthesis of unnatural amino acids,^4^ macrocyclisations,^5^ polymerisations,^6^ natural product^7^ and drug molecule synthesis.^8^ Within this context, Giese reactions that can proceed *via* direct decarboxylation from carboxylic acids (rather than *via* less atom economical activated radical precursors),^2*a*^ are highly sought after since carboxylic acids are readily available, non-toxic, easy to handle, atom economical and the carboxy group can be expelled as traceless CO_2_ from the reaction.^9^

**Scheme 1:**
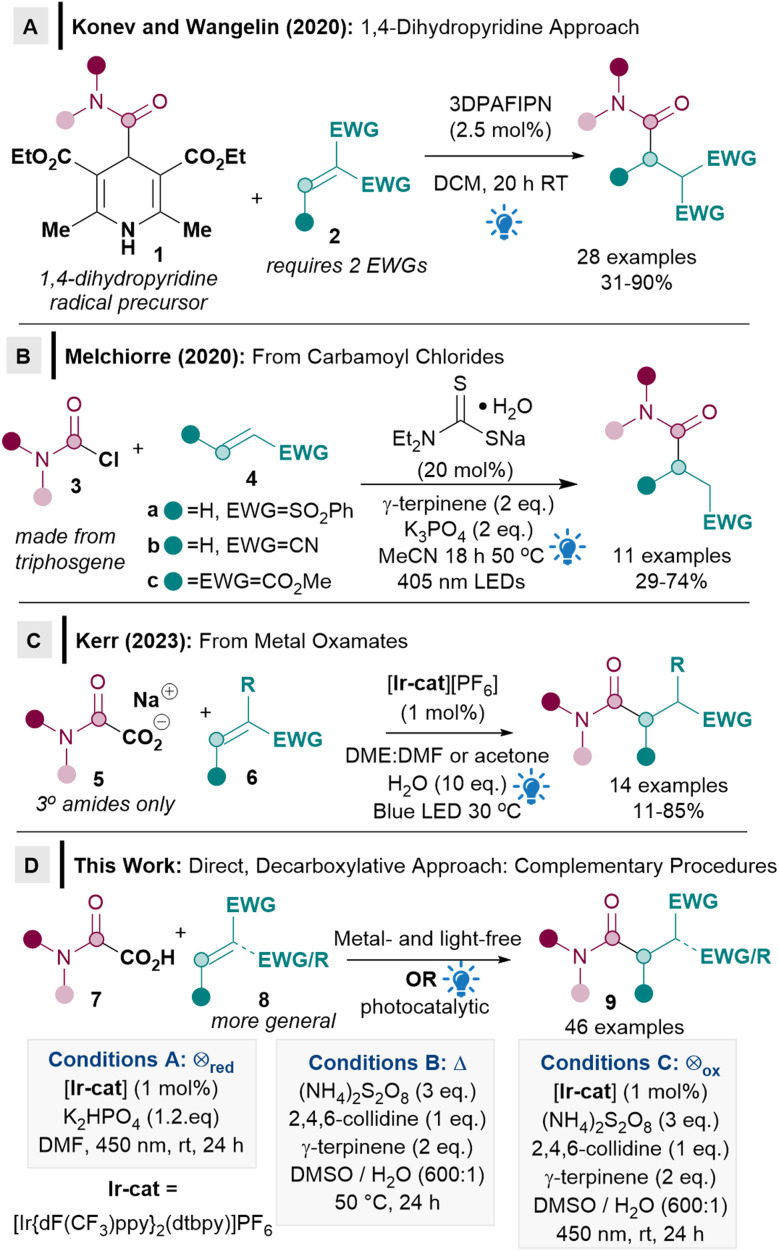
Intermolecular Giese amidations.

Although direct decarboxylative Giese alkylations^10^ and acylations^11^ have been well established, there are currently very few examples of Giese amidation reactions and crucially, no direct decarboxylative methods from oxamic acids are known.^2*a*,12^ Only two Giese amidation reactions were reported when we commenced our work, both from activated carbamoyl precursors.^13^ The seminal report by Konev and Wangelin utilised activated Hantzsch ester derivatives 1 as radical precursors under organophotocatalytic conditions (Scheme 1A).^13*a*^ Although 1 has the advantage of being activated, it however results in poor atom economy. The substrate scope of the acceptor is also limited to highly activated ones, usually with two strong electron-withdrawing groups (EWGs, 2).

Conversely, Melchiorre's pioneering procedure using carbamoyl chlorides 3 as radical precursors used acceptors 4 with only one EWG, although the exemplified substrate scope appeared restrictive (4a–c, Scheme 1B).^13*b*^ The use of moisture sensitive carbamoyl chlorides 3, some of which are carcinogenic, can also be problematic, since they are often made from highly toxic triphosgene.^13*b*^

While the above two approaches are important as they constitute the first two examples of Giese amidation, it is also clear that two major limitations exist. Firstly, the ease of use, toxicity, atom economy and accessibility related to the identity of the carbamoyl radical precursor (1, 3) needs to be improved significantly for Giese amidations to be synthetically useful and more widely adopted by the synthetic community. The use of oxamic acids 7 as an environmentally benign precursor to carbamoyl radicals^14^ would solve this issue, but there are currently no reports of its use in Giese reactions. Secondly, the acceptor substrate scope needs to be substantially expanded beyond the current limitation of requiring either two activating EWGs (2), phenyl vinyl sulfone, acrylonitrile, or dimethyl maleate (4). During the preparation of this manuscript, Kerr disclosed an elegant Giese amidation procedure from metal oxamates 5 (Scheme 1C).^15^ Kerr's procedure partly addresses some of the Michael acceptor scope limitations, however, the amidation scope seems to be limited to tertiary amides. Metal oxamates 5 are a significant improvement on precursor 3 in terms of toxicity, but oxamates 5 are still hygroscopic. The key challenges of a direct reaction from oxamic acids 7 and a more general substrate scope are therefore still pertinent.

We herein report the first direct decarboxylative Giese amidation reaction from bench stable, non-toxic and user friendly oxamic acids 7,^14^ which benefits from having only traceless CO_2_ released from the radical precursor (Scheme 1D). Crucially, a more general acceptor substrate scope 8 is now possible for the Giese amidation, which renders the reaction applicable to more complex substrates such as natural products and chiral building blocks. Three different conditions were developed and compared to ascertain the most suitable methodology: photocatalytic reductive quenching cycle (conditions A), metal- and light-free (conditions B), and photocatalytic oxidative quenching cycle (conditions C). The complementarity and flexibility provided by different conditions will prove to be crucial for enabling a more general substrate scope.

## Results and discussion

**Scheme 2:**
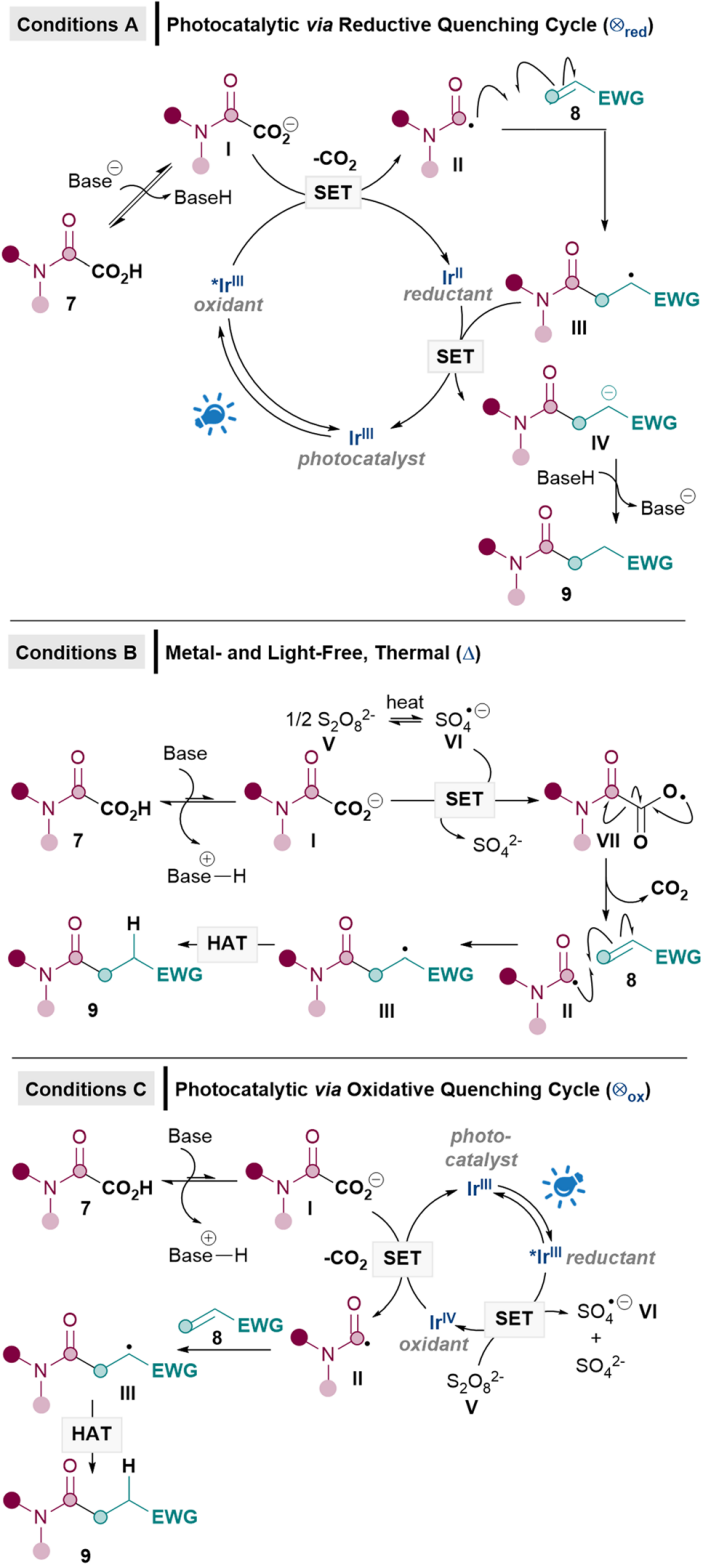
Proposed mechanisms. Conditions A: photocatalytic reductive quenching cycle. Conditions B: metal- and light-free thermal decarboxylation. Conditions C: photocatalytic oxidative quenching cycle.

Our proposed mechanisms for the three sets of conditions are shown in Scheme 2. We initially adapted the conditions originally developed by Macmillan based on a reductive quenching cycle mechanism (conditions A),^8^ since this protocol has been used in a number of decarboxylative Giese alkylation and acylation reactions reported thereafter.^2*a*^ In this reductive quenching cycle, the excited photocatalyst [*e.g.* *Ir^III^ (*E*_1/2_*^III/II^ = +1.21 V *vs.* SCE) for [Ir{dF(CF_3_)ppy}_2_(dtbpy)PF_6_]^16^ undergoes single electron transfer (SET) to yield the carboxylate radical from I (*E*_ox_ = +1.17 V *vs.* SCE),^17^ which should then decarboxylate to form the carbamoyl radical II. Radical addition of II to 8 furnishes radical III. SET reduction by Ir^II^ (*E*_1/2_^III/II^ = −1.37 V *vs.* SCE)^16^ to produce IV followed by protonation yields the Giese product 9. Unfortunately, it soon became apparent that adapting these Giese alkylation conditions for amidations was sub-optimal, yielding only a poor 34% of desired 9a with model substrates 7a and 8a (see later, Table 3).

When reductive quenching cycle conditions failed to work in a key decarboxylative Giese alkylation step in Baran's synthesis of (−)-maximiscin, silver catalysis using oxidative Kochi conditions [Ag(i) and Na_2_S_2_O_8_] was ultimately utilised.^7*d*^ For this reason, we decided to develop two oxidative methodologies in our effort to achieve the first efficient direct decarboxylative Giese amidations. Rather than using Kochi conditions, however, we set out to develop a metal- and light-free Giese method (conditions B), inspired by our recent success with metal- and light-free Minisci reactions.^18^ Using DMSO as the solvent allows for the breakdown of S_2_O_8_^2−^V to the active SO_4_^−·^VI (*E*_ox_ = +2.51–3.1 V *vs.* SHE)^19^ under mild conditions (40–50 °C), without the need for metal mediation or photolysis (Scheme 2).^18,20^ This could potentially be exploited in the Giese reaction, since SET between VI and carboxylate I (*E*_ox_ = +1.17 V *vs.* SCE)^17,21^ can then occur to give radical VII,^22^ which should decarboxylate to give the carbamoyl radical II^9*a*,23^ for the Giese addition with 8. Unlike conditions A, radical III would presumably undergo hydrogen atom transfer (HAT) instead of SET/protonation to yield 9, due to the absence of an obvious reductant.

We also envisaged a related photocatalytic oxidative quenching cycle (conditions C, Scheme 2) where excited state *Ir^III^ is generated from photoexcitation of the Ir^III^ catalyst at 450 nm (*E*_1/2_^Ir(III)^*^/Ir(IV)^ = −0.89 *vs.* SCE),^24^ which then undergoes SET with persulfate V (*E*_ox_ = +1.75 V *vs.* SCE)^20^ to produce the oxidising species Ir^IV^ (*E*_1/2_^Ir(IV)/Ir(III)^ = +1.69 *vs.* SCE).^24^ Subsequent SET with carboxylate I can either be induced by Ir^IV^ or the resulting sulfate radical anion VI (as in conditions B). Development of conditions C would allow the reaction to occur at ambient temperature as well as allow for a comparison between a photocatalytic oxidative (C) and reductive quenching cycle (A) for the Giese amidations.

We therefore commenced our optimisation of the metal- and light-free conditions B using model substrates 7a and 8a (Table 1). To our delight, the Giese amidation works very well as long as an efficient HAT source is present (entries 1 *vs.* entries 2–6),^25^ with 2 equiv. of γ-terpinene identified as optimal (entry 3). The presence of a base is required for good yields (entries 7–10), with 2,4,6-collidine providing the best results (entry 3). Persulfate is crucial for reactivity (entry 15), with (NH_4_)_2_S_2_O_8_ outperforming Na_2_S_2_O_8_ and K_2_S_2_O_8_ (entries 11–12), likely due to the former's superior solubility in DMSO. A solvent screen shows that the reaction requires DMSO for appreciable conversion (entries 3 *vs.* entries 16–19). The yield drops at lower temperature (35 °C, entry 20) and under air (entry 23). A control reaction in the dark proves that the reaction under conditions B is not light mediated (entry 22) and the reaction is inhibited in the presence of TEMPO (entry 24), consistent with a radical mechanistic pathway.

**Table 1:** Optimisation studies and control experiments: metal- and light-free^a^

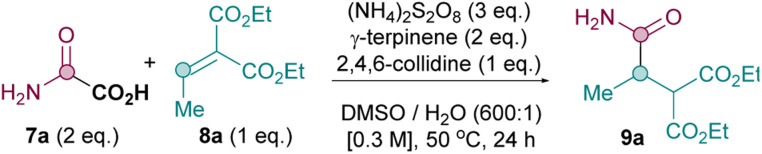		
Entry	Deviations from standard conditions	Yield^b^ (%)
1	No γ-terpinene	36
2	1,4-CHD instead of γ-terpinene	65
3	None	96
4	1 eq. of γ-terpinene	79
5	3 eq. of γ-terpinene	73
6	Hantzsch ester instead of γ-terpinene	83
7	Cs_2_CO_3_ instead of 2,4,6-collidine	65
8	K_2_HPO_4_ instead of 2,4,6-collidine	86
9	2,6-Lutidine instead of 2,4,6-collidine	84
10	No 2,4,6-collidine	55
11	Na_2_S_2_O_8_ instead of (NH_4_)_2_S_2_O_8_	14
12	K_2_S_2_O_8_ instead of (NH_4_)_2_S_2_O_8_	23
13	1 eq. of (NH_4_)_2_S_2_O_8_	72
14	5 eq. of (NH_4_)_2_S_2_O_8_	62
15	No (NH_4_)_2_S_2_O_8_	n.d.
16	H_2_O used as solvent	26
17	Acetone used as solvent	n.d.
18	DMF used as solvent	9
19	MeCN used as solvent	14
20	At 35 °C	38
21	At 80 °C	99
22	In the dark	97
23	Under air	79
24	With 3 eq. TEMPO	n.d.

a Reactions performed on a 0.12 mmol scale of 8a under Ar atmosphere.

b Yields estimated by ^1^H NMR analysis of the crude mixture using dibromomethane as the internal standard. 1,4-CHD: 1,4-cyclohexadiene. N.d.: not detected. See ESI† for full optimisation studies.

For the photocatalytic oxidative quenching cycle conditions C, optimisation studies showed that [Ir{dF(CF_3_)ppy}_2_(dtbpy)PF_6_] catalyst at 1 mol% loading yielded the best results (Table 2, entry 1, see ESI† for full optimisation studies). The Fukuzumi organophotocatalyst 9-mesityl-10-methylacridinium perchlorate^26^ gave a slightly lower yield (entry 2) but is a good alternative to the Ir catalyst should cost, toxicity and sustainability of the Ir catalyst be an issue. Control experiments prove that a HAT source (entry 3), persulfate (entry 4), photocatalyst (entry 5), base (entry 6) and light (entry 7) are all required for good reactivity under photocatalytic conditions C.

**Table 2:** Selected optimisation and control experiments: photocatalytic oxidative quenching cycle^a^

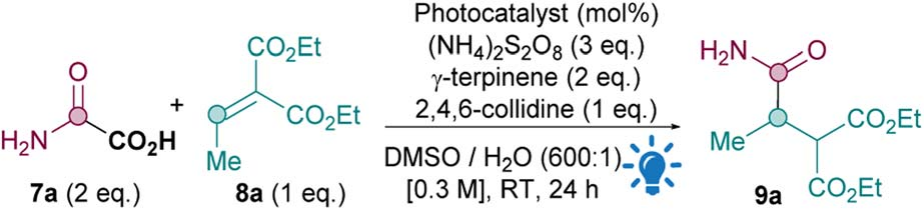				
Entry	Photocat.	Mol%	Deviations	Yield^b^ (%)
1	[Ir]	1	—	92
2	[Mes–Acr]^+^[ClO_4_]^−^	1.5	—	86
3	[Ir]	2	100% intensity; no γ-terpinene	13
4	[Ir]	1	No (NH_4_)_2_S_2_O_8_	22
5	None	0	No photocat.	17
6	[Ir]	1	No collidine	51
7	[Ir]	1	In dark	15

a Reactions performed on a 0.12 mmol scale of 8a under Ar atmosphere in a Penn PhD M2 Photoreactor, 450 nm at 50% light intensity.

b Yields estimated by ^1^H NMR analysis of the crude mixture using 1,3,5-trimethoxybenzene as the internal standard. [Ir] = [Ir{dF(CF_3_)ppy}_2_(dtbpy)PF_6_]. See ESI† for full optimisation studies.

In addition, the average quantum yield^27^ (*Φ*) was found to be 2.83 × 10^−3^ (std. dev. = 0.69 × 10^−3^) for conditions A and 11.5 × 10^−3^ (std. dev. = 8.8 × 10^−3^) for conditions C (see ESI†), thus ruling out the presence of any chain reactions under these conditions.

With optimal conditions in hand, an oxamic acid 7 substrate scope study was carried out next (Table 3). When comparing conditions A, B and C for amidation with 7a to form primary amide 9a, the metal- and light-free conditions B were superior to both photocatalytic methods A and C (A: 34%, B: 75%, C: 63%).

**Table 3:** Oxamic acid scope^a^

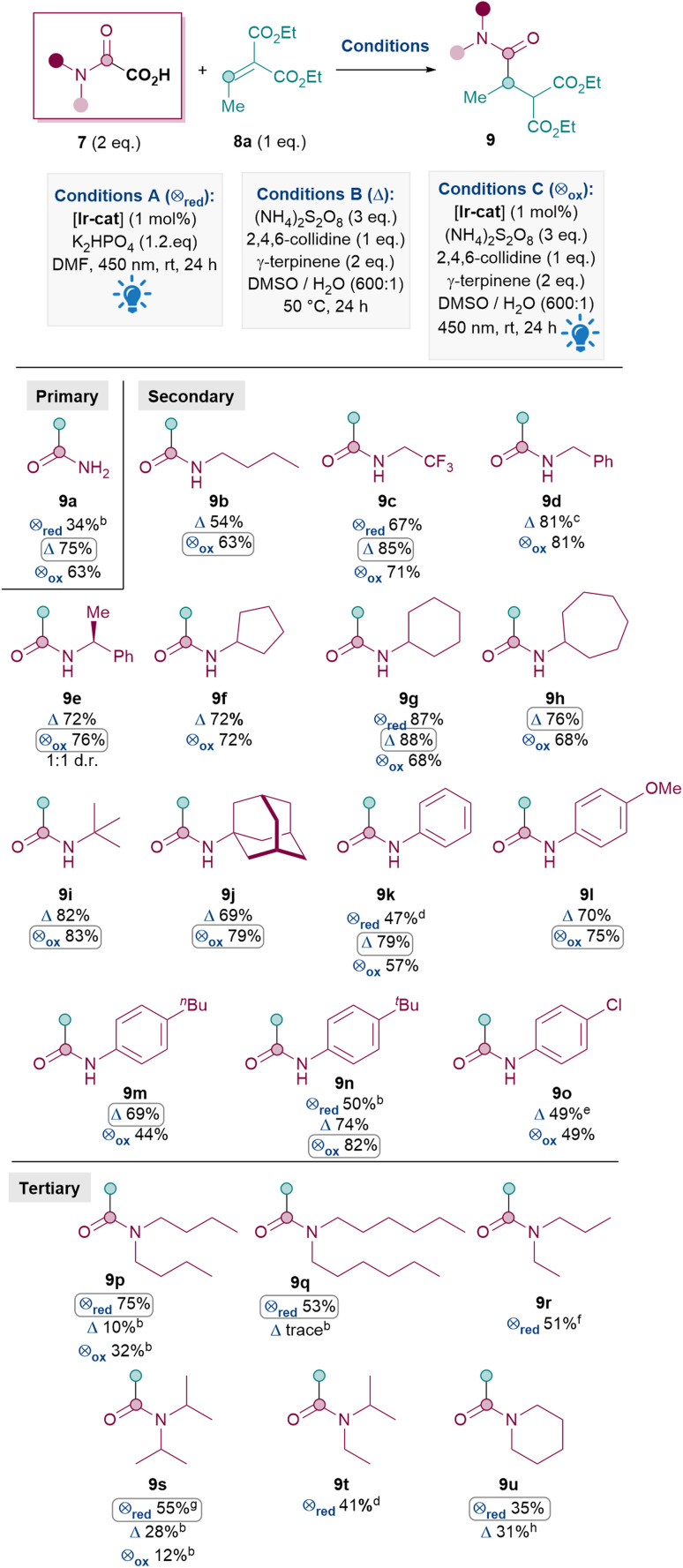

a Reactions performed on a 0.12 mmol scale of 8 under argon atmosphere and isolated yields reported unless otherwise stated. Conditions A and C were carried out in a Penn PhD M2 Photoreactor, 450 nm at 50% light intensity. [Ir-cat] = [Ir{dF(CF_3_)ppy}_2_(dtbpy)PF_6_].

b Yield determine ^1^H NMR using dibromomethane as internal standard.

c 72% yield at 1 mmol scale and 55% yield at 5.4 mmol scale.

d Reaction performed on a 0.20 mmol scale.

e Used 3 eq. of 7, 4 eq. of (NH_4_)_2_S_2_O_8_, 3 eq. of 2,4,6-collidine at 75 °C.

f Reaction performed on a 0.24 mmol scale.

g Used 4 eq. of 7 and 2.4 eq. of K_2_HPO_4_. Yield was 38% under standard conditions.

h Reacted for 48 h at 40 °C.

A similar pattern was observed for installing secondary amides: oxidative conditions (either B or C) generally outperformed reductive quenching cycle conditions A for 9c (A: 67%, B: 85%, C: 71%), 9g (A: 87%, B: 88%, C: 68%), 9k (A: 47%, B: 79%, C: 57%) and 9n (A: 50%, B: 74%, C: 82%). For this reason, only conditions B and C were investigated for the formation of the rest of the secondary amides shown in Table 3. Various aliphatic substituents on the nitrogen were tolerated well (9b–j, 63–88%), including primary alkyl (9b–d), secondary alkyl (9e–h) and tertiary alkyl (9i–j) substituents. Pleasingly, these include cyclic *N*-alkyl substituents (9f–h, 9j) as well as alkyl substituents with CF_3_ (9c 85%) and benzyls (9d 81%, 9e 76%). *N*-Aryl substituents were also tolerated (9k–o), with electron-rich aryls (9l–n, 69–82%) performing better than electron-poor ones (9o, 49%).^28^ This trend reflects the lower nucleophilicity of the resulting carbamoyl radical with electron-withdrawing substituents.

In general, for the synthesis of secondary amides, oxidative conditions B and C both performed well. The inferior yields under conditions A in these cases are likely due to significant formation of unwanted formamide (RR′NCHO 11) side products compared to conditions B (*e.g.*^1^H NMR analysis of the crude mixture for 9c shows ∼10% formamide 11 under conditions A *vs.* >20 : 1 9 : 11 under conditions B).

The Giese amidation reaction could also be scaled up using conditions B to yield appreciable amounts of 9d, albeit with a slight drop in yield with each 5 to 8-fold increase. Product 9d was successfully formed in 72% yield at 1 mmol scale and a still synthetically useful 55% at gram (5.4 mmol) scale.^29^

**Scheme 3:**
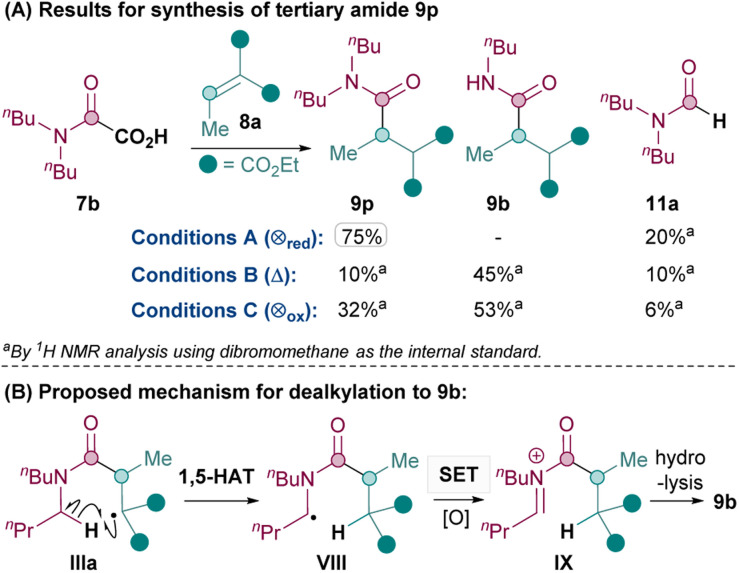
Dealkylation observed with conditions B and C for synthesis of tertiary amide.

Next, the synthesis of tertiary amides was investigated. As shown in Scheme 3A, standard oxidative conditions B and C using oxamic acid 7b surprisingly gave the dealkylated product 9b instead of the desired tertiary amide 9p as the major product. Subjecting product 9p to reaction conditions B did not result in 9b, thus ruling out dealkylation from the desired Giese products (see ESI†).^30^ Instead, we postulated that upon the conjugate addition of II to 8 to form IIIa (Scheme 2), 1,5-HAT^31^ could occur to give VIII (Scheme 3B). SET of VIII and hydrolysis of the corresponding iminium^32^IX could yield the dealkylated product 9b (Scheme 3B).^33^

Since the formation of undesired 9b requires an oxidation (VIII to IX), it was thought that exploiting the reductive quenching catalytic cycle (conditions A) should prevent the formation of 9b. Pleasingly, this hypothesis proved to be correct and conditions A successfully yielded 9p in 75% yield (Scheme 3A). It should be noted that the dealkylated side products such as 9b under oxidative conditions are only observed with tertiary amides and not secondary amides. The formation of the formamide side product 11 (from radical II) though, is generally much more prevalent with conditions A (*e.g.* 20% 11a and also 31% of the corresponding formamide was isolated along with 9q) than with standard oxidative conditions B and C (*e.g.* 10% 11a).

Thus, the Giese amidation formed tertiary amides 9p, 9q and 9r successfully in 75%, 53% and 51% respectively using reductive quenching cycle conditions A (Table 3). The amidation seemed sensitive to sterics, with 9s and 9t being formed in a moderate 38% and 41% yield respectively, although the yield of 9t was successfully improved to 55% upon more forcing conditions. Cyclic tertiary amides were produced in only moderate yields with both conditions A and B (35% and 31% 9u).^34^

Next, the Michael acceptor scope was investigated (Table 4). Since the model oxamic acid 7 chosen gave significantly better yields under conditions B in Table 3 (9k), a result that is further confirmed by direct comparison of conditions A, B and C for producing 9z, 9al and 9am, conditions B were therefore utilised for the rest of the Michael acceptor scope. Activated Michael acceptors with two electron-withdrawing groups performed well, as expected, to give 9k, 9v–x in 56–79% yields. The production of 9x from a coumarin derivative was an exception where conditions A performed better, due to competitive oxidative rearomatisation under conditions B (see ESI†). To our delight, Michael acceptors with only one electron-withdrawing group were also suitable substrates, including cyclic acceptors such as cyclopentenone (9y, 64%), cyclohexanone (9z, 75%), cycloheptanone (9aa, 74%), butenolide (9ab, 57%), pentenolide (9ac, 47%) and α,β-unsaturated amide (9ad, 54%). A substituent in the α-position of cyclohexanone was also tolerated (9ae, 59%), although the lower yield for 9af (31%) indicates that the reaction was sensitive to the alkene moiety in carvone. Cyclic Michael acceptors with the electron-withdrawing group *exo* to the ring can also be utilised (9ag, 58% and 9ah, 55%). Other acyclic acceptors reacted smoothly including diethyl maleate and diethyl fumarate, giving product 9ai in good yields (89% and 77% respectively). The EWGs need not be carbonyls, for example, diethyl vinylphosphonate and vinyl sulfones were also good substrates, furnishing 9aj, 9ak and 9al in 64%, 76% and 61% yields respectively. Nevertheless, a current limitation is that acyclic ketones such as 9am seem to react with more moderate yields (34%).^35^

**Table 4:** Michael acceptor scope^a^

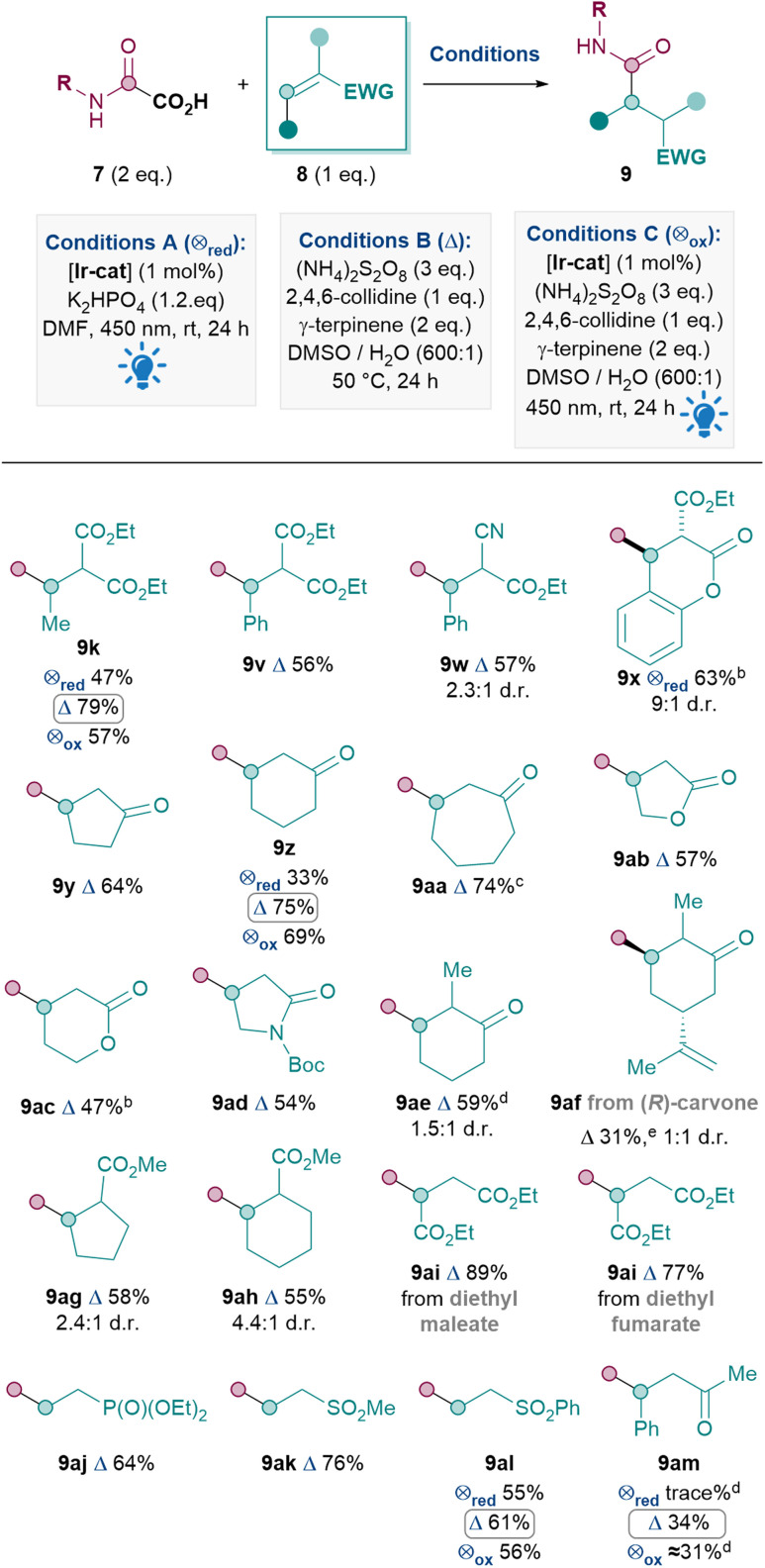

a Reactions performed on a 0.12 mmol scale of 7 under argon atmosphere, with R = Ph unless otherwise stated. [Ir-cat] = [Ir{dF(CF_3_)ppy}_2_(dtbpy)PF_6_].

b R = Cy.

c R = 1-Adamantyl.

d Yields determined by ^1^H NMR analysis of the crude mixture using dibromomethane as the internal standard.

e R = H

The Michael acceptor substrate scope has thus been significantly expanded compared to previous methods (2 and 4, Scheme 1). In particular, the ease of reaction with many endocyclic acceptors (*e.g.*9x–9ah) for the first time renders the Giese amidation applicable to various natural products and building blocks with such motifs.

Thus, the Giese amidation was successfully applied to amino acids, natural products and chiral building blocks (Table 5).^36^ Oxamic acids of alanine and valine reacted smoothly to give 9an and 9ao in 79% and 64% yields respectively, with conservation of enantiopurity for 9ao (see ESI†). More complex amines such as the natural product leelamine can also be introduced *via* the Giese amidation in good yield (9ap, 65%). The reaction can also be applied to amidate Michael acceptor natural product cryptone (9aq, 67%) and a common chiral building block^37^ (9ar, 51%). Finally, in order to challenge the system further, an attempt was made to combine a complex amine with a complex Michael acceptor. Despite the challenge, 9as was successfully formed in 35% yield.

**Table 5:** Application to amino acids, natural products and chiral building blocks.^a^

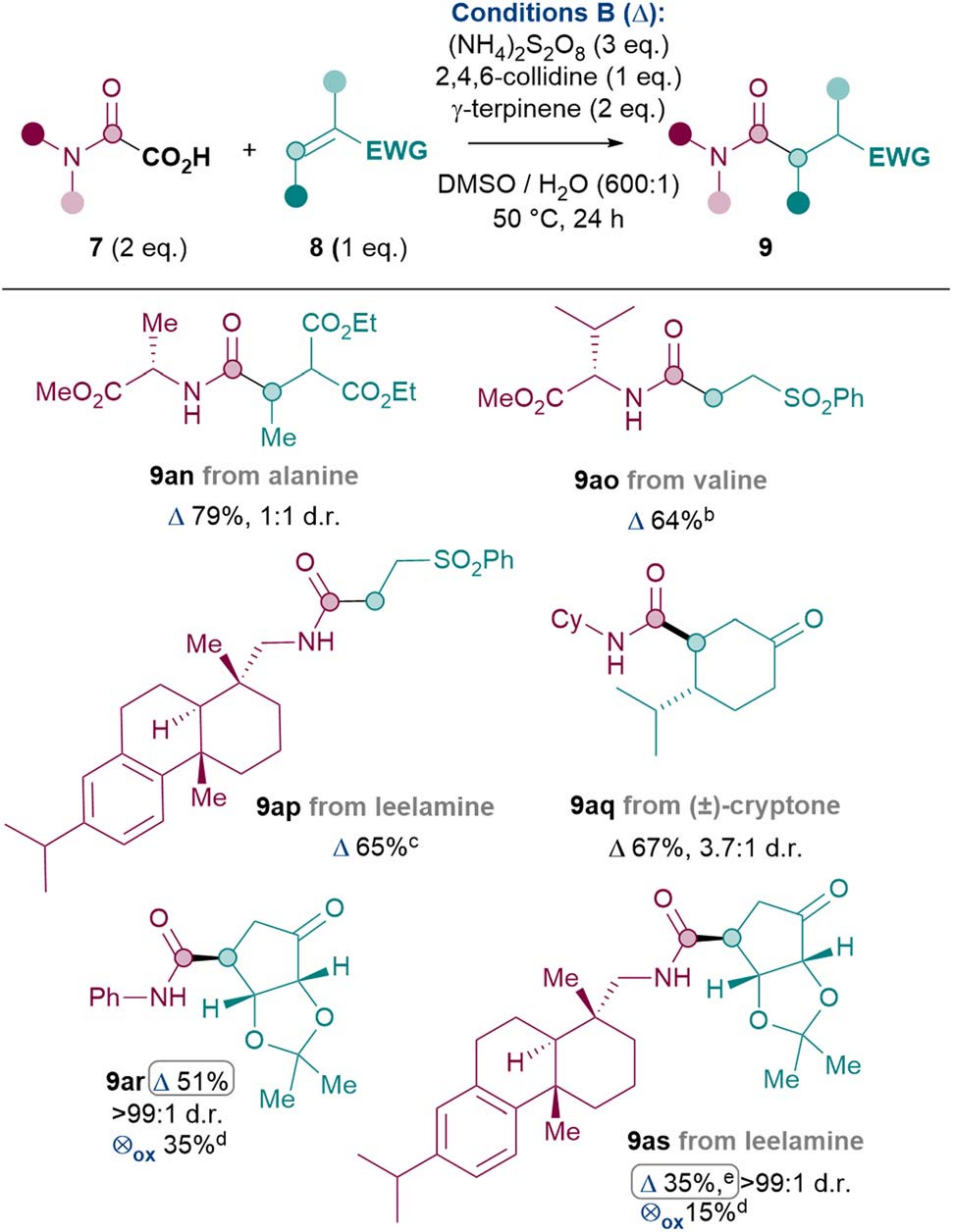

a Reactions performed on a 0.12 mmol scale of 7 under argon atmosphere unless otherwise stated.

b Reaction performed on a 0.24 mmol scale.; no racemisation of stereogenic centre observed by CSP-HPLC.

c Reaction performed on a 0.11 mmol scale.

d Yield determined by ^1^H NMR analysis using dibromomethane as internal standard. ⊗_ox_ = Cond. C.

e 3 eq. of 8, 3 eq. of 2,4,6-collidine and 4 eq. (NH_4_)_2_S_2_O_8_, 24 h at 50 °C and 24 h at 75 °C. Yield was 25% under standard conditions.

## Conclusions

We have successfully developed the first direct Giese amidation reaction from oxamic acids 7, which benefits from having a significantly better substrate scope compared to previously reported Giese amidation methods. Crucially, the ability to use the bench stable, non-toxic and environmentally benign oxamic acids 7 as the carbamoyl precursor directly for the first time greatly improves the practicality of the Giese amidation. The significantly expanded Michael acceptor substrate scope, especially the applicability of endocyclic Michael acceptors for the first time, now renders the Giese amidation applicable to natural products and chiral building blocks.

Three different conditions were developed and compared: photocatalytic reductive quenching cycle (conditions A), metal- and light-free (conditions B) and photocatalytic oxidative quenching cycle (conditions C). The methods were found to be complementary, with the flexibility provided by different conditions allowing for a more general substrate scope.

## Data availability

RAW NMR data, HRMS and IR spectra available at: DOI: 10.17861/50eb4ef7-ce19-4ac4-952d-76b7c96386c3.

## Author contributions

DMK performed the bulk of the experiments and analysed the data. KAS and ER both assisted with optimisation studies and/or part of the substrate scope studies. A-LL conceived and supervised the research. SN co-supervised the research. A-LL and DMK co-wrote the manuscript, with input from SN.

## Conflicts of interest

There are no conflicts to declare.

## Supplementary Material

SC-014-D3SC03143H-s001

SC-014-D3SC03143H-s002
