# Supplementary material for: Direct decarboxylative Giese amidations: photocatalytic vs. metal- and light-free
Source: Chem Sci. 2023 Aug 24;14(36):9806–13. doi: 10.1039/d3sc03143h (PMC10510818; doi:10.1039/d3sc03143h)
Supplement: SC-014-D3SC03143H-s001 [file SC-014-D3SC03143H-s001.pdf]

Electronic Supplementary Material (ESI) for Chemical Science.
This journal is © The Royal Society of Chemistry 2023

Supplementary Information

Direct Decarboxylative Giese Amidations: Photocatalytic *vs.* Metal- and Light-free

David M. Kitcatt,[a] Katie A. Scott,[a] Elena Rongione,[a] Simon Nicolle,[b] and Ai-Lan Lee[a,*]

[a]Institute of Chemical Sciences, Heriot-Watt University, Edinburgh EH14 4AS, Scotland, U. K.

[b]GlaxoSmithKline, Gunnels Wood Rd, Stevenage SG1 2NY, U. K.

*A.Lee@hw.ac.uk

Table of Contents

1. General Experimental S-3
2. Further controls and optimisation S-4
3. Quantum Yield Determination S-9
4. Starting material synthesis S-13
5. Product Characterisation S-23
6. References S-199

1. General Experimental

All reagents and solvents were purchased from commercially available sources and used without any further purification. ^{1}H and ^{13}C Nuclear Magnetic Resonances were recorded on Bruker® AV300 or AV400 (^{1}H NMR at 300 MHz or 400 MHz, respectively, and $^{13}C\{^{1}H\}$ NMR at 75 MHz or 101 MHz, respectively) spectrometers with chemical shifts (δ) given in parts per million (ppm), employing chloroform-*d* or acetone-d_6 as solvents with their respective residual solvent signals[1] reported as their standard reference peaks. Multiplicities are indicated as br (broad), s (singlet), d (doublet), t (triplet), q (quartet), quin (quintet), m (multiplet) or a combination of these. Coupling constants (*J*) are given in Hertz (Hz). Yields calculated by ^{1}H NMR analysis were determined using either one of these internal standards which were added after work-up: 1,3,5-trimethoxybenzene (3H, 6.08 ppm and 9H, 3.76 ppm) or dibromomethane (2H, 4.93 ppm). Note that for ^{13}C NMR characterisation, only signals that could not be differentiated by 1 d.p. were quoted to 2 d.p. High resolution mass spectrometric (HRMS) data were reported with ion mass/charge (m/z) ratios as values in atomic mass units. High-Resolution Mass Spectra were recorded under ESI conditions by the analytical services at the University of Edinburgh. Infrared spectra were obtained on Perkin-Elmer Spectrum 100 FT-IR Universal ATR Sampling Accessory, deposited neat to a diamond/ZnSe plate. Column chromatography was carried out using Matrix silica gel 60 from Fluorochem. TLC was performed using Merck silica gel 60 F254 and visualised by UV (254 nm) and/or stained using aqueous Hanessian's Stain or potassium permanganate solution. Unless otherwise stated, all reactions requiring irradiation were carried out using Penn PhD Photoreactor M2 (fan speed = 6800 rpm, light intensity = 50%, wavelength = 450 nm).

2. Further controls and optimisation

Control reaction to show that 9b does not form from 9p under oxidative Conditions B:

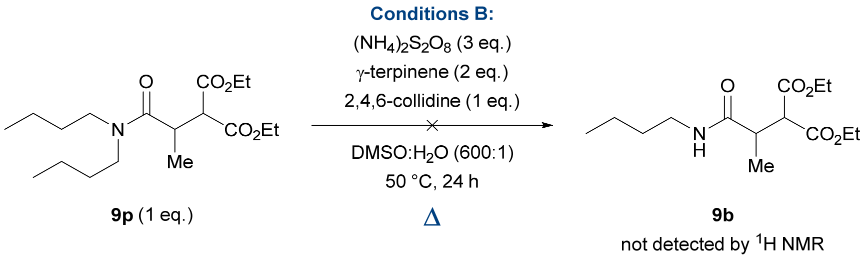

General procedure for conditions B was followed. **9p** (23.7 mg, 0.07 mmol, 1.0 eq.), $(NH_4)_2S_2O_8$ (47.5 mg, 0.21 mmol, 3.0 eq.), γ-terpinene (22.1 µL, 0.14 mmol, 2.0 eq.), and 2,4,6-collidine (9.1 µL, 0.07 mmol, 1.0 eq.) in DMSO:H_2O (600:1) (0.23 mL) was heated at 50 °C for 24 h followed by work-up Y. ^{1}H NMR analysis of the crude reaction mixture after work-up showed unreacted starting material **9p**, with no desired product **9b** present.

Reaction with TEMPO

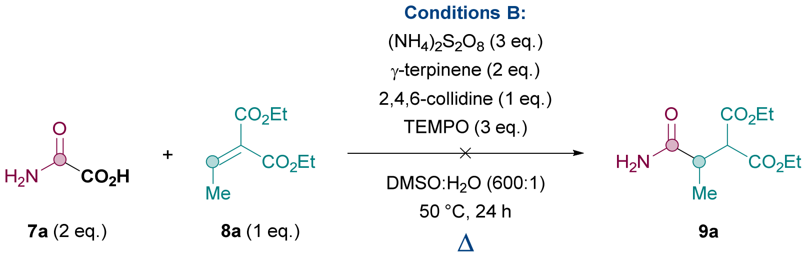

General procedure for conditions B was followed. **8a** (44.6 mg, 0.24 mmol, 1.0 eq.), **7a** (42.9 mg, 0.48 mmol, 2.0 eq.), 2 $(NH_4)_2S_2O_8$ (165.0 mg, 0.72 mmol, 3.0 eq.), γ-terpinene (76.8 µL, 0.48 mmol, 2.0 eq.), 2,4,6-collidine (31.8 µL, 0.24 mmol, 1.0 eq.) in DMSO:H_2O (600:1) (0.8 mL) with the addition of TEMPO (113.2 mg, 0.72 mmol, 3 eq.) was heated at 50 °C for 24 h followed by work-up Y. ^{1}H NMR analysis of the crude reaction mixture after work-up showed unreacted starting material **8a**, with no desired product **9a** present.

Table S1. Full optimisation studies for Conditions B[a]

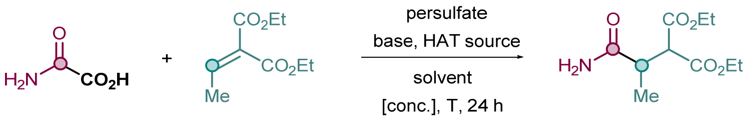

7a (x eq.) **8a** (y eq.) **9a**

Entry	x	y	Persulfate	Base	HAT source	Solvent	T (°C)	Yield (%)[b]
**1**	**2**	**1**	**$(NH_4)_2S_2O_8$ (3 eq.)**	**2,4,6-collidine (1 eq.)**	**γ-terpinene (2 eq.)**	**DMSO / H_2O (600:1)**	**50**	**96**
**2**	2	1	$(NH_4)_2S_2O_8$ (3 eq.)	2,4,6-collidine (1 eq.)	γ-terpinene (2 eq.)	DMSO / H_2O (600:1)	50	97 (in dark)
**3**	2	1	$(NH_4)_2S_2O_8$ (3 eq.)	2,4,6-collidine (1 eq.)	γ-terpinene (2 eq.)	DMSO / H_2O (600:1)	50	79 (in air)
**4**	1	2	$(NH_4)_2S_2O_8$ (3 eq.)	2,4,6-collidine (1 eq.)	γ-terpinene (2 eq.)	DMSO / H_2O (600:1)	50	43
**5**	2	1	$(NH_4)_2S_2O_8$ (1 eq.)	2,4,6-collidine (1 eq.)	γ-terpinene (2 eq.)	DMSO / H_2O (600:1)	50	72
**6**	2	1	$(NH_4)_2S_2O_8$ (5 eq.)	2,4,6-collidine (1 eq.)	γ-terpinene (2 eq.)	DMSO / H_2O (600:1)	50	62
**7**	2	1	$Na_2S_2O_8$ (3 eq.)	2,4,6-collidine (1 eq.)	γ-terpinene (2 eq.)	DMSO / H_2O (600:1)	50	14
**8**	2	1	$K_2S_2O_8$ (3 eq.)	2,4,6-collidine (1 eq.)	γ-terpinene (2 eq.)	DMSO / H_2O (600:1)	50	23
**9**	2	1	-	2,4,6-collidine (1 eq.)	γ-terpinene (2 eq.)	DMSO / H_2O (600:1)	50	n.d.
**10**	2	1	$(NH_4)_2S_2O_8$ (3 eq.)	2,6-lutidine (1 eq.)	γ-terpinene (2 eq.)	DMSO / H_2O (600:1)	50	84
**11**	2	1	$(NH_4)_2S_2O_8$ (3 eq.)	K_2HPO_4 (1 eq.)	γ-terpinene (2 eq.)	DMSO / H_2O (600:1)	50	86
**12**	2	1	$(NH_4)_2S_2O_8$ (3 eq.)	Cs_2CO_3 (1 eq.)	γ-terpinene (2 eq.)	DMSO / H_2O (600:1)	50	65
**13**	2	1	$(NH_4)_2S_2O_8$ (3 eq.)	-	γ-terpinene (2 eq.)	DMSO / H_2O (600:1)	50	55
**14**	2	1	$(NH_4)_2S_2O_8$ (3 eq.)	2,4,6-collidine (1 eq.)	γ-terpinene (1 eq.)	DMSO / H_2O (600:1)	50	79
**15**	2	1	$(NH_4)_2S_2O_8$ (3 eq.)	2,4,6-collidine (1 eq.)	γ-terpinene (3 eq.)	DMSO / H_2O (600:1)	50	73
**16**	2	1	$(NH_4)_2S_2O_8$ (3 eq.)	2,4,6-collidine (1 eq.)	1,4-CHD (2 eq.)	DMSO / H_2O (600:1)	50	65
**17**	2	1	$(NH_4)_2S_2O_8$ (3 eq.)	2,4,6-collidine (1 eq.)	Hantzsch ester (2 eq.)	DMSO / H_2O (600:1)	50	83
**18**	2	1	$(NH_4)_2S_2O_8$ (3 eq.)	2,4,6-collidine (1 eq.)	-	DMSO / H_2O (600:1)	50	36
**19**	2	1	$(NH_4)_2S_2O_8$ (3 eq.)	2,4,6-collidine (1 eq.)	γ-terpinene (2 eq.)	DMSO / H_2O (1:1)	50	84
**20**	2	1	$(NH_4)_2S_2O_8$ (3 eq.)	2,4,6-collidine (1 eq.)	γ-terpinene (2 eq.)	H_2O	50	26
**21**	2	1	**$Na_2S_2O_8$ (3 eq.)**	2,4,6-collidine (1 eq.)	γ-terpinene (2 eq.)	**H_2O**	50	24
**22**	2	1	**$K_2S_2O_8$ (3 eq.)**	2,4,6-collidine (1 eq.)	γ-terpinene (2 eq.)	**H_2O**	50	26
**23**	2	1	$(NH_4)_2S_2O_8$ (3 eq.)	**Cs_2CO_3 (1 eq.)**	γ-terpinene (2 eq.)	**H_2O**	50	32
**24**	2	1	$(NH_4)_2S_2O_8$ (3 eq.)	**Cs_2CO_3 (1 eq.)**	γ-terpinene (2 eq.)	**H_2O w/ 10 eq. DMSO**	50	<5
**25**	2	1	$(NH_4)_2S_2O_8$ (3 eq.)	2,4,6-collidine (1 eq.)	γ-terpinene (2 eq.)	Acetone	50	n.d.
**26**	2	1	$(NH_4)_2S_2O_8$ (3 eq.)	2,4,6-collidine (1 eq.)	γ-terpinene (2 eq.)	DMF	50	9

**27**	2	1	$(NH_4)_2S_2O_8$ (3 eq.)	2,4,6-collidine (1 eq.)	γ-terpinene (2 eq.)	MeCN	50	trace
**28**	2	1	$(NH_4)_2S_2O_8$ (3 eq.)	2,4,6-collidine (1 eq.)	γ-terpinene (2 eq.)	DMSO / MeCN (1:1)	50	37
**29**	2	1	$(NH_4)_2S_2O_8$ (3 eq.)	2,4,6-collidine (1 eq.)	γ-terpinene (2 eq.)	DMSO / H_2O / MeCN (1:2:3)	50	49
**30**	2	1	$(NH_4)_2S_2O_8$ (3 eq.)	2,4,6-collidine (1 eq.)	γ-terpinene (2 eq.)	DMSO / H_2O (600:1)	35	38
**31**	2	1	$(NH_4)_2S_2O_8$ (3 eq.)	2,4,6-collidine (1 eq.)	γ-terpinene (2 eq.)	DMSO / H_2O (600:1)	80	99

[a]Reactions performed on a 0.12 mmol scale of **8a**, 0.3 M and under Ar atmosphere unless otherwise stated. [b]Yields estimated by ^{1}H NMR analysis of the crude mixture using dibromomethane as the internal standard. 1,4-CHD: 1,4-cyclohexadiene. n.d.: not detected.

As described in the paper, Conditions B were inspired by our recent success with metal- and light-free Minisci reactions,[2] but it was clear from the proposed mechanism (Scheme 2B) that in addition to persulfate as oxidant used in the Minisci reactions, the addition of a base and a HAT source may be necessary for good yields.[3] Adapting the Minisci conditions[2d] with an added base (2,4,6-collidine) resulted in only 36% **9a** (Entry 18) but adding a suitable HAT source (γ-terpinene) in addition to the base immediately gave us a good result (Entry 1). Further optimisation such as modifying equivalents (Entry 4), changing the persulfate source (Entries 5-8), base (Entries 10-12), HAT source and equivalents (Entries 14-17), solvent (Entries 19-20, 25-29), a combination of changes (Entries 21-24), and temperature (Entries 30-31) failed to improve the reaction further. Control reactions in the dark show that this reaction is not light mediated (Entry 2) and persulfate (Entry 9), base (Entry 13), HAT source (Entry 18) are all necessary components for good yields. The reaction also works well in air albeit with a drop in yield (Entry 3). As described in the paper, we have previously discovered that using DMSO as the solvent allows for the breakdown of $S_2O_8^{2-}$ to the active $SO_4^{\cdot-}$ under mild conditions, without the need for metal mediation or photolysis.[2] This explains why the reaction only works well in DMSO, and we have previously also shown that yields tended to be better in "wet" DMSO, so using the 600:1 ratio of DMSO:water allows for more consistent yields regardless of the batch of DMSO used.

Table S2. Optimisation Studies and Controls for Conditions C: Photocatalytic *via* Oxidative Quenching Cycle

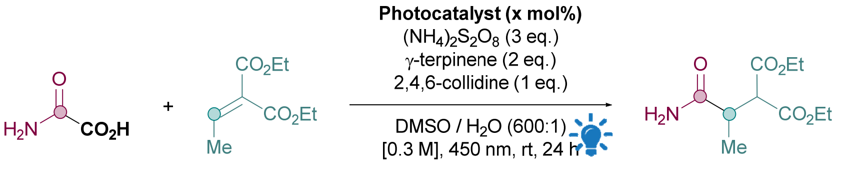

Photocatalysts used in this study

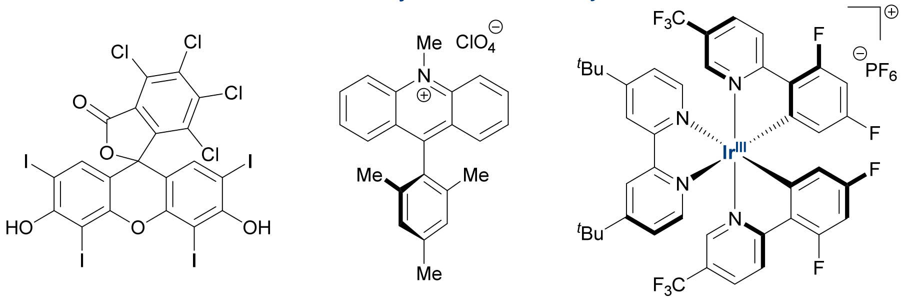

Rose Bengal **Fukuzumi catalyst** **Ir-cat**

[Ir(dF[CF$_3$]ppy)$_2$(dtbbpy)]PF$_6$

Entry	Photocatalyst	x mol%	Light intensity used (%)	Deviations for standard conditions	Yield of 9a (%)[a]
1	Ir-cat	1.0	50	-	92
2	Ir-cat	2.0	50	-	57
3	Ir-cat	1.0	100	-	62
4	Ir-cat	2.5	100	-	36
5	Ir-cat	1.0	50	48 h	67
6	Ir-cat	1.0	50	0.15 M	69
7	Fukuzumi	1.0	100	-	70
8	Fukuzumi	1.5	50	-	86
9	Fukuzumi	2.0	50	-	78
10	Fukuzumi	3.5	100	-	38
11	Rose Bengal	1.0	50	-	34
12	Ir-cat	2.0	100	No γ-terpinene	13
13	Ir-cat	1.0	50	No persulfate	22
14	-	-	50	No photocatalyst	17
15	Ir-cat	1.0	50	No 2,4,6-collidine	51
16	Ir-cat	1.0	50	In dark	15

[a]All reactions performed on 0.12 mmol scale of **8a** under Ar atmosphere in a Penn PhD M2 Photoreactor, 450 nm at 50% light intensity unless otherwise stated. Yields were determined by ^{1}H NMR using 1,3,5-trimethoxybenzene as internal standard.

Table S3. Optimisation for Synthesis of Tertiary Amides[a]

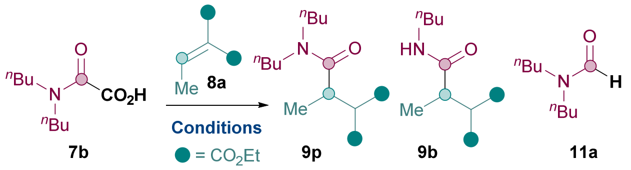

Entry	Conditions	γ-terpinene (eq.)	9p (%)[b]	9b (%)[b]	11a (%)[b]
1	B	2	10	45	10
2	B	1,4-CHD (2 eq.)	<5	66	14
3	B	Hantzsch ester (2 eq.)	<5	47	12
4	B	-	<5	69	10
5	B	10	<5	<5	<5
6	C	2	32	53	6
7	C	4	17	22	27
8	C	10	9	<5	7
9	C	-	n.d.	64	11%
**10**	**A**	**-**	**75**[c]	**-**	**20**

[a]Reactions performed on a 0.12 mmol scale of **8a** and 2 eq. **7b** under Ar atmosphere. Conditions A and B were carried out in a Penn PhD M2 Photoreactor, 450 nm at 50% light intensity. [b]Yields determined by ^{1}H NMR analysis of the crude mixture using dibromomethane as the internal standard. Yields quoted for **11a** are with respect to **7b**, whereas yields for **9p** and **9b** are quoted with respect to **8a**. [c]Isolated yield.

3. Quantum Yield Determination

General Information:

The following procedure was adapted from the literature.[4] Samples were irradiated using Penn PhD M2 Photoreactor M2 at 450 nm with 50% light intensity and fan speed = 6800 rpm.

Photon flux measurements:

a. Potassium ferrioxalate trihydrate

To a warm, stirred aqueous solution of potassium oxalate monohydrate (12 g, 65.1 mmol, 3.3 eq.) in DI water (20 mL) at 70 °C was added an aqueous solution of iron (III) chloride (3.2 g, 19.7 mmol, 1.0 eq.) in DI water (8 mL). The reaction was then cooled to rt and further cooled to 0 °C to precipitate a light green solid. This solid was then recrystallised three more times with water and left to air-dry overnight. ***Caution:*** *potassium ferrioxalate trihydrate is sensitive to light and should be kept in the dark as much as possible.*

b. 1,10-Phenanthroline buffer

An aqueous solution in DI water of sodium acetate (4.92 g, 60.0 mmol), 1,10-phenanthroline (100 mg, 0.555 mmol) and concentrated sulfuric acid (1 mL) was prepared using a 100 mL volumetric flask.

c. Determination of photon flux

A 0.15 M aqueous solution of potassium ferrioxalate trihydrate (1.47 g, 3.00 mmol) in DI water (20 mL) was prepared using a 20 mL volumetric flask. 1.0 mL of the prepared solution was transferred to a 2 mL vial and irradiated for 20 seconds. The vial was then returned to darkness. 0.5 mL of the irradiated solution was transferred to a 25 mL volumetric flask, and 5 mL of the phenanthroline buffer was added and diluted to the mark with DI water. A stirrer bar was added and the solution was stirred for 20 minutes at room temperature. 250 µL was transferred to a quartz cuvette along with DI water (2.5 mL). A UV-Vis spectrum was then obtained from 650-200 nm using the “slow” scan rate. The absorbance at 510 nm was then used to determine the

amount of Fe(II) which formed during irradiation and thereby the photon flux of the photoreactor.

d. Calculating photon flux

Step 1: Concentration of the Fe(II) in the cuvette

The photolysis gives a ligated Fe^{2+} complex that displays a characteristic absorbance peak at 510 nm. (ε = 11110 $L^{-1}cm^{-1}mol^{-1}$). The concentration of Fe^{2+} in the cuvette can be calculated using the Beer-Lambert Law:

$$A = \varepsilon l C$$

Where ε is molar absorptivity, l is pass length, and C is concentration.

Step 2: Concentration of Fe(II) upon irradiation

From the cuvette concentration calculated above, the concentration of Fe(II) in the vial after photolysis can be found using the dilution equation (two times):

$$C_1 V_1 = C_2 V_2$$

Step 3: Photon flux

The moles of incident photons can be approximated using the absolute quantum yield of Fe(II), previously found to be $\Phi_{Fe(II),\ 457.9\ nm} = 0.85$.[5] Dividing the moles of photons by the time irradiated then gives the photon flux in the units photons per second. Independent trials with irradiation times 10 s, 15 s and 20 s gave an average photon flux of 1.12 x 10^{-6} mol s^{-1} (std. dev. = 0.09 x 10^{-6}).

Table S4. Determination of photon flux

Time (s)	Absorbance at 510 nm	Photon flux (x10^{-6} mol s^{-1})
10	0.204	1.19
15	0.306	1.19
20	0.341	0.99

Example photon flux calculation (for 20 s):

Step 1: Concentration of the Fe(II) in the cuvette

$$C_{cuvette} = \frac{A}{\varepsilon l} = \frac{0.341}{(11110\ L^{-1}cm^{-1}mol^{-1})\ \times\ (1 \times 10^{-2}\ cm)} = 3.07 \times 10^{-5}\ M$$

Step 2: Concentration of Fe(II) upon irradiation

$$C_{vol\ flask} = \frac{C_{cuvette}V_{cuvette}}{V_{vol\ flask}} = \frac{(3.07 \times 10^{-5}M) \times (2.75 \times 10^{-3}L)}{0.25 \times 10^{-3}\ L} = 3.38 \times 10^{-4}\ M$$

$$C_{sample} = \frac{C_{vol\ flask}V_{vol\ flask}}{V_{sample}} = \frac{(2.26 \times 10^{-4}M) \times (25 \times 10^{-3}L)}{0.50 \times 10^{-3}\ L} = 1.69 \times 10^{-2}\ M$$

Step 3: Photon flux

$$mol\ photons = \frac{C_{sample}V_{reaction}}{\Phi_{Fe(II),457.9\ nm}} = \frac{(1.67 \times 10^{-2}M) \times (1.0 \times 10^{-3}L)}{0.85} = 1.99 \times 10^{-5}\ mol$$

$$photon\ flux = \frac{mol\ photons}{t_{irradiation}} = \frac{1.99 \times 10^{-5}\ mol}{30\ s} = 9.93 \times 10^{-7}\ mol\ s^{-1}$$

e. Determining quantum yield

The quantum yield (Φ) was determine both for Conditions A and C. The quantum yield of a reaction can be obtained by stopping the reaction at varying degrees of conversion using the following relationship:

$$\Phi = \frac{moles\ of\ product}{moles\ of\ incident\ photons} = \frac{moles\ of\ product}{photon\ flux \times reaction\ time}$$

Quantum yield for the Giese amidation under Conditions A:

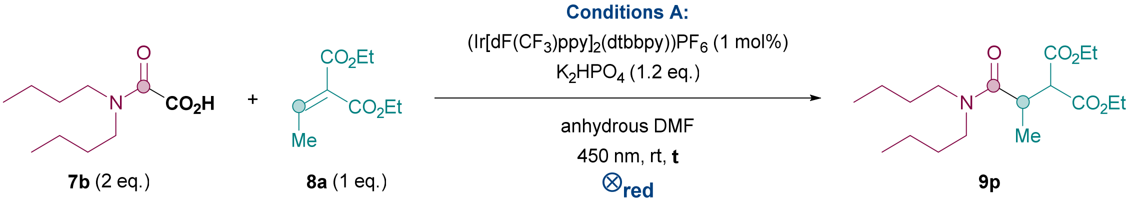

The above model reaction (0.12 mmol scale) was used to determine the quantum yield of the reaction under Conditions A. General procedure for Conditions A was followed and NMR yields were determined for reactions conducted over 2, 4, 6, and 8 hours, giving an average Φ of 2.83 x 10^{-3} (std. dev. = 0.69 x 10^{-3}).

Table S5. Determination of average quantum yield for the Giese amidation under Conditions A.

t (h)	NMR yield of 9p (%)[a]	Quantum yield Φ (x10^{-3})
2	27	4.00
4	36	2.70
6	49	2.42
8	60	0.22

[a]Yields were determine by ^1H NMR using CH_2Br_2 as internal standard.

Quantum yield for the Giese amidation under Conditions C:

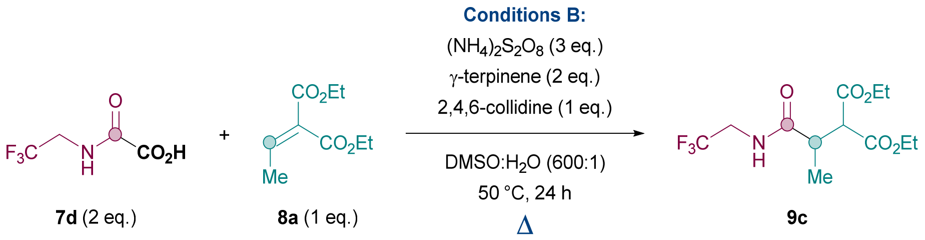

The above model reaction (0.12 mmol scale) was used to determine the quantum yield of the reaction under Conditions C. General procedure for Conditions C was followed and NMR yields were determined for reactions conducted over 0.5, 2, 4, and 6 hours, giving an average Φ of 1.15 x 10^{-2} (std. dev. = 0.88 x 10^{-2}).

Table S6. Determination of average quantum yield for the Giese amidation under Conditions C

t (h)	NMR yield of 9c (%)[a]	Quantum yield Φ (x10^{-2})
0.5	44	2.61
2	69	1.02
4	75	0.556
6	81	0.400

[a]Yields were determine by ^1H NMR using CH_2Br_2 as internal standard.

4. Starting material synthesis

Oxamic acid **7a** (Figure 1) was purchased from Sigma Aldrich. Oxamic acids **7b-u** and 7**x** were synthesised previously following the **General Procedure** described below.[2b, 2d] Amino acid oxamic acids **7v-w** were synthesised according to literature procedures.[6] All oxamic acids shown below are literature precedented apart from **7r** and **7t**; procedures and characterisation for the new oxamic acids are shown below.

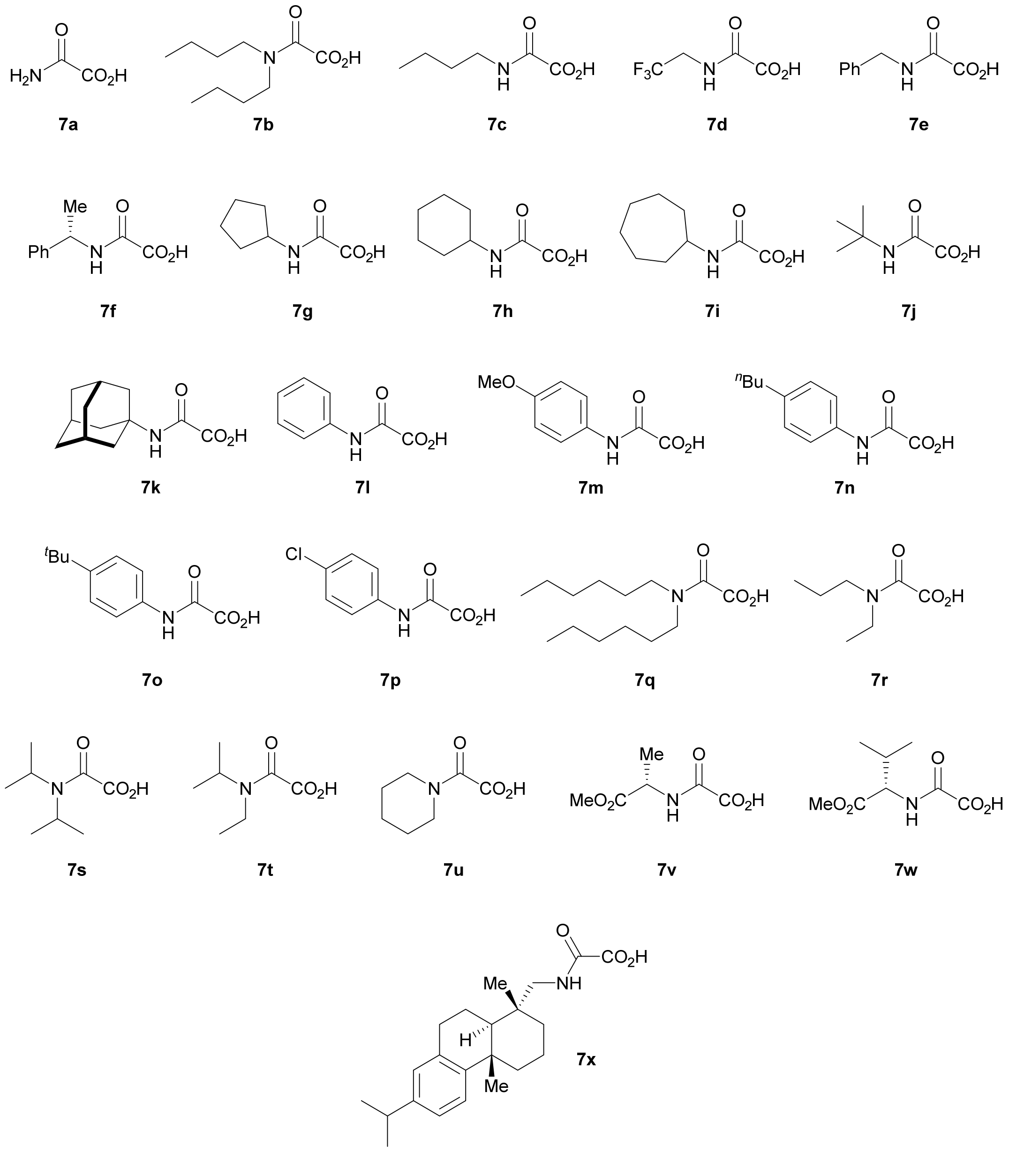

Figure 1. Oxamic acids used in this study.

All Michael acceptors (Figure 2) were purchased and used as purchased, apart from **8u** which was synthesised according to literature procedure.[7]

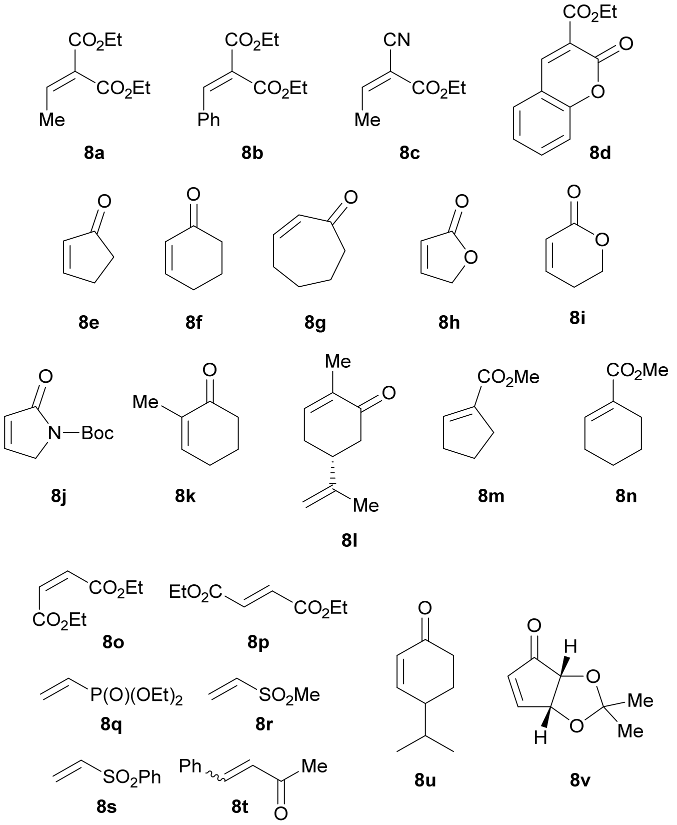

Figure 2. Michael acceptors used in this study.

Other substrates:

As described in the paper, more reactive acyclic acceptors such as acrylonitrile and methyl acrylate formed a complex mixture of products with <20% desired product observed when reacted with **7** (R=Ph). A styrene (methyl 4-vinylbenzoate) also produced a complex mixture of products when reacted with **7** (R=Ph).

General Procedure: Synthesis of Oxamic Acids[2b, 2d]

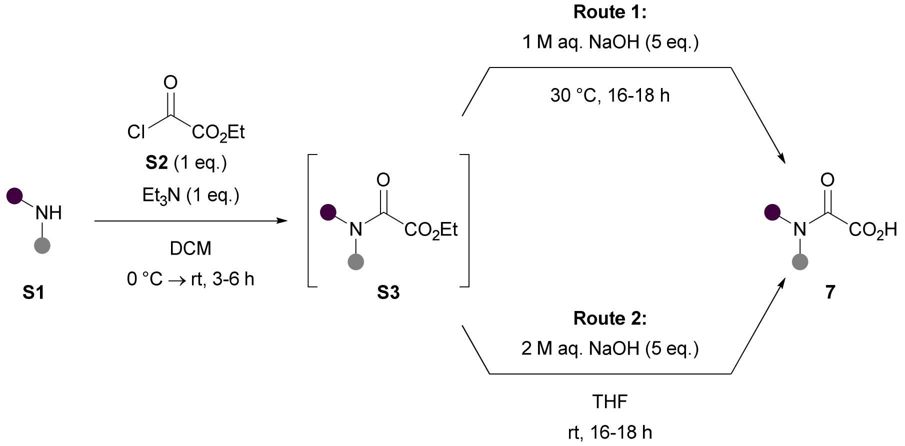

Ethyl oxalyl chloride **S2** (11 mmol, 1.1 equiv) was added dropwise to a solution of the desired amine **S1** (10 mmol, 1 equiv) and triethylamine (11 mmol, 1.1 equiv) at 0 °C. The solution was then allowed to warm to room temperature and stirred at room temperature for 3-6 h. 1 M HCl (aq.) (20 mL) was then added and the organic phase separated. The aqueous phase was then extracted with CH_2Cl_2 (3× 30 mL) and the organic phases were combined, washed with brine (70 mL), dried over $MgSO_4$ and the solvent removed *in vacuo*. The oily residue of **S3** was then carried through to the next step without further purification. Hydrolysis of crude **S3** was carried out using either Route 1 or Route 2, both of which are described below. We recommend Route 2 if Route 1 is unsuccessful.

Route 1: the crude **S3** was then dissolved in 1 M NaOH (aq.) (50 mL, 50 mmol, 5 equiv) was added and the mixture stirred overnight at 30 °C or starting material consumption was monitored *via* TLC. The mixture was then acidified (~ pH 1) with conc. HCl (aq.) followed by dilution with EtOAc (70 mL). The layers were then separated, and the aq. layer was extracted with more EtOAc (3 × 30 mL). The organic phases were then combined, washed with brine (70 mL), and dried over $MgSO_4$. The solvent was then removed *in vacuo* and the product **7** was either used without further purification or recrystallized from $CHCl_3$/hexane if further purification was required.

Route 2: The crude **S3** was then dissolved in THF (25 mL) followed by 2 M NaOH (aq.) (25 mL, 50 mmol, 5 eq.) and the mixture stirred overnight at rt or starting material consumption was monitored *via* TLC. The mixture was then acidified (~ pH 1) with conc. HCl (aq.) followed by dilution with EtOAc (70 mL). The layers were then separated, and the aq.

layer was extracted with more EtOAc (3 × 30 mL). The organic phases were then combined, washed with brine (70 mL), and dried over $MgSO_4$. The solvent was then removed *in vacuo* and the product **7** was either used without further purification or recrystallized from $CHCl_3$/hexane if further purification was required.

2-(Ethyl(propyl)amino)-2-oxoacetic acid (7s)

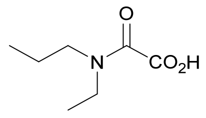

7s
Chemical Formula: $C_7H_{13}NO_3$
Molecular Weight: 159.19

General procedure for oxamic acid synthesis was followed. Ethyl oxalyl chloride (770 µL, 5.5 mmol, 1.1 eq.) was added dropwise to a solution of *N*-ethylpropan-2-amine (590 µL, 5.0 mmol, 1 eq.) and triethylamine (620 µL, 11 mmol, 1.1 eq.) in CH_2Cl_2 (15 mL) at 0 °C. The solution was then allowed to warm to room temperature and stirred at room temperature for 16 h. 1 M HCl (aq.) (10 mL) was then added and the organic phase separated. The aqueous phase was then extracted with CH_2Cl_2 (3× 15 mL) and the organic phases were combined, washed with brine (70 mL), dried over $MgSO_4$, and the solvent removed *in vacuo*. The oily residue was then carried through to the next step without further purification where Route 2 of the General Procedure was followed. The residue was then dissolved in THF (12.5 mL) followed by aq. 2 M NaOH (12.5 mL, 50 mmol, 5 eq.) and the mixture stirred overnight at rt. The mixture was then acidified (~ pH 1) with conc. HCl (aq.) followed by dilution with EtOAc (70 mL). The layers were then separated, and the aq. layer was further washed with EtOAc (3× 30 mL). The organic phases were then combined, washed with brine (70 mL), and dried over $MgSO_4$. The solvent was then removed *in vacuo* to give a 50:50 mixture of rotamers of 2-(ethyl(propyl)amino)-2-oxoacetic acid **7s** (829.5 mg, 5.0 mmol, quant.) as an off-white solid.

Characterisation:

^{1}H NMR (300 MHz, Chloroform-*d*): (50:50 mixture of rotamers) δ ppm 11.11 (s, 1H, CO_2H), 3.60 (q, *J* = 7.2 Hz, 0.6H, CH_2), 3.55 – 3.40 (m, 2H, CH_2), 3.40 – 3.29 (m, 1H, CH_2), 1.76 – 1.53 (m, 2H, CH_2), 1.25 (t, J = 7.1 Hz, 1.5H, CH_3), 1.19 (t, *J* = 7.2 Hz, 1.5H, CH_3), 0.97 – 0.86 (m, 3H, CH_3). **^{13}C NMR (101 MHz, Chloroform-*d*):** (50:50 mixture of rotamers) δ ppm 161.60 (C), 161.57 (C), 161.50 (C plus one overlapping C), 161.48 (C), 161.46 (C), 161.3 (C plus one overlapping C), 50.4 (CH_2), 48.1 (CH_2), 44.1 (CH_2), 42.0 (CH_2), 22.2 (CH_2), 20.5 (CH_2), 14.4 (CH_3), 12.3 (CH_3), 11.4 (CH_3), 11.0 (CH_3). **IR:** ν_{max}/cm^{-1}: 2964, 2942, 2879, 2360, 1948, 1732, 1589, 1505, 1469, 1449, 1373, 1296, 1235, 1206, 1151. **HRMS (ESI-TOF):** *m*/*z* $[M + H]^+$ calcd for $C_7H_{14}NO_3$, 160.09682; found, 160.0975. **m.p.** 92-94 °C.

Compound 7s – ^{1}H NMR (300 MHz, Chloroform-*d*): (50:50 mixture of rotamers)

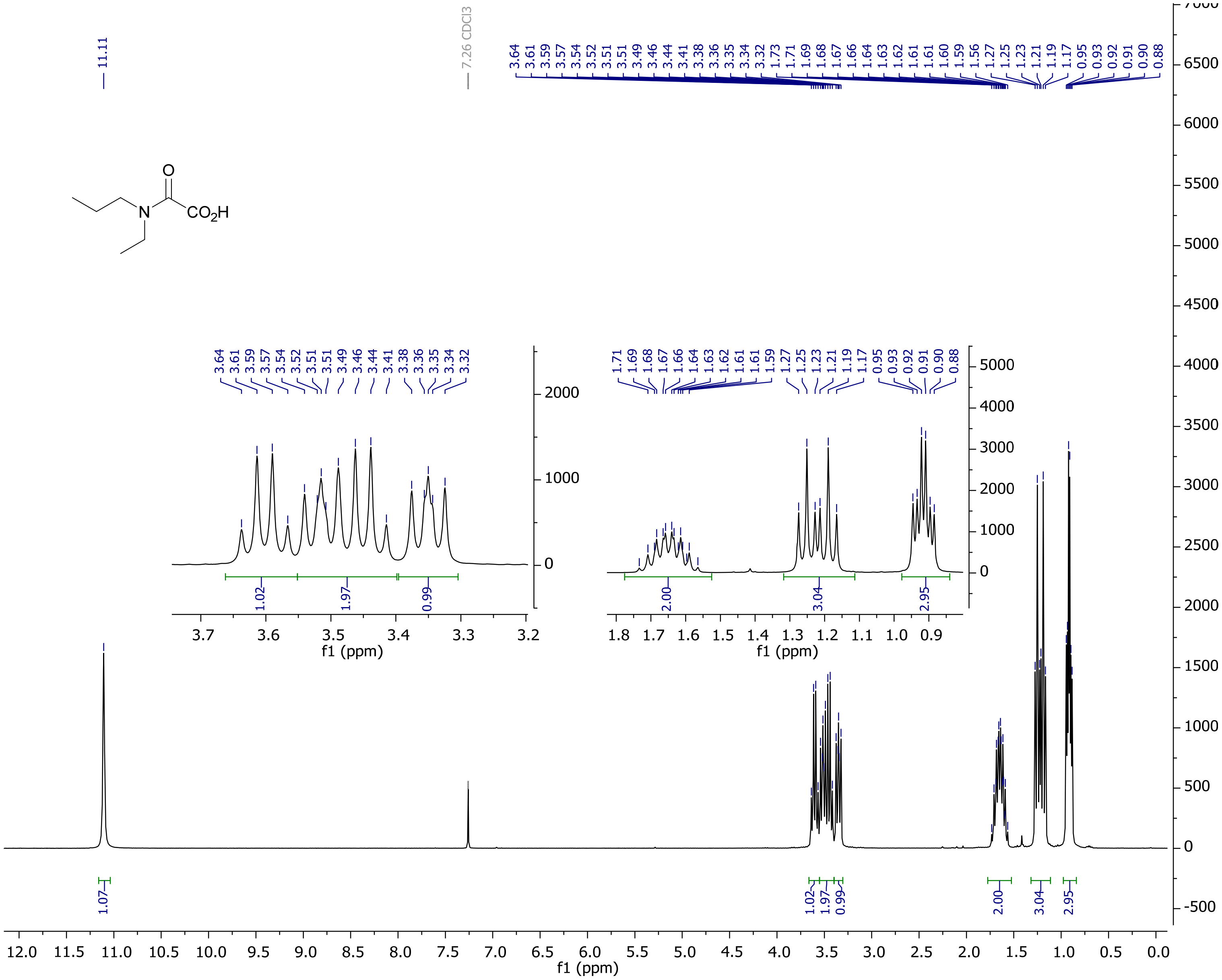

Compound 7s – ^{13}C NMR (101 MHz, Chloroform-*d*): (50:50 mixture of rotamers)

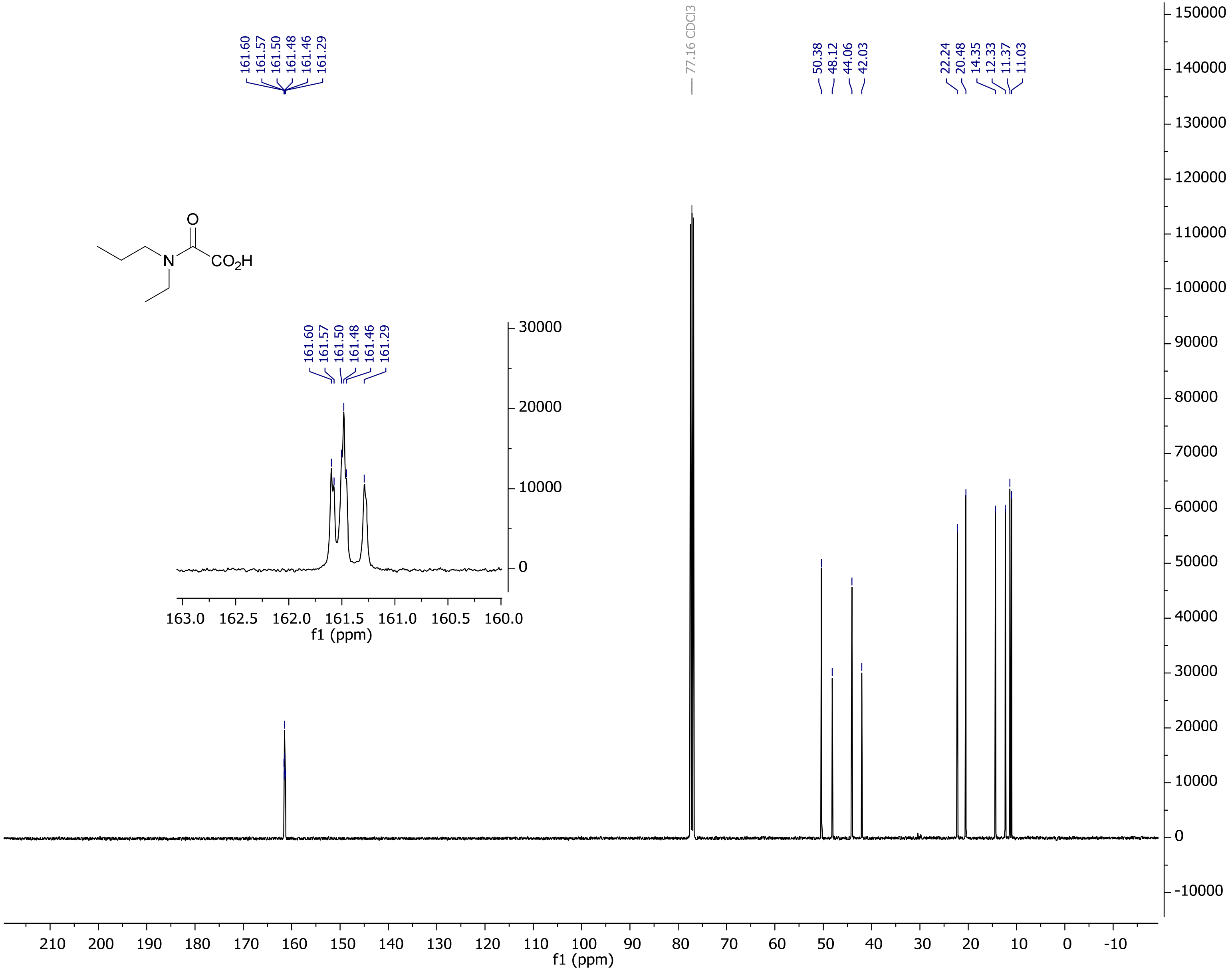

2-(Ethyl(*i*-propyl)amino)-2-oxoacetic acid (7u)

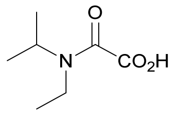

7u

Chemical Formula: $C_7H_{13}NO_3$

Molecular Weight: 159.19

General procedure for oxamic acid synthesis was followed. Ethyl oxalyl chloride (770 µL, 5.5 mmol, 1.1 eq.) was added dropwise to a solution of *N*-ethylpropan-2-amine (600 µL, 5 mmol, 1 eq.) and triethylamine (620 µL, 11 mmol, 1.1 eq.) in CH_2Cl_2 (15 mL) at 0 °C. The solution was then allowed to warm to room temperature and stirred at room temperature for 5 h. 1 M HCl (aq.) (10 mL) was then added and the organic phase separated. The aqueous phase was then extracted with CH_2Cl_2 (3× 15 mL) and the organic phases were combined, washed with brine (70 mL), dried over $MgSO_4$, and the solvent removed *in vacuo*. The oily residue was then carried through to the next step without further purification where Route 2 of the General Procedure was followed. The residue was then dissolved in THF (12.5 mL) followed by aq. 2 M NaOH (12.5 mL, 50 mmol, 5 eq.) and the mixture stirred overnight at rt. The mixture was then acidified (~ pH 1) with conc. HCl (aq.) followed by dilution with EtOAc (70 mL). The layers were then separated, and the aq. layer was further washed with EtOAc (3× 30 mL). The organic phases were then combined, washed with brine (70 mL), and dried over $MgSO_4$. The solvent was then removed *in vacuo* to afford a 58:42 mixture of rotamers of 2-(ethyl(*i*-propyl)amino)-2-oxoacetic acid **7u** (806.0 mg, 5.0 mmol, quant.) as an off-white solid.

Characterisation:

^{1}H NMR (300 MHz, Chloroform-*d*): (58:42 mixture of rotamers) δ ppm 10.68 (br s, 1H, CO_2H), 4.51 (dp, *J* = 13.6, 6.7 Hz, 1H, CHNH), 3.59 (q, *J* = 7.1 Hz, 0.8H, CH_2 minor), 3.36 (q, *J* = 7.1 Hz, 1.2H, CH_2 major), 1.34 – 1.18 (m, 9H, 3 x CH_3). **^{13}C NMR (101 MHz, Chloroform-*d*):** (58:42 mixture of rotamers) δ ppm 162.5 (C), 162.4 (C), 162.0 (C), 161.9 (C), 161.31 (C), 161.28 (C), 161.05 (C), 161.00 (C), 50.5 (CH), 48.8 (CH), 40.1 (CH_2), 36.5 (CH_2), 21.2 (CH_3), 20.0 (CH_3), 16.6 (CH_3), 14.1 (CH_3). **IR:** ν_{max}/cm^{-1}: 2993, 2980, 2944, 2324, 1967, 1733, 1575, 1557, 1495, 1436, 1369, 1242. **HRMS (ESI-TOF):** *m*/*z* $[M + H]^+$ calcd for $C_7H_{14}NO_3$, 160.09682; found, 160.0972. **m.p.** 108-112 °C.

Compound 7u – ^{1}H NMR (300 MHz, Chloroform-*d*): (58:42 mixture of rotamers)

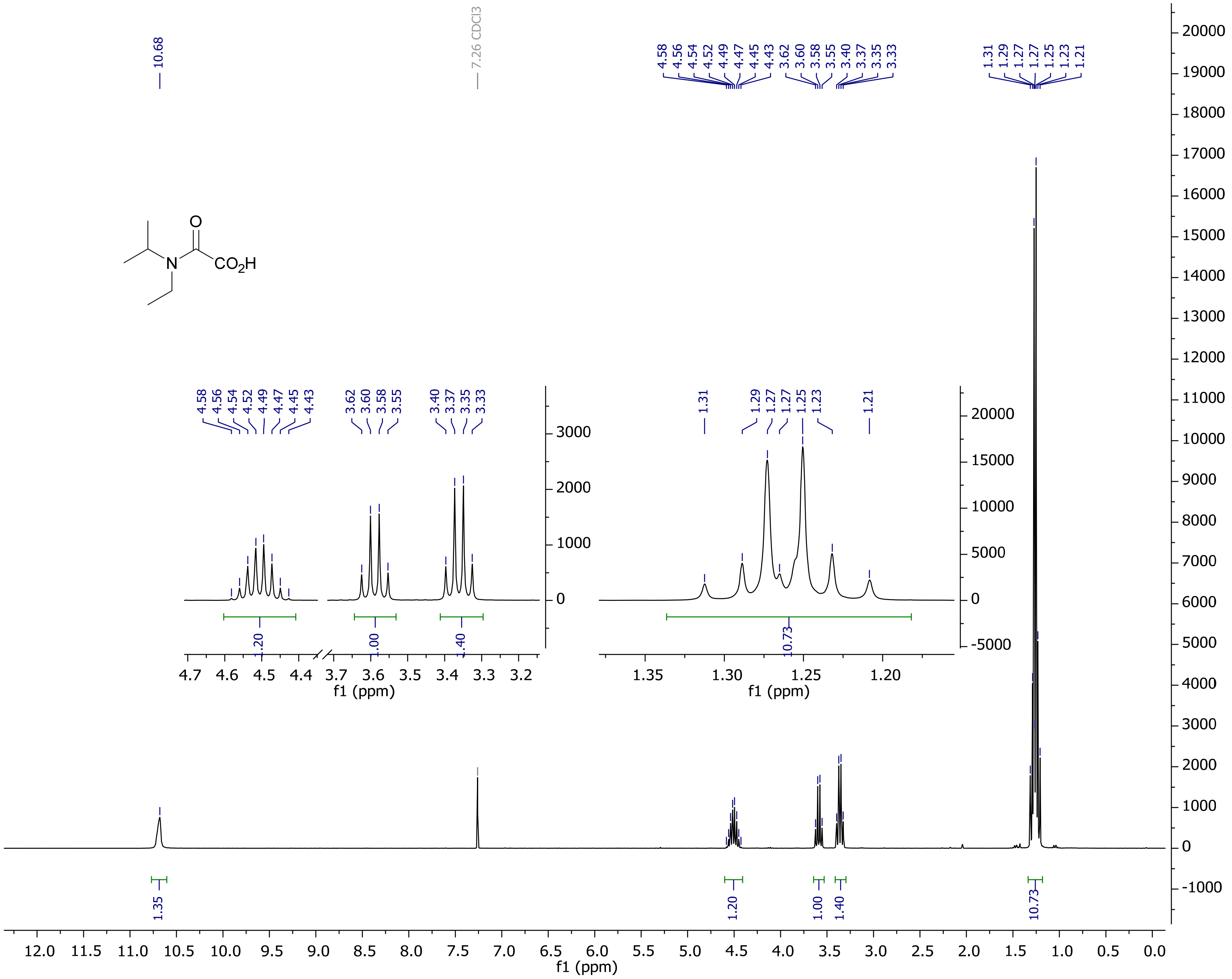

Compound 7u – ^{13}C NMR (101 MHz, Chloroform-*d*): (58:42 mixture of rotamers)

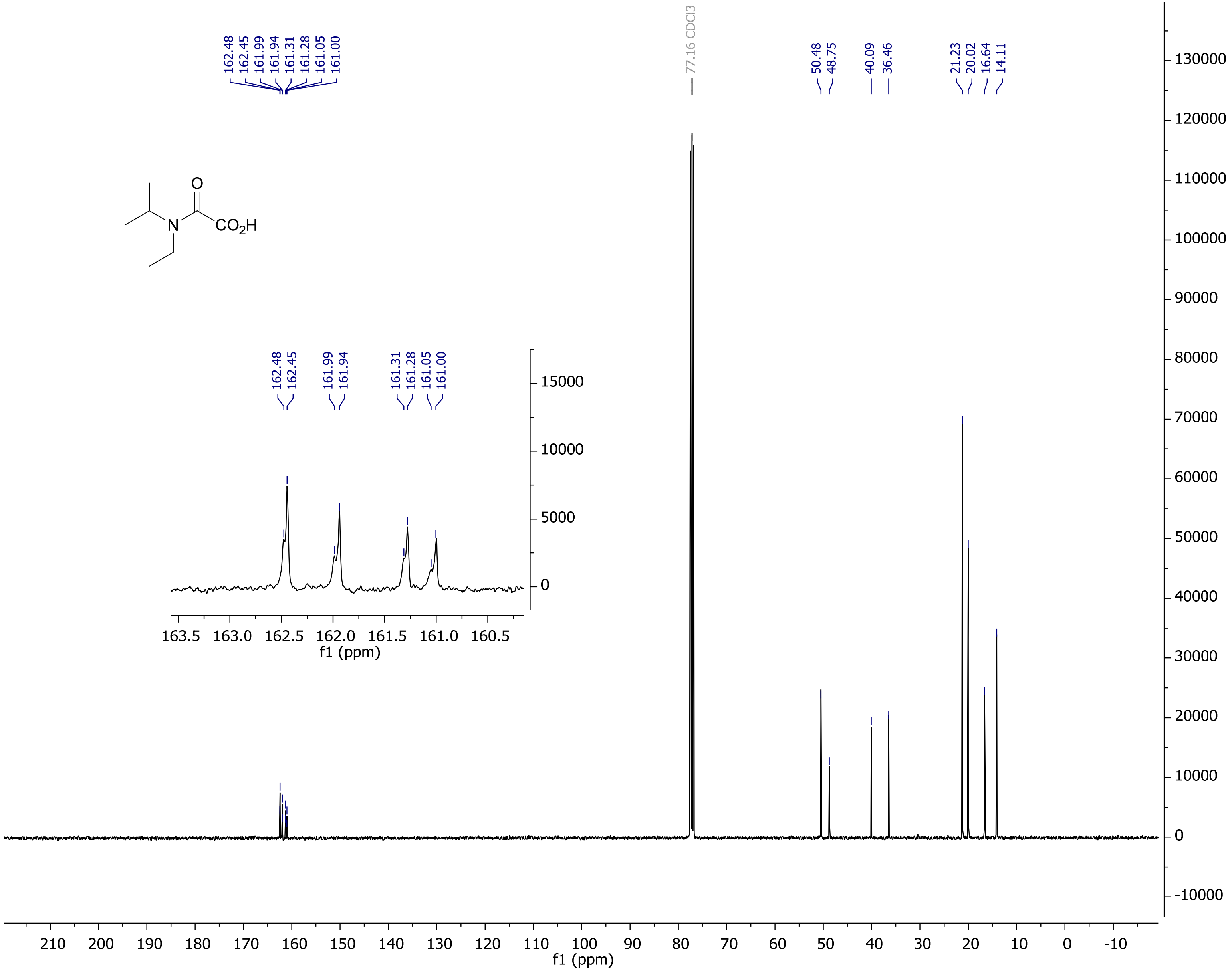

5. Product Characterisation

General Procedures for Giese Amidation Reactions

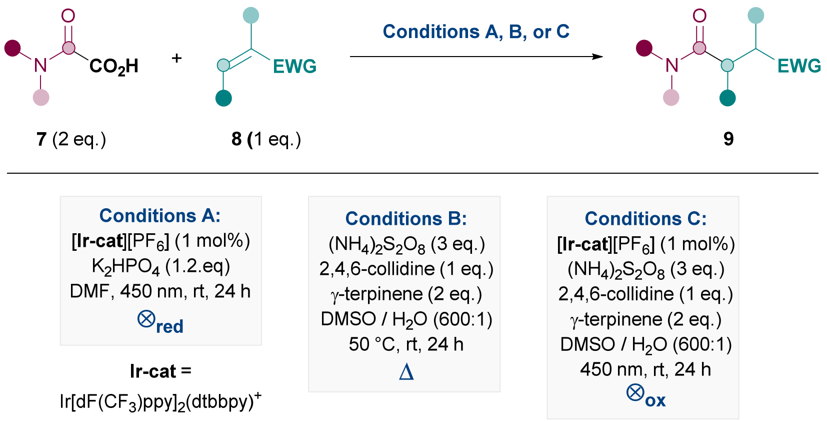

General Procedure for Conditions A: To an oven-dried 4 mL vial equipped with a magnetic stirrer bar was added Michael acceptor (1 eq.), oxamic acid (2 eq.), (Ir[dF(CF_3)ppy]$_2$(dtbbpy))PF_6 (1 mol%), and K_2HPO_4 (1.2 eq.). Separately, a flask containing anhydrous DMF over 4 Å molecular sieves was sparged with argon (balloon) for 15-20 min. Anhydrous DMF [0.4 M] was then transferred to the 4 mL vial, and the resulting solution was sparged with argon for 5 min. The vial was quickly sealed tight, and the reaction mixture was irradiated with 450 nm blue LEDs for 24 h at rt with continuous stirring. The reaction mixture was then diluted with CH_2Cl_2 (15 mL) and washed with sat. $NaHCO_3$ solution (50 mL). The aqueous phase was then extracted with CH_2Cl_2 (3 x 10 mL). The combined organic layers were then washed with brine (60 mL), dried over $MgSO_4$, and concentrated *in vacuo* to give the crude product.

General Procedure for Conditions B: To an oven-dried 4 mL vial equipped with a magnetic stirrer bar was added Michael acceptor (1 eq.), oxamic acid (2 eq.), $(NH_4)_2S_2O_8$ (3 eq.), γ-terpinene (2 eq.), and 2,4,6-collidine (1 eq.). Separately, a Schlenk tube containing a 600:1 DMSO:H_2O solvent mixture was sparged with argon (balloon) for 15-20 min. The 600:1 DMSO:H_2O mixture [0.3 M] was then transferred to the 4 mL vial, and the resulting solution was sparged with argon for 5 min. The vial was quickly sealed tight and stirred at 50 °C for 24 h with continuous stirring. The reaction mixture was then diluted with CH_2Cl_2 (15 mL) and subjected to either Work-up X or Y.

Work-up X: The diluted solution was washed with sat. $NaHCO_3$ solution (50 mL) and the aqueous phase was then extracted with CH_2Cl_2 (3 x 10 mL). The combined organic layers were then washed with brine (60 mL), dried over $MgSO_4$, and concentrated *in vacuo* to give the crude product.

Work-up Y: The diluted solution was washed with sat. $NaHCO_3$ solution (50 mL) and the aqueous phase was then extracted with CH_2Cl_2 (3 x 10 mL). The combined organic layers were then washed with 1 M HCl (60 mL), and the aq. later was extracted with more CH_2Cl_2 (20 mL). The combined organic layers were then dried over $MgSO_4$ and concentrated *in vacuo* to give the crude product.

General Procedure for Conditions C: To an oven-dried 4 mL vial equipped with a magnetic stirrer bar was added Michael acceptor (1 eq.), oxamic acid (2 eq.), $(Ir[dF(CF_3)ppy]_2(dtbbpy))PF_6$ (1 mol%), $(NH_4)_2S_2O_8$ (3 eq.), γ-terpinene (2 eq.), and 2,4,6-collidine (1 eq.). Separately, a Schlenk tube containing a 600:1 DMSO:H_2O solvent mixture was sparged with argon (balloon) for 15-20 min. The 600:1 DMSO:H_2O mixture [0.3 M] was then transferred to the 4 mL vial, and the resulting solution was sparged with argon for 5 min. The vial was quickly sealed tight, and the reaction mixture was irradiated with 450 nm blue LEDs for 24 h at rt with continuous stirring. The reaction mixture was then diluted with CH_2Cl_2 (15 mL) and subjected to work-up X shown above.

Diethyl 2-(1-amino-1-oxopropan-2-yl)malonate (9a)

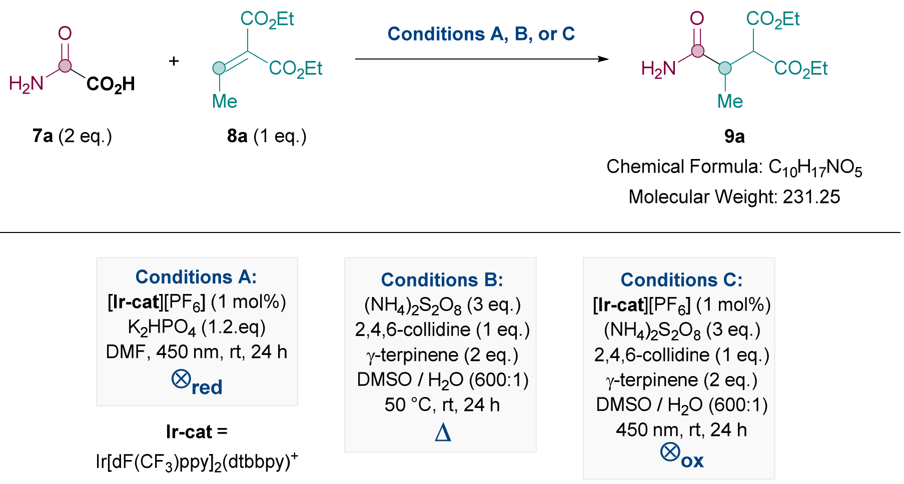

Using Conditions A: General procedure for Conditions A was followed. Diethyl 2-ethylidenemalonate **8a** (22.4 mg, 0.12 mmol, 1.0 eq.), oxamic acid **9a** (21.6 mg, 0.24 mmol, 2.0 eq.), K_2HPO_4 (25.3 mg, 0.14 mmol, 1.2 eq.), Ir[dF(CF_3)ppy](dtbppy)PF_6 (1.3 mg, 1 µmol, 1 mol%) in dry DMF (0.3 mL) was irradiated with 450 nm for 24 h. The yield of diethyl 2-(1-amino-1-oxopropan-2-yl)malonate **9a** was determined by ^{1}H NMR analysis of the crude product using CH_2Br_2 as the internal standard (34%).

Using Conditions B: General procedure for Conditions B was followed. Diethyl 2-ethylidenemalonate **9a** (22.4 mg, 0.12 mmol, 1.0 eq.), oxamic acid **7a** (21.5 mg, 0.24 mmol, 2.0 eq.), $(NH_4)_2S_2O_8$ (82.7 mg, 0.36 mmol, 3.0 eq.), γ-terpinene (38.5 µL, 0.24 mmol, 2.0 eq.), and 2,4,6-collidine (15.9 µL, 0.12 mmol, 1.0 eq.) in DMSO:H_2O (600:1) (0.4 mL) was heated at 50 ºC for 24 h followed by work-up X. The crude was then purified *via* column flash chromatography eluting with 60:40 hexane:EtOAc with 2.5% MeOH added. The solvent was then removed *in vacuo* to afford diethyl 2-(1-amino-1-oxopropan-2-yl)malonate **9a** as an off-white solid (20.9 mg, 0.090 mmol, 75%).

Using Conditions C: General procedure for Conditions C was followed. Diethyl 2-ethylidenemalonate **8a** (21.9 mg, 0.12 mmol, 1.0 eq.), oxamic acid **7a** (26.3 mg, 0.26 mmol, 2.2 eq.), $(NH_4)_2S_2O_8$ (82.2 mg, 0.36 mmol, 3.0 eq.), Ir[dF(CF_3)ppy](dtbppy)PF_6 (1.0 mg, 1 µmol, 1 mol%), γ-terpinene (38.5 µL, 0.24 mmol, 2.0 eq.), and 2,4,6-collidine (15.9 µL, 0.12 mmol, 1.0 eq.) in DMSO:H_2O (600:1) (0.4 mL) was irradiated with 450 nm for 24 h. The crude was then purified *via* column flash chromatography eluting with 60:40 hexane:EtOAc with

2.5% MeOH. The solvent was then removed *in vacuo* to afford diethyl 2-(1-amino-1-oxopropan-2-yl)malonate **9a** as an off-white solid (17.1 mg, 0.076 mmol, 63%).

Characterisations:

^{1}H NMR (400 MHz, Chloroform-*d*): δ ppm 5.94 (br s, 1H, NH), 5.64 (br s, 1H, NH), 4.25 – 4.08 (m, 4H, 2 x OCH_2), 3.72 (d, *J* = 10.0 Hz, 1H, C<u>H</u>($CO_2Et)_2$), 3.03 (dq, *J* = 10.1, 7.1 Hz, 1H, MeC<u>H</u>), 1.27 (t, *J* = 7.0 Hz, 3H, $OCH_2C\underline{H}_3$), 1.22 (t, *J* = 7.1 Hz, 3H, $OCH_2C\underline{H}_3$), 1.22 (d, *J* = 7.1 Hz, 3H, $CHC\underline{H}_3$). **^{13}C NMR (101 MHz, Chloroform-*d*):** δ ppm 176.5 (C), 168.6 (C), 168.5 (C), 61.84 (CH_2), 61.80 (CH_2), 54.9 (CH), 40.0 (CH), 16.2 (CH_3), 14.2 (CH_3), 14.1 (CH_3). **IR:** ν_{max}/cm^{-1} 3416, 3317, 3215, 2980, 2937, 1752, 1718, 1668, 1621, 1467, 1391. **HRMS (ESI-TOF):** *m/z* $[M + H]^+$ calcd for $C_{10}H_{17}NO_5$, 232.11795; found, 232.11850. **TLC:** R_f = 0.20 (40:60 hexane:EtOAc). **m.p.** 83-86 °C.

Compound 9a – ^{1}H NMR (400 MHz, Chloroform-*d*):

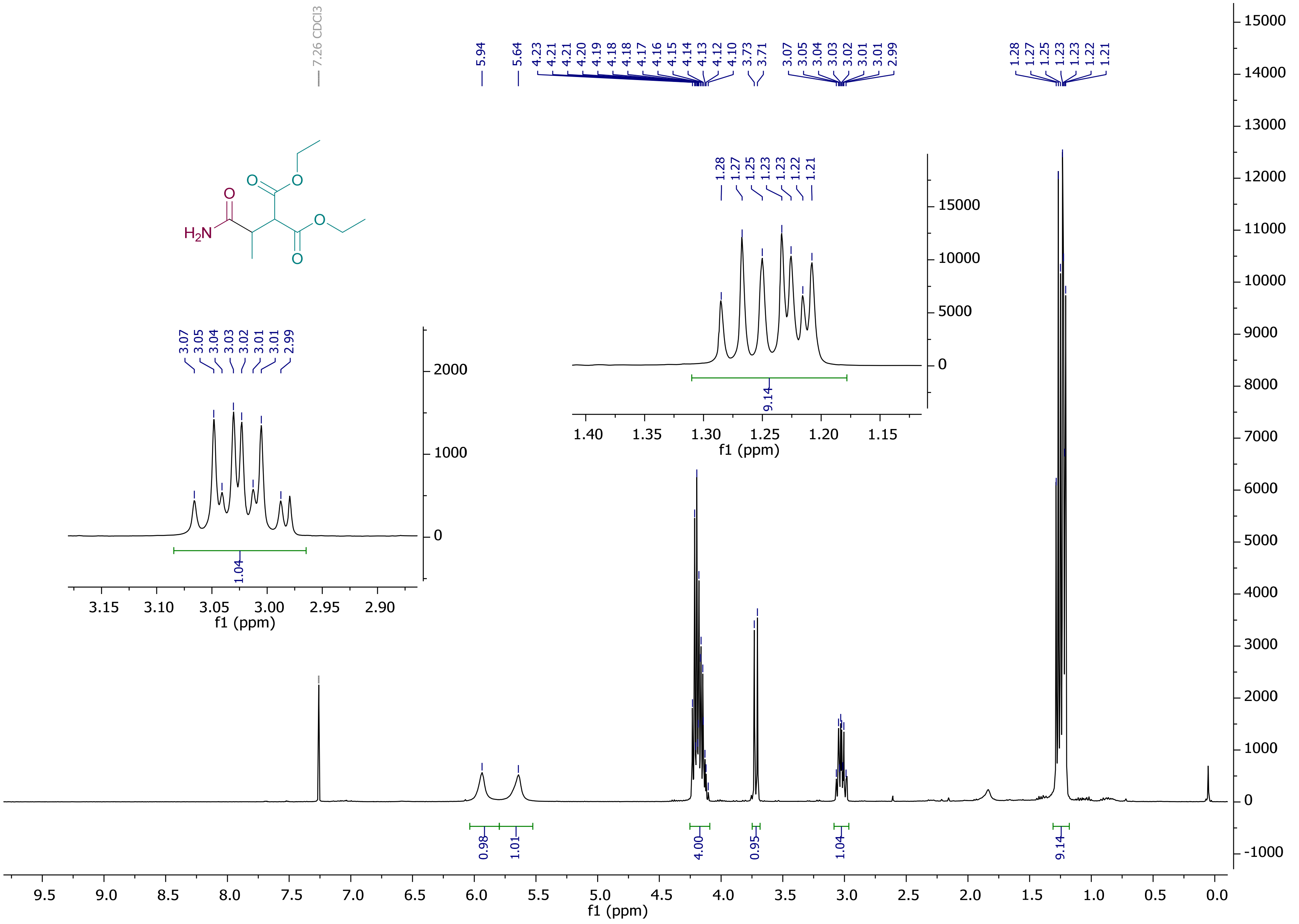

Compound 9a – ^{13}C NMR (101 MHz, Chloroform-*d*):

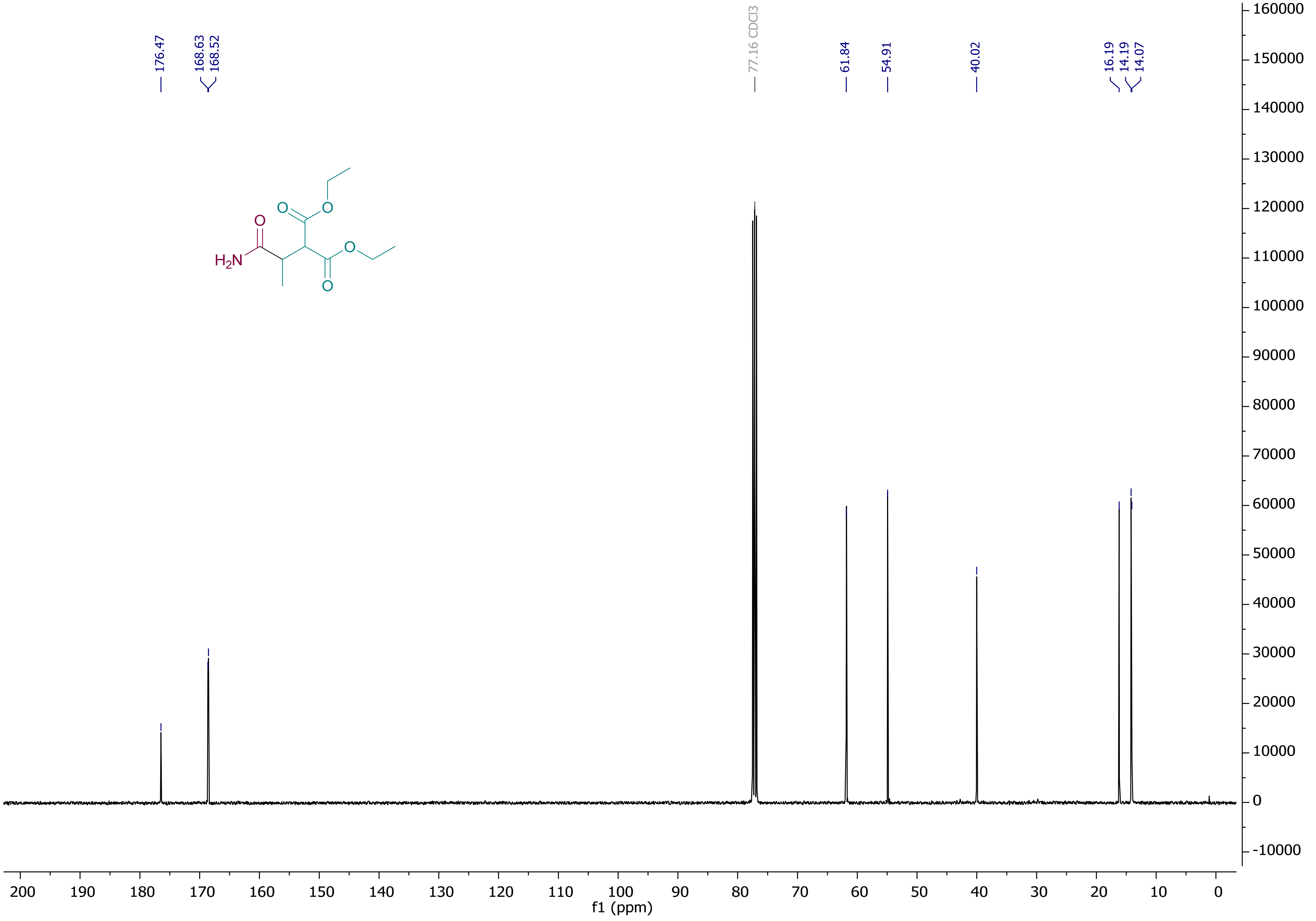

Diethyl 2-(1-(butylamino)-1-oxopropan-2-yl)malonate (9b)

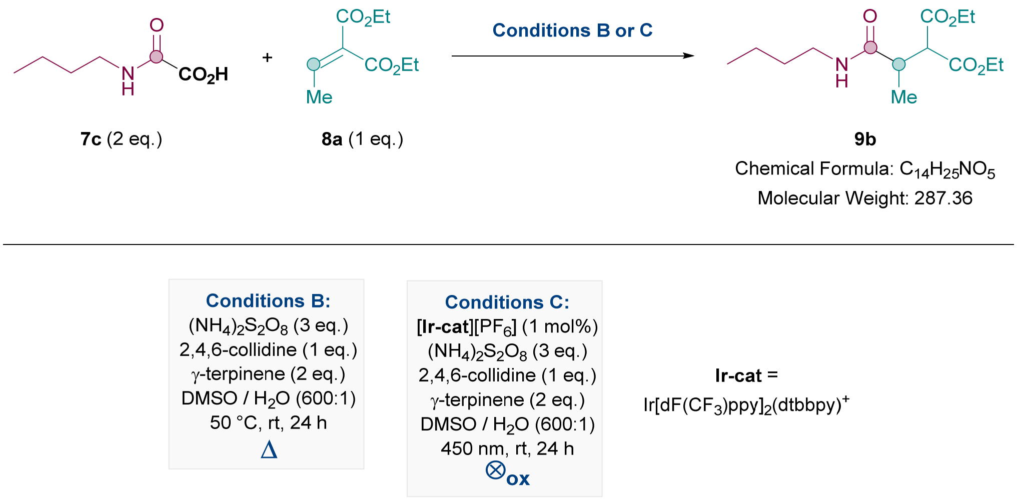

b

Using Conditions B: General procedure for Conditions B was followed. Diethyl 2-ethylidenemalonate **8a** (22.4 mg, 0.12 mmol, 1.0 eq.), *N*-butyl-oxoacetic acid **7c** (35.1 mg, 0.24 mmol, 2.0 eq.), $(NH_4)_2S_2O_8$ (82.3 mg, 0.36 mmol, 3.0 eq.), γ-terpinene (38.5 µL, 0.24 mmol, 2.0 eq.), and 2,4,6-collidine (15.9 µL, 0.12 mmol, 1.0 eq.) in DMSO:H_2O (600:1) (0.4 mL) was heated at 50 °C for 24 h followed by work-up X. The crude was then purified *via* column flash chromatography eluting with 70:30 hexane:EtOAc. The solvent was then removed *in vacuo* to afford diethyl 2-(1-(butylamino)-1-oxopropan-2-yl)malonate **9b** as a colourless oil (18.6 mg, 0.065 mmol, 54%).

Using Conditions C: General procedure for Conditions C was followed. Diethyl 2-ethylidenemalonate **8a** (23.2 mg, 0.12 mmol, 1.0 eq.), *N*-butyl-oxoacetic acid **7c** (35.0 mg, 0.24 mmol, 2.0 eq.), Ir[dF(CF_3)ppy](dtbppy)PF_6 (1.3 mg, 1 µmol, 1 mol%), $(NH_4)_2S_2O_8$ (82.7 mg, 0.36 mmol, 3.0 eq.), γ-terpinene (38.5 µL, 0.24 mmol, 2.0 eq.), and 2,4,6-collidine (15.9 µL, 0.12 mmol, 1.0 eq.) in DMSO:H_2O (600:1) (0.4 mL) was irradiated with 450 nm for 24 h. The crude was then purified *via* column flash chromatography eluting with 60:40 hexane:EtOAc. The solvent was then removed *in vacuo* to afford diethyl 2-(1-(butylamino)-1-oxopropan-2-yl)malonate **9b** as an off-white solid (22.3 mg, 0.76 mmol, 63%).

Characterisation:

^{1}H NMR (300 MHz, Chloroform-*d*): δ ppm 5.77 (br t, *J* = 5.3 Hz, 1H, NH), 4.23 – 4.07 (m, 4H, 2 x OCH_2), 3.73 (d, *J* = 10.0 Hz, 1H, C<u>H</u>(CO_2Et)$_2$), 3.22 (td, *J* = 7.1, 5.8 Hz, 2H, C<u>H</u>$_2$NH), 2.90 (dq, *J* = 10.0, 7.0 Hz, 1H, C<u>H</u>Me), 1.52 – 1.42 (m, 2H, C<u>H</u>$_2$$CH_2$NH), 1.39 – 1.31 (m, 2H, CH_3C<u>H</u>$_2$), 1.26 (t, *J* = 7.1 Hz, 3H, OCH_2C<u>H</u>$_3$), 1.22 (t, *J* = 7.1 Hz, 3H, OCH_2C<u>H</u>$_3$), 1.18 (d, *J* = 7.1 Hz, 3H, CHC<u>H</u>$_3$), 0.90 (t, *J* = 7.2 Hz, 3H, CH_2C<u>H</u>$_3$). **^{13}C NMR (75 MHz, Chloroform-*d*):** δ ppm 173.8 (C), 168.7 (C), 168.6 (C), 61.7 (CH_2), 55.0 (CH), 40.8 (CH), 39.5 (CH_2), 31.7 (CH_2), 20.1 (CH_2), 16.3 (CH_3), 14.2 (CH_3), 14.1 (CH_3), 13.8 (CH_3). **IR:** ν_{max}/cm^{-1} 3306, 2961, 2936, 2874, 2357, 1750, 1734, 1645, 1554, 1464, 1368, 1301. **HRMS (ESI-TOF):** *m*/*z* [M + H]$^+$ calcd for $C_{14}H_{25}NO_5$, 288.18055; found, 288.1810. **TLC:** R_f = 0.28 (70:30 hexane:EtOAc).

Compound 9b – ^{1}H NMR (300 MHz, Chloroform-*d*):

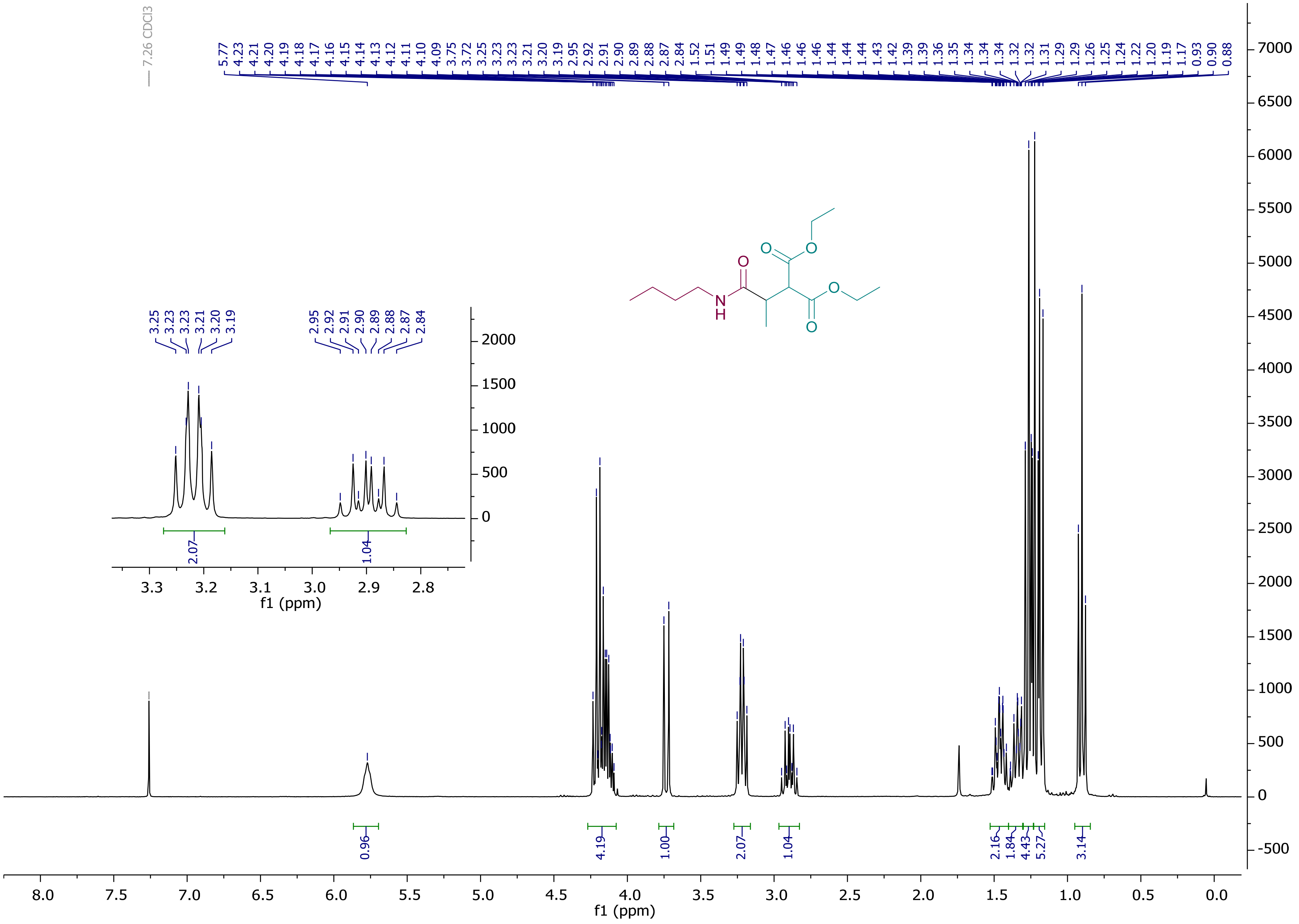

Compound 9b – ^{13}C NMR (75 MHz, Chloroform-*d*):

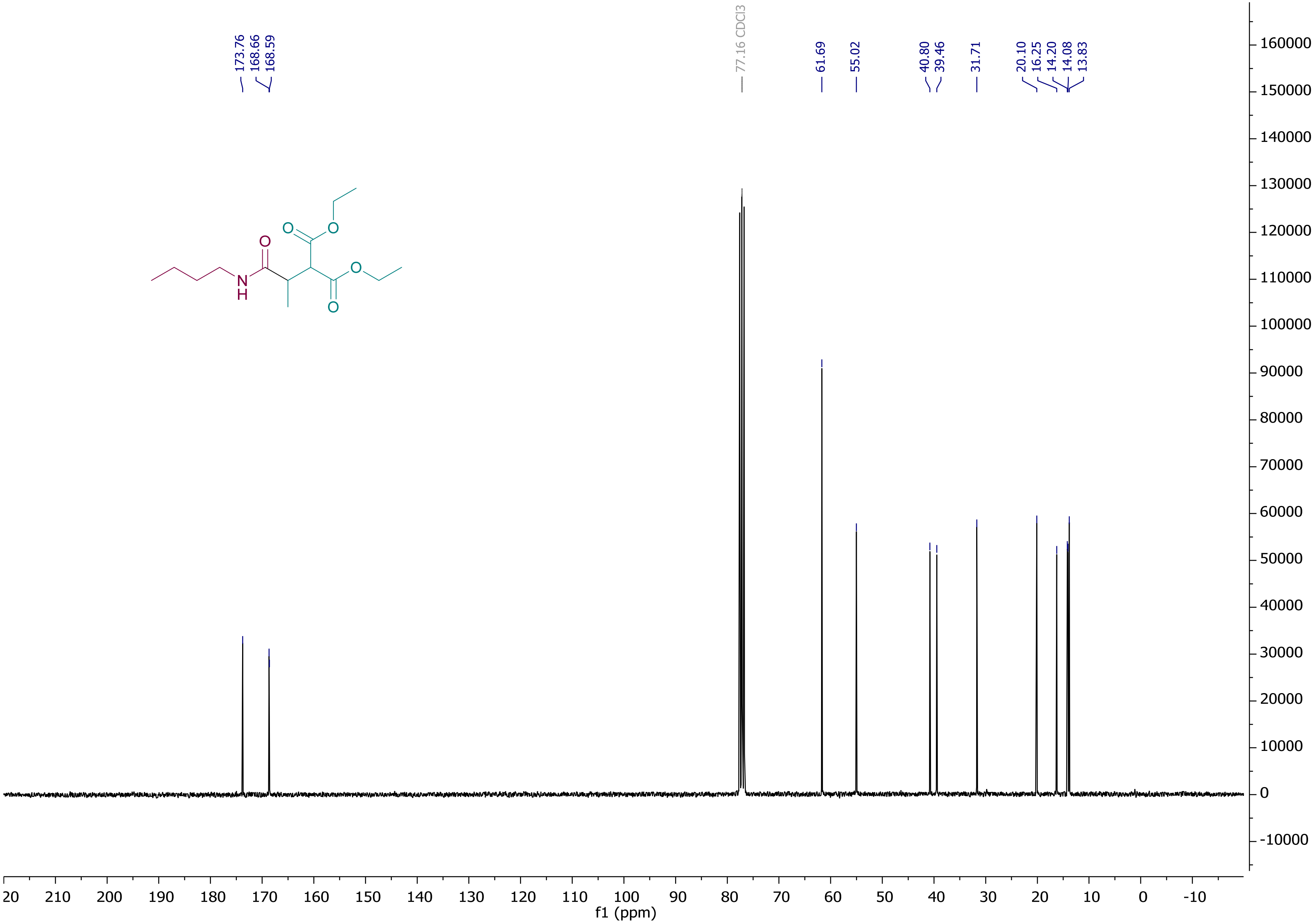

Diethyl 2-(1-oxo-1-((2,2,2-trifluoroethyl)amino)propan-2-yl)malonate (9c)

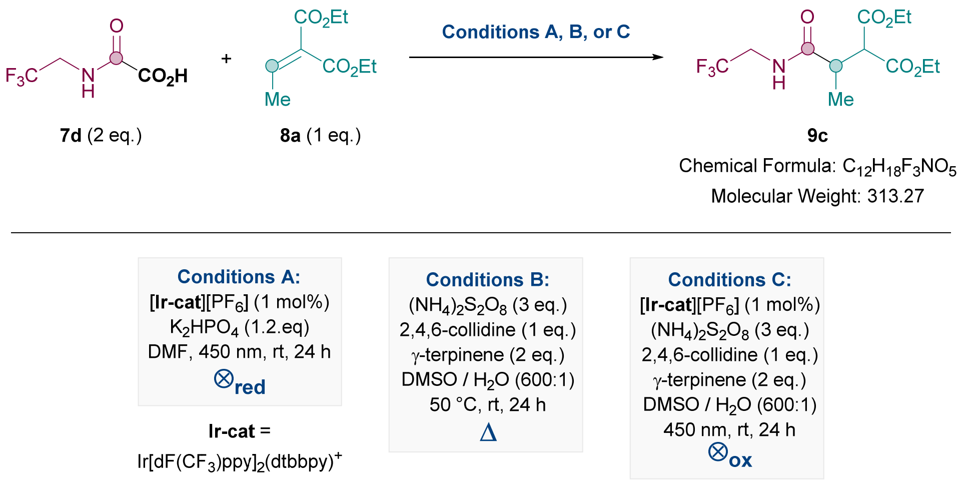

Using Conditions A: General procedure for Conditions A was followed. Diethyl 2-ethylidenemalonate **8a** (22.4 mg, 0.12 mmol, 1.0 eq.), 2-oxo-2-((2,2,2-trifluoroethyl)amino)acetic acid **7d** (41.3 mg, 0.24 mmol, 2.0 eq.), K_2HPO_4 (25.3 mg, 0.14 mmol, 1.2 eq.), Ir[dF(CF_3)ppy](dtbppy)PF_6 (1.3 mg, 1 μmol, 1 mol%) in dry DMF (0.3 mL) was irradiated with 450 nm for 24 h. The crude was then purified *via* column flash chromatography eluting with 65:35 hexane:EtOAc. The solvent was then removed *in vacuo* to afford diethyl 2-(1-oxo-1-((2,2,2-trifluoroethyl)amino)propan-2-yl)malonate **9c** as an off-white solid (25.2 mg, 0.080 mmol, 67%).

Using Conditions B: General procedure for Conditions B was followed. Diethyl 2-ethylidenemalonate **8a** (22.4 mg, 0.12 mmol, 1.0 eq.), 2-oxo-2-((2,2,2-trifluoroethyl)amino)acetic acid **7d** (41.2 mg, 0.24 mmol, 2.0 eq.), $(NH_4)_2S_2O_8$ (82.3 mg, 0.36 mmol, 3.0 eq.), γ-terpinene (38.5 μL, 0.24 mmol, 2.0 eq.), and 2,4,6-collidine (15.9 μL, 0.12 mmol, 1.0 eq.) in DMSO:H_2O (600:1) (0.4 mL) was heated at 50 °C for 24 h followed by work-up X. The crude was then purified *via* column flash chromatography eluting with 65:35 hexane:EtOAc. The solvent was then removed *in vacuo* to afford diethyl 2-(1-oxo-1-((2,2,2-trifluoroethyl)amino)propan-2-yl)malonate **9c** as an off-white solid (32.0 mg, 0.10 mmol, 85%).

Using Conditions C: General procedure for Conditions C was followed. Diethyl 2-ethylidenemalonate **8a** (22.9 mg, 0.12 mmol, 1.0 eq.), 2-oxo-2-((2,2,2-trifluoroethyl)amino)acetic acid **7d** (41.4 mg, 0.24 mmol, 2.0 eq.), Ir[dF(CF_3)ppy](dtbppy)PF_6

(1.6 mg, 1 μmol, 1 mol%), $(NH_4)_2S_2O_8$ (82.6 mg, 0.36 mmol, 3.0 eq.), γ-terpinene (38.5 μL, 0.24 mmol, 2.0 eq.), and 2,4,6-collidine (15.9 μL, 0.12 mmol, 1.0 eq.) in DMSO:H_2O (600:1) (0.4 mL) was irradiated with 450 nm for 24 h. The crude was then purified *via* column flash chromatography eluting with 65:35 hexane:EtOAc. The solvent was then removed *in vacuo* to afford diethyl 2-(1-oxo-1-((2,2,2-trifluoroethyl)amino)propan-2-yl)malonate **9c** as an off-white solid (27.5 mg, 0.085 mmol, 71%).

Characterisation:

^{1}H NMR (300 MHz, Chloroform-*d*): δ ppm 6.49 (br t, *J* = 6.4 Hz, 1H, NH), 4.21 (q, *J* = 7.1 Hz, 2H, OCH_2), 4.15 (qd, *J* = 7.1, 1.0 Hz, 2H, OCH_2), 4.08 – 3.89 (m, 1H, C<u>H</u>HCF$_3$), 3.89 – 3.67 (m, 1H, CH<u>H</u>CF$_3$), 3.74 (d, *J* = 10.0 Hz, 1H, C<u>H</u>(CO$_2$Et)$_2$), 3.04 (dq, *J* = 10.0, 7.1 Hz, 1H, MeC<u>H</u>), 1.33 – 1.17 (m, 9H, 3 x CH_3). **^{13}C NMR (75 MHz, Chloroform-*d*):** δ ppm 174.5 (C), 168.5 (C plus one overlapping C), 124.2 (q, $J_{C\text{-}F}$ = 278.4 Hz, C), 61.99 (CH_2), 61.97 (CH_2), 54.9 (CH), 40.77 (q, $J_{C\text{-}F}$ = 34.7 Hz, CH_2), 40.55 (CH) 16.1 (CH_3), 14.1 (CH_3), 14.0 (CH_3). **^{19}F NMR (282 MHz, Chloroform-*d*):** δ ppm -72.61 (t, *J* = 9.2 Hz). **IR:** ν_{max}/cm^{-1} 2958, 2929, 2873, 2859, 1724, 1600, 1580, 1541, 1462, 1380, 1270, 1120. **HRMS (ESI-TOF):** *m*/*z* [M + H]$^+$ calcd for $C_{12}H_{19}F_3NO_5$, 314.12098; found, 314.1214. **TLC:** R_f = 0.38 (60:40 hexane:EtOAc). **m.p.** 80-82 °C.

Compound 9c – ^{1}H NMR (300 MHz, Chloroform-*d*):

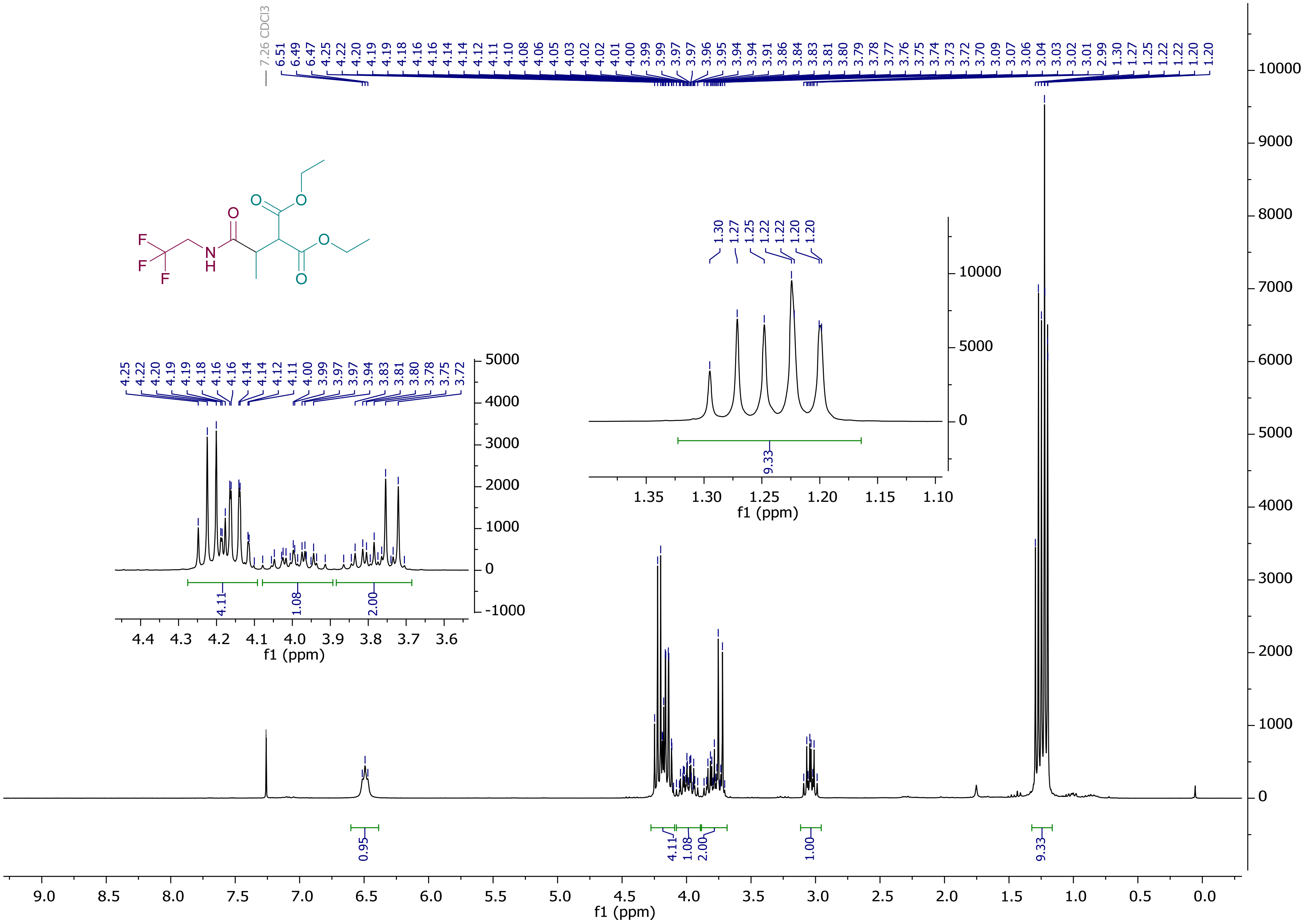

Compound 9c – ^{13}C NMR (75 MHz, Chloroform-*d*):

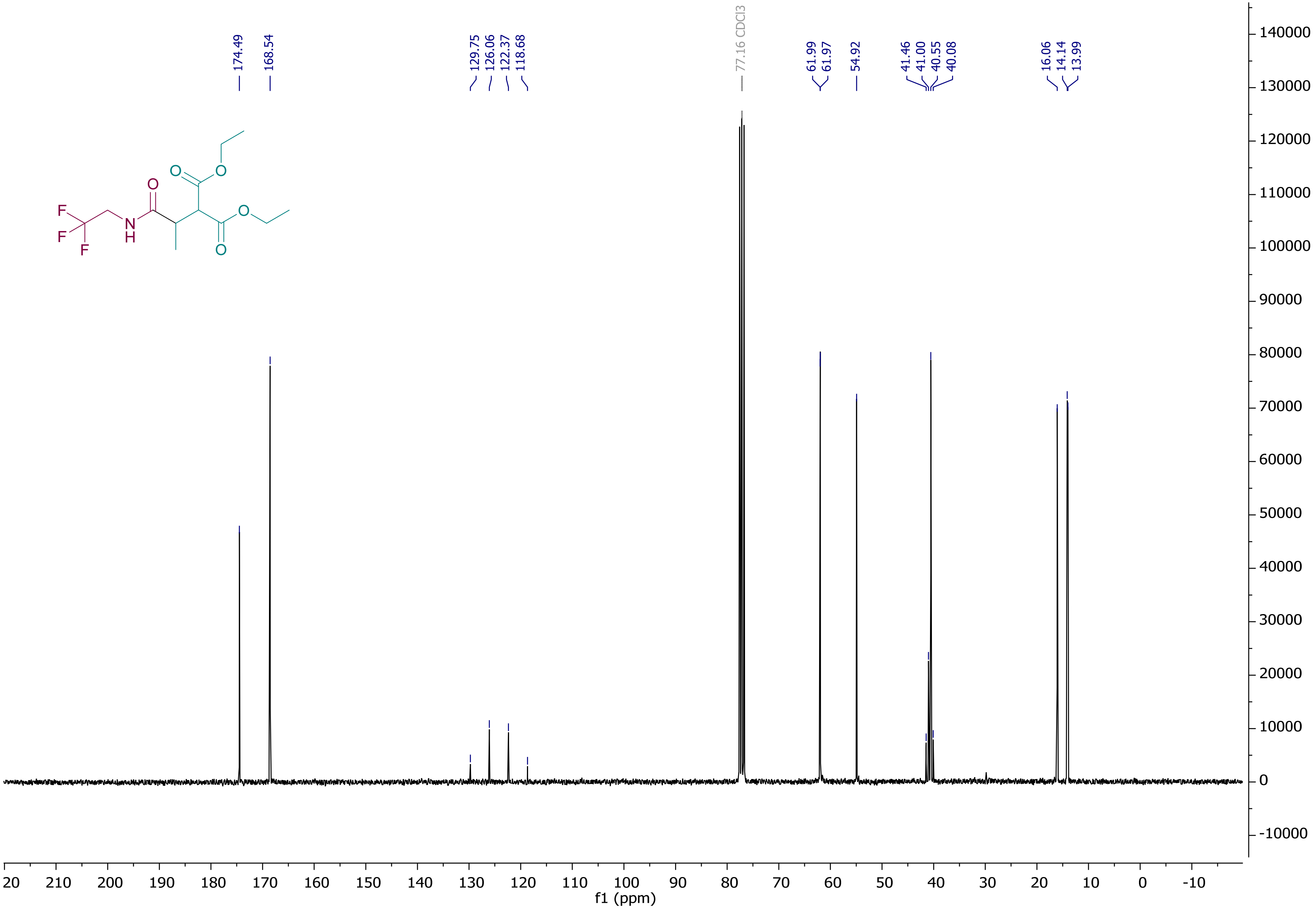

Compound 9c – ^{19}F NMR (282 MHz, Chloroform-*d*):

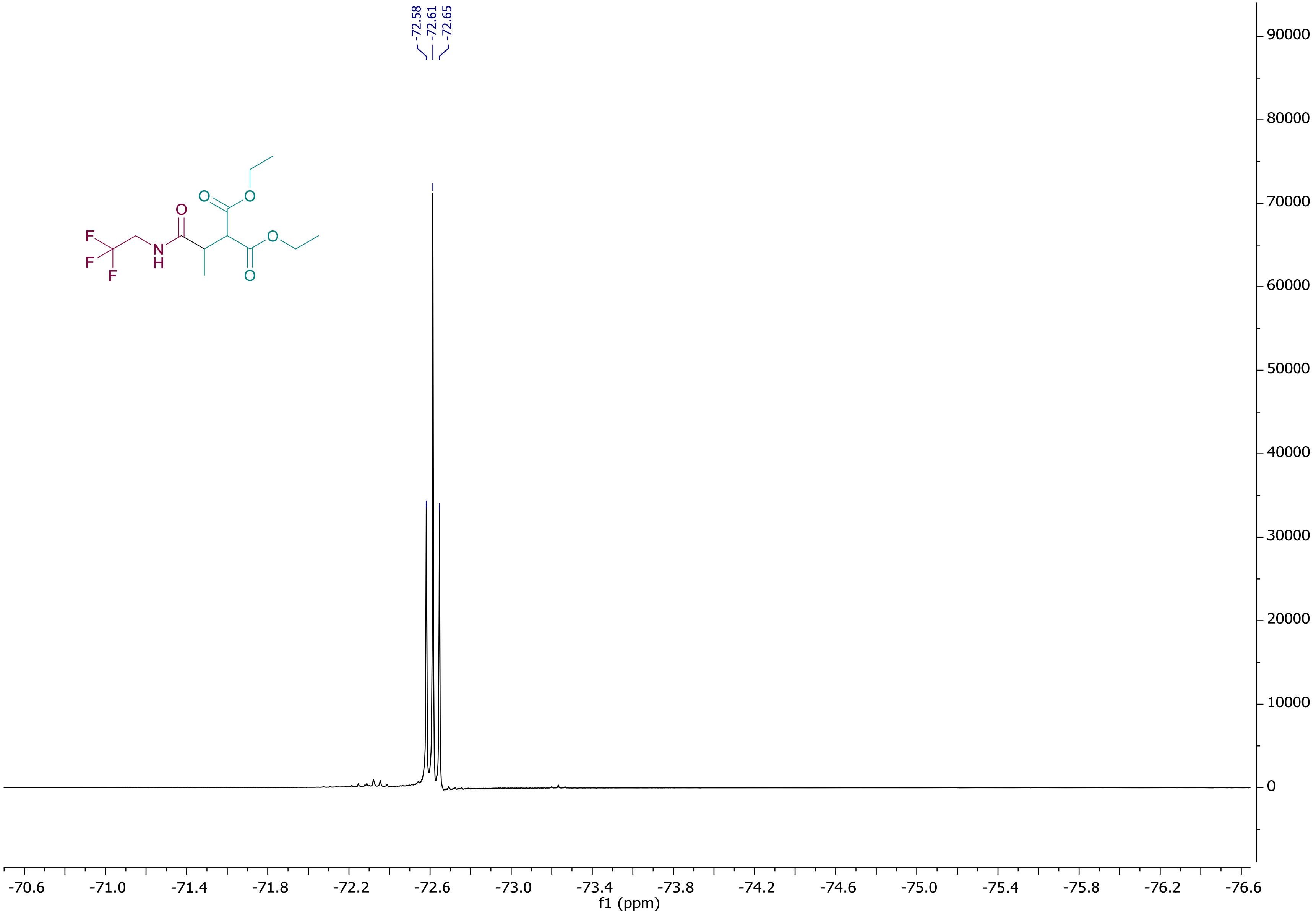

Diethyl 2-(1-(benzylamino)-1-oxopropan-2-yl)malonate) (9d)

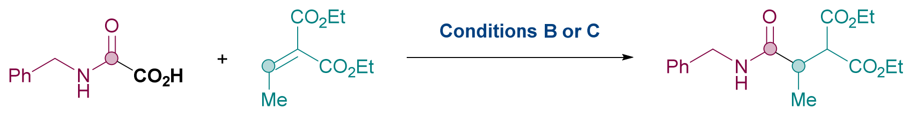

7e (2 eq.) **8a** (1 eq.) **9d**

Chemical Formula: $C_{17}H_{23}NO_5$
Molecular Weight: 321.37

Conditions B:
$(NH_4)_2S_2O_8$ (3 eq.)
2,4,6-collidine (1 eq.)
γ-terpinene (2 eq.)
DMSO / H_2O (600:1)
50 °C, rt, 24 h
Δ

Conditions C:
[Ir-cat][PF_6] (1 mol%)
$(NH_4)_2S_2O_8$ (3 eq.)
2,4,6-collidine (1 eq.)
γ-terpinene (2 eq.)
DMSO / H_2O (600:1)
450 nm, rt, 24 h
⊗**ox**

Ir-cat =
Ir[dF(CF_3)ppy]$_2$(dtbbpy)$^+$

Using Conditions B: General procedure for Conditions B was followed. Diethyl 2-ethylidenemalonate **8a** (22.3 mg, 0.12 mmol, 1.0 eq.), *N*-benzyl-oxoacetic acid **7e** (43.3 mg, 0.24 mmol, 2.0 eq.), $(NH_4)_2S_2O_8$ (82.6 mg, 0.36 mmol, 3.0 eq.), γ-terpinene (38.5 µL, 0.24 mmol, 2.0 eq.), and 2,4,6-collidine (15.9 µL, 0.12 mmol, 1.0 eq.) in DMSO:H_2O (600:1) (0.4 mL) was heated at 50 °C for 24 h followed by work-up X. The crude was then purified *via* column flash chromatography eluting with 65:35 hexane:EtOAc. The solvent was then removed *in vacuo* to afford diethyl 2-(1-(benzylamino)-1-oxopropan-2-yl)malonate **9d** as an off-white solid (31.1 mg, 0.097 mmol, 81%).

Using Conditions C: General procedure for Conditions C was followed. Diethyl 2-ethylidenemalonate **8a** (22.4 mg, 0.12 mmol, 1.0 eq.), *N*-benzyl-oxoacetic acid **7e** (43.4 mg, 0.24 mmol, 2.0 eq.), $(NH_4)_2S_2O_8$ (82.1 mg, 0.36 mmol, 3.0 eq.), Ir[dF(CF_3)ppy](dtbppy)PF_6 (1.3 mg, 1 µmol, 1 mol%), γ-terpinene (38.5 µL, 0.24 mmol, 2.0 eq.), and 2,4,6-collidine (15.9 µL, 0.12 mmol, 1.0 eq.) in DMSO:H_2O (600:1) (0.4 mL) was irradiated with 450 nm for 24 h. The crude was then purified *via* column flash chromatography eluting with 65:35 hexane:EtOAc. The solvent was then removed *in vacuo* to afford diethyl 2-(1-amino-1-oxopropan-2-yl)malonate **9d** as an off-white solid (31.3 mg, 0.097 mmol, 81%).

1 mmol scale: A pre-made, 20 mL, stock solution of DMSO:H_2O (600:1) was sparged with argon (2 balloons) for 1.5 h. To an oven-dried, 25 ml round-bottom flask equipped with a stirrer bar, which has previously been evacuated and re-filled with Ar three times, was added diethyl

2-ethylidenemalonate **8a** (0.18 mL, 1.00 mmol, 1.0 eq.), *N*-benzyl-oxoacetic acid **7e** (358.04 mg, 2.00 mmol, 2.0 eq.), $(NH_4)_2S_2O_8$ (684.7 mg, 3.00 mmol, 3.0 eq.), γ-terpinene (0.37 mL, 2.00 mmol, 2.0 eq.), and 2,4,6-collidine (0.13 mL, 1.00 mmol, 1.0 eq.). After sparging, an aliquot of the stock solution of DMSO:H_2O (600:1) (18 mL) was added to the flask, and the resulting solution was sparged with more argon for 30 min (2 balloons) with continuous stirring, before being heated to 50 °C for 24 h. The reaction was then diluted with CH_2Cl_2 (60 mL) and the resulting solution was washed with sat. aq. $NaHCO_3$ (80 mL) and the layers were separated. The aq. layer was extracted with more CH_2Cl_2 (3 x 30 mL), and organic layers were combined and washed with 1 M HCl (60 mL) and the layers were separated. The acid aq. layer was extracted with more CH_2Cl_2 (30 mL) and the organic layers were combined, dried over $MgSO_4$, and concentrated *in vacuo* to give the crude product. The crude was then purified *via* column flash chromatography eluting with 65:35 hexane:EtOAc. The solvent was then removed *in vacuo* to afford diethyl 2-(1-amino-1-oxopropan-2-yl)malonate **9d** as an off-white solid (229.5 mg, 0.72 mmol, 72%).

5.4 mmol scale: A pre-made, 20 mL, stock solution of DMSO:H_2O (600:1) was sparged with argon (2 balloons) for 2 h. To an oven-dried, 150 ml two-necked flask equipped with a stirrer bar, which has previously been evacuated and re-filled with Ar three times, was added diethyl 2-ethylidenemalonate **8a** (0.99 mL, 5.4 mmol, 1.0 eq.), *N*-benzyl-oxoacetic acid **7e** (1.94 g, 10.8 mmol, 2.0 eq.), $(NH_4)_2S_2O_8$ (3.70 g, 16.2 mmol, 3.0 eq.), γ-terpinene (1.75 mL, 10.8 mmol, 2.0 eq.), and 2,4,6-collidine (0.72 mL, 5.4 mmol, 1.0 eq.). After sparging, an aliquot of the stock solution of DMSO:H_2O (600:1) (18 mL) was added to the flask, and the resulting solution was sparged with more argon for 1 h (2 balloons) with continuous stirring, before being heated to 50 °C for 24 h. The reaction was then diluted with CH_2Cl_2 (70 mL) and the resulting solution was washed with sat. aq. $NaHCO_3$ (100 mL) and the layers were separated. The aq. layer was extracted with more CH_2Cl_2 (3 x 30 mL), and organic layers were combined and washed with 1 M HCl (100 mL) and the layers were separated. The acid aq. layer was extracted with more CH_2Cl_2 (30 mL) and the organic layers were combined, dried over $MgSO_4$, and concentrated *in vacuo* to give the crude product. The crude was then purified *via* column flash chromatography eluting with 65:35 hexane:EtOAc. The solvent was then removed *in vacuo* to afford diethyl 2-(1-amino-1-oxopropan-2-yl)malonate **9d** as an off-white solid (960.3 mg, 2.97 mmol, 55%).

Characterisation:

^{1}H NMR (400 MHz, Chloroform-*d*): δ ppm 7.33 – 7.28 (m, 2H, ArH), 7.28 – 7.23 (m, 3H, ArH), 6.17 (br t, *J* = 5.2 Hz, 1H, NH), 4.42 (d, *J* = 5.7 Hz, 2H, CH_2), 4.21 – 4.06 (m, 4H, 2 x OCH_2), 3.78 (d, *J* = 10.1 Hz, 1H, C<u>H</u>(CO_2Et)$_2$), 2.97 (dq, *J* = 10.2, 7.0 Hz, 1H, MeCH), 1.26 (t, *J* = 7.1 Hz, 3H, $OCH_2C\underline{H}_3$), 1.21 (d, *J* = 7.1 Hz, 3H, C<u>H</u>CH_3), 1.21 (t, *J* = 7.1 Hz, 3H, $OCH_2C\underline{H}_3$). **^{13}C NMR (101 MHz, Chloroform-*d*):** δ ppm 173.8 (C), 168.6 (C), 168.5 (C), 138.4 (C), 128.7 (CH), 127.8 (CH), 127.5 (CH), 61.74 (CH_2), 61.72 (CH_2), 55.0 (CH), 43.7 (CH_2), 40.7 (CH), 16.2 (CH_3), 14.2 (CH_3), 14.1 (CH_3). **IR:** ν_{max}/cm^{-1} 3323, 3257, 3088, 2987, 2937, 1726, 1640, 1554, 1455, 1367, 1243. **HRMS (ESI-TOF):** *m/z* [M + H]$^+$ calcd for $C_{17}H_{24}NO_5$, ; found. **TLC:** R_f = 0.28 (65:35 hexane:EtOAc). **M.p.** 45-48 °C.

Compound 9d – ^{1}H NMR (400 MHz, Chloroform-*d*):

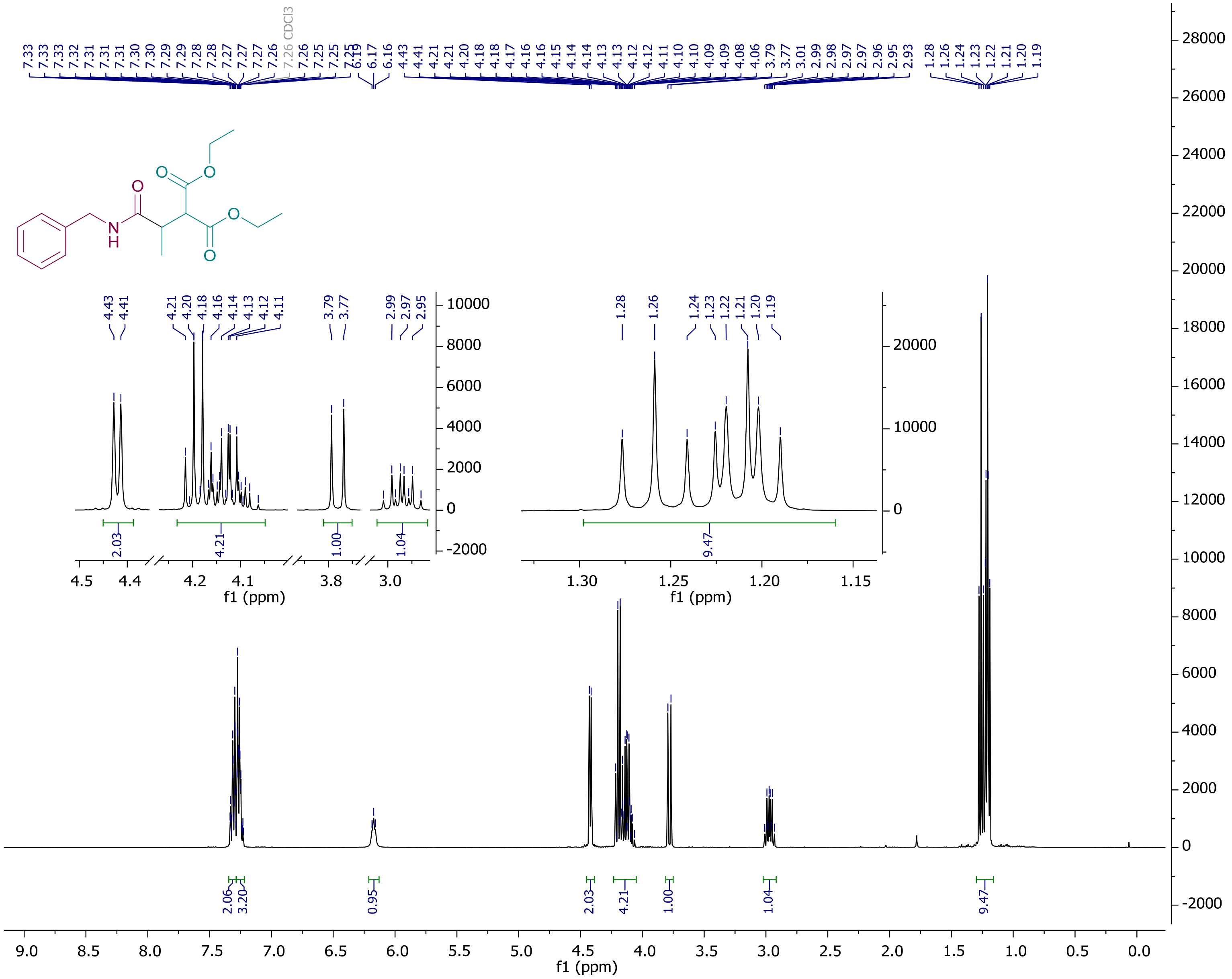

Compound 9d – ^{13}C NMR (101 MHz, Chloroform-*d*):

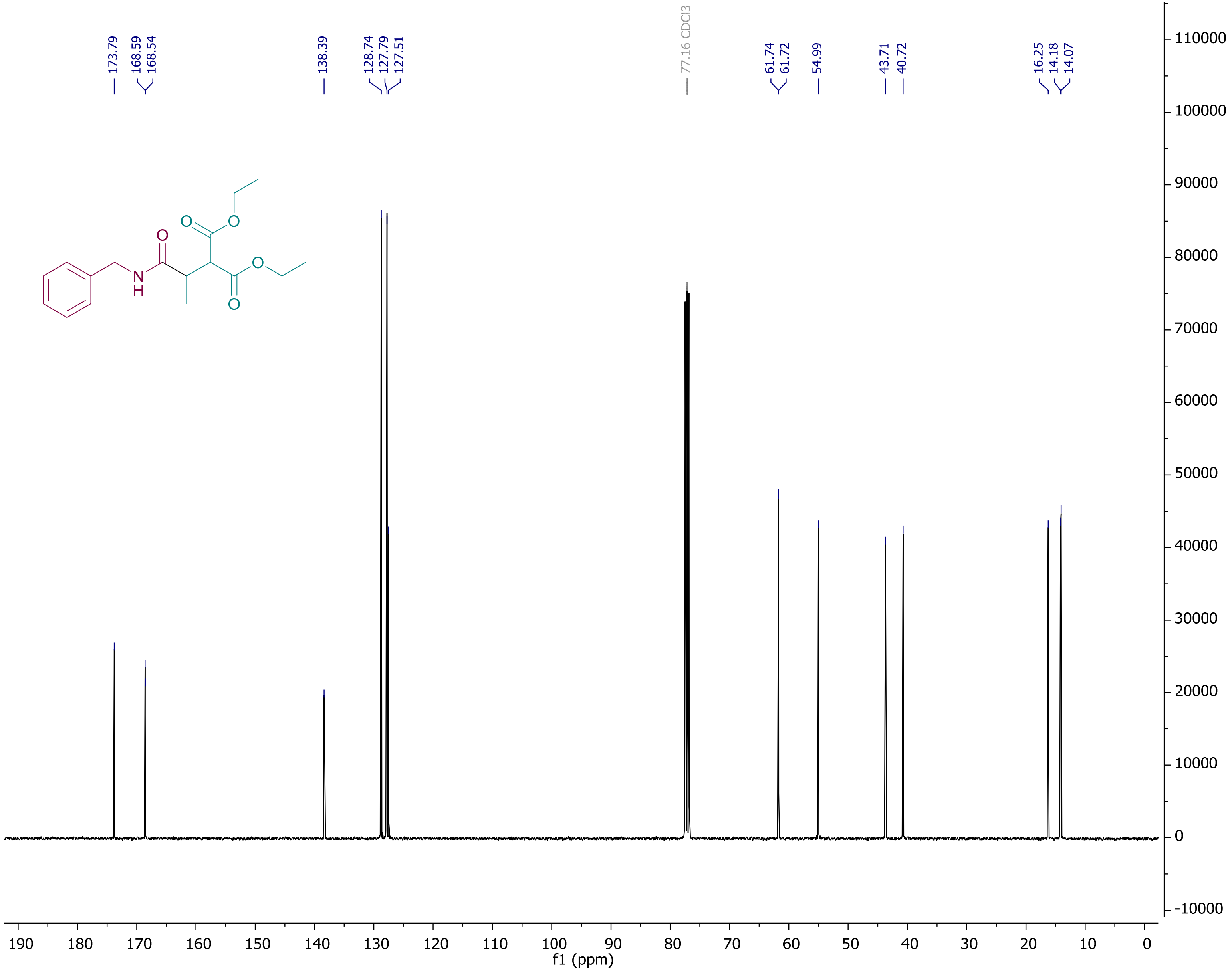

Diethyl 2-(1-oxo-1-(((*S*)-1-phenylethyl)amino)propan-2-yl)malonate (9e)

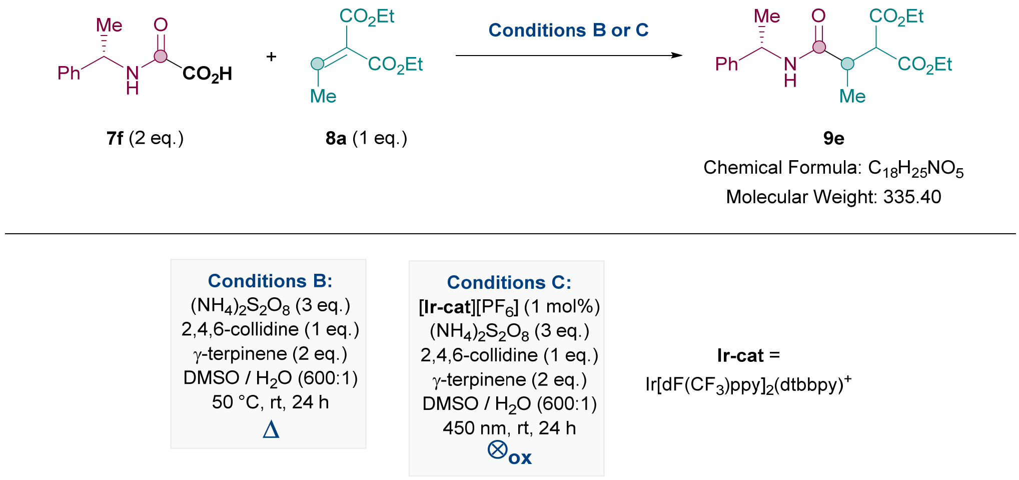

Using Conditions B: General procedure for Conditions B was followed. Diethyl 2-ethylidenemalonate **8a** (22.4 mg, 0.12 mmol, 1.0 eq.), (*S*)-2-oxo-2-((1-phenylethyl)amino)acetic acid **7f** (46.4 mg, 0.24 mmol, 2.0 eq.), $(NH_4)_2S_2O_8$ (82.3 mg, 0.36 mmol, 3.0 eq.), γ-terpinene (38.5 µL, 0.24 mmol, 2.0 eq.), and 2,4,6-collidine (15.9 µL, 0.12 mmol, 1.0 eq.) in DMSO:H_2O (600:1) (0.4 mL) was heated at 50 °C for 24 h followed by work-up X. The crude was then purified *via* column flash chromatography eluting with 65:35 hexane:EtOAc. The solvent was then removed *in vacuo* to afford a 50:50 diastereomeric mixture of diethyl 2-(1-oxo-1-(((*S*)-1-phenylethyl)amino)propan-2-yl)malonate **9e** as an off-white solid (29.0 mg, 0.086 mmol, 72%).

Using Conditions C: General procedure for Conditions C was followed. Diethyl 2-ethylidenemalonate **8a** (22.5 mg, 0.12 mmol, 1.0 eq.), (*S*)-2-oxo-2-((1-phenylethyl)amino)acetic acid **7f** (46.2 mg, 0.24 mmol, 2.0 eq.), $(NH_4)_2S_2O_8$ (82.0 mg, 0.36 mmol, 3.0 eq.), Ir[dF(CF_3)ppy](dtbppy)PF_6 (1.3 mg, 1 µmol, 1 mol%), γ-terpinene (38.5 µL, 0.24 mmol, 2.0 eq.), and 2,4,6-collidine (15.9 µL, 0.12 mmol, 1.0 eq.) in DMSO:H_2O (600:1) (0.4 mL) was irradiated with 450 nm for 24 h. The crude was then purified *via* column flash chromatography eluting with 65:35 hexane:EtOAc. The solvent was then removed *in vacuo* to afford a 50:50 diastereomeric mixture of diethyl 2-(1-oxo-1-(((*S*)-1-phenylethyl)amino)propan-2-yl)malonate **9e** as an off-white solid (30.4 mg, 0.091 mmol, 76%).

Characterisation:

^{1}H NMR (300 MHz, Chloroform-*d*): (50:50 mixture of diastereomers) δ ppm 7.41 – 7.16 (m, 5H, ArH), 6.11 (br d, *J* = 8.0 Hz, 1H, NH), 5.15 – 4.99 (m, 1H, C<u>H</u>Ph), 4.26 – 4.10 (m, 3H, 3 x OCH<u>H</u>), 4.03 (m, 1H, OC<u>H</u>H), 3.73 (apparent t, *J* = 10.1 Hz, 1H, C<u>H</u>(($CO_2Et)_2$)), 3.03 – 2.86 (m, 1H, C<u>H</u>Me), 1.47 (d, *J* = 6.9 Hz, 3H, CH_3), 1.29 – 1.19 (m, 6H, 2 x CH_3), 1.19 – 1.08 (m, 3H, CH_3). **^{13}C NMR (75 MHz, Chloroform-*d*):** (50:50 mixture of diastereomers) δ ppm 172.9 (C), 172.8 (C), 168.64 (C), 168.58 (C), 168.56 (C), 168.4 (C), 143.3 (C), 143.2 (C) 128.7 (CH), 128.6 (CH), 127.4 (CH), 127.3 (CH), 126.3 (CH), 126.2 (CH), 61.72 (CH_2), 61.70 (CH_2), 61.67 (CH_2 plus one overlapping CH_2), 55.0 (CH), 56.0 (CH), 48.9 (2 x CH overlapping), 40.75 (CH), 40.72 (CH), 21.9 (CH_3), 21.7 (CH_3), 16.2 (CH_3), 16.1 (CH_3), 14.2 (CH_3), 14.1(CH_3), 14.0 (CH_3) plus one overlapping CH_3. **IR:** ν_{max}/cm^{-1} 3381, 3302, 3063, 3030, 2978, 2936, 2878, 1748, 1729, 1642, 1538, 1449, 1368, 1299. **HRMS (ESI-TOF):** *m*/*z* $[M + H]^+$ calcd for $C_{18}H_{26}NO_5$, 336.18055; found, 336.1806. **TLC:** R_f = 0.31 (60:40 hexane:EtOAc).

Compound 9e – ^{1}H NMR (300 MHz, Chloroform-d):

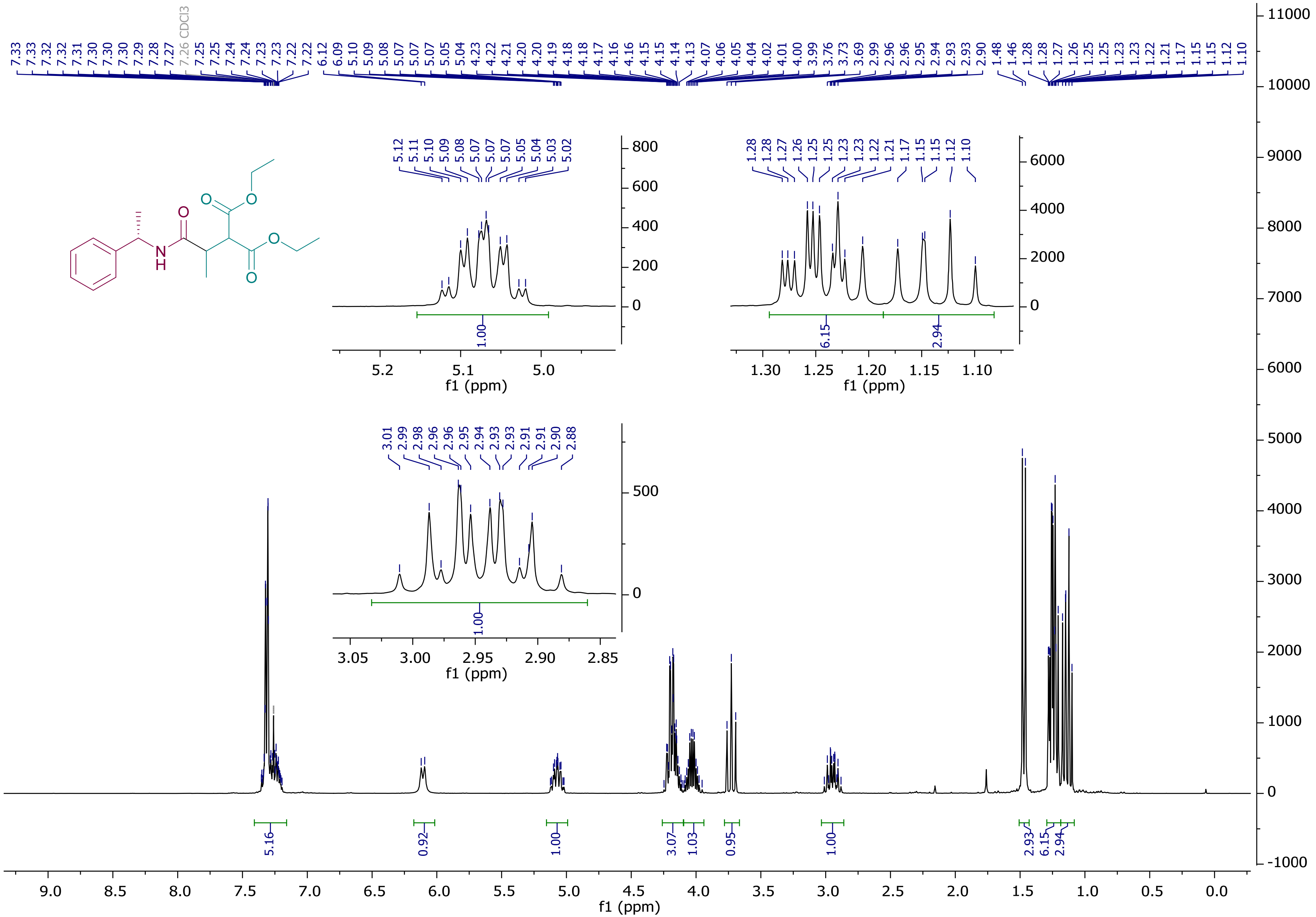

Compound 9e – ^{13}C NMR (75 MHz, Chloroform-d):

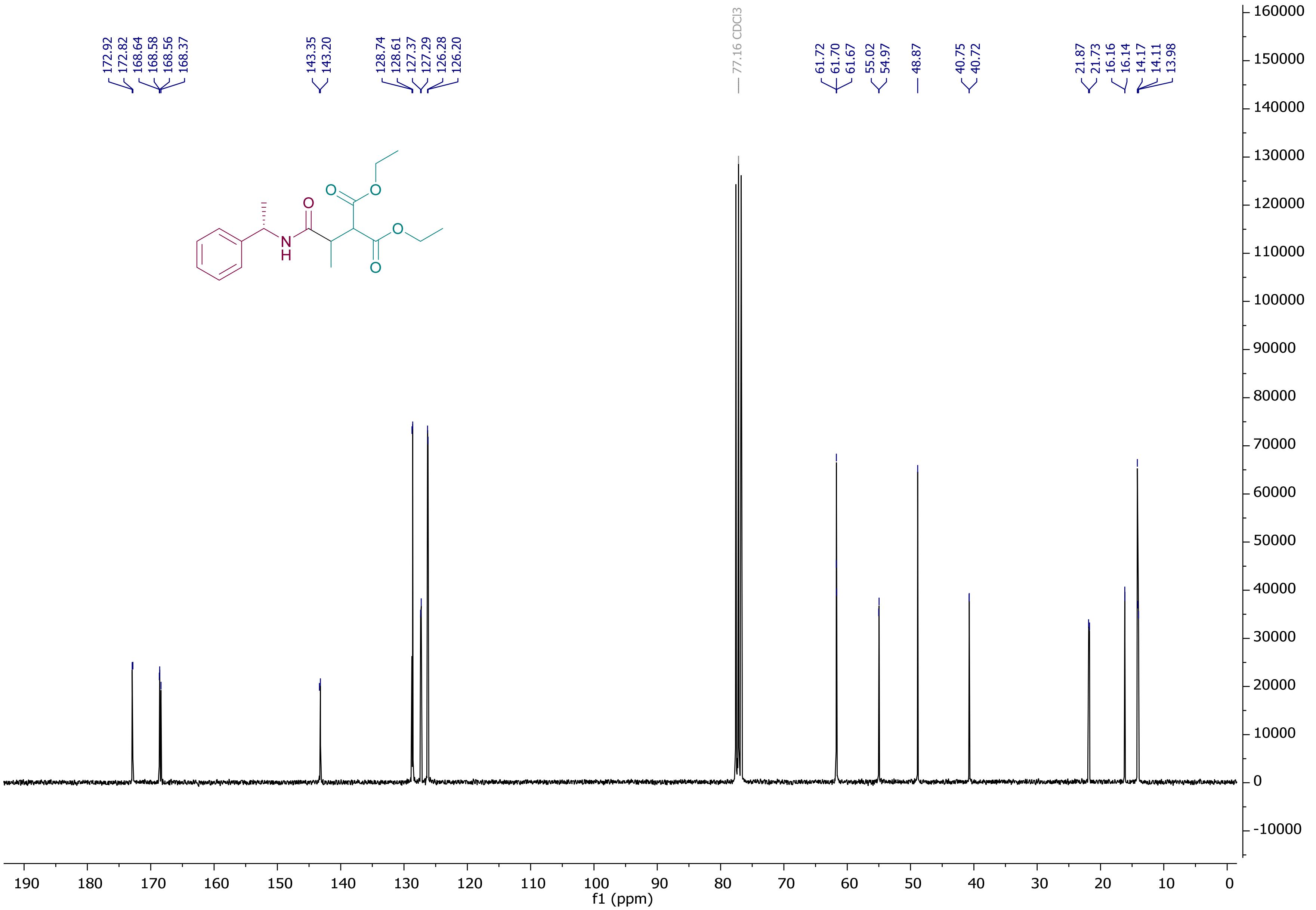

Diethyl 2-(1-(cyclopentylamino)-1-oxopropan-2-yl)malonate (9f)

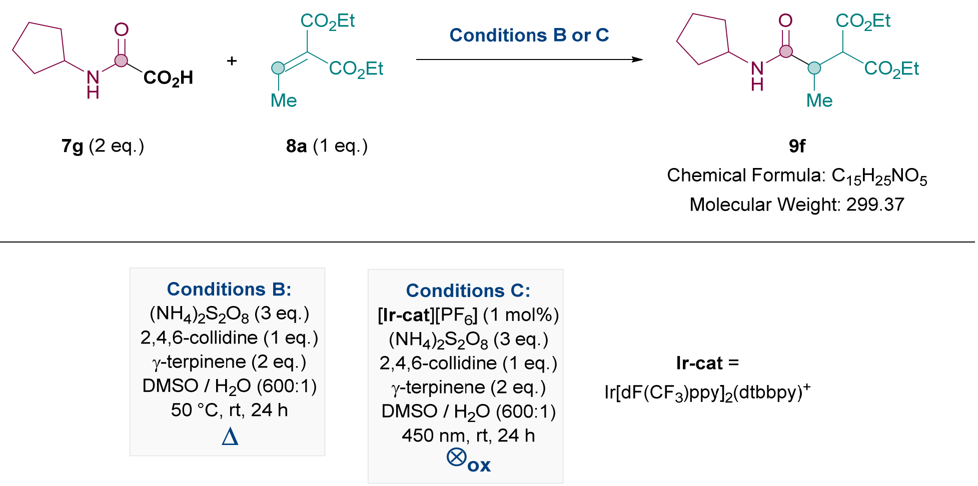

Using Conditions B: General procedure for Conditions B was followed. Diethyl 2-ethylidenemalonate **8a** (22.4 mg, 0.12 mmol, 1.0 eq.), *N*-(cyclopentyl)-oxoacetic acid **7g** (38.0 mg, 0.24 mmol, 2.0 eq.), $(NH_4)_2S_2O_8$ (82.4 mg, 0.36 mmol, 3.0 eq.), γ-terpinene (38.5 µL, 0.24 mmol, 2.0 eq.), and 2,4,6-collidine (15.9 µL, 0.12 mmol, 1.0 eq.) in DMSO:H_2O (600:1) (0.4 mL) was heated at 50 °C for 24 h followed by work-up X. The crude was then purified *via* column flash chromatography eluting with 65:35 hexane:EtOAc. The solvent was then removed *in vacuo* to afford diethyl 2-(1-(cyclopentylamino)-1-oxopropan-2-yl)malonate **9f** as an off-white solid (26.0 mg, 0.086 mmol, 72%).

Using Conditions C: General procedure for Conditions C was followed. Diethyl 2-ethylidenemalonate **8a** (22.6 mg, 0.12 mmol, 1.0 eq.), *N*-(cyclopentyl)-oxoacetic acid **7g** (37.6 mg, 0.24 mmol, 2.0 eq.), Ir[dF(CF_3)ppy](dtbppy)PF_6 (1.5 mg, 1 µmol, 1 mol%), $(NH_4)_2S_2O_8$ (82.2 mg, 0.36 mmol, 3.0 eq.), γ-terpinene (38.5 µL, 0.24 mmol, 2.0 eq.), and 2,4,6-collidine (15.9 µL, 0.12 mmol, 1.0 eq.) in DMSO:H_2O (600:1) (0.4 mL) was irradiated with 450 nm for 24 h. The crude was then purified *via* column flash chromatography eluting with 65:35 hexane:EtOAc. The solvent was then removed *in vacuo* to afford diethyl 2-(1-(cyclopentylamino)-1-oxopropan-2-yl)malonate **9f** as an off-white solid (26.0 mg, 0.086 mmol, 72%).

Characterisation:

^{1}H NMR (300 MHz, Chloroform-*d*): δ ppm 5.76 (br d, *J* = 7.0 Hz, 1H, NH), 4.25 – 4.03 (m, 5H, 2 x OCH_2, C<u>H</u>NH), 3.71 (d, *J* = 10.1 Hz, 1H, C<u>H</u>(CO_2Et)$_2$), 2.86 (dq, *J* = 10.0, 7.0 Hz, 1H, MeC<u>H</u>), 2.02 – 1.85 (m, 4H, 2 x CH_2), 1.74 – 1.48 (m, 2H, CH_2), 1.44 – 1.30 (m, 2H, CH_2), 1.26 (t, *J* = 7.1 Hz, 3H, $OCH_2C\underline{H}_3$), 1.22 (t, *J* = 7.1 Hz, 3H, $OCH_2C\underline{H}_3$), 1.16 (d, *J* = 7.0 Hz, 3H, $CHC\underline{H}_3$). **^{13}C NMR (75 MHz, Chloroform-*d*):** δ ppm 173.3 (C), 168.7 (C), 168.6 (C), 61.7 (2 x CH_2), 55.1 (CH), 51.3 (CH), 40.8 (CH), 33.08 (CH_2), 33.05 (CH_2), 23.9 (2 x CH_2), 16.2 (CH_3), 14.2 (CH_3), 14.1 (CH_3). **IR:** ν_{max}/cm^{-1} 3283, 2970, 2943, 2870, 1729, 1637, 1554, 1464, 1389, 1306. **HRMS (ESI-TOF):** *m*/*z* [M + H]$^+$ calcd for $C_{15}H_{26}NO_5$, 300.18055; found, 300.1819. **TLC:** R_f = 0.28 (65:35 hexane:EtOAc). **M.p.** 55-58 °C.

Compound 9f – ^{1}H NMR (300 MHz, Chloroform-*d*):

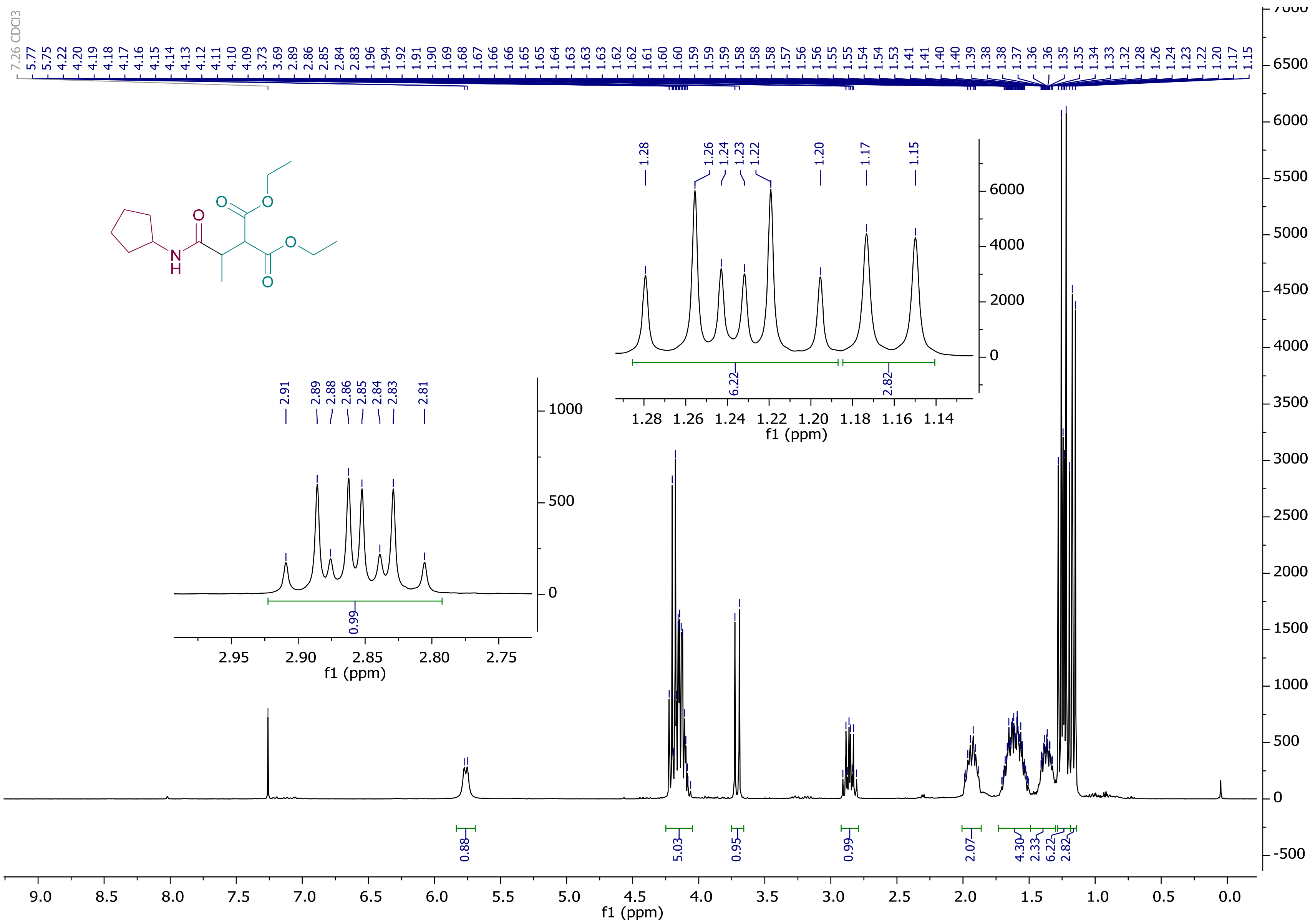

Compound 9f – ^{13}C NMR (75 MHz, Chloroform-*d*):

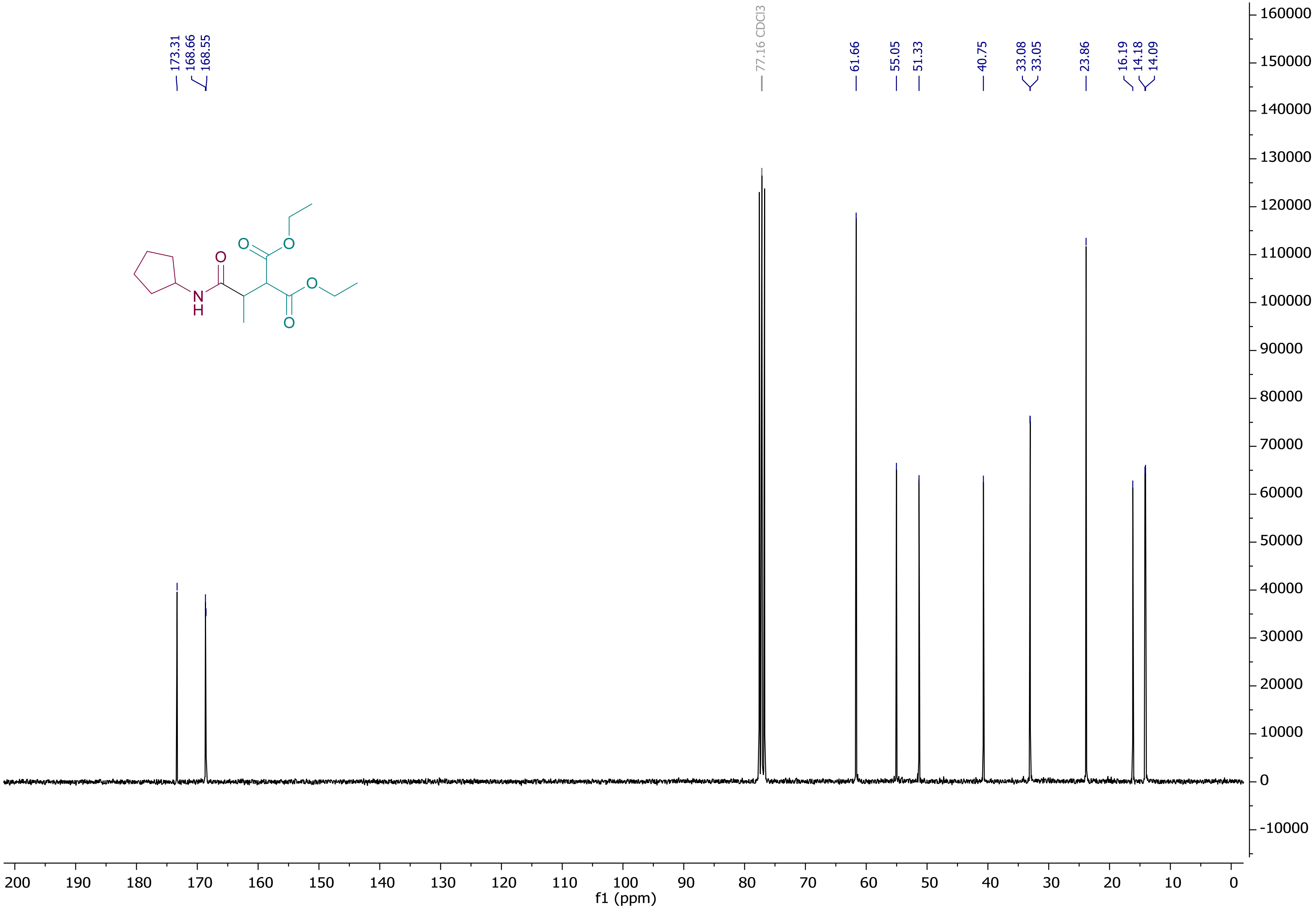

Diethyl 2-(1-(cyclohexylamino)-1-oxopropan-2-yl)malonate (9g)

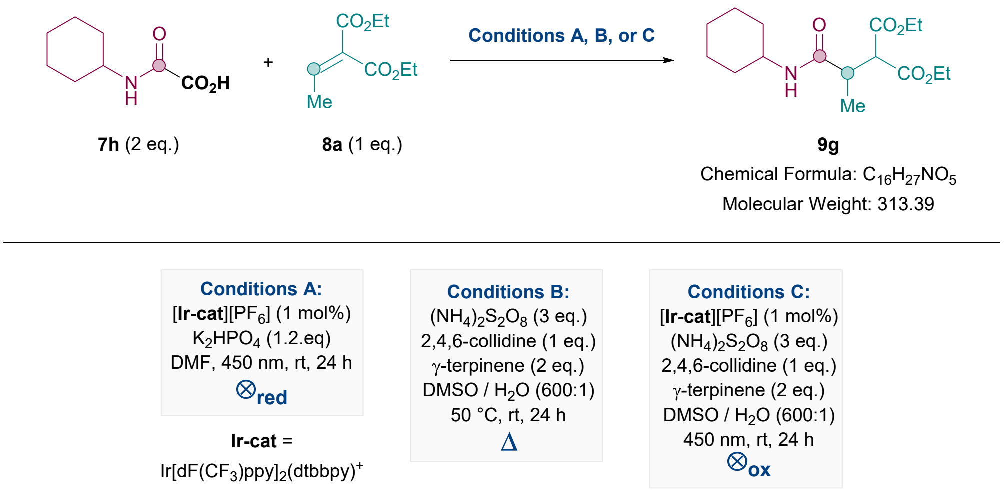

Using Conditions A: General procedure for Conditions A was followed. Diethyl 2-ethylidenemalonate **8a** (22.6 mg, 0.12 mmol, 1.0 eq.), 2 *N*-(cyclohexyl)-oxoacetic acid **7h** (41.6 mg, 0.24 mmol, 2.0 eq.), K_2HPO_4 (25.2 mg, 0.14 mmol, 1.2 eq.), Ir[dF(CF_3)ppy](dtbppy)PF_6 (1.4 mg, 1 µmol, 1 mol%) in dry DMF (0.3 mL) was irradiated with 450 nm for 24 h. The crude was then purified *via* column flash chromatography eluting with 75:25 hexane:EtOAc. The solvent was then removed *in vacuo* to afford diethyl diethyl 2-(1-(cyclohexylamino)-1-oxopropan-2-yl)malonate **9g** as an off-white solid (33.0 mg, 0.10 mmol, 87%).

Using Conditions B: General procedure for Conditions B was followed. Diethyl 2-ethylidenemalonate **8a** (22.4 mg, 0.12 mmol, 1.0 eq.), *N*-(cyclohexyl)-oxoacetic acid **7h** (41.1 mg, 0.24 mmol, 2.0 eq.), $(NH_4)_2S_2O_8$ (82.3 mg, 0.36 mmol, 3.0 eq.), γ-terpinene (38.5 µL, 0.24 mmol, 2.0 eq.), and 2,4,6-collidine (15.9 µL, 0.12 mmol, 1.0 eq.) in DMSO:H_2O (600:1) (0.4 mL) was heated at 50 ºC for 24 h followed by work-up X. The crude was then purified *via* column flash chromatography eluting with 60:40 hexane:EtOAc. The solvent was then removed *in vacuo* to afford diethyl 2-(1-(cyclohexylamino)-1-oxopropan-2-yl)malonate **9g** as an off-white solid (33.0 mg, 0.11 mmol, 88%).

Using Conditions C: General procedure for Conditions C was followed. Diethyl 2-ethylidenemalonate **8a** (22.3 mg, 0.12 mmol, 1.0 eq.), *N*-(cyclohexyl)-oxoacetic acid **7h** (41.1 mg, 0.24 mmol, 2.0 eq.), $(NH_4)_2S_2O_8$ (82.0 mg, 0.36 mmol, 3.0 eq.), Ir[dF(CF_3)ppy](dtbppy)PF_6 (1.5 mg, 1 µmol, 1 mol%), γ-terpinene (38.5 µL, 0.24 mmol, 2.0 eq.), and 2,4,6-collidine (15.9 µL, 0.12 mmol, 1.0 eq.) in DMSO:H_2O (600:1) (0.4 mL) was

irradiated with 450 nm for 24 h. The crude was then purified *via* column flash chromatography eluting with 65:35 hexane:EtOAc. The solvent was then removed *in vacuo* to afford diethyl 2-(1-(cyclohexylamino)-1-oxopropan-2-yl)malonate **9g** as an off-white solid (25.4 mg, 0.082 mmol, 68%).

Characterisation:

^{1}H NMR (400 MHz, Chloroform-*d*): δ ppm 5.65 (br d, *J* = 8.3 Hz, 1H, NH), 4.19 (q, *J* = 7.1 Hz, 2H, OCH_2), 4.18 – 4.09 (m, 2H, OCH_2), 3.76 – 3.65 (m, 1H, C<u>H</u>NH), 3.72 (d, *J* = 10.1 Hz, 1H, C<u>H</u>(CO_2Et)$_2$), 2.86 (dq, *J* = 10.0, 7.0 Hz, 1H, C<u>H</u>Me), 1.91 – 1.82 (m, 2H, CyH), 1.68 (dt, *J* = 12.1, 3.6 Hz, 2H, CyH), 1.58 (dt, *J* = 12.8, 3.7 Hz, 1H, CyH), 1.41-1.05 (m. 5H, CyH), 1.26 (t, *J* = 7.7 Hz, 3H, $OCH_2C\underline{H}_3$), 1.22 (t, *J* = 7.7 Hz, 3H, $OCH_2C\underline{H}_3$), 1.17 (d, *J* = 7.1 Hz, 3H, $CHC\underline{H}_3$). **^{13}C NMR (101 MHz, Chloroform-*d*):** δ ppm 172.9 (C), 168.7 (C), 168.5 (C), 61.7 (2 x CH_2), 55.0 (CH), 48.3 (CH), 40.9 (CH), 33.1 (CH_2), 33.0 (CH_2), 25.6 (CH_2), 24.92 (CH_2), 24.87 (CH_2), 16.3 (CH_3), 14.2 (CH_3), 14.1 (CH_3). **IR:** ν_{max}/cm^{-1} 3281, 2971, 2929, 2851, 1734, 1637, 1559, 1451, 1366, 1305. **HRMS (ESI-TOF):** *m*/*z* $[M + H]^+$ calcd for $C_{16}H_{28}NO_5$, 314.19620; found, 314.1962. **TLC:** R_f = 0.25 (70:30 hexane:EtOAc). **M.p.** 81-83 °C.

Compound 9g – ^{1}H NMR (400 MHz, Chloroform-*d*):

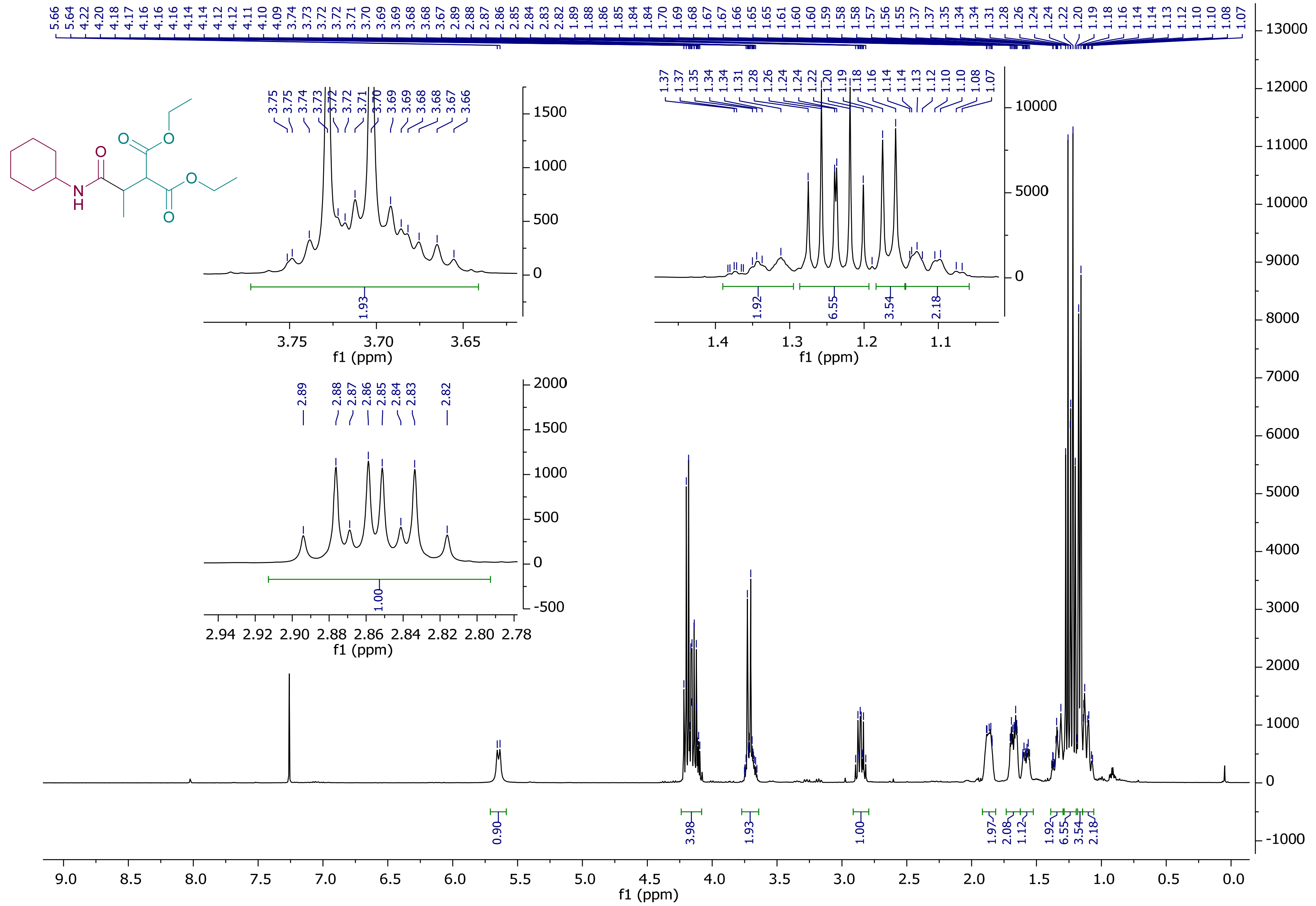

Compound 9g – ^{13}C NMR (101 MHz, Chloroform-*d*):

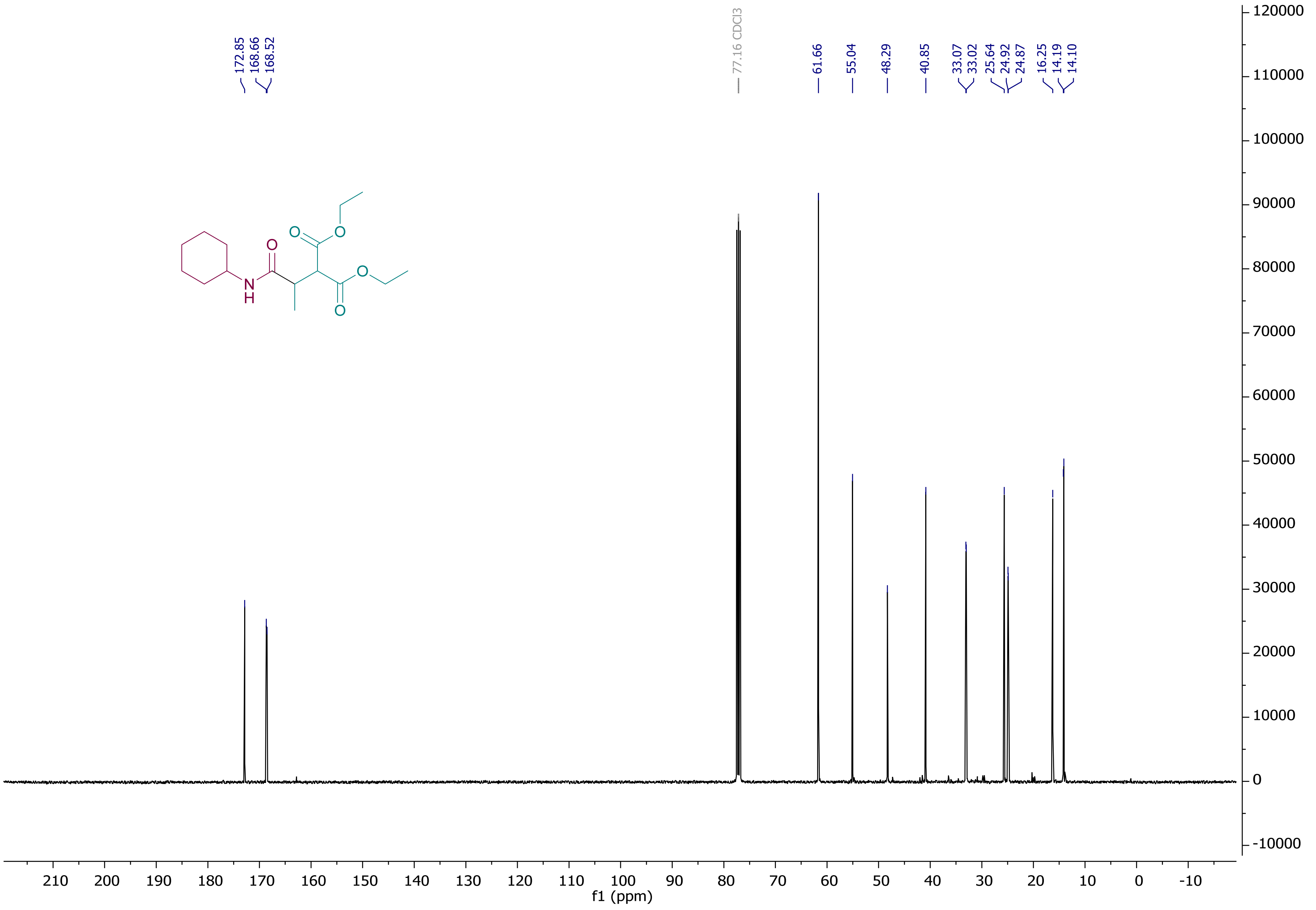

Diethyl 2-(1-(cycloheptylamino)-1-oxopropan-2-yl)malonate (9h)

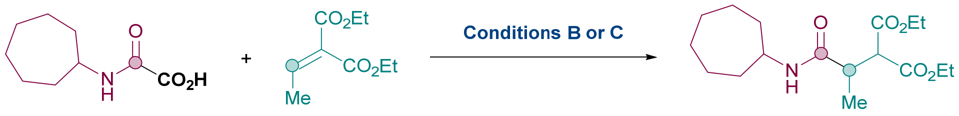

7i (2 eq.) **8a** (1 eq.) **9h**

Chemical Formula: $C_{17}H_{29}NO_5$

Molecular Weight: 327.42

Conditions B:
$(NH_4)_2S_2O_8$ (3 eq.)
2,4,6-collidine (1 eq.)
γ-terpinene (2 eq.)
DMSO / H_2O (600:1)
50 °C, rt, 24 h
Δ

Conditions C:
[Ir-cat][PF_6] (1 mol%)
$(NH_4)_2S_2O_8$ (3 eq.)
2,4,6-collidine (1 eq.)
γ-terpinene (2 eq.)
DMSO / H_2O (600:1)
450 nm, rt, 24 h
⊗**ox**

Ir-cat =
Ir[dF(CF_3)ppy]$_2$(dtbbpy)$^+$

Using Conditions B: General procedure for Conditions B was followed. Diethyl 2-ethylidenemalonate **8a** (22.5 mg, 0.12 mmol, 1.0 eq.), *N*-(cycloheptyl)-oxoacetic acid **7i** (44.6 mg, 0.24 mmol, 2.0 eq.), $(NH_4)_2S_2O_8$ (82.3 mg, 0.36 mmol, 3.0 eq.), γ-terpinene (38.5 μL, 0.24 mmol, 2.0 eq.), and 2,4,6-collidine (15.9 μL, 0.12 mmol, 1.0 eq.) in DMSO:H_2O (600:1) (0.4 mL) was heated at 50 ºC for 24 h followed by work-up X. The crude was then purified *via* column flash chromatography eluting with 70:30 hexane:EtOAc. The solvent was then removed *in vacuo* to afford diethyl 2-(1-(cycloheptylamino)-1-oxopropan-2-yl)malonate **9h** as a white solid (29.7 mg, 0.091 mmol, 76%).

Using Conditions C: General procedure for Conditions C was followed. Diethyl 2-ethylidenemalonate **8a** (22.1 mg, 0.12 mmol, 1.0 eq.), *N*-(cycloheptyl)-oxoacetic acid **7i** (44.6 mg, 0.24 mmol, 2.0 eq.), $(NH_4)_2S_2O_8$ (82.3 mg, 0.36 mmol, 3.0 eq.), Ir[dF(CF_3)ppy](dtbppy)PF_6 (1.3 mg, 1 μmol, 1 mol%), γ-terpinene (38.5 μL, 0.24 mmol, 2.0 eq.), and 2,4,6-collidine (15.9 μL, 0.12 mmol, 1.0 eq.) in DMSO:H_2O (600:1) (0.4 mL) was irradiated with 450 nm for 24 h. The crude was then purified *via* column flash chromatography eluting with 65:35 hexane:EtOAc. The solvent was then removed *in vacuo* to afford diethyl 2-(1-(cycloheptylamino)-1-oxopropan-2-yl)malonate **9h** as an off-white solid (26.6 mg, 0.081 mmol, 68%).

Characterisation:

^{1}H NMR (300 MHz, Chloroform-*d*): δ ppm 5.71 (br d, *J* = 8.2 Hz, 1H, NH), 4.19 (q, *J* = 6.7, 6.2 Hz, 2H, OCH_2), 4.22 – 4.05 (m, 2H, OCH_2), 3.98 – 3.80 (m, 1H, C<u>H</u>NH), 3.71 (d, *J* = 10.1 Hz, 1H, $CH(CO_2Et)_2$), 2.84 (dq, *J* = 10.1, 7.0 Hz, 1H, MeC<u>H</u>), 1.95 – 1.80 (m, 2H, alkyl CH_2), 1.63 – 1.31 (m, 10H, alkyl CH_2), 1.26 (t, *J* = 7.1 Hz, 3H, $OCH_2C\underline{H}_3$), 1.22 (t, *J* = 7.1 Hz, 3H, $OCH_2C\underline{H}_3$), 1.16 (d, *J* = 7.1 Hz, 3H, $CHC\underline{H}_3$). **^{13}C NMR (75 MHz, Chloroform-*d*):** δ ppm 172.5 (C), 168.7 (C), 168.5 (C), 61.7 (2 x CH_2), 55.0 (CH), 50.6 (CH), 40.8 (CH), 35.1 (CH_2), 35.0 (CH_2), 28.1 (2 x CH_2), 24.3 (CH_2), 24.2 (CH_2), 16.2 (CH_3), 14.2 (CH_3), 14.1 (CH_3). **IR:** ν_{max}/cm^{-1} 3289, 2970, 2935, 2869, 1733, 1683, 1549, 1464, 1365, 1287. **HRMS (ESI-TOF):** *m*/*z* $[M + H]^+$ calcd for $C_{17}H_{30}NO_5$, 328.21185; found, 328.2126. **TLC:** R_f = 0.30 (65:35 hexane:EtOAc). **M.p.** 105-108 °C.

Compound 9h – ^{1}H NMR (300 MHz, Chloroform-*d*):

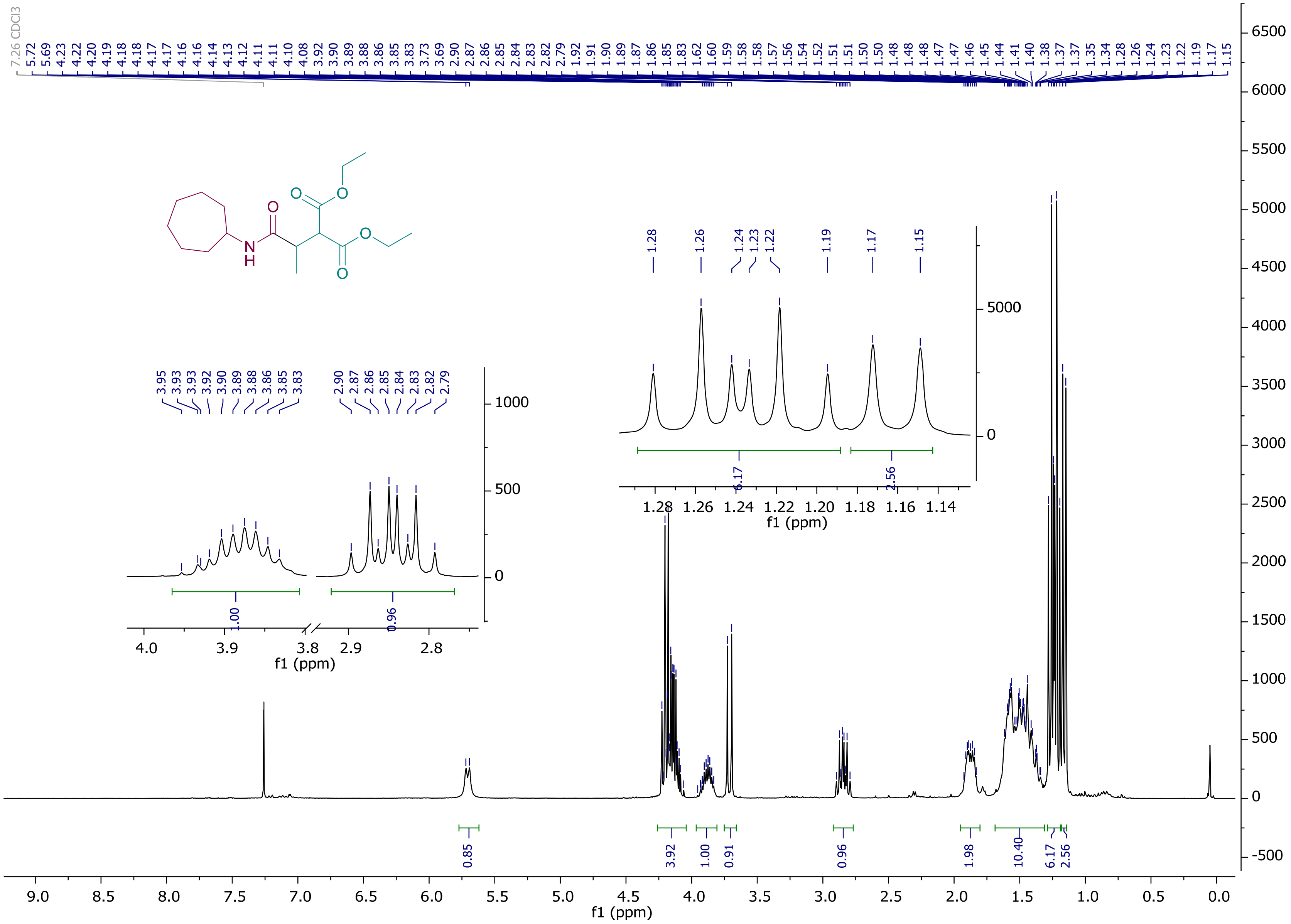

Compound 9h – ^{13}C NMR (75 MHz, Chloroform-*d*):

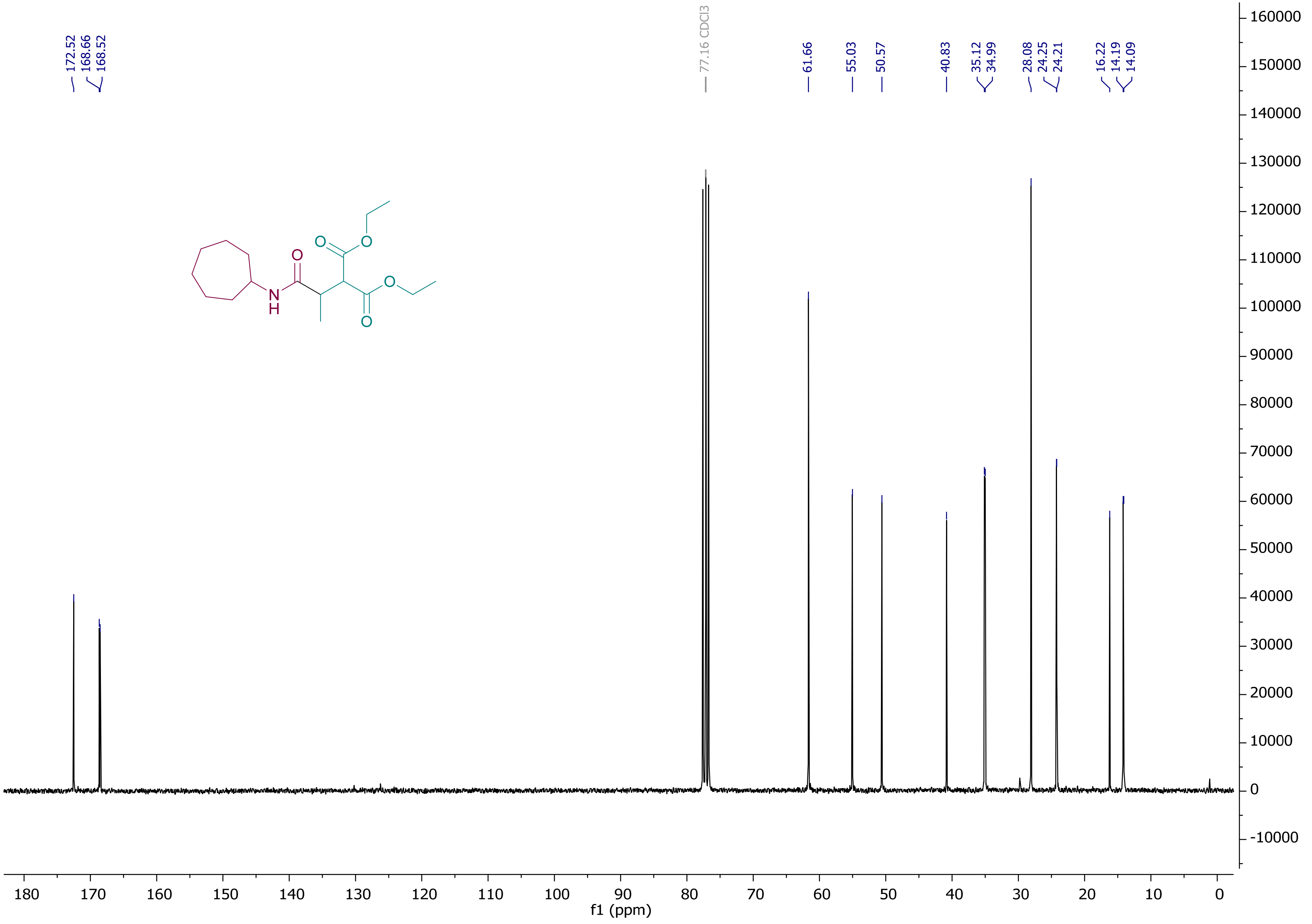

Diethyl 2-(1-(*t*-amino)-1-oxopropan-2-yl)malonate (9i)

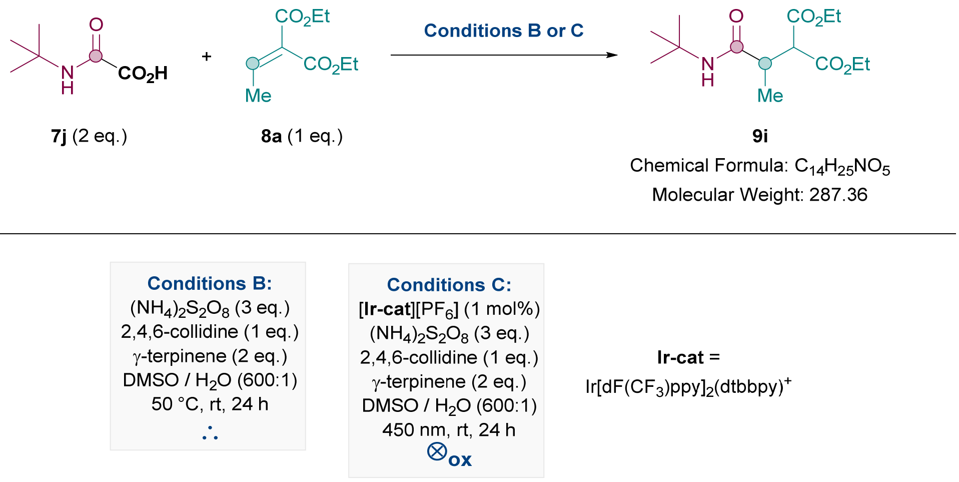

Using Conditions B: General procedure for Conditions B was followed. Diethyl 2-ethylidenemalonate **8a** (22.6 mg, 0.12 mmol, 1.0 eq.), *N*-(*t*-butyl)-oxoacetic acid **7j** (35.1 mg, 0.24 mmol, 2.0 eq.), $(NH_4)_2S_2O_8$ (82.3 mg, 0.36 mmol, 3.0 eq.), γ-terpinene (38.5 µL, 0.24 mmol, 2.0 eq.), and 2,4,6-collidine (15.9 µL, 0.12 mmol, 1.0 eq.) in DMSO:H_2O (600:1) (0.4 mL) was heated at 50 °C for 24 h followed by work-up X. The crude was then purified *via* column flash chromatography eluting with 95:5→70:30 hexane:EtOAc. The solvent was then removed *in vacuo* to afford diethyl 2-(1-(*t*-butylamino)-1-oxopropan-2-yl)malonate **9i** as an off-white solid (28.4 mg, 0.098 mmol, 82%).

Using Conditions C: General procedure for Conditions C was followed. Diethyl 2-ethylidenemalonate **8a** (22.4 mg, 0.12 mmol, 1.0 eq.), *N*-(*t*-butyl)-oxoacetic acid **7j** (35.1 mg, 0.24 mmol, 2.0 eq.), $(NH_4)_2S_2O_8$ (82.3 mg, 0.36 mmol, 3.0 eq.), Ir[dF(CF_3)ppy](dtbppy)PF_6 (1.4 mg, 1 µmol, 1 mol%), γ-terpinene (38.5 µL, 0.24 mmol, 2.0 eq.), and 2,4,6-collidine (15.9 µL, 0.12 mmol, 1.0 eq.) in DMSO:H_2O (600:1) (0.4 mL) was irradiated with 450 nm for 24 h. The crude was then purified *via* column flash chromatography eluting with 75:25 hexane:EtOAc. The solvent was then removed *in vacuo* to afford diethyl 2-(1-(*t*-butylamino)-1-oxopropan-2-yl)malonate **9i** as an off-white solid (28.6 mg, 0.10 mmol, 83%).

Characterisation:

^{1}H NMR (300 MHz, Chloroform-*d*): δ ppm 5.56 (br s, 1H, NH), 4.25 – 4.08 (m, 4H, 2 x OCH_2), 3.67 (d, *J* = 10.1 Hz, 1H, C<u>H</u>(CO_2Et)$_2$), 2.79 (dq, *J* = 10.1, 7.0 Hz, 1H, MeC<u>H</u>), 1.31

(s, 9H, 3 x CH_3), 1.26 (t, *J* = 7.1 Hz, 3H, $OCH_2C\underline{H}_3$), 1.23 (t, *J* = 7.1 Hz, 3H, $OCH_2C\underline{H}_3$), 1.15 (d, *J* = 7.0 Hz, 3H, $CHC\underline{H}_3$). **^{13}C NMR (101 MHz, Chloroform-*d*):** δ ppm 173.2 (C), 168.7 (C), 168.6 (C), 61.7 (CH_2 plus one overlapping CH_2), 55.1 (CH), 51.3 (C), 41.4 (CH), 28.7 (CH_3), 16.3 (CH_3), 14.2 (CH_3), 14.1 (CH_3). **IR:** ν_{max}/cm^{-1} 3308, 3080, 2978, 2909, 2852, 1739, 1724, 1644, 1551. **HRMS (ESI-TOF):** *m*/*z* $[M + H]^+$ calcd for $C_{14}H_{26}NO_5$, 288.18055; found, 288.1809. **TLC:** R_f = 0.24 (80:20 hexane:EtOAc). **M.p.** 60-64 °C.

Compound 9i – ¹H NMR (300 MHz, Chloroform-*d*):

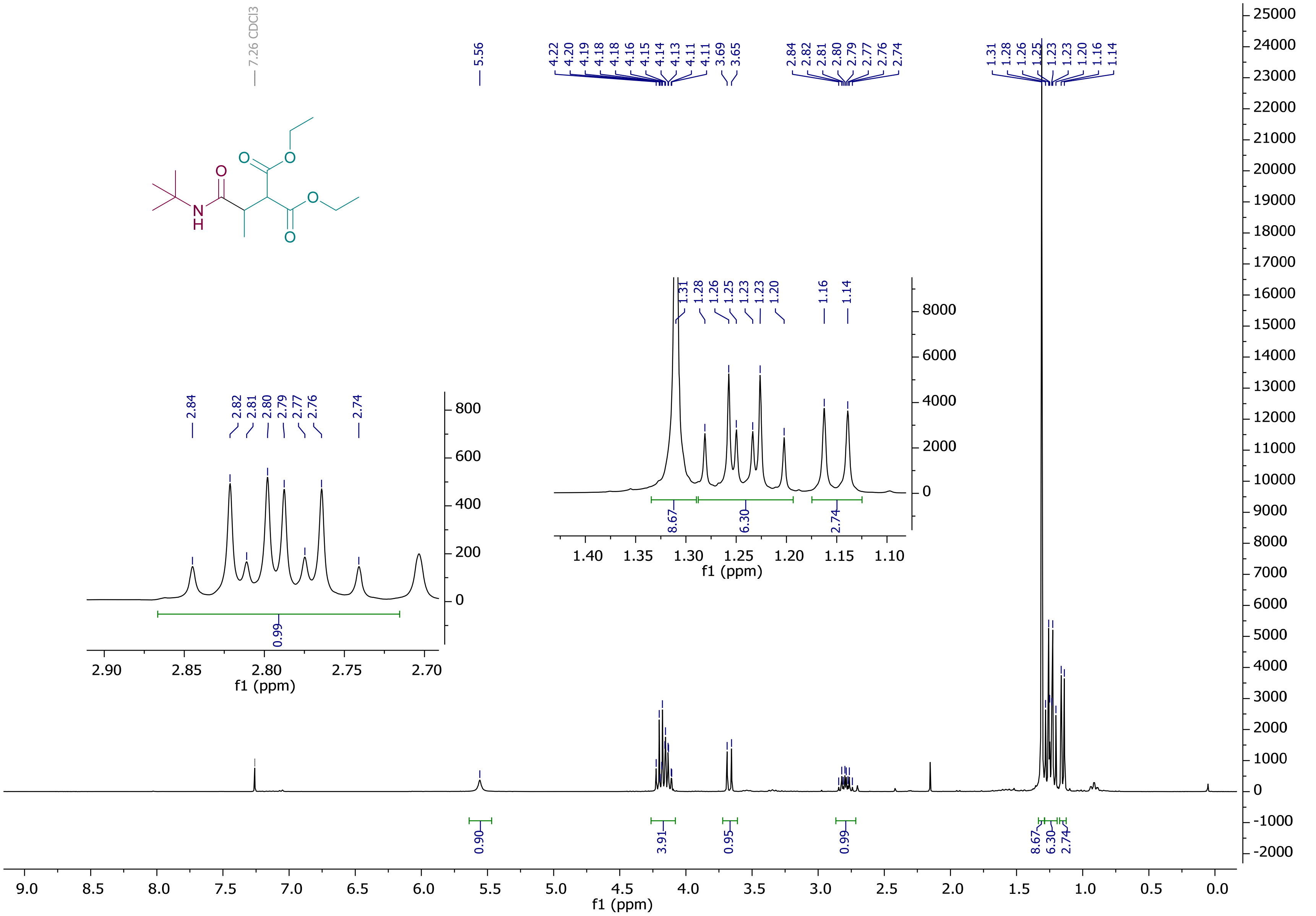

Compound 9i – ^{13}C NMR (101 MHz, Chloroform-*d*):

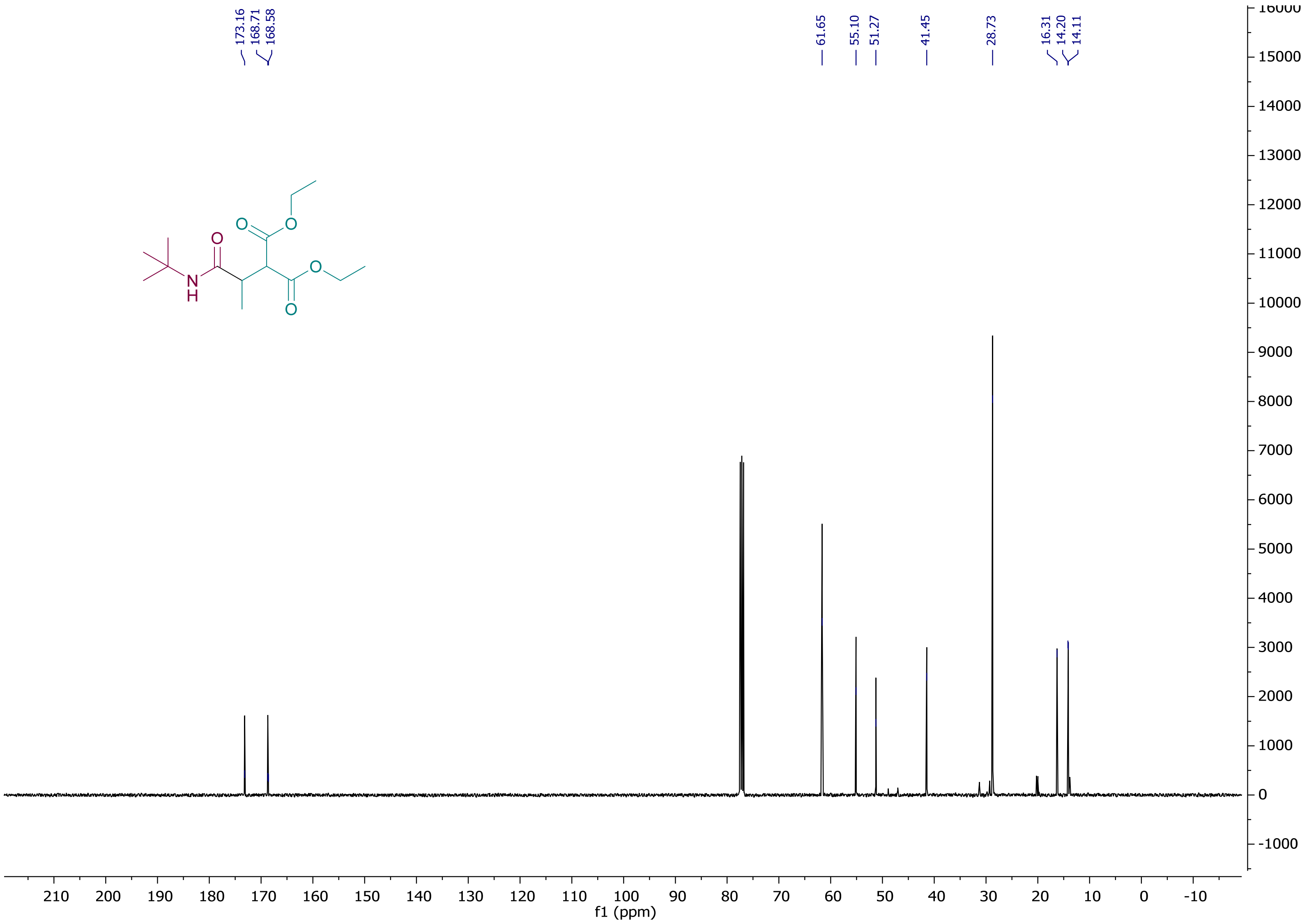

Diethyl 2-(1-((adamantan-1-yl)amino)-1-oxopropan-2-yl)malonate (9j)

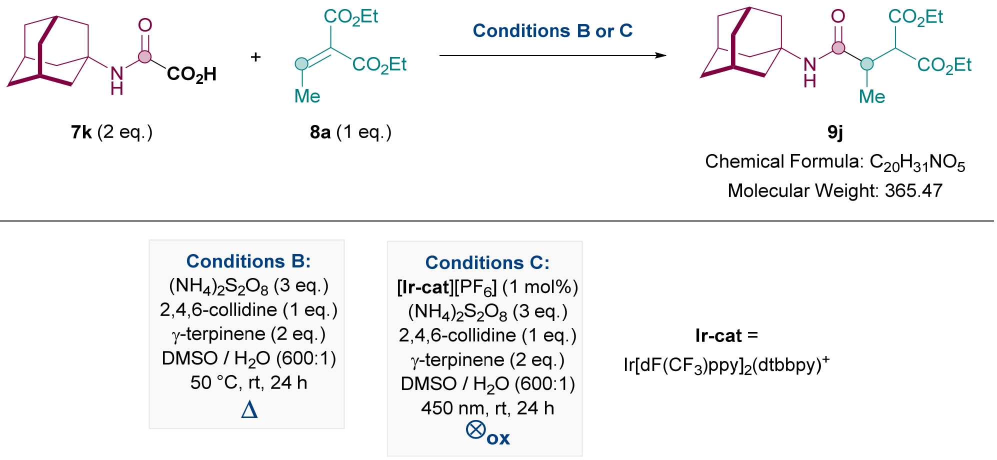

Using Conditions B: General procedure for Conditions B was followed. Diethyl 2-ethylidenemalonate **8a** (22.4 mg, 0.12 mmol, 1.0 eq.), *N*-(1-adamantyl)-oxoacetic acid **7k** (53.7 mg, 0.24 mmol, 2.0 eq.), $(NH_4)_2S_2O_8$ (82.2 mg, 0.36 mmol, 3.0 eq.), γ-terpinene (38.5 µL, 0.24 mmol, 2.0 eq.), and 2,4,6-collidine (15.9 µL, 0.12 mmol, 1.0 eq.) in DMSO:H_2O (600:1) (0.4 mL) was heated at 50 ºC for 24 h followed by work-up X. The crude was then purified *via* column flash chromatography eluting with 90:10→80:20 hexane:EtOAc. The solvent was then removed *in vacuo* to afford diethyl 2-(1-((adamantan-1-yl)amino)-1-oxopropan-2-yl)malonate **9j** as an off-white solid (30.1 mg, 0.083 mmol, 69%).

Using Conditions C: General procedure for Conditions C was followed. Diethyl 2-ethylidenemalonate **8a** (22.4 mg, 0.12 mmol, 1.0 eq.), *N*-(1-adamantyl)-oxoacetic acid **7k** (53.6 mg, 0.24 mmol, 2.0 eq.), $(NH_4)_2S_2O_8$ (82.3 mg, 0.36 mmol, 3.0 eq.), Ir[dF(CF_3)ppy](dtbppy)PF_6 (1.4 mg, 1 µmol, 1 mol%), γ-terpinene (38.5 µL, 0.24 mmol, 2.0 eq.), and 2,4,6-collidine (15.9 µL, 0.12 mmol, 1.0 eq.) in DMSO:H_2O (600:1) (0.4 mL) was irradiated with 450 nm for 24 h. The crude was then purified *via* column flash chromatography eluting with 75:25 hexane:EtOAc. The solvent was then removed *in vacuo* to afford 2-(1-((adamantan-1-yl)amino)-1-oxopropan-2-yl)malonate **9j** as an off-white solid (34.6 mg, 0.094 mmol, 79%).

Characterisation:

^{1}H NMR (400 MHz, Chloroform-*d*): δ 5.39 (br s, 1H, NH), 4.19 (q, *J* = 7.2 Hz, 2H, OCH_2), 4.23 – 4.07 (m, 2H, OCH_2), 3.66 (d, *J* = 10.2 Hz, 1H, C$\underline{H}$(CO_2Et)$_2$), 2.78 (dq, *J* = 10.2, 7.0 Hz, 1H, MeC$\underline{H}$), 2.07 – 1.98 (m, 3H, 3 x CH), 1.98 – 1.90 (m, 6H, 3 x CH_2), 1.65 (s, 6H, 3 x CH_2), 1.26 (t, *J* = 7.1 Hz, 3H, $OCH_2C\underline{H}_3$), 1.23 (t, *J* = 7.1 Hz, 3H, $OCH_2C\underline{H}_3$), 1.15 (d, *J* = 7.0 Hz, 3H, $CHC\underline{H}_3$). **^{13}C NMR (101 MHz, Chloroform-*d*):** 173.0 (C), 168.7 (C), 168.6 (C), 61.6 (CH_2 plus one overlapping CH_2), 55.1 (CH), 52.0 (C), 41.52 (CH_2), 41.50 (CH), 36.4 (CH_2), 29.5 (CH), 16.4 (CH_3), 14.2 (CH_3), 14.1 (CH_3). **IR:** ν_{max}/cm^{-1} 3307, 3078, 2976, 2909, 2852, 1739, 1723, 1643, 1551. **HRMS (ESI-TOF):** *m*/*z* [M + H]$^+$ calcd for $C_{20}H_{32}NO_5$, 366.22750; found, 366.2274. **TLC:** R_f = 0.26 (80:20 hexane:EtOAc). **M.p.** 104-108 °C.

Compound 9j – ^{1}H NMR (400 MHz, Chloroform-*d*):

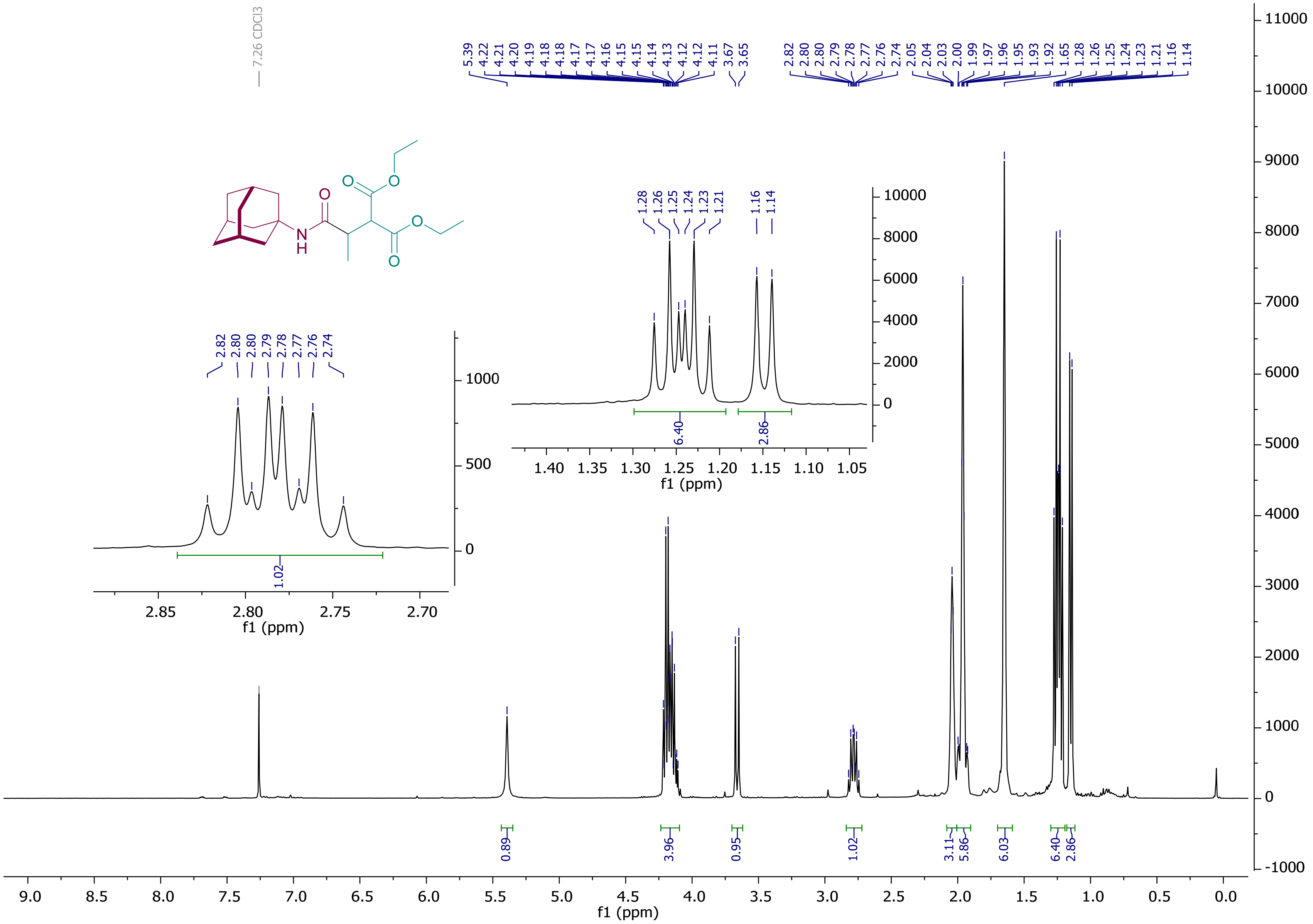

Compound 9j – ^{13}C NMR (101 MHz, Chloroform-*d*):

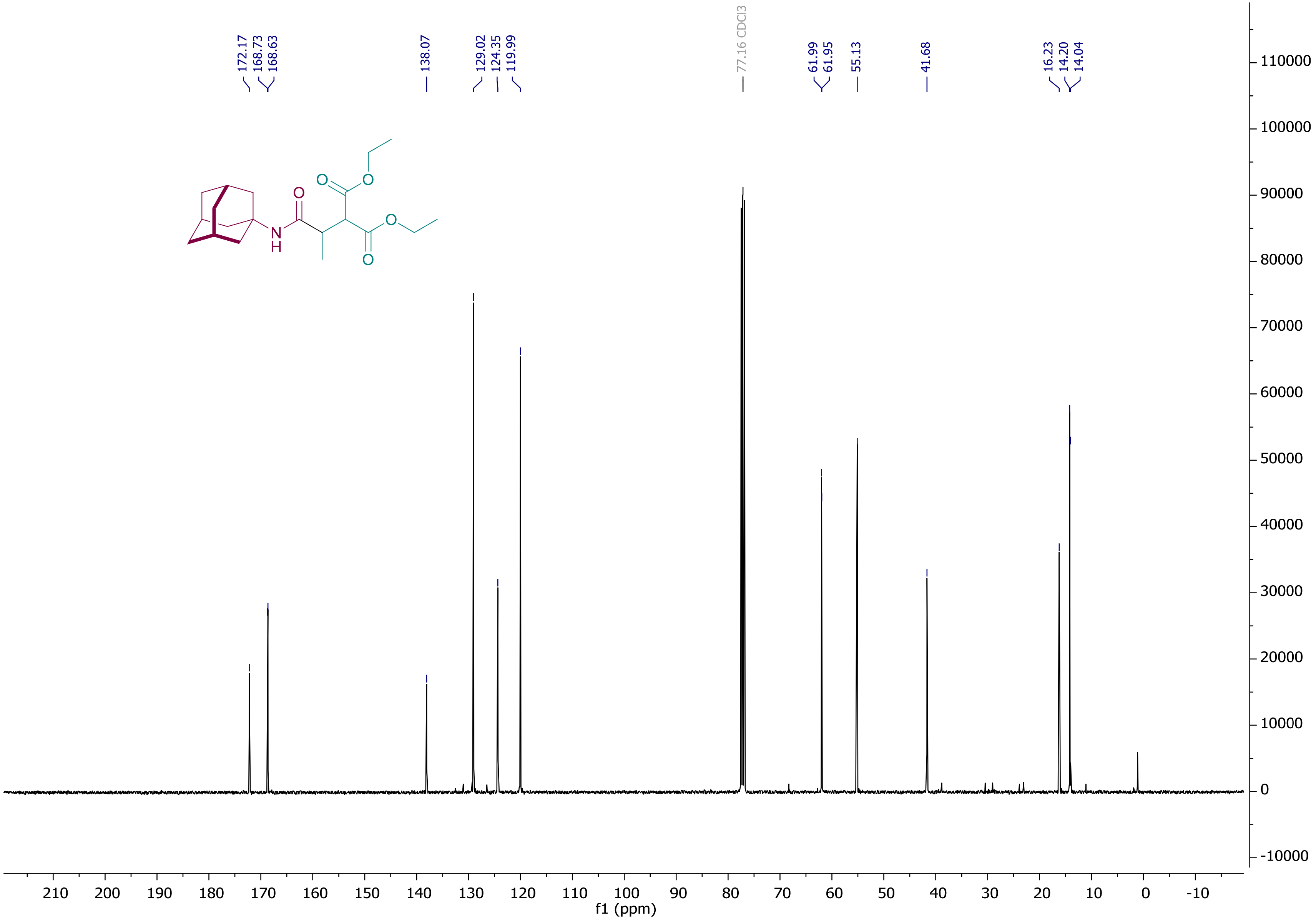

Diethyl 2-(1-oxo-1-(phenylamino)propan-2-yl)malonate (9k)

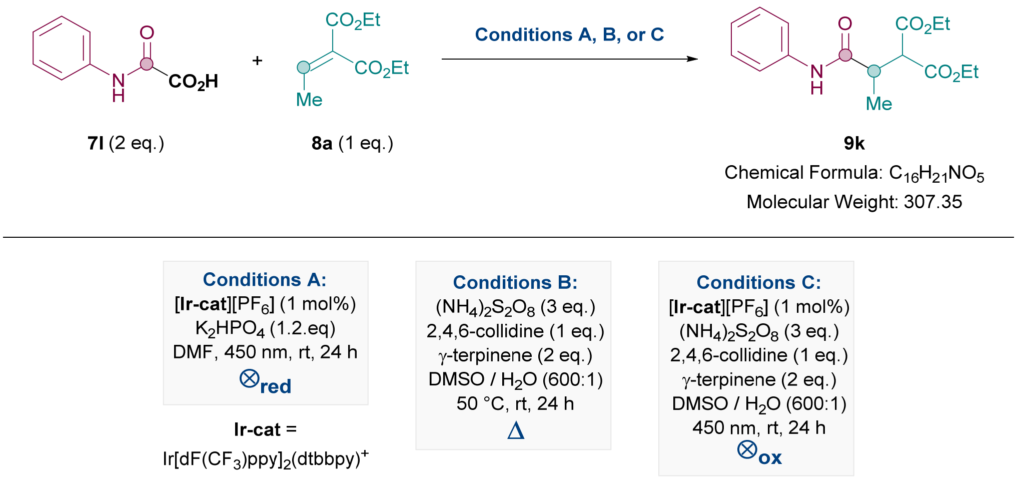

Using Conditions A: General procedure for Conditions A was followed. Diethyl 2-ethylidenemalonate **8a** (37.3 mg, 0.20 mmol, 1.0 eq.), *N*-(phenyl)-oxoacetic acid **7l** (66.0 mg, 0.40 mmol, 2.0 eq.), K_2HPO_4 (41.7 mg, 0.24 mmol, 1.2 eq.), Ir[dF(CF_3)ppy](dtbppy)PF_6 (2.3 mg, 2 µmol, 1 mol%) in dry DMF (0.5 mL) was irradiated with 450 nm for 24 h. The crude was then purified *via* column flash chromatography eluting with 70:30 hexane:EtOAc. The solvent was then removed *in vacuo* to afford diethyl 2-(1-oxo-1-(phenylamino)propan-2-yl)malonate **9k** as an off-white solid (24.8 mg, 0.094 mmol, 47%).

Using Conditions B: General procedure for Conditions B was followed. Diethyl 2-ethylidenemalonate **8a** (22.5 mg, 0.12 mmol, 1.0 eq.), *N*-(phenyl)-oxoacetic acid **7l** (40.0 mg, 0.24 mmol, 2.0 eq.), $(NH_4)_2S_2O_8$ (82.3 mg, 0.36 mmol, 3.0 eq.), γ-terpinene (38.5 µL, 0.24 mmol, 2.0 eq.), and 2,4,6-collidine (15.9 µL, 0.12 mmol, 1.0 eq.) in DMSO:H_2O (600:1) (0.4 mL) was heated at 50 ºC for 24 h followed by work-up X. The crude was then purified *via* column flash chromatography eluting with 85:15→70:30 hexane:EtOAc. The solvent was then removed *in vacuo* to afford diethyl 2-(1-oxo-1-(phenylamino)propan-2-yl)malonate **9k** as a white solid (29.2 mg, 0.095 mmol, 79%).

Using Conditions C: General procedure for Conditions C was followed. Diethyl 2-ethylidenemalonate **8a** (22.5 mg, 0.12 mmol, 1.0 eq.), *N*-(phenyl)-oxoacetic acid **7l** (39.9 mg, 0.24 mmol, 2.0 eq.), $(NH_4)_2S_2O_8$ (82.1 mg, 0.36 mmol, 3.0 eq.), Ir[dF(CF_3)ppy](dtbppy)PF_6 (1.5 mg, 1 µmol, 1 mol%), γ-terpinene (38.5 µL, 0.24 mmol, 2.0 eq.), and 2,4,6-collidine (15.9 µL, 0.12 mmol, 1.0 eq.) in DMSO:H_2O (600:1) (0.4 mL) was irradiated with 450 nm for 24 h.

The crude was then purified *via* column flash chromatography eluting with 70:30 hexane:EtOAc. The solvent was then removed *in vacuo* to afford diethyl 2-(1-oxo-1-(phenylamino)propan-2-yl)malonate **9k** as an off-white solid (21.3 mg, 0.068 mmol, 57%).

Characterisation:

^{1}H NMR (400 MHz, Chloroform-*d*): δ 7.89 (br s, 1H, NH), 7.49 (d, *J* = 7.4 Hz, 2H, ArH), 7.28 (t, *J* = 7.8 Hz, 2H, ArH), 7.07 (t, *J* = 7.4 Hz, 1H, ArH), 4.24 (q, *J* = 7.1 Hz, 2H, OCH_2), 4.29 – 4.07 (m, 2H, OCH_2), 3.81 (d, *J* = 9.9 Hz, 1H, C<u>H</u>(CO_2Et)), 3.14 (dq, *J* = 9.9, 7.0 Hz, 1H, MeC<u>H</u>), 1.29 (t, *J* = 7.0 Hz, 6H, 2 x OCH_2C<u>H</u>$_3$), 1.20 (t, *J* = 7.1 Hz, 3H, CHC<u>H</u>$_3$). **^{13}C NMR (101 MHz, Chloroform-*d*):** 172.2 (C), 168.7 (C), 168.6 (C), 138.1 (C), 129.0 (CH), 124.3 (CH), 120.0 (CH), 62.0 (CH_2), 61.9 (CH_2), 55.1 (CH), 41.7 (CH), 16.2 (CH_3), 14.2 (CH_3), 14.0 (CH_3). **IR:** ν_{max}/cm^{-1} 3308, 3079, 2977, 2909, 2852, 1740, 1644, 1548. **HRMS (ESI-TOF):** *m*/*z* $[M + H]^+$ calcd for $C_{16}H_{22}NO_5$, 308.14925; found, 308.1500. **TLC:** R_f = 0.32 (75:25 hexane:EtOAc). **M.p.** 94-97 °C.

Compound 9k – ^{1}H NMR (400 MHz, Chloroform-*d*):

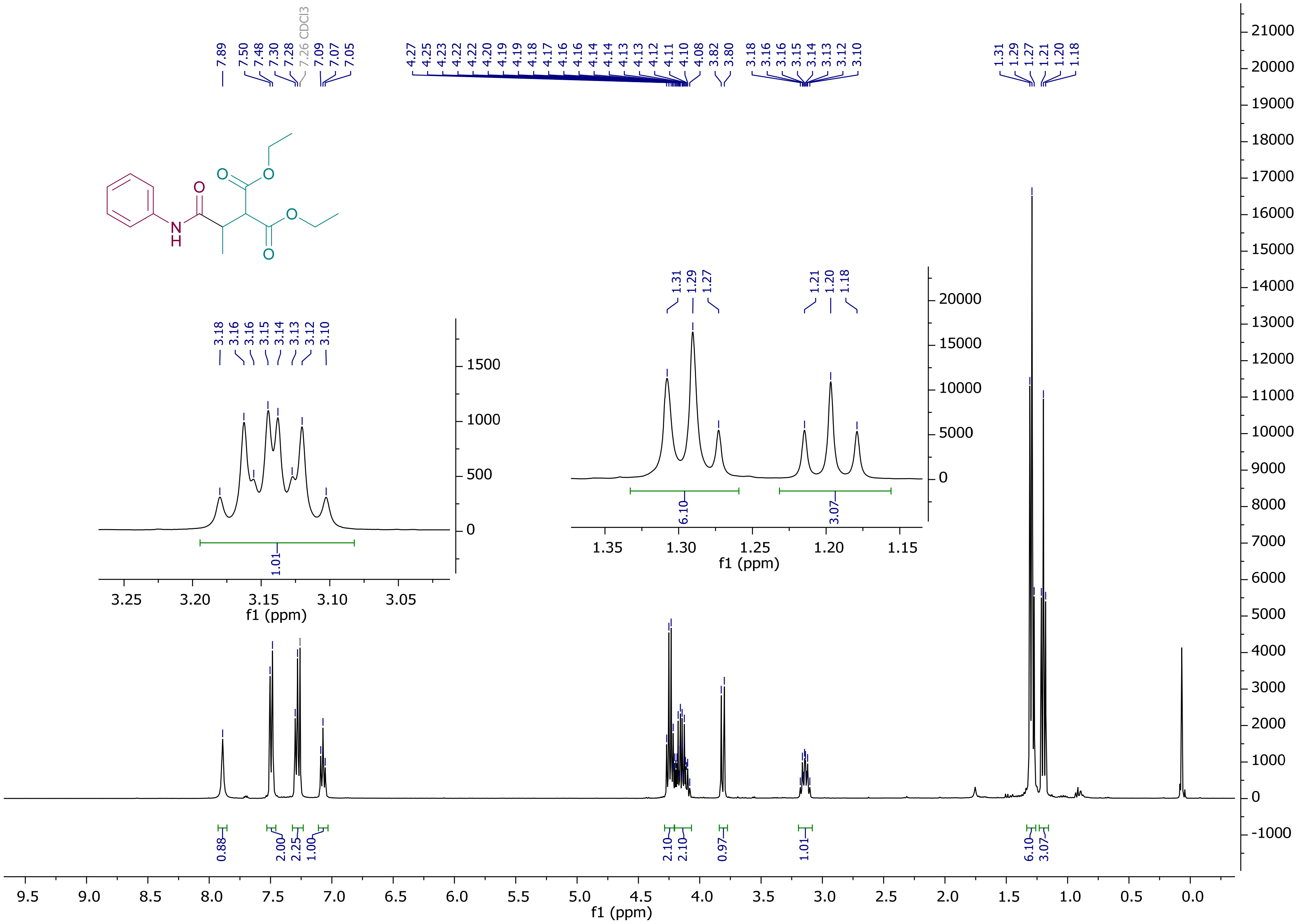

Compound 9k – ^{13}C NMR (101 MHz, Chloroform-*d*):

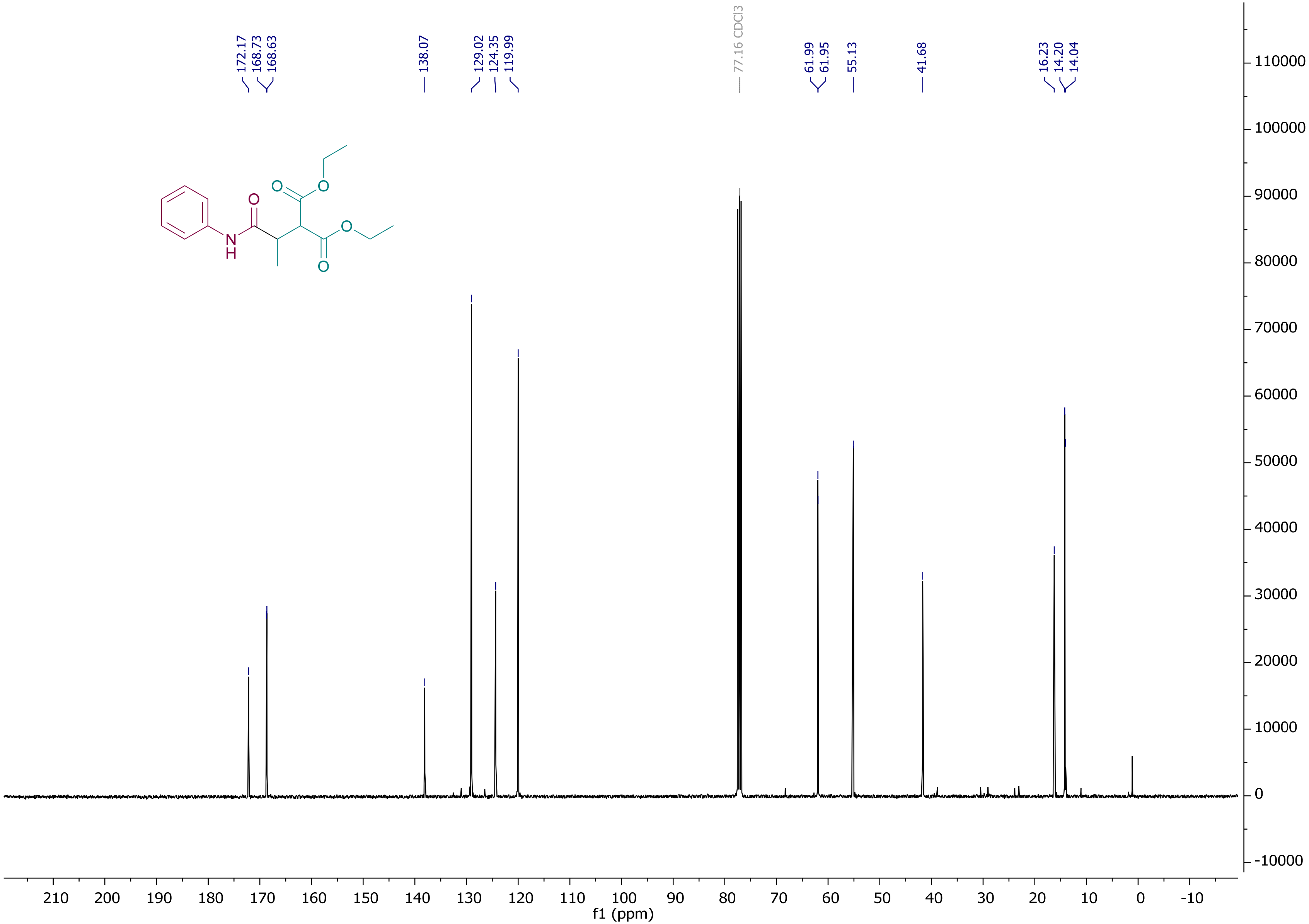

Diethyl 2-(1-((4-methoxyphenyl)amino)-1-oxopropan-2-yl)malonate (9l)

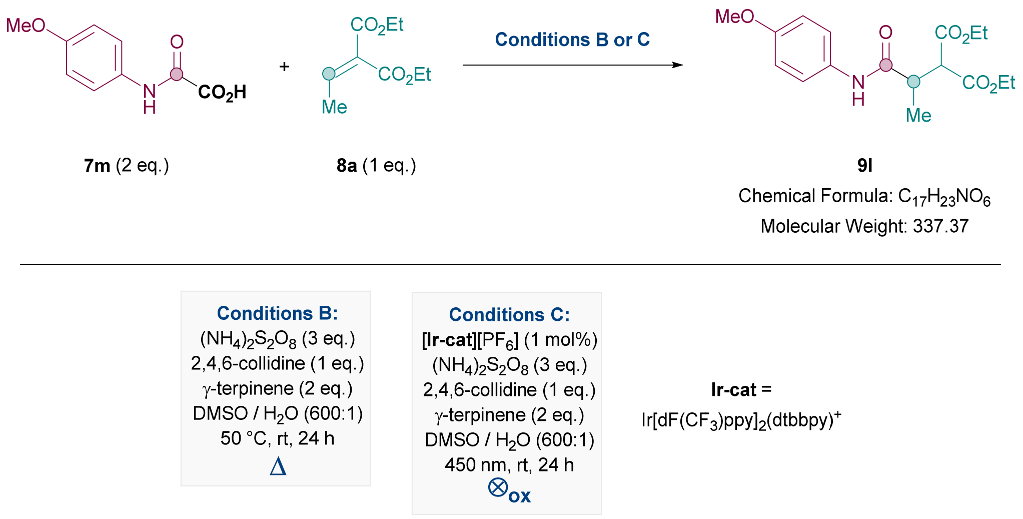

Using Conditions B: General procedure for Conditions B was followed. Diethyl 2-ethylidenemalonate **8a** (22.5 mg, 0.12 mmol, 1.0 eq.), *N*-(4-methoxyphenyl)-oxoacetic acid **7m** (47.1 mg, 0.24 mmol, 2.0 eq.), $(NH_4)_2S_2O_8$ (82.2 mg, 0.36 mmol, 3.0 eq.), γ-terpinene (38.5 µL, 0.24 mmol, 2.0 eq.), and 2,4,6-collidine (15.9 µL, 0.12 mmol, 1.0 eq.) in DMSO:H_2O (600:1) (0.4 mL) was heated at 50 ºC for 24 h followed by work-up X. The crude was then purified *via* column flash chromatography eluting with 95:5→70:30 hexane:EtOAc. The solvent was then removed *in vacuo* to afford diethyl 2-(1-((4-methoxyphenyl)amino)-1-oxopropan-2-yl)malonate **9l** as a dark red solid (28.2 mg, 0.084 mmol, 70%).

Using Conditions C: General procedure for Conditions C was followed. Diethyl 2-ethylidenemalonate **8a** (23.0 mg, 0.12 mmol, 1.0 eq.), *N*-(4-methoxyphenyl)-oxoacetic acid **7m** (47.5 mg, 0.24 mmol, 2.0 eq.), $(NH_4)_2S_2O_8$ (83.0 mg, 0.36 mmol, 3.0 eq.), Ir[dF(CF_3)ppy](dtbppy)PF_6 (1.4 mg, 1 µmol, 1 mol%), γ-terpinene (38.5 µL, 0.24 mmol, 2.0 eq.), and 2,4,6-collidine (15.9 µL, 0.12 mmol, 1.0 eq.) in DMSO:H_2O (600:1) (0.4 mL) was irradiated with 450 nm for 24 h. The crude was then purified *via* column flash chromatography eluting with 65:35 hexane:EtOAc. The solvent was then removed *in vacuo* to afford diethyl 2-(1-((4-methoxyphenyl)amino)-1-oxopropan-2-yl)malonate **9l** as an off-white solid (31.1 mg, 0.090 mmol, 75%).

Characterisation:

^{1}H NMR (400 MHz, Chloroform-*d*): δ ppm 7.77 (br s, 1H, NH), 7.38 (d, *J* = 9.0 Hz, 2H, ArH), 6.81 (d, *J* = 9.0 Hz, 2H, ArH), 4.23 (q, *J* = 7.1 Hz, 2H, OCH_2), 4.22 – 4.05 (m, 2H, OCH_2), 3.80 (d, *J* = 10.0 Hz, 1H, C<u>H</u>(CO_2Et)$_2$), 3.76 (s, 3H, OCH_3), 3.10 (dq, *J* = 10.0, 7.0 Hz, 1H, MeC<u>H</u>), 1.29 (t, *J* = 7.1, 3H, $OCH_2C\underline{H}_3$), 1.27 (t, *J* = 7.1, 3H, $OCH_2C\underline{H}_3$), 1.20 (t, *J* = 7.1 Hz, 3H, $CHC\underline{H}_3$). **^{13}C NMR (101 MHz, Chloroform-*d*):** δ ppm 172.0 (C), 168.72 (C), 168.66 (C), 156.5 (C), 131.2 (CH), 121.8 (CH), 114.2 (CH), 61.93 (CH_2), 61.89 (CH_2), 55.6 (CH_3), 55.1 (CH), 41.5 (CH), 16.2 (CH_3), 14.2 (CH_3), 14.0 (CH_3). **IR:** ν_{max}/cm^{-1} 3307, 3079, 2978, 2909, 2852, 1739, 1723, 1644, 1549. **HRMS (ESI-TOF):** *m/z* $[M + H]^+$ calcd for $C_{17}H_{24}NO_6$, 338.15981; found, 338.16010. **TLC:** R_f = 0.3 (75:25 hexane:EtOAc). **M.p.** 93-96 °C.

Compound 9l – ¹H NMR (400 MHz, Chloroform-*d*):

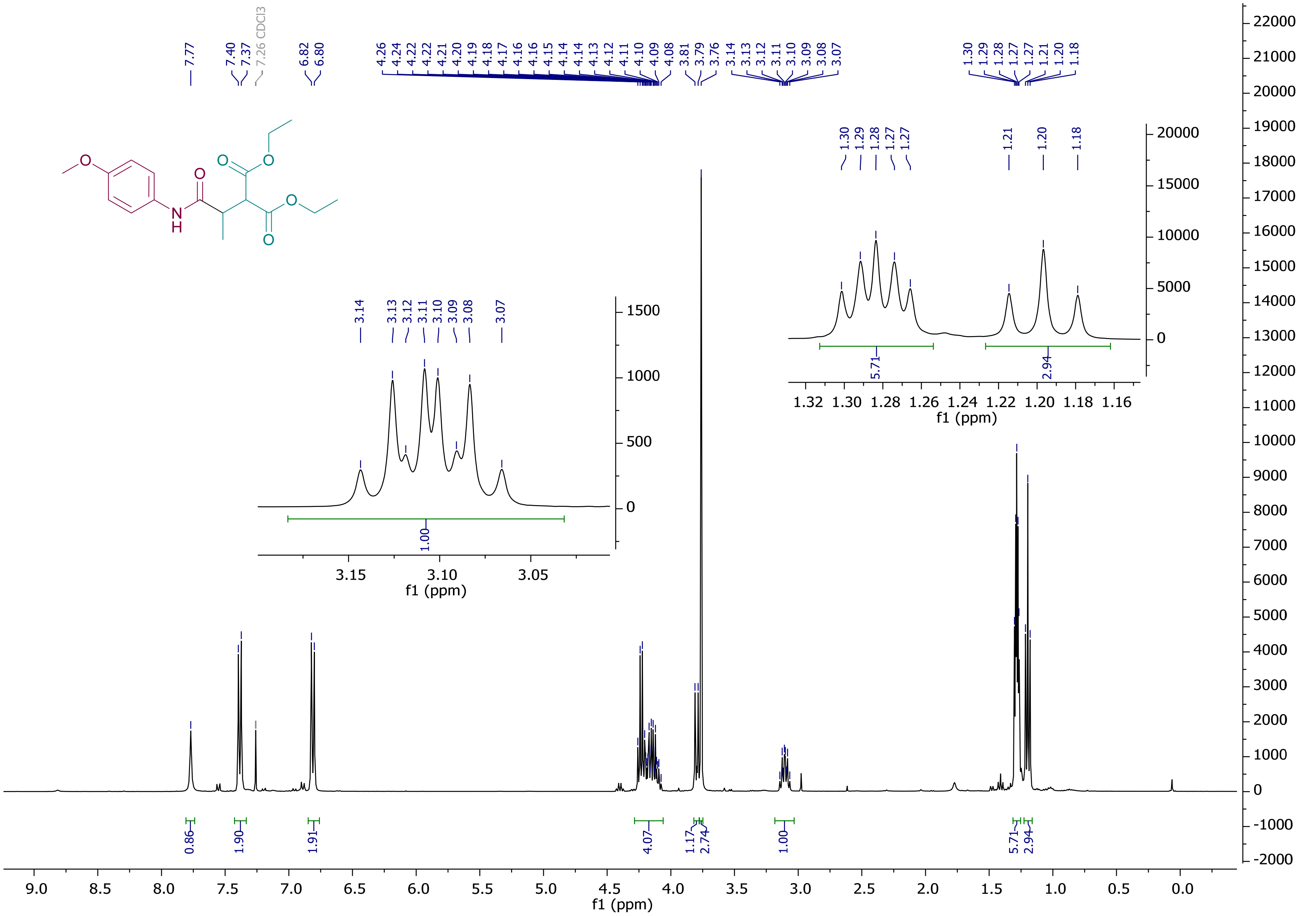

Compound 9l – ^{13}C NMR (101 MHz, Chloroform-*d*):

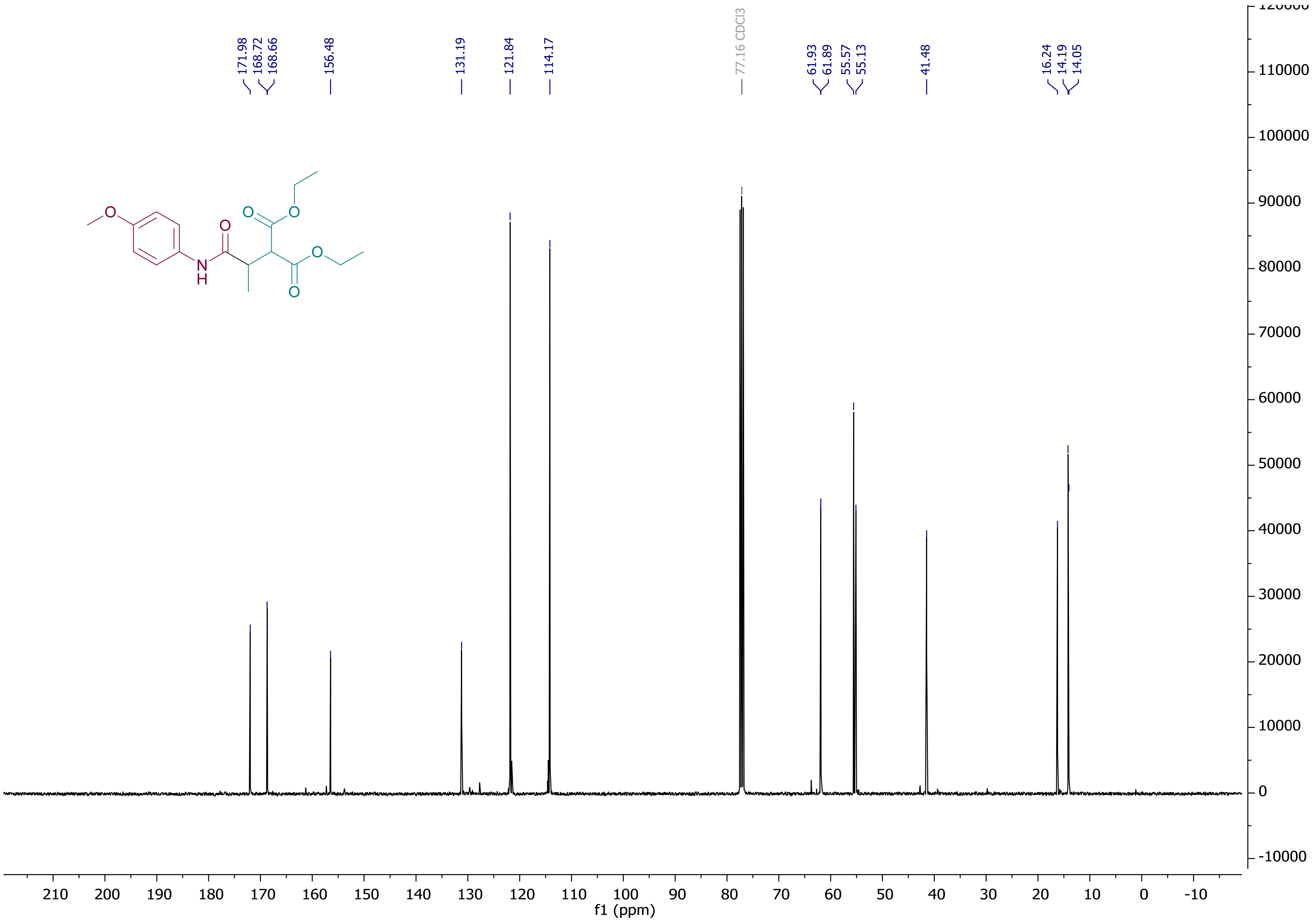

Diethyl 2-(1-oxo-1-((4-butyl)phenylamino)propan-2-yl)malonate (9m)

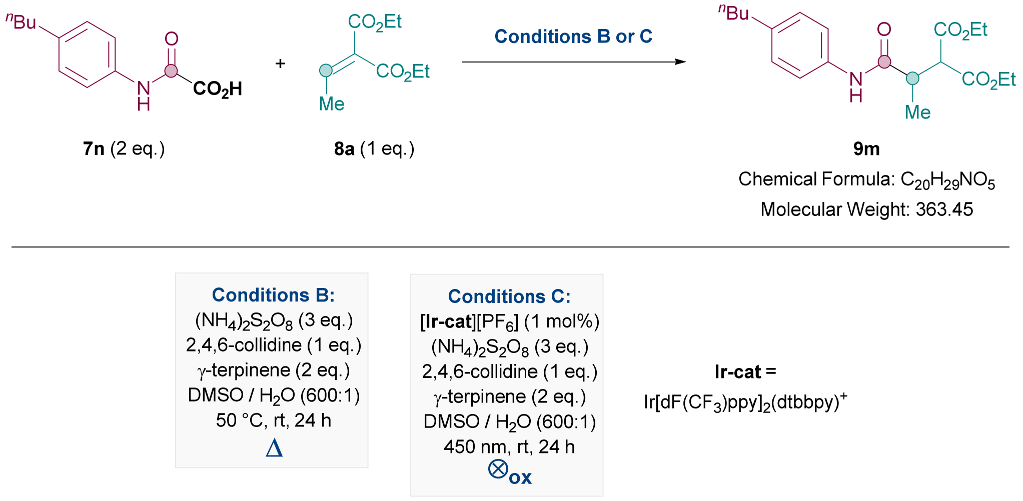

Using Conditions B: General procedure for Conditions B was followed. Diethyl 2-ethylidenemalonate **8a** (22.4 mg, 0.12 mmol, 1.0 eq.), *N*-((4-butyl)-phenyl)-oxoacetic acid **7n** (53.8 mg, 0.24 mmol, 2.0 eq.), $(NH_4)_2S_2O_8$ (82.7 mg, 0.36 mmol, 3.0 eq.), γ-terpinene (38.5 μL, 0.24 mmol, 2.0 eq.), and 2,4,6-collidine (15.9 μL, 0.12 mmol, 1.0 eq.) in DMSO:H_2O (600:1) (0.4 mL) was heated at 50 ºC for 24 h followed by work-up X. The crude was then purified *via* column flash chromatography eluting with 80:20 hexane:EtOAc. The solvent was then removed *in vacuo* to afford diethyl 2-(1-oxo-1-((4-butyl)phenylamino)propan-2-yl)malonate **9m** as a brown oil (30.1 mg, 0.083 mmol, 69%).

Using Conditions C: General procedure for Conditions C was followed. Diethyl 2-ethylidenemalonate **8a** (22.4 mg, 0.12 mmol, 1.0 eq.), *N*-((4-butyl)-phenyl)-oxoacetic acid **7n** (53.2 mg, 0.24 mmol, 2.0 eq.), $(NH_4)_2S_2O_8$ (82.3 mg, 0.36 mmol, 3.0 eq.), Ir[dF(CF_3)ppy](dtbppy)PF_6 (1.4 mg, 1 μmol, 1 mol%), γ-terpinene (38.5 μL, 0.24 mmol, 2.0 eq.), and 2,4,6-collidine (15.9 μL, 0.12 mmol, 1.0 eq.) in DMSO:H_2O (600:1) (0.4 mL) was irradiated with 450 nm for 24 h. The crude was then purified *via* column flash chromatography eluting with 75:25 hexane:EtOAc. The solvent was then removed *in vacuo* to afford diethyl 2-(1-oxo-1-((4-butyl)phenylamino)propan-2-yl)malonate **9m** as an off-white solid (19.3 mg, 0.053 mmol, 44%).

Characterisation:

^{1}H NMR (400 MHz, Chloroform-*d*): δ ppm 7.80 (br s, 1H, NH), 7.39 (d, *J* = 8.4 Hz, 2H, ArH), 7.09 (d, *J* = 8.4 Hz, 2H, ArH), 4.24 (q, *J* = 7.1 Hz, 2H, OCH_2), 4.29 – 3.97 (m, 2H, OCH_2), 3.81 (d, *J* = 10.0 Hz, 1H, C$\underline{H}$(CO_2Et)$_2$), 3.12 (dq, *J* = 10.0, 7.0 Hz, 1H, MeC$\underline{H}$), 2.55 (t, *J* = 7.7 Hz, 2H, CH_2), 1.61 – 1.49 (m, 2H, CH_2), 1.37 – 1.31 (m, 2H, CH_2), 1.31-1.27 (m, 6H, 2 x $OCH_2C\underline{H}_3$), 1.20 (t, *J* = 7.1 Hz, 3H, CH_3), 0.90 (t, *J* = 7.3 Hz, 3H, CH_3). **^{13}C NMR (101 MHz, Chloroform-*d*):** δ ppm 172.0 (C), 168.7 (C), 168.6 (C), 139.1 (C), 135.6 (CH), 128.9 (CH), 120.1 (CH), 61.94 (CH_2), 61.90 (CH_2), 55.1 (CH), 41.6 (CH), 35.2 (CH_2), 33.8 (CH_2), 22.4 (CH_2), 16.2 (CH_3), 14.2 (CH_2), 14.05 (CH_3), 14.03 (CH_3). **IR:** ν_{max}/cm^{-1} 3321, 2958, 2932, 2872, 2858, 1734, 1688, 1602, 1533, 1464, 1368, 1306, 1277. **HRMS (ESI-TOF):** *m*/*z* [M + H]$^+$ calcd for $C_{20}H_{30}NO_5$, 364.21185; found, 364.2123. **TLC:** R_f = 0.26 (80:20 hexane:EtOAc).

Compound 9m – ^{1}H NMR (400 MHz, Chloroform-*d*):

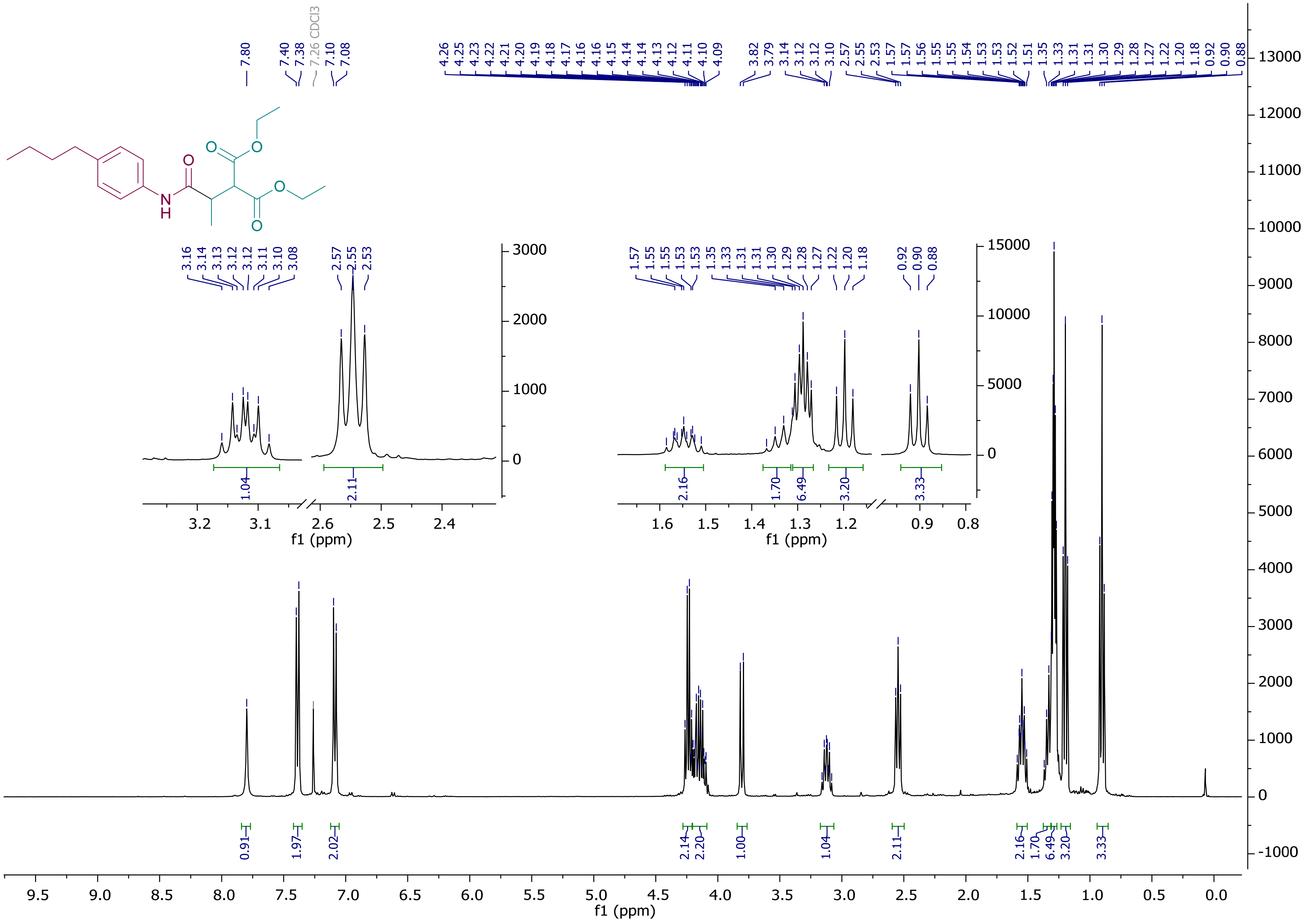

Compound 9m – ^{13}C NMR (101 MHz, Chloroform-*d*):

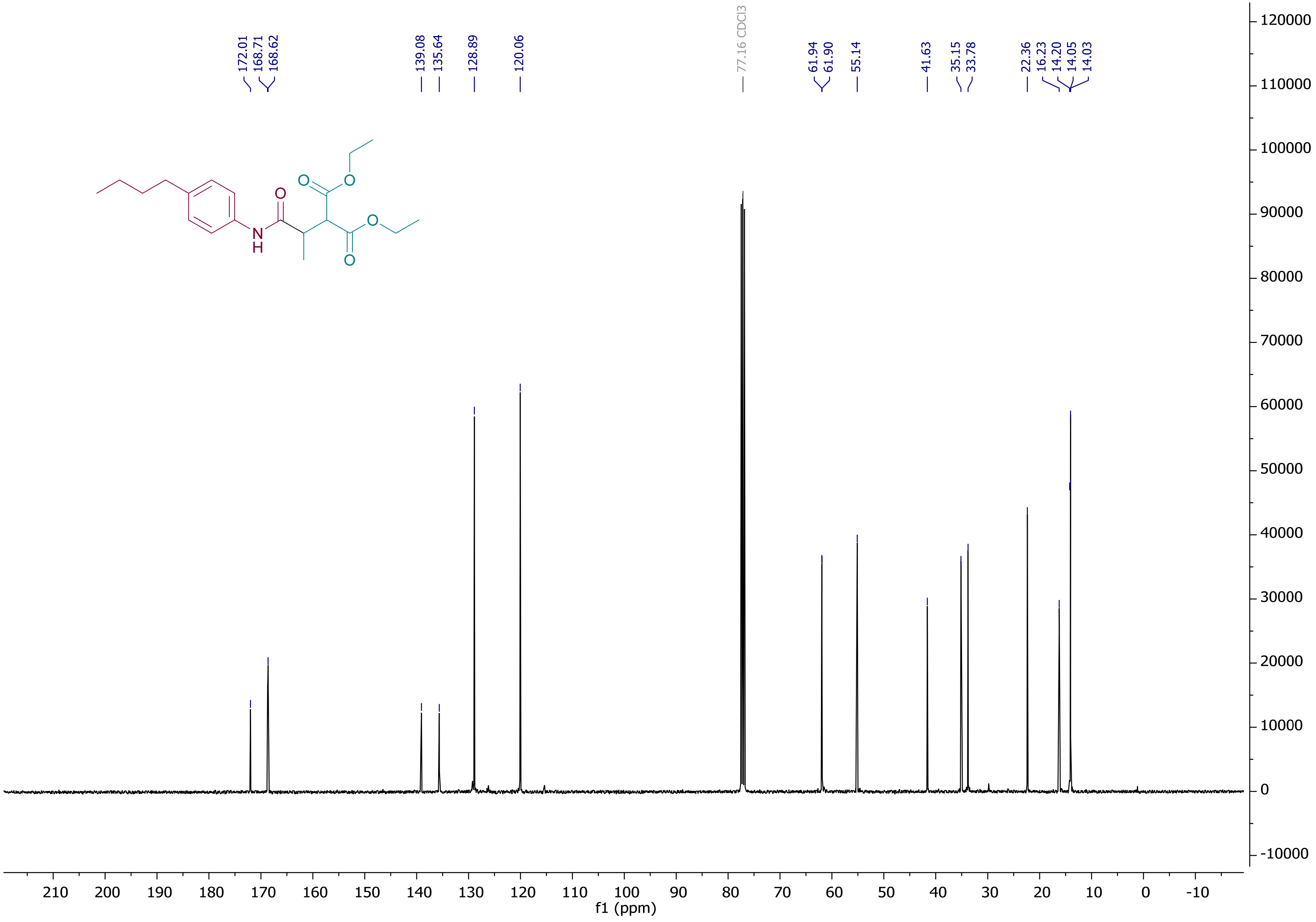

Diethyl 2-(1-oxo-1-((4-*t*-butyl)phenylamino)propan-2-yl)malonate (9n)

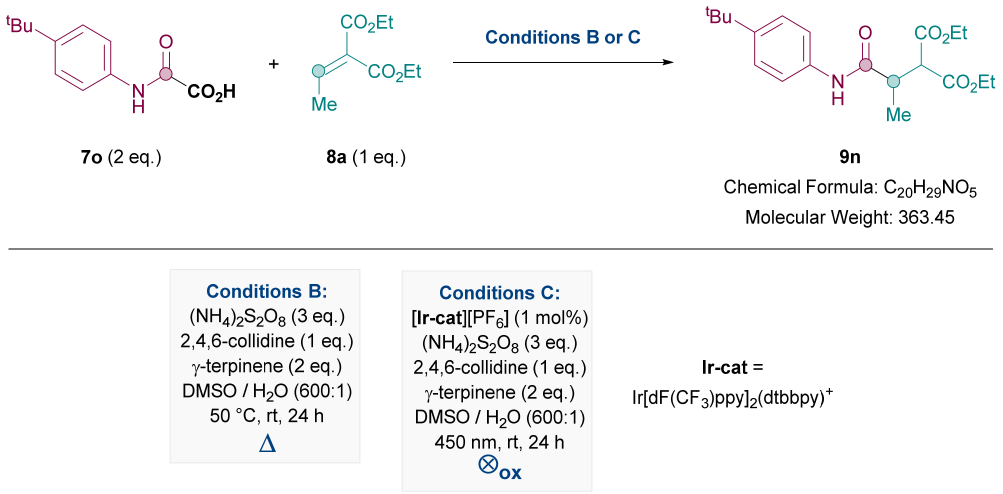

Using Conditions B: General procedure for Conditions B was followed. Diethyl 2-ethylidenemalonate **8a** (22.5 mg, 0.12 mmol, 1.0 eq.), *N*-((4-*t*-butyl) phenyl)-oxoacetic acid **7o** (53.1 mg, 0.24 mmol, 2.0 eq.), $(NH_4)_2S_2O_8$ (82.3 mg, 0.36 mmol, 3.0 eq.), γ-terpinene (38.5 μL, 0.24 mmol, 2.0 eq.), and 2,4,6-collidine (15.9 μL, 0.12 mmol, 1.0 eq.) in DMSO:H_2O (600:1) (0.4 mL) was heated at 50 ºC for 24 h followed by work up X. The crude was then purified *via* column flash chromatography eluting with 75:25 hexane:EtOAc. The solvent was then removed *in vacuo* to afford diethyl 2-(1-oxo-1-((4-*t*-butyl)phenylamino)propan-2-yl)malonate **9n** as a brown oil (32.4 mg, 0.089 mmol, 74%).

Using Conditions C: General procedure for Conditions C was followed. Diethyl 2-ethylidenemalonate **8a** (22.5 mg, 0.12 mmol, 1.0 eq.), *N*-((4-*t*-butyl) phenyl)-oxoacetic acid **7o** (53.0 mg, 0.24 mmol, 2.0 eq.), $(NH_4)_2S_2O_8$ (82.0 mg, 0.36 mmol, 3.0 eq.), Ir[dF(CF_3)ppy](dtbppy)PF_6 (1.5 mg, 1 μmol, 1 mol%), γ-terpinene (38.5 μL, 0.24 mmol, 2.0 eq.), and 2,4,6-collidine (15.9 μL, 0.12 mmol, 1.0 eq.) in DMSO:H_2O (600:1) (0.4 mL) was irradiated with 450 nm for 24 h. The crude was then purified *via* column flash chromatography eluting with 70:30 hexane:EtOAc. The solvent was then removed *in vacuo* to afford diethyl 2-(1-oxo-1-((4-*t*-butyl)phenylamino)propan-2-yl)malonate **9n** as an off-white solid (35.6 mg, 0.098 mmol, 82%).

Characterisation:

^{1}H NMR (400 MHz, Chloroform-*d*): δ ppm 7.81 (br s, 1H, NH), 7.42 (d, *J* = 8.7 Hz, 2H, ArH), 7.30 (d, *J* = 8.7 Hz, 1H, ArH), 4.24 (q, *J* = 7.1 Hz, 2H, OCH_2), 4.22 – 4.06 (m, 2H, OCH_2), 3.81 (d, *J* = 10.0 Hz, 1H, C$\underline{H}$(CO_2Et)$_2$), 3.13 (dq, *J* = 10.0, 7.0 Hz, 1H, MeC$\underline{H}$), 1.33 – 1.25 (m, 6H, 2 x $OCH_2C\underline{H}_3$), 1.28 (s, 9H, 3 x CH_3), 1.20 (t, *J* = 7.1 Hz, 3H, $CHC\underline{H}_3$). **^{13}C NMR (101 MHz, Chloroform-*d*):** δ ppm 172.1 (C), 168.7 (C), 168.6 (C), 147.3 (C), 135.4 (CH), 125.8 (CH), 119.8 (CH), 61.93 (CH_2), 61.89 (CH_2), 55.1 (CH), 41.6 (CH), 34.4 (C), 31.5 (CH_3), 16.2 (CH_3), 14.2 (CH_3), 14.1 (CH_3). **IR:** ν_{max}/cm^{-1} 3314, 2962, 2904, 2870, 1734, 1690, 1660, 1601, 1533, 1463, 1367, 1300. **HRMS (ESI-TOF):** *m*/*z* [M + H]$^+$ calcd for $C_{20}H_{30}NO_5$, 364.21185; found, 364.2127. **TLC:** R_f = 0.21 (80:20 hexane:EtOAc).

Compound 9n – ^{1}H NMR (400 MHz, Chloroform-*d*):

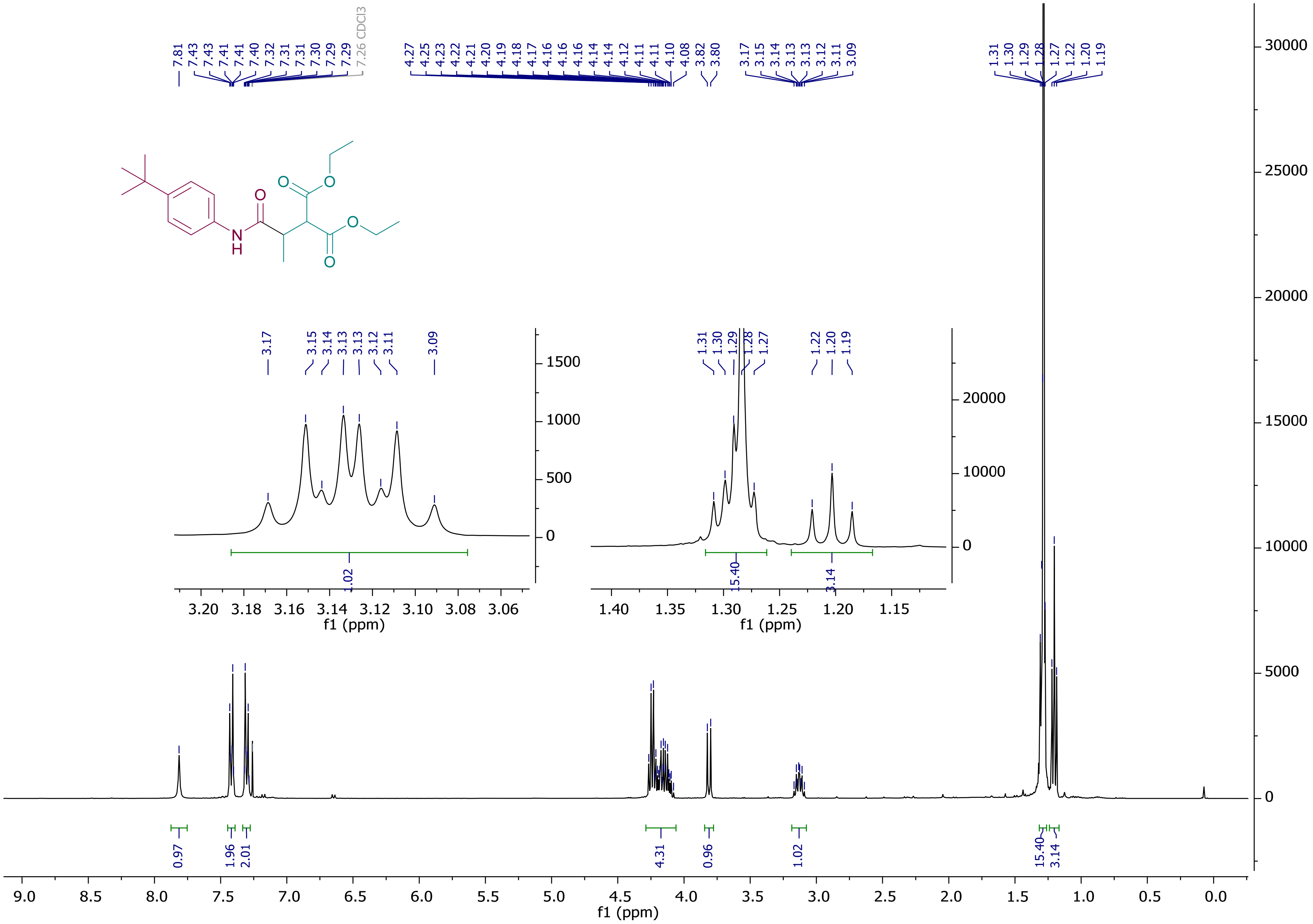

Compound 9n – ^{13}C NMR (101 MHz, Chloroform-*d*):

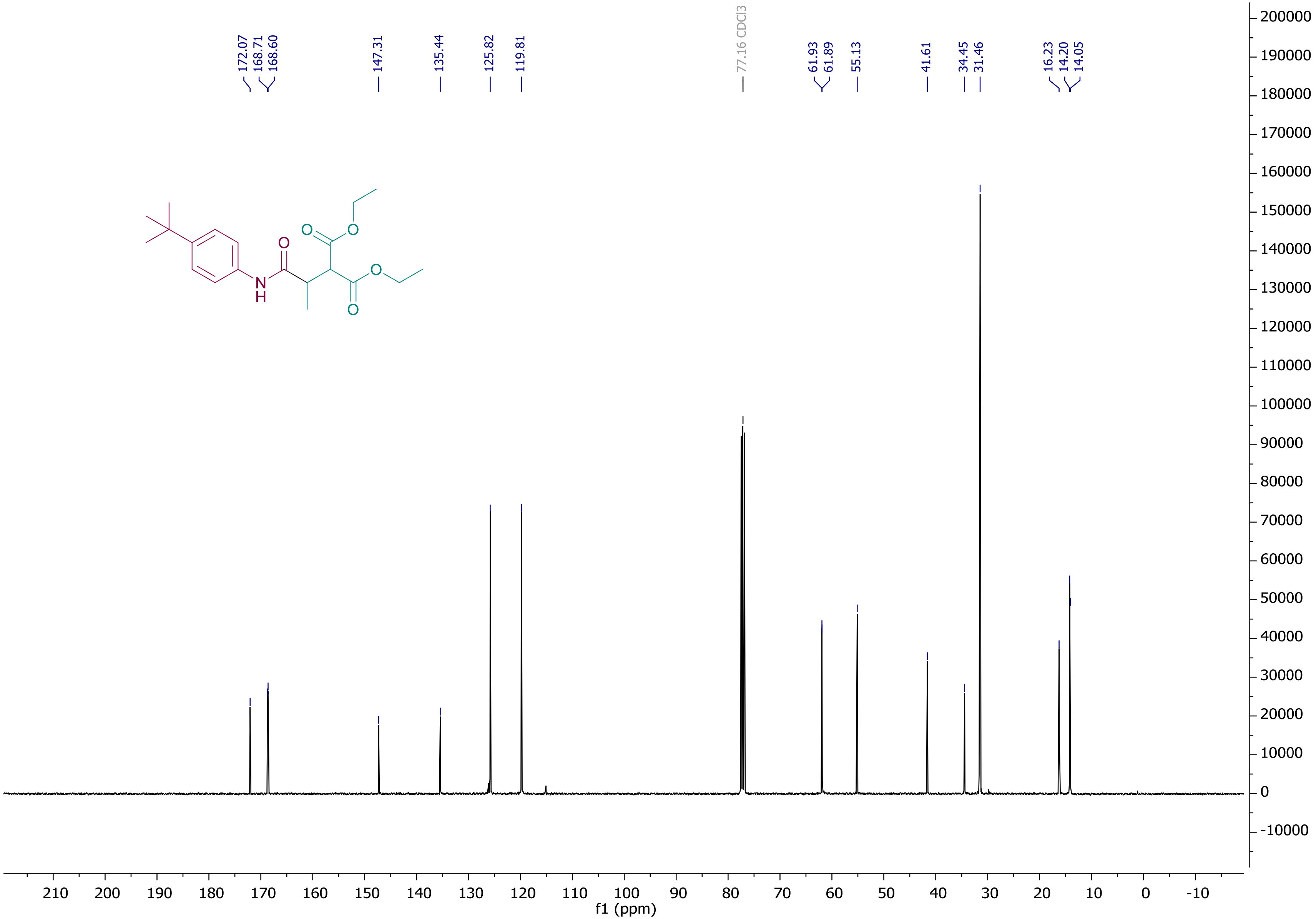

Diethyl 2-(1-((4-chlorophenyl)amino)-1-oxopropan-2-yl)malonate (9o)

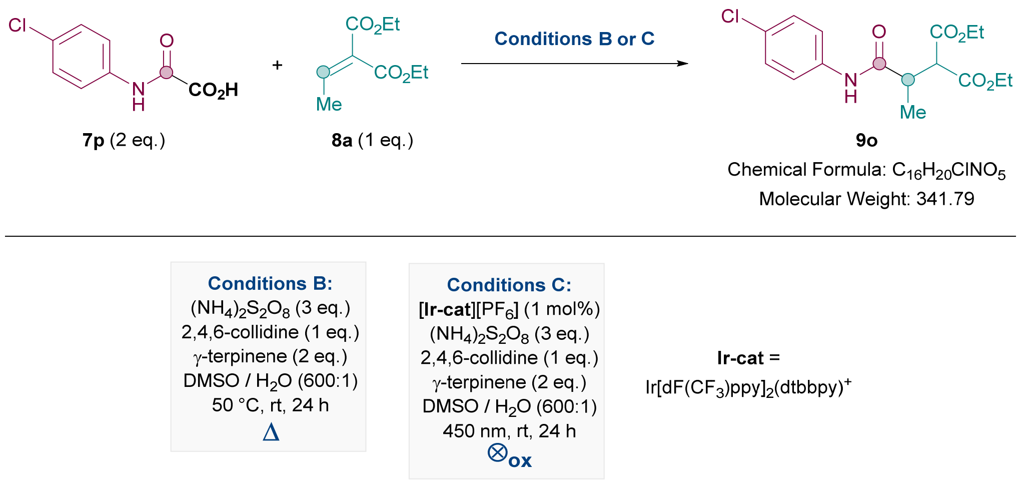

Using Conditions B: General procedure for Conditions B was followed. Diethyl 2-ethylidenemalonate **8a** (22.3 mg, 0.12 mmol, 1.0 eq.), *N*-(4-chlorophenyl)-oxoacetic acid **7p** (72.2 mg, 0.36 mmol, 3.0 eq.), $(NH_4)_2S_2O_8$ (109.7 mg, 0.48 mmol, 4.0 eq.), γ-terpinene (38.5 µL, 0.24 mmol, 2.0 eq.), and 2,4,6-collidine (47.7 µL, 0.36 mmol, 3.0 eq.) in DMSO:H_2O (600:1) (0.4 mL) was heated at 75 °C for 24 h followed by work-up X. The crude was then purified *via* column flash chromatography eluting with 75:25 hexane:EtOAc. The solvent was then removed *in vacuo* to afford diethyl 2-(1-((4-chlorophenyl)amino)-1-oxopropan-2-yl)malonate **9o** as a brown solid (20.3 mg, 0.059 mmol, 49%).

Using Conditions C: General procedure for Conditions C was followed. Diethyl 2-ethylidenemalonate **8a** (22.3 mg, 0.12 mmol, 1.0 eq.), *N*-(4-chlorophenyl)-oxoacetic acid **7p** (23.9 mg, 0.24 mmol, 2.0 eq.), $(NH_4)_2S_2O_8$ (82.5 mg, 0.36 mmol, 3.0 eq.), Ir[dF(CF_3)ppy](dtbppy)PF_6 (1.5 mg, 1 µmol, 1 mol%), γ-terpinene (38.5 µL, 0.24 mmol, 2.0 eq.), and 2,4,6-collidine (15.9 µL, 0.12 mmol, 1.0 eq.) in DMSO:H_2O (600:1) (0.4 mL) was irradiated with 450 nm for 24 h. The crude was then purified *via* column flash chromatography eluting with 70:30 hexane:EtOAc. The solvent was then removed *in vacuo* to afford diethyl 2-(1-((4-chlorophenyl)amino)-1-oxopropan-2-yl)malonate **9o** as an off-white solid (20.2 mg, 0.059 mmol, 49%).

Characterisation:

^{1}H NMR (300 MHz, Chloroform-*d*): δ ppm 7.95 (br s, 1H, NH), 7.44 (d, *J* = 8.9 Hz, 1H, ArH), 7.23 (d, *J* = 8.9 Hz, 2H, ArH), 4.24 (q, *J* = 7.1 Hz, 2H, OCH_2), 4.26 – 4.04 (m, 2H, OCH_2), 3.79 (d, *J* = 9.7 Hz, 1H, C$\underline{H}$(CO_2Et)$_2$), 3.12 (dq, *J* = 9.6, 7.0 Hz, 1H, MeC$\underline{H}$), 1.30 (t, *J* = 7.2 Hz, 3H, $OCH_2C\underline{H}_3$), 1.28 (t, *J* = 7.2 Hz, 3H, $OCH_2C\underline{H}_3$), 1.20 (t, *J* = 7.1 Hz, 3H, $CHC\underline{H}_3$). **^{13}C NMR (75 MHz, Chloroform-*d*):** δ ppm 172.2 (C), 168.7 (C plus one overlapping C), 136.7 (C), 129.3 (CH), 129.0 (CH), 121.2 (CH), 62.1 (CH_2), 62.0 (CH_2), 55.1 (CH), 41.6 (CH), 16.2 (CH_3), 14.2 (CH_3), 14.1 (CH_3). **IR:** ν_{max}/cm^{-1} 3251, 3192, 3126, 2982, 1745, 1734, 1699, 1652, 1596, 1493, 1402, 1309, 1297. **HRMS (ESI-TOF):** *m*/*z* [M + H]$^+$ calcd for $C_{16}H_{21}NO_5{}^{35}Cl$, 342.11028; found, 342.1111. **TLC:** R_f = 0.25 (25% EtOAc). **m.p.** 72-75 °C.

Compound 9o – ^{1}H NMR (300 MHz, Chloroform-*d*):

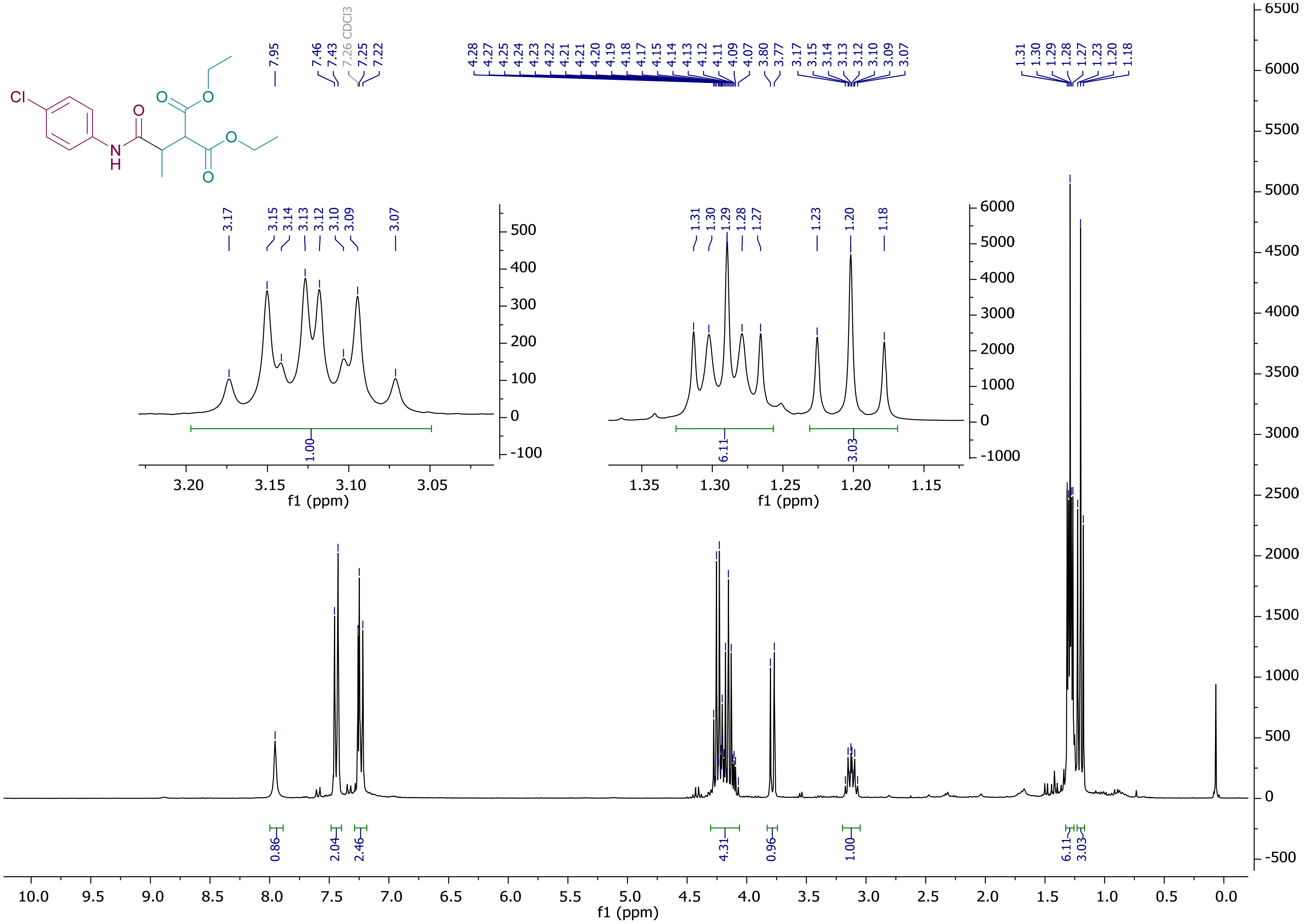

Compound 9o – ^{13}C NMR (75 MHz, Chloroform-*d*):

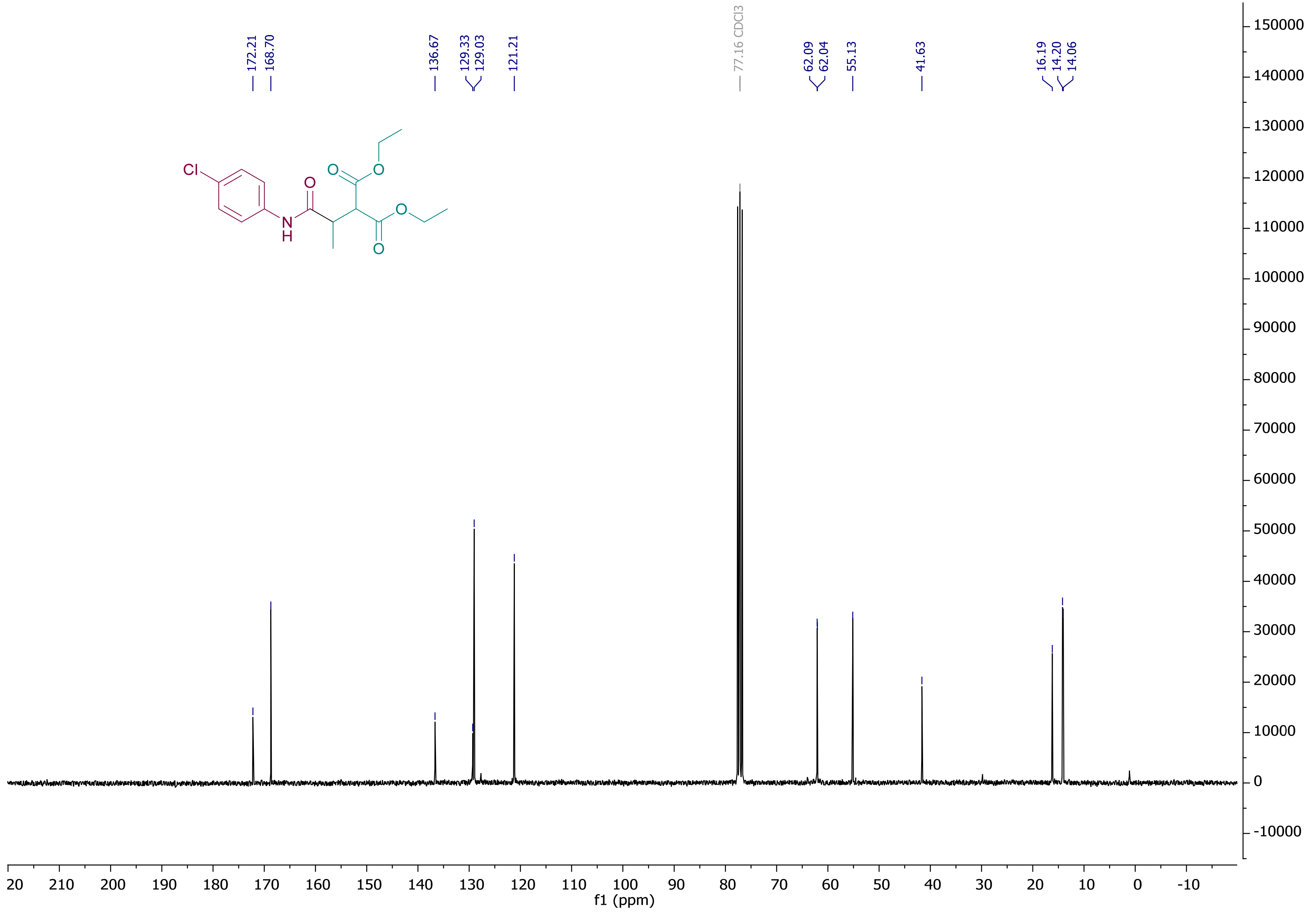

Diethyl 2-(1-(dibutylamino)-1-oxopropan-2-yl)malonate (9p)

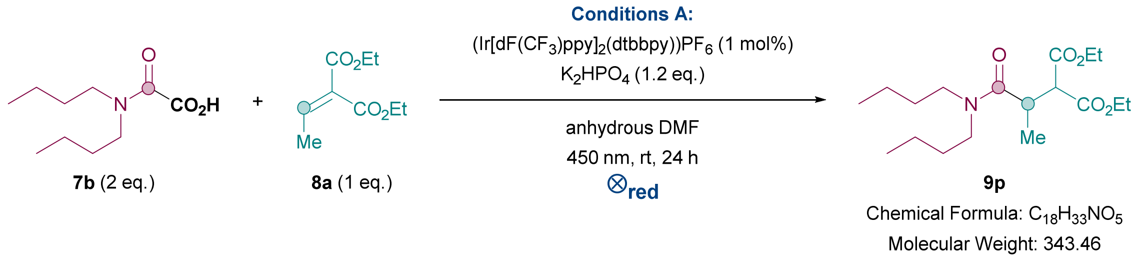

General procedure for Conditions A was followed. Diethyl 2-ethylidenemalonate **8a** (22.4 mg, 0.12 mmol, 1.0 eq.), *N*,*N*-(dibutyl)-oxoacetic acid **7b** (48.7 mg, 0.24 mmol, 2.0 eq.), K_2HPO_4 (25.3 mg, 0.14 mmol, 1.2 eq.), Ir[dF(CF_3)ppy](dtbppy)PF_6 (1.3 mg, 1 µmol, 1 mol%) in dry DMF was irradiated for 24 h. The crude was then purified *via* column flash chromatography eluting with 80:20 hexane:EtOAc. The solvent was then removed *in vacuo* to afford diethyl 2-(1-(dibutylamino)-1-oxopropan-2-yl)malonate **9p** as a green oil (31.0 mg, 0.090 mmol, 75%).

Characterisation:

^{1}H NMR (300 MHz, Chloroform-*d*): δ ppm 4.24 – 4.03 (m, 4H, 2 x OCH_2), 3.85 (d, J = 10.8 Hz, 1H, C<u>H</u>CO(OEt)$_2$), 3.43 – 3.11 (m, 5H, C<u>H</u>Me and 2 x CH_2), 1.80 – 1.54 (m, 2H, CH_2), 1.52 – 1.33 (m, 4H, 2 x CH_2), 1.31 – 1.24 (m, 5H, CH_2 and CH_3), 1.20 (t, J = 7.1 Hz, 4H, CH_3), 1.11 (d, J = 7.0 Hz, 3H, CH_3), 0.96 (t, J = 7.3 Hz, 3H, CH_3), 0.89 (t, J = 7.3 Hz, 3H, CH_3). **^{13}C NMR (75 MHz, Chloroform-*d*):** δ ppm 173.8 (C), 169.1 (C), 168.6 (C), 61.6 (CH_2), 61.5 (CH_2), 55.8 (CH), 47.9 (CH_2), 46.2 (CH_2), 36.2 (CH), 31.2 (CH_2), 29.8 (CH_2), 20.29 (CH_2), 20.27 (CH_2), 16.3 (CH_3), 14.2 (CH_3), 14.1 (CH_3), 14.0 (CH_3), 13.9 (CH_3). **IR:** ν_{max}/cm^{-1} 2960, 2934, 2873, 1750, 1733, 1637, 1464, 1429, 1368, 1296, 1277. **HRMS (ESI-TOF):** *m/z* [M + H]$^+$ calcd for $C_{18}H_{34}NO_5$, 344.24315; found, 344.2440. **TLC:** R_f = 0.32 (80:20 hexane:EtOAc).

Compound 9p – ^{1}H NMR (300 MHz, Chloroform-*d*):

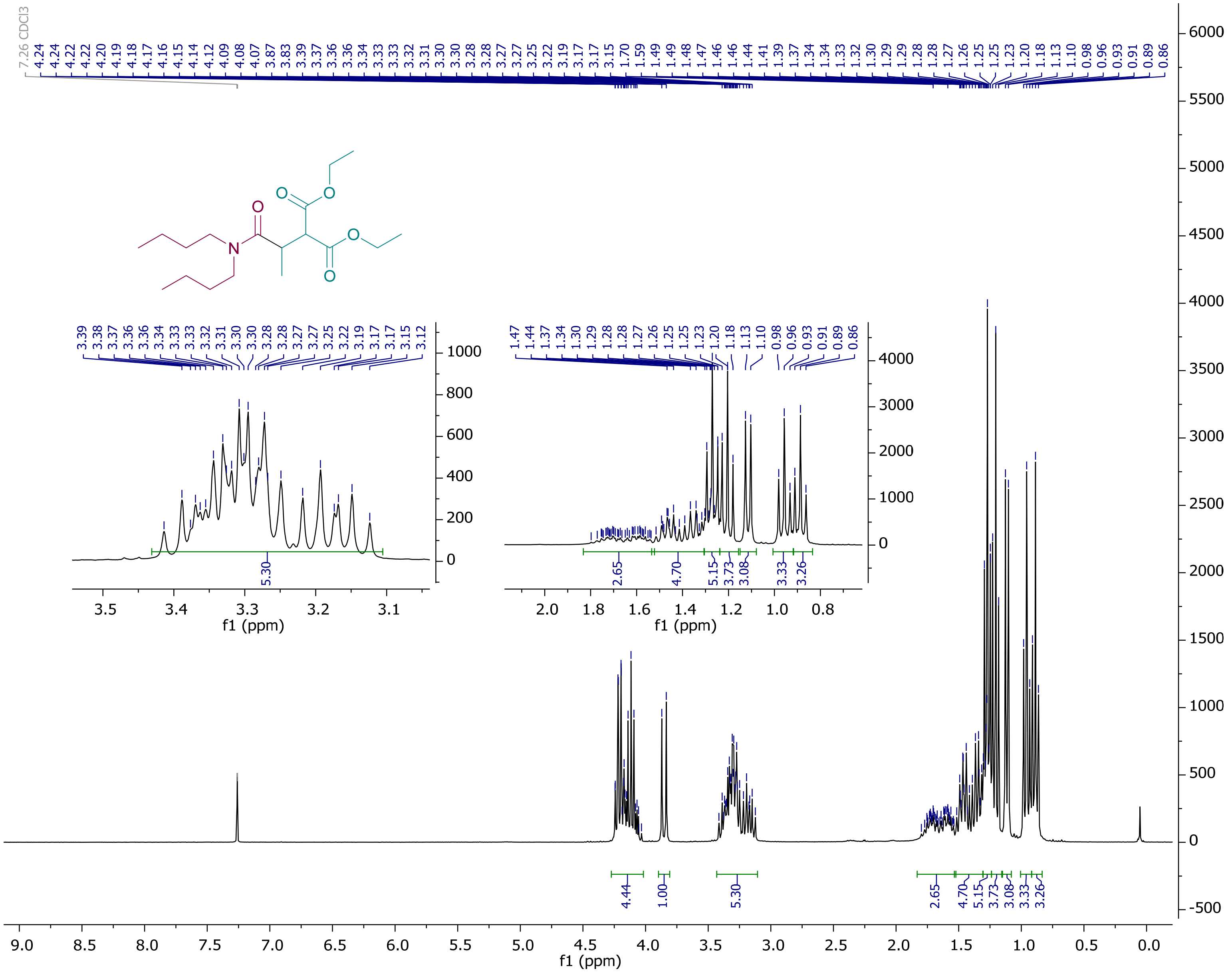

Compound 9p – ^{13}C NMR (75 MHz, Chloroform-*d*):

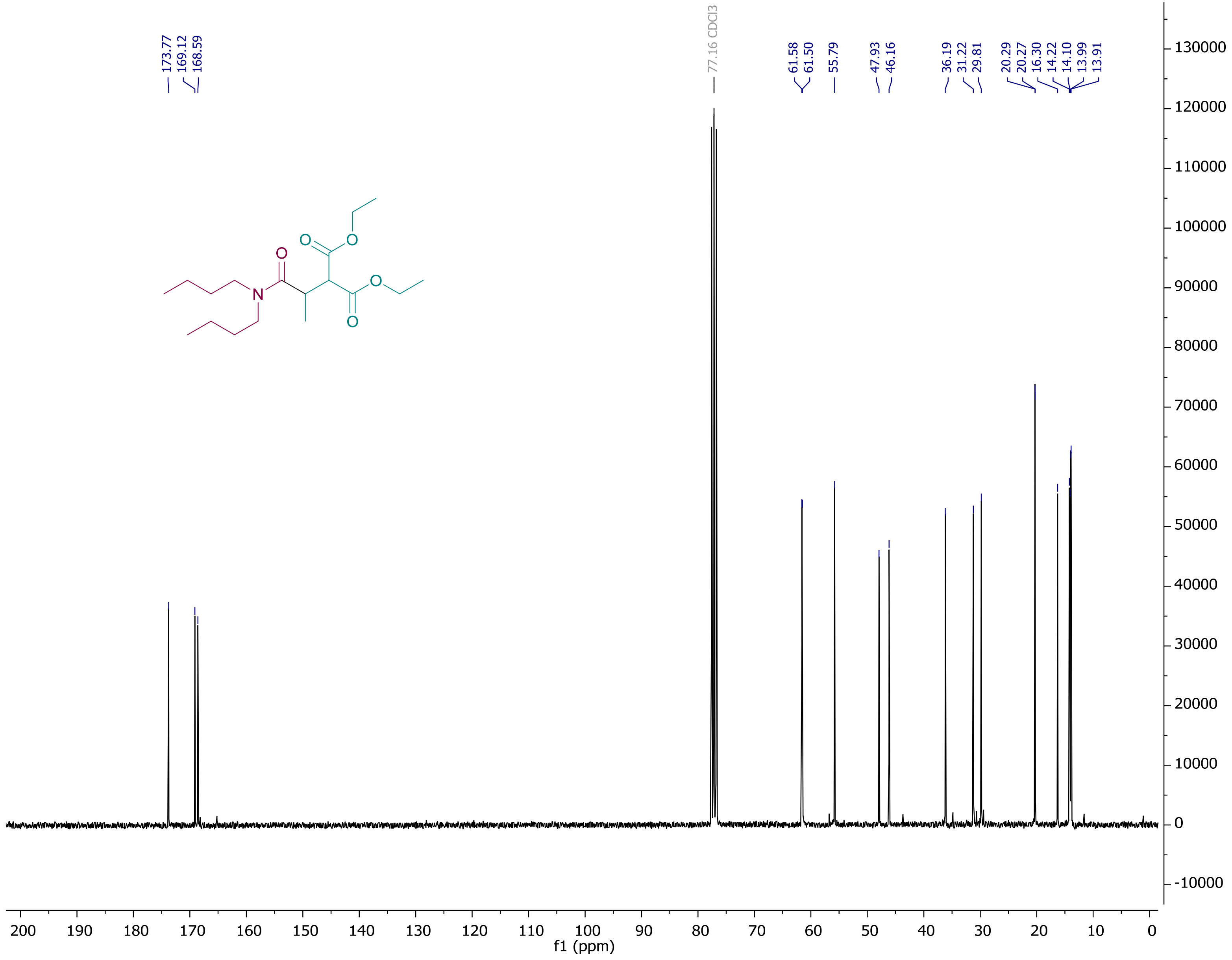

Diethyl 2-(1-(dihexylamino)-1-oxopropan-2-yl)malonate (9q)

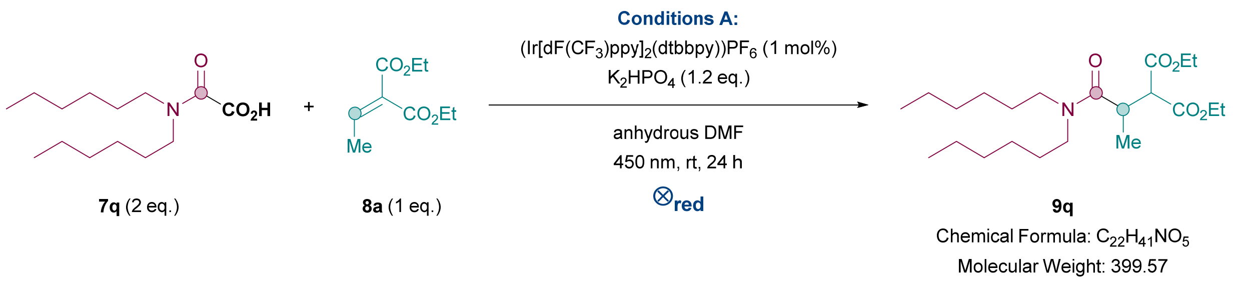

General procedure for Conditions A was followed. Diethyl 2-ethylidenemalonate **8a** (23.2 mg, 0.12 mmol, 1.0 eq.), *N,N*-(dihexyl)-oxoacetic acid **7q** (62.4 mg, 0.24 mmol, 2.0 eq.), K_2HPO_4 (26.3 mg, 0.15 mmol, 1.2 eq.), Ir[dF(CF_3)ppy](dtbppy)PF_6 (1.4 mg, 1 µmol, 1 mol%) in dry DMF (0.3 mL) was irradiated at 450 nm for 24 h. The crude was then purified twice *via* column flash chromatography eluting with 80:20 hexane:EtOAc. The solvent was then removed *in vacuo* to afford diethyl 2-(1-(dihexylamino)-1-oxopropan-2-yl)malonate **9q** as a colourless oil (26.6 mg, 0.067 mmol, 53%).

Characterisation:

^{1}H NMR (400 MHz, Chloroform-*d*): δ ppm 4.31 – 4.03 (m, 4H, 2 x OCH_2), 3.86 (d, *J* = 10.8 Hz, 1H C<u>H</u>(CO_2Et)$_2$), 3.41 – 3.11 (m, 5H, CHMe, 2 x NCH_2), 1.79 – 1.67 (m, 1H, m, 1H, C<u>H</u>H), 1.66 – 1.55 (m, 1H, CH<u>H</u>), 1.52 – 1.40 (m, 2H. CH_2), 1.37 – 1.24 (m, 15H, 6 x CH_2, CH_3), 1.21 (t, *J* = 7.2 Hz, 3H, CH_3), 1.12 (d, *J* = 7.0 Hz, 3H, CH_3), 0.90 (t, *J* = 6.7 Hz, 3H, CH_3), 0.86 (t, *J* = 6.9 Hz, 3H).**^{13}C NMR (101 MHz, Chloroform-*d*):** 173.7 (C), 169.1 (C), 168.6 (C), 61.6 (CH_2), 61.5 (CH_2), 55.8 (CH), 48.2 (CH_2), 46.4 (CH_2), 36.2 (CH), 31.7 (CH_2), 31.6 (CH_2), 29.1 (CH_2), 27.6 (CH_2), 26.8 (CH_2), 26.7 (CH_2), 22.74 (CH_2), 27.72 (CH_2), 16.3 (CH_3), 14.2 (CH_3), 14.14 (CH_3), 14.12 (CH_3 plus one overlapping CH_3). **IR:** ν_{max}/cm^{-1} 2957, 2929, 2873, 2858, 1750, 1732, 1640, 1464, 1446, 1429, 1368, 1349, 1299, 1276, 1243. **HRMS (ESI-TOF):** *m/z* $[M + H]^+$ calcd for $C_{22}H_{42}NO_5$, 400.30575; found, 400.3076. **TLC:** R_f = 0.18 (86:14 hexane:EtOAc).

Compound 9q – ^{1}H NMR (400 MHz, Chloroform-*d*):

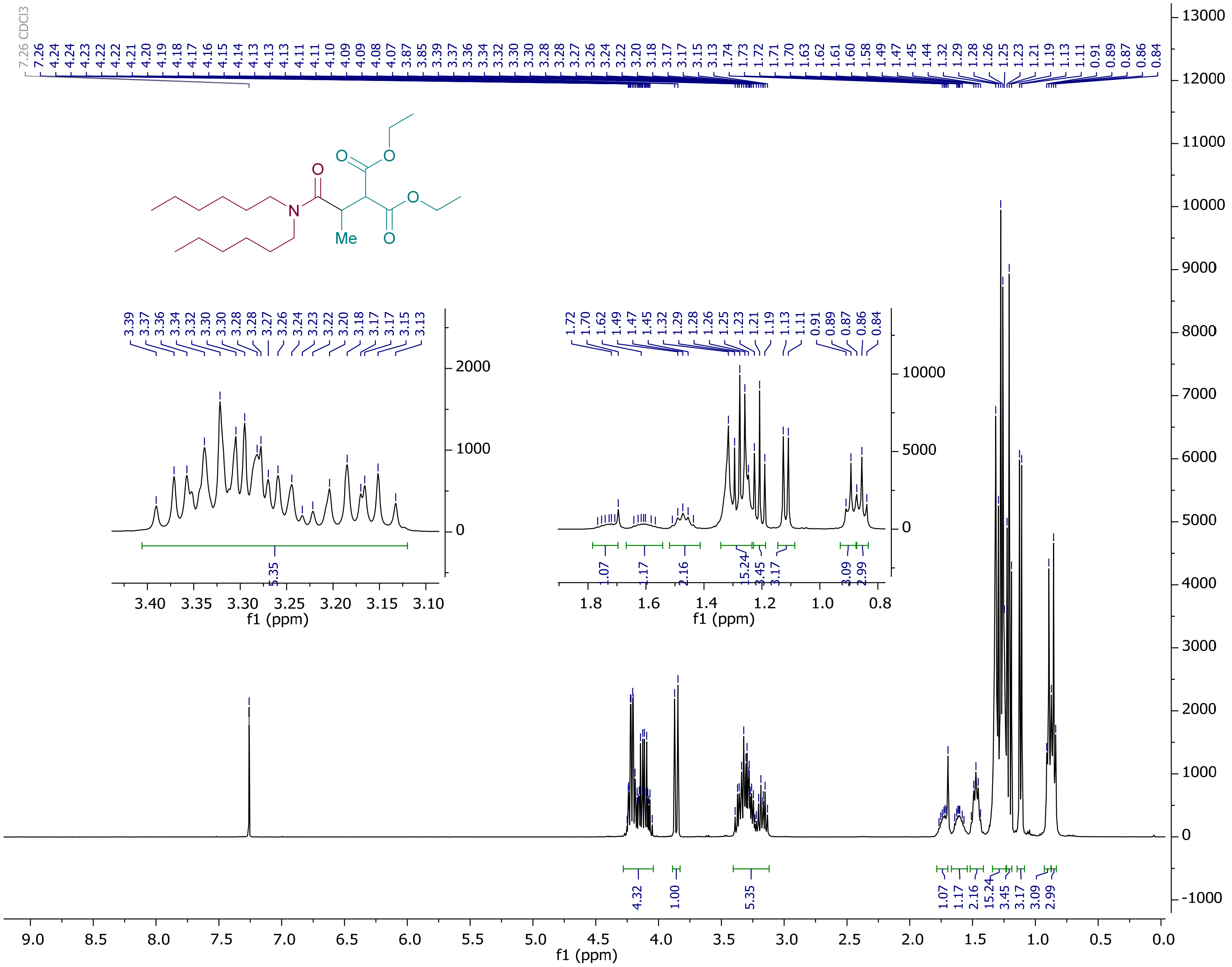

Compound 9q – ^{13}C NMR (101 MHz, Chloroform-*d*):

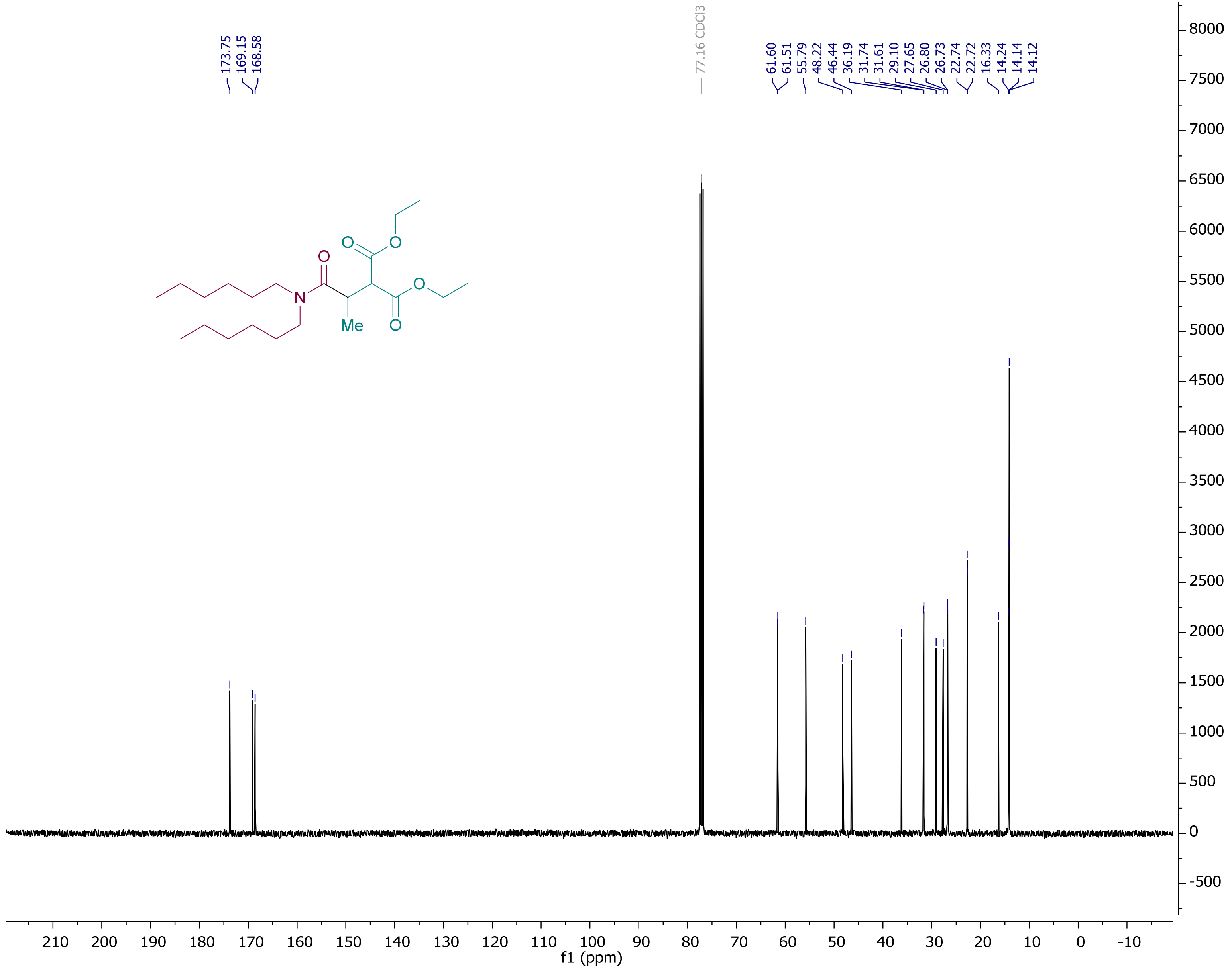

Diethyl 2-(1-(ethyl(propyl)amino)-1-oxopropan-2-yl)malonate (9r)

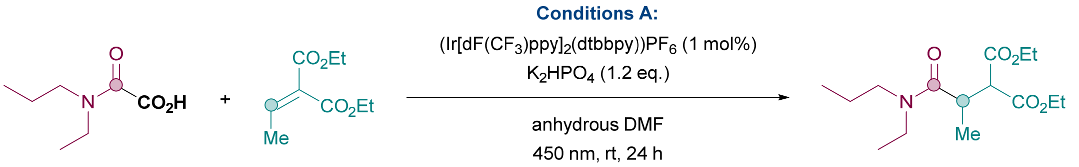

7r (2 eq.) **8a** (1 eq.) **9r**

Chemical Formula: $C_{15}H_{27}NO_5$
Molecular Weight: 301.38

General procedure for Conditions A was followed. Diethyl 2-ethylidenemalonate **8a** (44.7 mg, 0.24 mmol, 1.0 eq.), 2-(ethyl(propyl)amino)-2-oxoacetic acid **7r** (76.4 mg, 0.48 mmol, 2.0 eq.), (Ir[dF(CF3)ppy]2(dtbbpy))PF6 (2.7 mg, 2.4 µmol, 1 mol%), and K_2HPO_4 (50.3, 0.29 mmol, 1.2 eq.) in anhydrous DMF (0.6 mL) was irradiated with 450 nm for 23 h. The crude was then purified *via* column flash chromatography eluting with 70:30 hexane:EtOAc. The solvent was then removed *in vacuo* to afford as a mixture of rotamers diethyl 2-(1-(ethyl(isopropyl)amino)-1-oxopropan-2-yl)malonate **9r** as a colourless oil (38.9 mg, 0.061 mmol, 51%).

Characterisation:

^{1}H NMR (300 MHz, Chloroform-*d*): (mixture of rotamers) δ ppm 4.28 – 4.04 (m, 4H, 2 x OCH_2, 2 x OCH'$_2$), 3.86 (d, *J* = 10.8, Hz, 0.5H, C<u>H</u>($CO_2Et)_2$), 3.85 (d, *J* = 10.7 Hz, 0.5H, C<u>H</u>'($CO_2Et)_2$) 3.48 – 3.20 (m, 4.5H, C<u>H</u>Me, C<u>H'</u>Me, 3 x CH_2, C<u>H'</u>H'), 3.20 – 3.05 (m, 0.5H, CH'<u>H'</u>), 1.96 – 1.59 (m, 1H, CH_2), 1.51 (sextet, *J* = 7.5 Hz, 1H, CH'$_2$), 1.32 – 1.23 (m, 4.5H, 3 x CH_3), 1.20 (t, *J* = 7.1 Hz, 3H, 2 x CH'$_3$), 1.12 (d, *J* = 7.0 Hz, 1.5H, CHC<u>H</u>$_3$), 1.11 (d, *J* = 7.0 Hz, 1.5H, CHC<u>H'</u>$_3$), 1.06 (t, *J* = 7.1 Hz, 1.5H, CH'$_3$), 0.94 (t, *J* = 7.4 Hz, 1.5H, CH_3), 0.84 (t, *J* = 7.4 Hz, 1.5H, CH'$_3$). **^{13}C NMR (101 MHz, Chloroform-*d*):** (mixture of rotamers) δ ppm 173.8 (C), 173.6 (C), 169.10 (C), 169.09 (C), 168.61 (C), 168.60 (C), 61.6 (CH_2 plus one overlapping CH_2), 61.5 (CH_2 plus one overlapping CH_2), 55.8 (CH), 55.7 (CH), 49.5 (CH_2), 47.4 (CH_2), 42.5 (CH_2), 41.2 (CH_2), 36.2 (CH), 36.1 (CH), 22.5 (CH_2), 21.0 (CH_2), 16.3 (CH_3), 16.2 (CH_3), 14.3 (CH_3), 14.2 (CH_3 plus one overlapping CH_3), 14.1 (CH_3 plus one overlapping CH_3), 12.8 (CH_3), 11.5 (CH_3), 11.4 (CH_3). **IR:** ν_{max}/cm^{-1} 2970, 2936, 2876, 1748, 1729, 1637, 1541, 1464, 1442, 1368, 1298, 1277, 1245, 1192. **HRMS (ESI-TOF):** *m/z* $[M + H]^+$ calcd for $C_{15}H_{28}NO_5$, 302.19620; found, 302.1963. **TLC:** R_f = 0.23 (70:30 hexane:EtOAc).

Compound 9r – ^{1}H NMR (300 MHz, Chloroform-*d*): (mixture of rotamers)

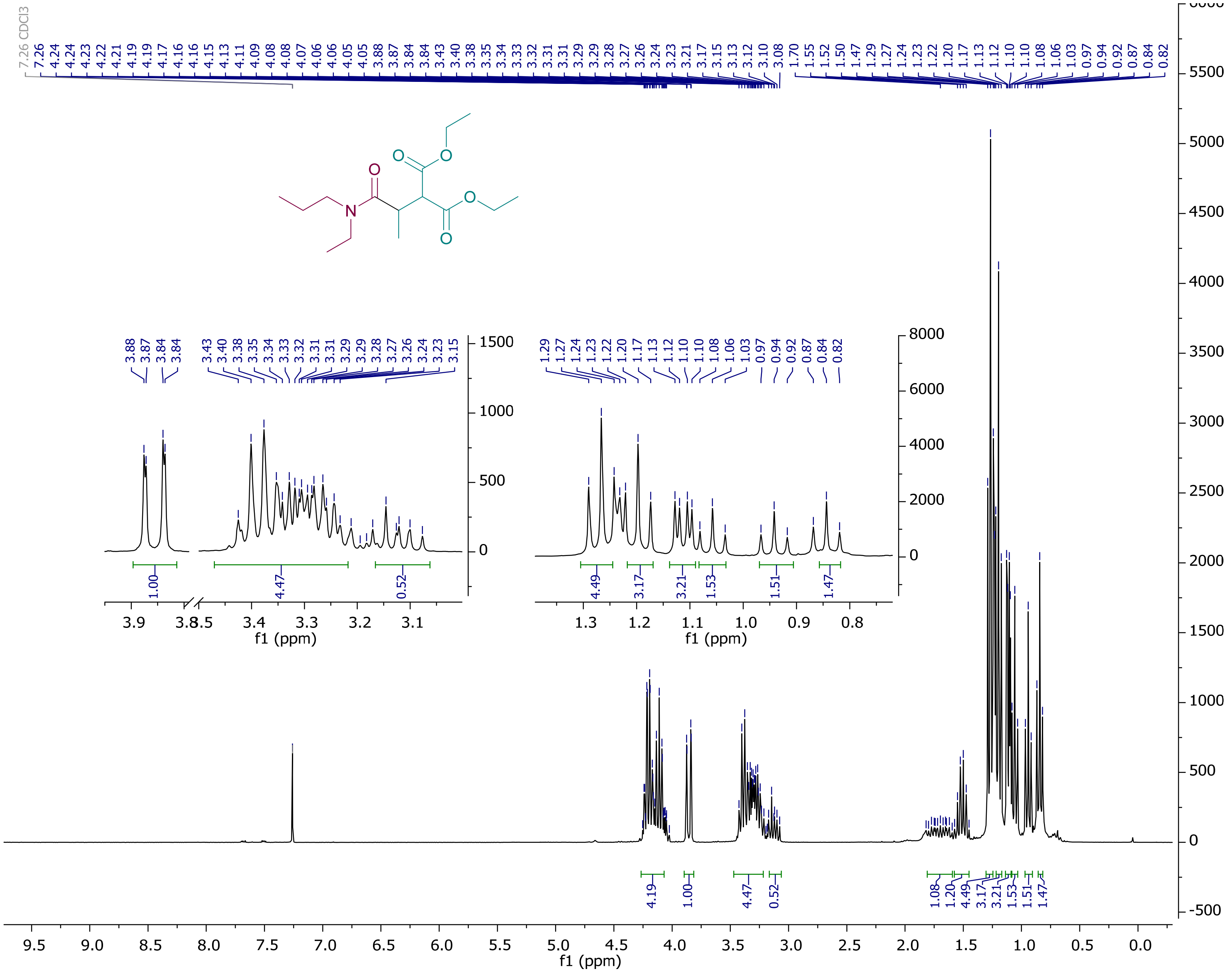

Compound 9r – ^{13}C NMR (101 MHz, Chloroform-*d*): (mixture of rotamers)

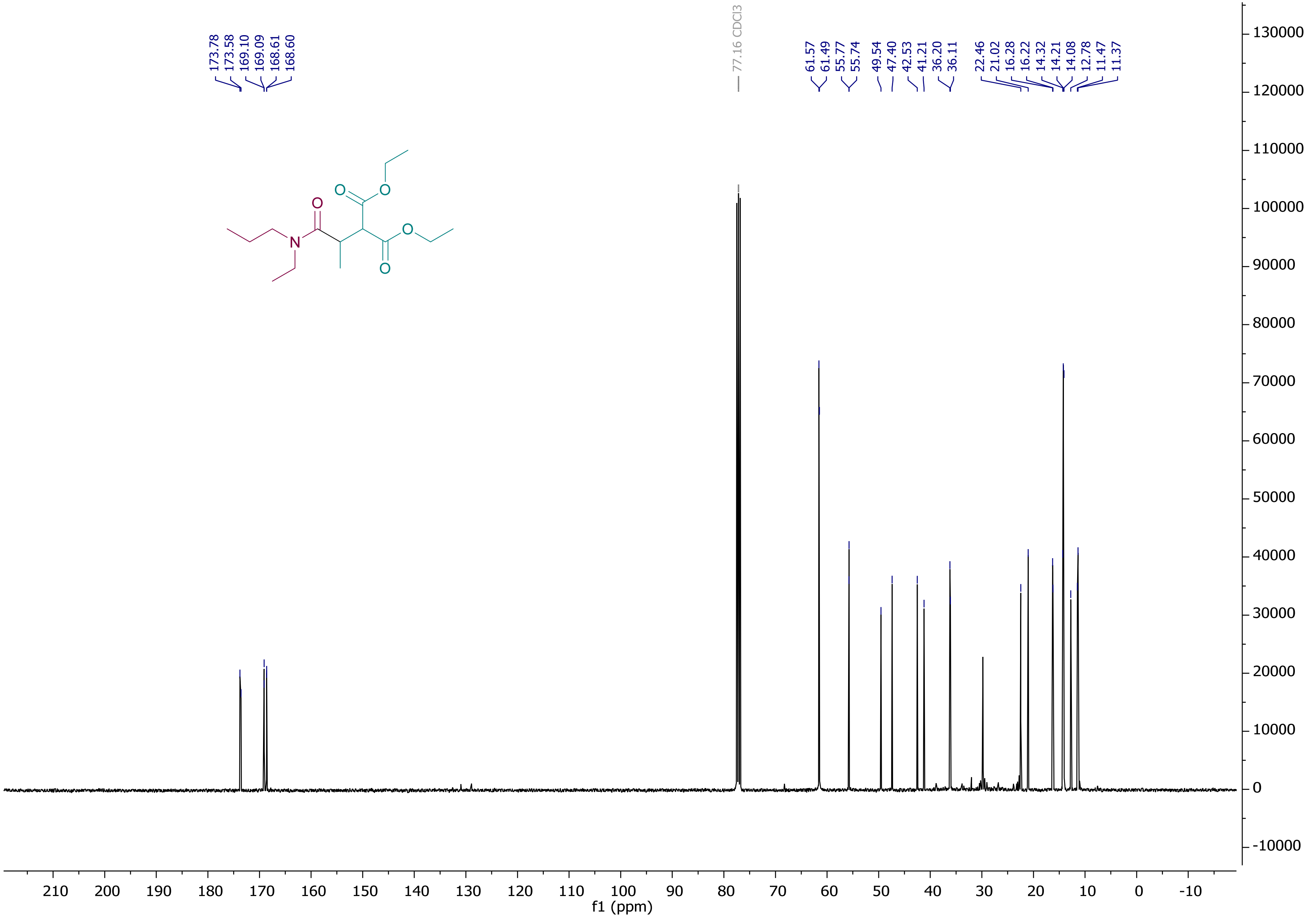

Diethyl 2-(1-(di-(*i*-propyl)amino)-1-oxopropan-2-yl)malonate (9s)

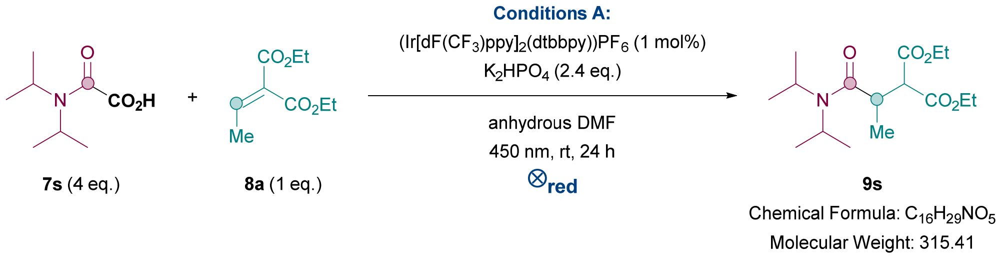

General procedure for Conditions A was followed. Diethyl 2-ethylidenemalonate **8a** (22.4 mg, 0.12 mmol, 1.0 eq.), *N*,*N*-(di-(*i*-propyl))-oxoacetic acid **7s** (83.4 mg, 0.48 mmol, 4.0 eq.), K_2HPO_4 (50.2 mg, 0.29 mmol, 2.4 eq.), Ir[dF(CF_3)ppy](dtbppy)PF_6 (1.3 mg, 1 µmol, 1 mol%) in dry DMF was irradiated for 24 h. The crude was then purified *via* column flash chromatography eluting with 80:20 hexane:EtOAc. The solvent was then removed *in vacuo* to afford diethyl 2-(1-(di-(*i*-propyl)amino)-1-oxopropan-2-yl)malonate **9s** as a pale-green oil (20.9 mg, 0.066 mmol, 55%).

Characterisation:

^{1}H NMR (300 MHz, Chloroform-*d*): δ ppm 4.25 – 4.09 (m, 5H, 2 x OCH_2, CH), 3.85 (d, J = 10.8 Hz, 1H, C<u>H</u>(CO_2Et)$_2$), 3.45 – 3.26 (m, 2H, 2 x CH), 1.35 (d, J = 3.8 Hz, 3H, CH_3), 1.33 (d, J = 3.8 Hz, 3H, CH_3), 1.29-1.19 (m, 12H, 4 x CH_3), 1.09 (d, J = 7.1 Hz, 3H, CH_3). **^{13}C NMR (75 MHz, Chloroform-*d*):** δ ppm 172.9 (C), 169.3 (C), 168.7 (C), 61.5 (CH_2), 61.4 (CH_2), 55.8 (CH), 48.8 (CH), 45.9 (CH), 37.5 (CH), 21.0 (CH_3), 20.9 (CH_3), 20.7 (CH_3), 20.4 (CH_3), 15.9 (CH_3), 14.3(CH_3), 14.1 (CH_3). **IR:** ν_{max}/cm^{-1} 2970, 2937, 2875, 1748, 1729, 1636, 1464, 1443, 1368, 1335, 1303, 1276. **HRMS (ESI-TOF):** *m*/*z* [M + H]$^+$ calcd for $C_{16}H_{30}NO_5$, 316.21185; found, 316.2124 . **TLC:** R_f = 0.35 (75:25 hexane:EtOAc).

Compound 9s – ^{1}H NMR (300 MHz, Chloroform-*d*):

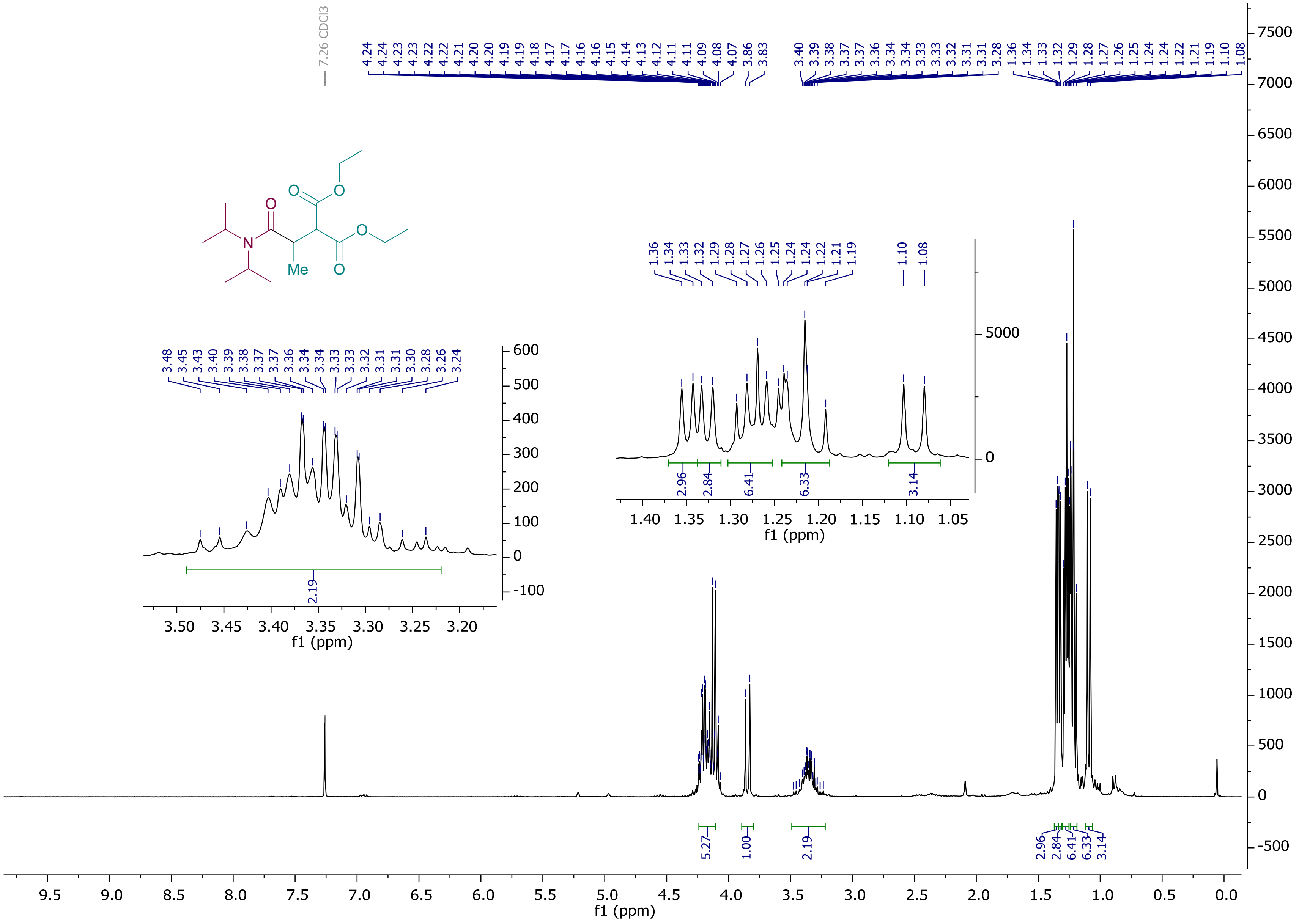

Compound 9s – ^{13}C NMR (75 MHz, Chloroform-*d*):

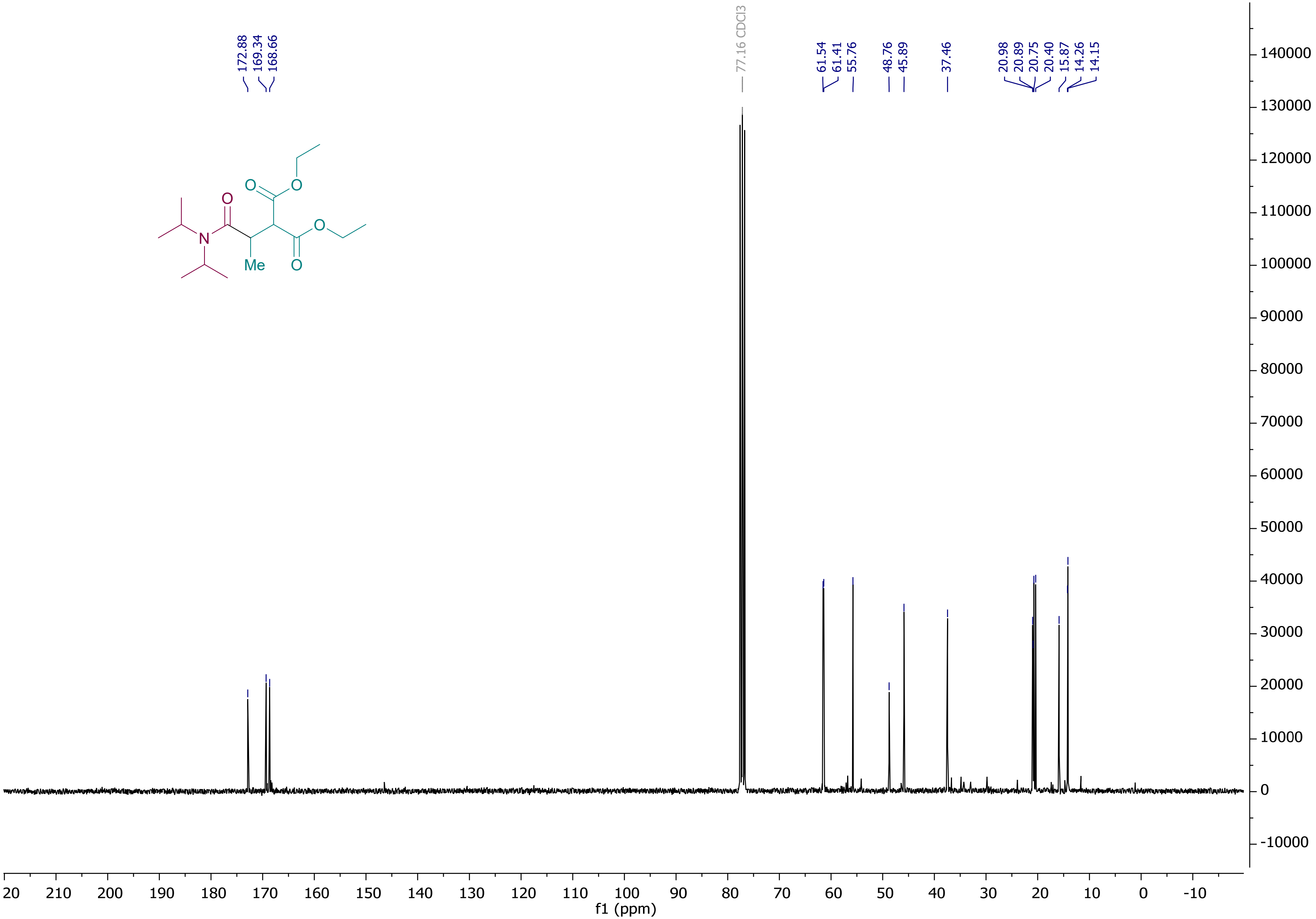

Diethyl 2-(1-(ethyl(*i*-propyl)amino)-1-oxopropan-2-yl)malonate (9t)

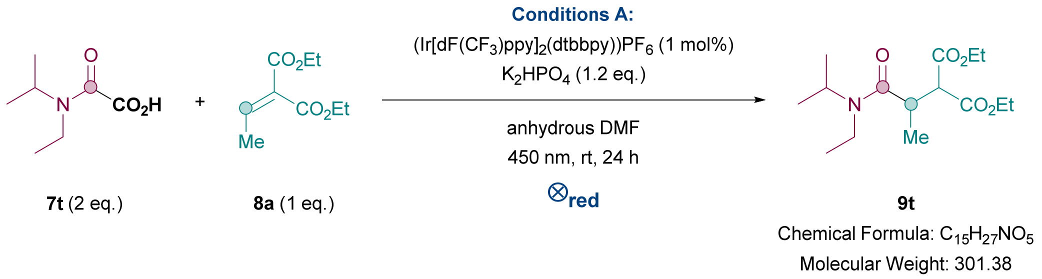

General procedure for Conditions A was followed. Diethyl 2-ethylidenemalonate **8a** (37.4 mg, 0.20 mmol, 1.0 eq.), 2-(ethyl(isopropyl)amino)-2-oxoacetic acid **7t** (63.8 mg, 0.40 mmol, 2.0 eq.), (Ir[dF(CF_3)ppy]$_2$(dtbbpy))PF_6 (2.1 mg, 2 µmol, 1 mol%), and K_2HPO_4 (41.7, 0.24 mmol, 1.2 eq.) in anhydrous DMF (0.5 mL) was irradiated with 450 nm for 24 h. The crude was then purified *via* column flash chromatography eluting with 70:30 hexane:EtOAc. The solvent was then removed *in vacuo* to afford as a 50:50 mixture of rotamers diethyl 2-(1-(ethyl(*i*-propyl)amino)-1-oxopropan-2-yl)malonate **9t** as a yellow oil (24.6 mg, 0.049 mmol, 41%).

Characterisation:

^{1}H NMR (300 MHz, Chloroform-*d*): (mixture of rotamers) 4.62 (hept, J = 6.8 Hz, 0.5H, CH(*i*-Pr)$_2$ one rotamer), 4.27 – 4.02 (m, 4.5H, CH(*i*-Pr)$_2$ second rotamer and 2 x OCH_2), 3.89 (d, J = 10.8 Hz, 0.5 H, C<u>H</u>(CO_2Et)$_2$ one rotamer), 3.88 (d, J = 10.8 Hz, 0.5 H, C<u>H</u>(CO_2Et)$_2$ one rotamer), 3.52 – 3.33 (m, 2H, C<u>H</u>H, CH), 3.33 – 3.08 (m, 4H, CH<u>H</u>, CH, CH_2), 1.36 – 1.17 (m, 12H, 4 x CH_3), 1.16 – 1.05 (m, 6H, 2 x CH_3). **^{13}C NMR (101 MHz, Chloroform-*d*):** (mixture of rotamers) δ ppm 174.1 (C), 173.1 (C), 169.2 (C), 169.1 (C), 168.6 (C plus one overlapping C), 61.6 (CH_2 plus one overlapping CH_2), 61.5 (CH_2), 61.4 (CH_2), 55.9 (CH), 55.7 (CH), 48.3 (CH), 45.6 (CH), 37.4 (CH_2), 36.7 (CH), 36.4 (CH), 35.6 (CH_2), 21.4 (CH_3), 20.40 (CH_3), 20.39 (CH_3), 16.61 (CH_3), 16.57 (CH_3), 16.0 (CH_3), 14.7 (CH_3), 14.2 (CH_3), 14.10 (CH_3), 14.09 (CH_3) plus 2 overlapping CH_3s **IR:** ν_{max}/cm^{-1} 2977, 2938, 2876, 1748, 1729, 1634, 1464, 1447, 1429, 1368, 1301, 1277, 1244, 1216. **HRMS (ESI-TOF):** *m/z* $[M + H]^+$ calcd for $C_{15}H_{28}NO_5$, 302.19620; found, 302.1971. **TLC:** R_f = 0.26 (70:30 hexane:EtOAc).

Compound 9t – ^{1}H NMR (300 MHz, Chloroform-*d*): (mixture of rotamers)

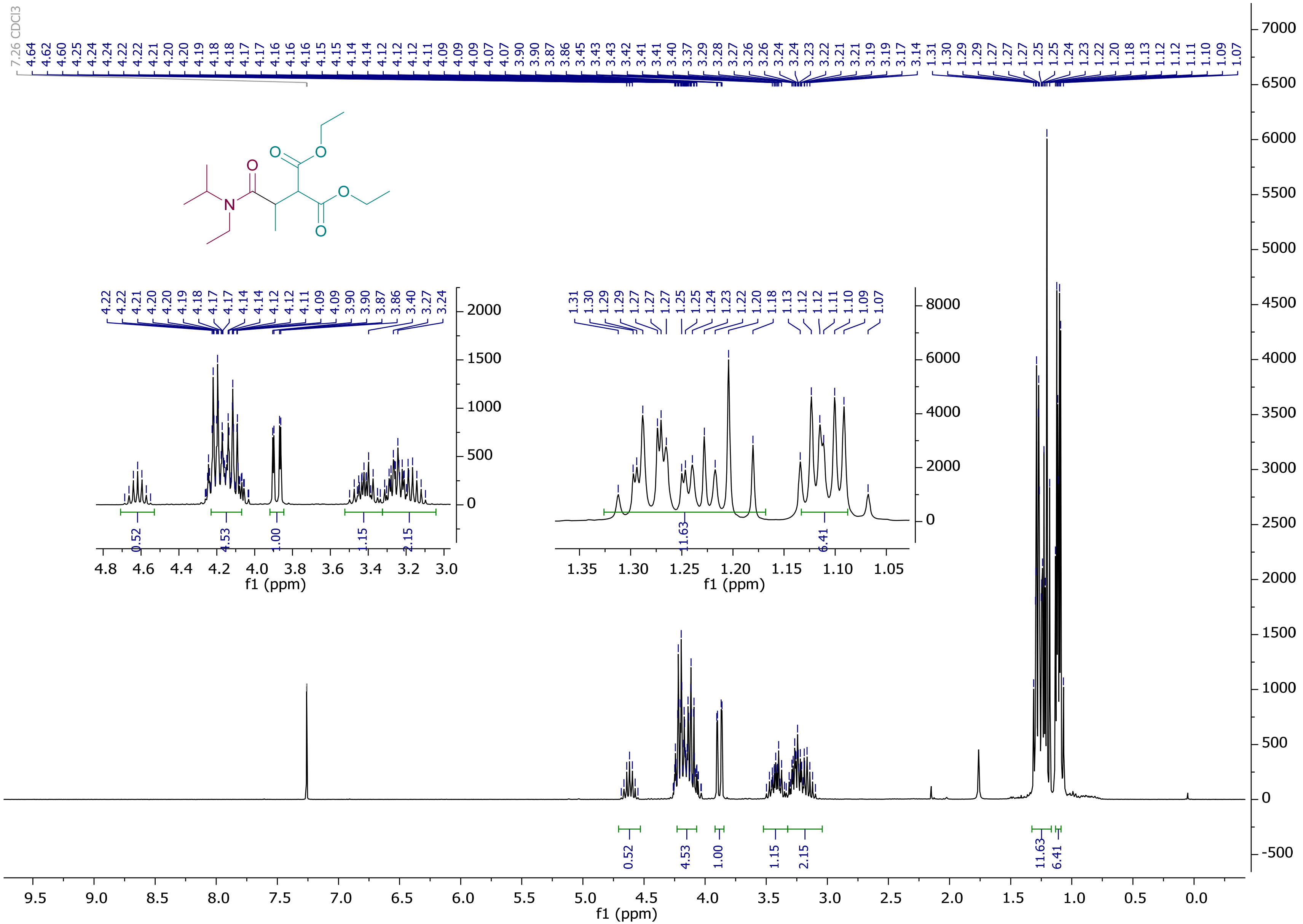

Compound 9t – ^{13}C NMR (101 MHz, Chloroform-*d*): (mixture of rotamers)

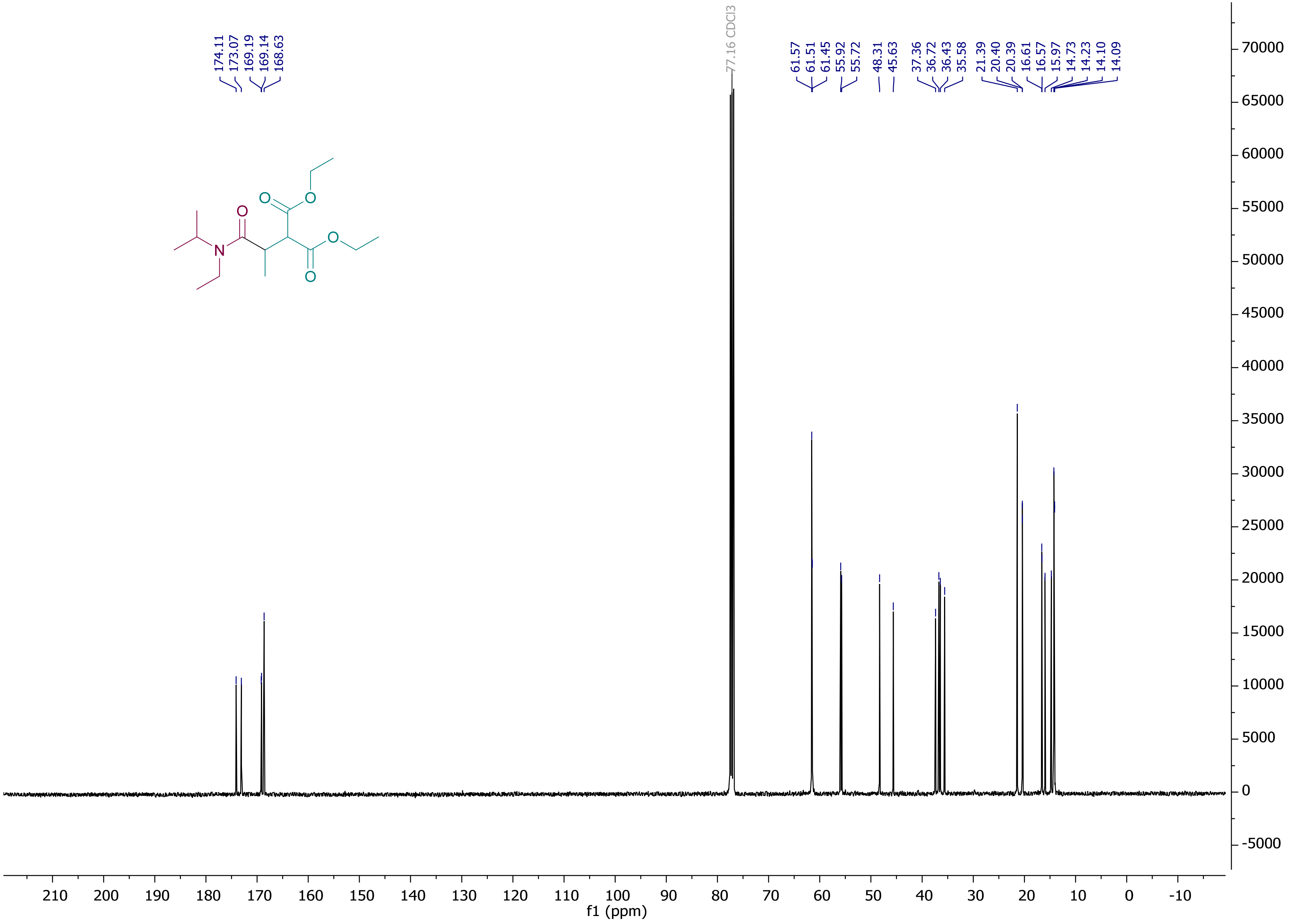

Diethyl 2-(1-oxo-1-(piperidin-1-yl)propan-2-yl)malonate (9u)

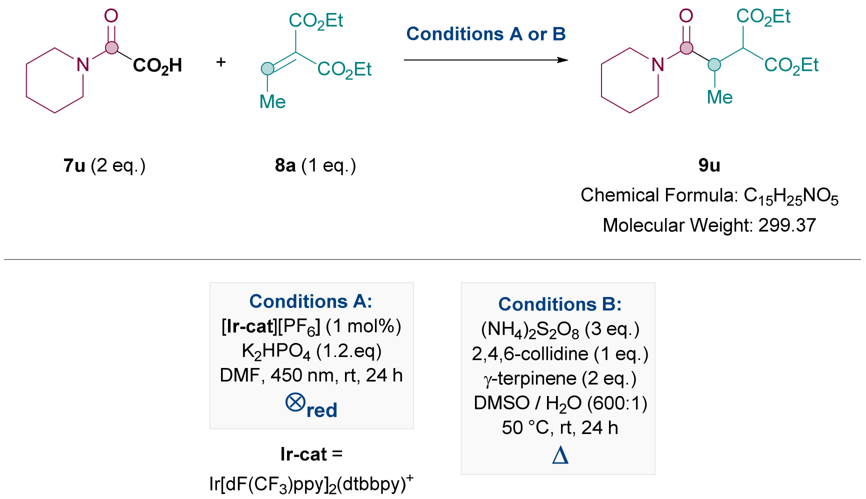

Using Conditions A: General procedure for Conditions A was followed. Diethyl 2-ethylidenemalonate **8a** (22.4 mg, 0.12 mmol, 1.0 eq.), 2-oxo-2-(piperidin-1-yl)acetic acid **7u** (38.0 mg, 0.24 mmol, 2.0 eq.), K_2HPO_4 (25.6 mg, 0.24 mmol, 1.2 eq.), Ir[dF(CF_3)ppy](dtbppy)PF_6 (1.4 mg, 1 µmol, 1 mol%) in dry DMF (0.3 mL) was irradiated with 450 nm for 24 h. The crude was then purified *via* column flash chromatography eluting with 75:25 hexane:EtOAc. The solvent was then removed *in vacuo* to afford diethyl 2-(1-oxo-1-(phenylamino)propan-2-yl)malonate **9u** as an off-white solid (12.4 mg, 0.042 mmol, 35%).

Using Conditions B: General procedure for Conditions B was followed. Diethyl 2-ethylidenemalonate **8a** (22.3 mg, 0.12 mmol, 1.0 eq.), 2-oxo-2-(piperidin-1-yl)acetic acid **7u** (37.9 mg, 0.24 mmol, 2.0 eq.), $(NH_4)_2S_2O_8$ (82.6 mg, 0.36 mmol, 3.0 eq.), γ-terpinene (38.5 µL, 0.24 mmol, 2.0 eq.), and 2,4,6-collidine (15.9 µL, 0.12 mmol, 1.0 eq.) in DMSO:H_2O (600:1) (0.4 mL) was heated at 40 °C for 48 h followed by work-up X. The crude was then purified *via* column flash chromatography eluting with 75:25 hexane:EtOAc. The solvent was then removed *in vacuo* to afford diethyl 2-(1-oxo-1-(piperidin-1-yl)propan-2-yl)malonate **9u** as a pale green oil (11.1 mg, 0.037 mmol, 31%).

Characterisation:

^{1}H NMR (400 MHz, Chloroform-*d*): δ ppm 4.29 – 4.05 (m, 4H, 2 x OCH_2), 3.89 (d, J = 10.8 Hz, 1H, C<u>H</u>(CO_2Et)$_2$), 3.71 – 3.52 (m, 2H, C<u>H</u>H, C<u>H</u>H), 3.50 – 3.35 (m, 3H, CH<u>H</u>, CH<u>H</u>, CHMe), 1.86 – 1.44 (m, 6H, 3 x CH_2), 1.28 (t, J = 7.2 Hz, 3H, CH_3), 1.22 (t, J = 7.1 Hz, 3H,

CH_3), 1.11 (d, *J* = 7.1 Hz, 3H, CHC<u>H</u>$_3$). **^{13}C NMR (101 MHz, Chloroform-*d*):** δ ppm 172.5 (C), 169.2 (C), 168.7 (C), 61.63 (CH_2), 61.56 (CH_2), 55.6 (CH), 47.0 (CH_2), 43.3 (CH_2), 35.9 (CH), 26.6 (CH_2), 25.7 (CH_2), 24.8 (CH_2), 15.7 (CH_3), 14.3 (CH_3), 14.1 (CH_3). **IR:** ν_{max}/cm^{-1} 2979, 2937, 2857, 1747, 1729, 1634, 1464. 1443, 1368, 1276, 1246, 1184. **HRMS (ESI-TOF):** *m*/*z* [M + H]$^+$ calcd for $C_{15}H_{26}NO_5$, 300.18055; found, 300.1806. **TLC:** R_f = 0.36 (75:25% hexane:EtOAc).

Compound 9u – ^{1}H NMR (400 MHz, Chloroform-*d*):

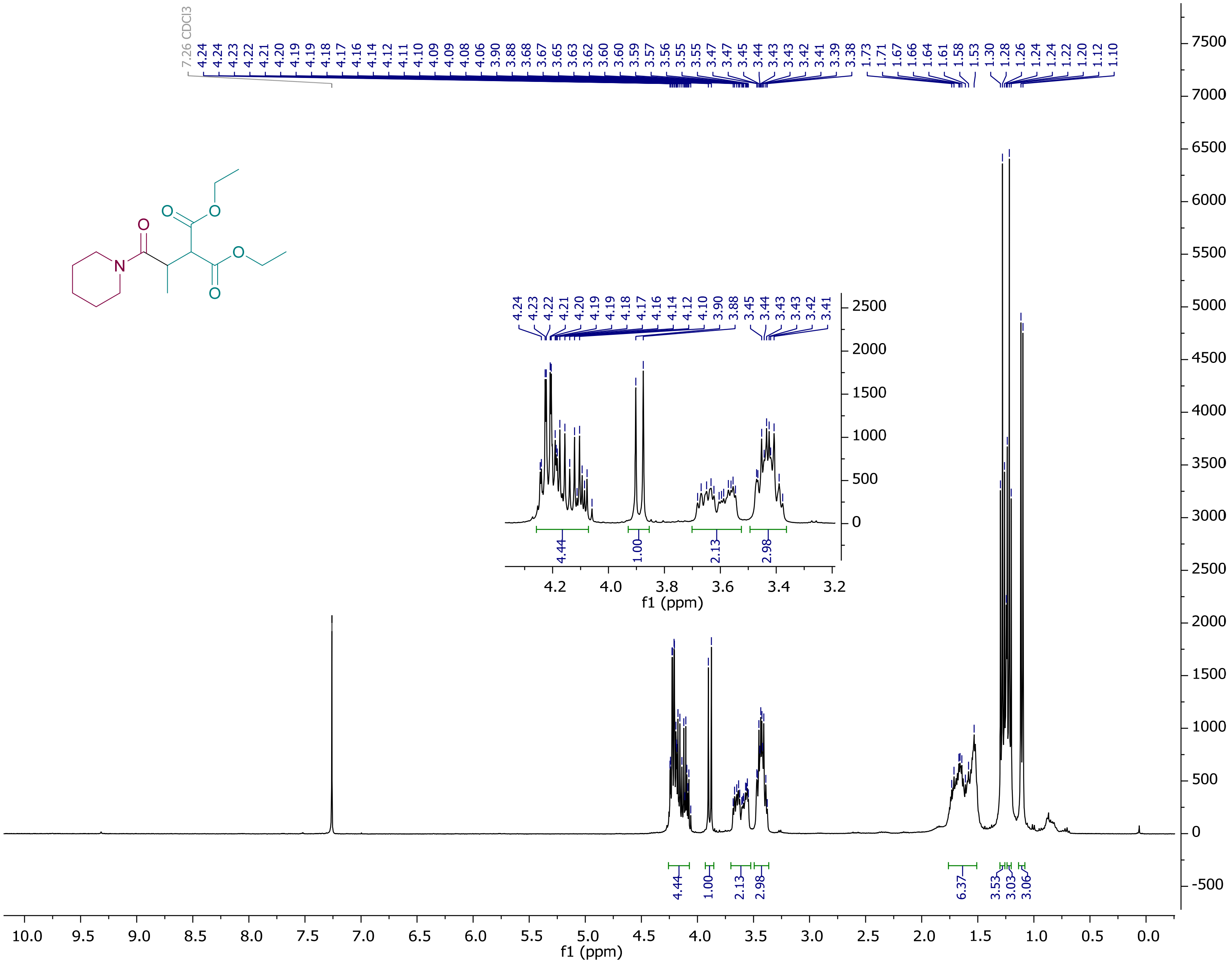

Compound 9u – ^{13}C NMR (101 MHz, Chloroform-*d*):

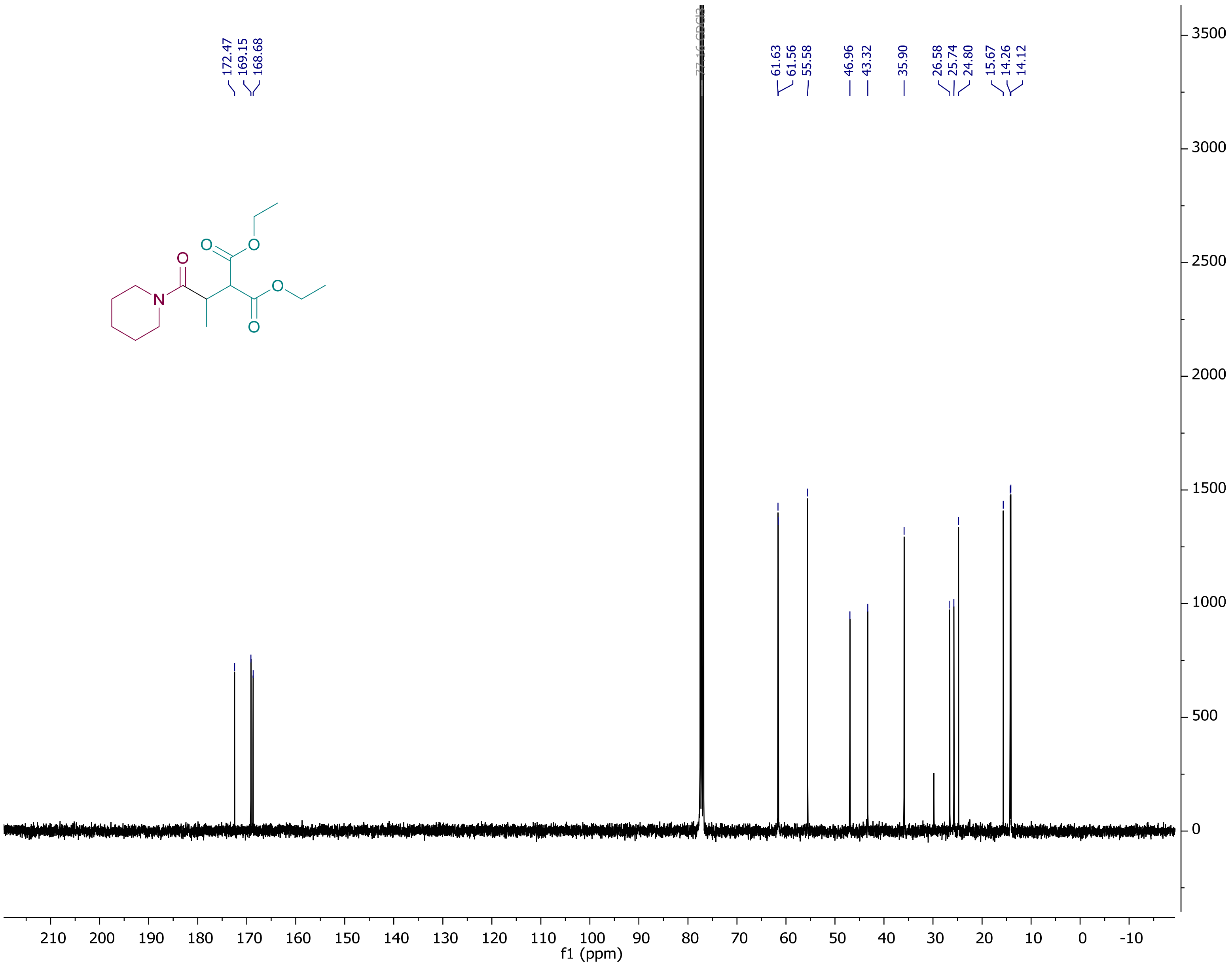

Diethyl 2-(2-oxo-1-phenyl-2-(phenylamino)ethyl)malonate (9v)

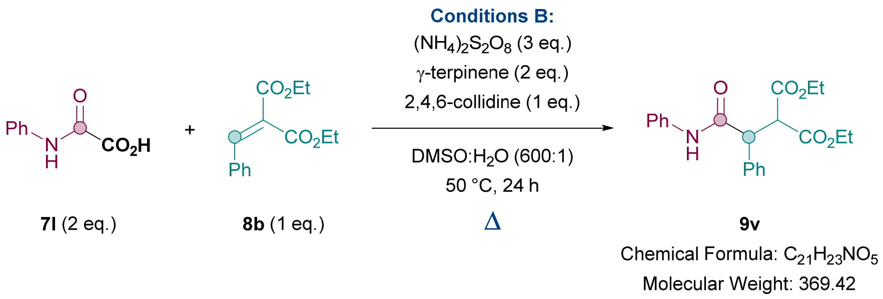

General procedure for Conditions B was followed. Diethyl 2-benzylidenemalonate **8b** (29.8 mg, 0.12 mmol, 1.0 eq.), *N*-(phenyl)-oxoacetic acid **7l** (40.0 mg, 0.24 mmol, 2.0 eq.), $(NH_4)_2S_2O_8$ (82.7 mg, 0.36 mmol, 3.0 eq.), γ-terpinene (38.5 µL, 0.24 mmol, 2 eq.), and 2,4,6-collidine (15.9 µL, 0.12 mmol, 1.0 eq.) in DMSO:H_2O (600:1) (0.4 mL) was heated at 50 °C for 24 h followed by work-up X. The crude was then purified *via* column flash chromatography eluting with 70:30 hexane:EtOAc. The solvent was then removed *in vacuo* to afford diethyl 2-(2-oxo-1-phenyl-2-(phenylamino)ethyl)malonate **9v** as a yellow/green oil (24.7 mg, 0.067 mmol, 56%).

Characterisation:

^{1}H NMR (300 MHz, Chloroform-*d*): δ ppm 7.62 (br s, 1H, NH), 7.48 – 7.35 (m, 4H, ArH), 7.35 – 7.27 (m, 3H, ArH), 7.20 (t, *J* = 7.9 Hz, 2H, ArH), 7.01 (t, *J* = 7.4 Hz, 1H, ArH), 4.39 (d, *J* = 11.4 Hz, 1H, CH), 4.34 (d, *J* = 11.4 Hz, 1H, CH), 4.29 – 4.16 (m, 2H, OCH_2), 3.93 (q, *J* = 7.1 Hz, 2H, OCH_2), 1.26 (t, *J* = 7.1 Hz, 3H, CH_3), 0.94 (t, *J* = 7.1 Hz, 3H, CH_3). **^{13}C NMR (75 MHz, Chloroform-*d*):** δ ppm 169.4 (C), 168.6 (C), 167.8 (C), 137.9 (C), 135.6 (C), 129.0 (CH), 128.9 (CH), 128.7 (CH), 128.4 (CH), 124.4 (CH), 120.0 (CH), 62.2 (CH_2), 61.6 (CH_2), 55.5 (CH), 53.2 (C), 14.1 (CH_3), 13.8 (CH_3). **IR:** ν_{max}/cm^{-1} 3368, 2983, 1746, 1715, 1683, 1661, 1600, 1541, 1496, 1442, 1367, 1314, 1386, 1252, 1209, 1142. **HRMS (ESI-TOF):** *m*/*z* $[M + H]^+$ calcd for $C_{21}H_{24}NO_5$, 370.16490; found, 370.1658. **TLC:** R_f = 0.27 (70:30 hexane:EtOAc).

Compound 9v – ^{1}H NMR (300 MHz, Chloroform-*d*):

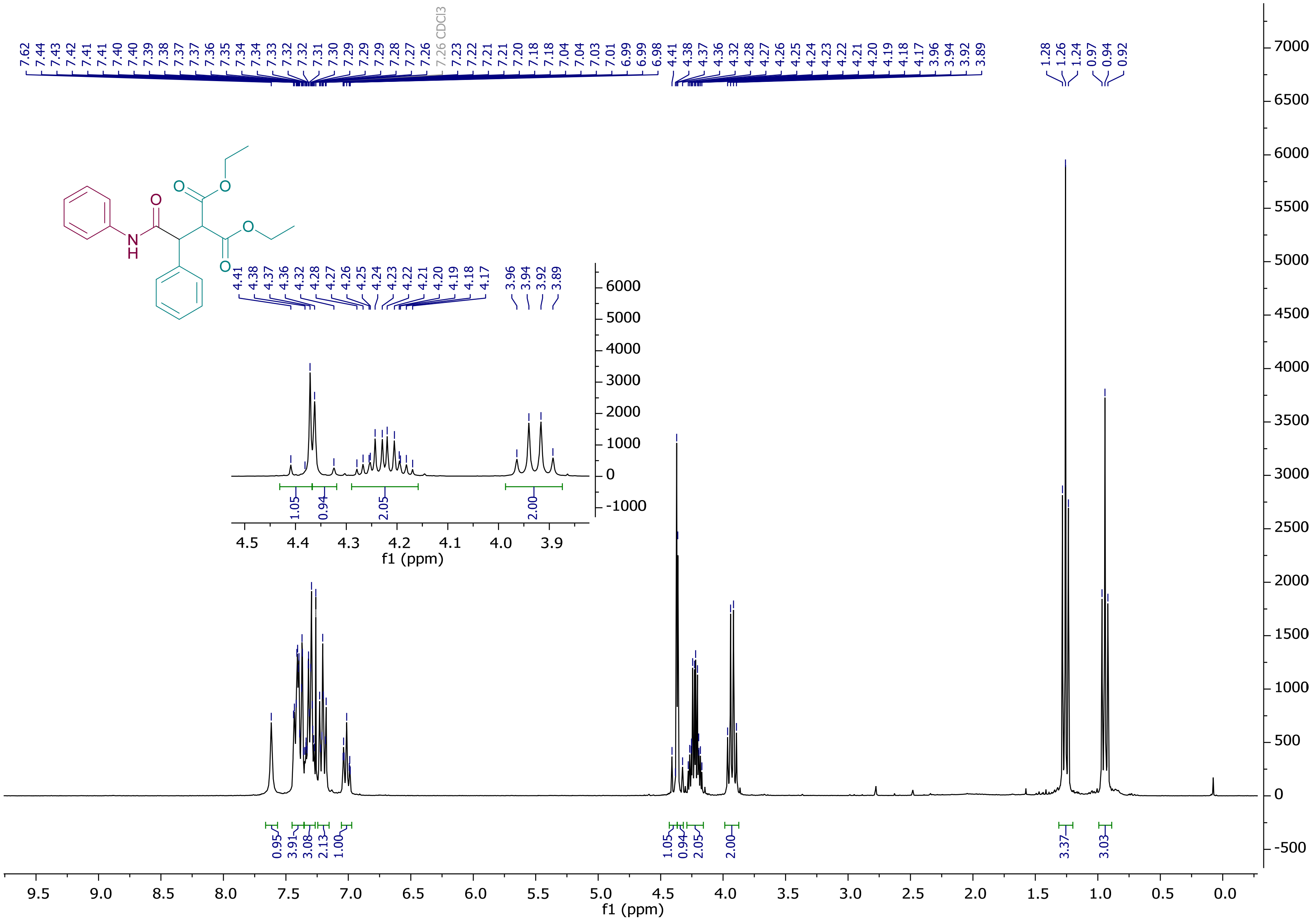

Compound 9v – ^{13}C NMR (75 MHz, Chloroform-*d*):

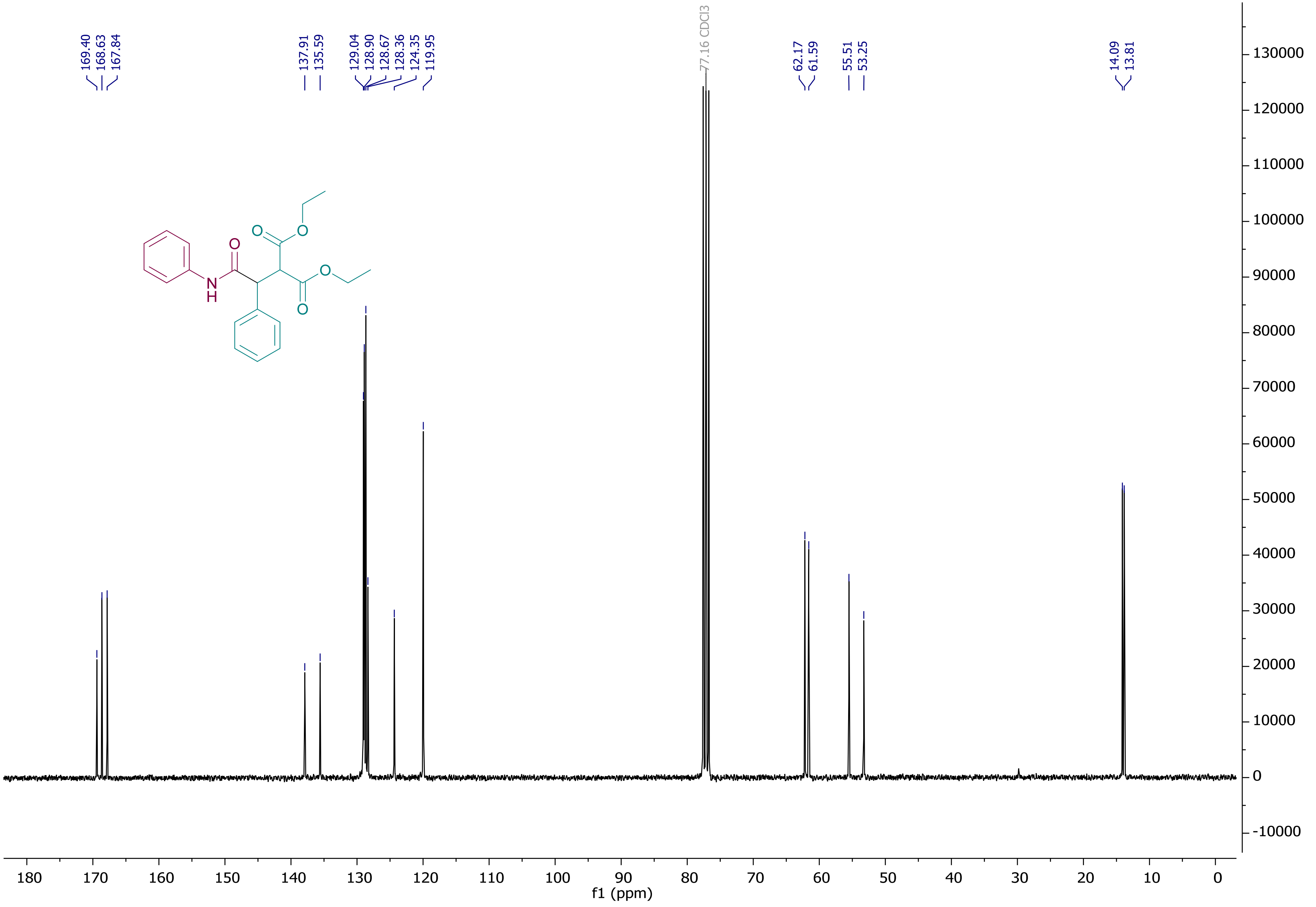

Ethyl 2-cyano-4-oxo-3-phenyl-4-(phenylamino)butanoate (9w)

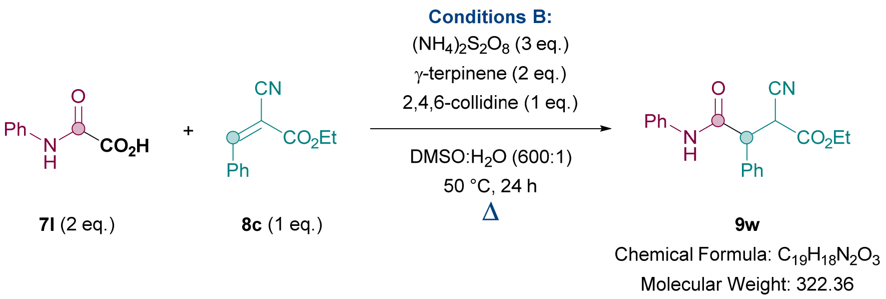

General procedure for Conditions B was followed. Ethyl 2-cyano-3-phenylacrylate **8c** (24.3 mg, 0.12 mmol, 1.0 eq.), *N*-(phenyl)-oxoacetic acid **7l** (39.7 mg, 0.24 mmol, 2.0 eq.), $(NH_4)_2S_2O_8$ (82.2 mg, 0.36 mmol, 3.0 eq.), γ-terpinene (38.5 μL, 0.24 mmol, 2 eq.), and 2,4,6-collidine (15.9 μL, 0.12 mmol, 1.0 eq.) in DMSO:H_2O (600:1) (0.4 mL) was heated at 50 ºC for 24 h followed by work-up X. The crude was then purified twice *via* column flash chromatography eluting with 70:30 hexane:EtOAc. The solvent was then removed *in vacuo* to a 70:30 diastereomeric mixture of ethyl 2-cyano-4-oxo-3-phenyl-4-(phenylamino)butanoate **9w** as a pale red oil (22.0 mg, 0.068 mmol, 57%)

Characterisation:

^{1}H NMR (300 MHz, Chloroform-*d*): (70:30 mixture of diastereomers) δ ppm 7.52 – 7.35 (m, 6.4H, ArH), 7.34 – 7.23 (m, 3.6H, ArH, NH major + minor), 7.09 (t, *J* = 7.3 Hz, 1H, ArH), 4.52 (d, *J* = 9.6 Hz, 0.7H, CHPh major), 4.44 (d, *J* = 6.7 Hz, 0.3H, CHPh minor), 4.33 – 4.14 (m, 2.7H, OCH_2 major + minor, C<u>H</u>($CO_2Et)_2$ major), 4.00 (d, *J* = 6.7 Hz, 0.3H, C<u>H</u>($CO_2Et)_2$ minor), 1.29 (t, *J* = 7.2 Hz, 2.1H, CH_3 major), 1.21 (t, *J* = 7.1 Hz, 0.9H, CH_3 minor). **^{13}C NMR (75 MHz, Chloroform-*d*):** (70:30 mixture of diastereomers) δ ppm 167.8 (C), 167.3 (C), 165.2 (C), 165.1 (C), 137.3 (C), 137.2 (C), 134.2 (C), 134.0 (C), 129.74 (CH), 129.70 (CH), 129.4 (CH plus one overlapping CH), 129.1 (CH), 129.0 (CH), 128.7 (CH plus one overlapping CH), 125.0 (CH plus one overlapping CH), 120.2 (CH plus one overlapping CH), 115.6 (C), 115.3 (C), 63.4 (CH_2), 63.3 (CH_2), 53.2 (CH), 52.7 (CH), 41.7 (CH), 40.8 (CH), 14.0 (CH_3), 13.9 (CH_3). **IR:** ν_{max}/cm^{-1} 3314, 2937, 1746, 1659, 1652, 1600, 1538, 1498, 1443, 1380, 1297, 1253, 1177. **HRMS (ESI-TOF):** *m*/*z* [M + H]$^+$ calcd for $C_{19}H_{19}N_2O_3$, 323.13902; found, 323.1398. **TLC:** R_f = 0.22 (75:25 hexane:EtOAc).

Compound 9w – ^{1}H NMR (300 MHz, Chloroform-*d*): (70:30 mixture of diastereomers)

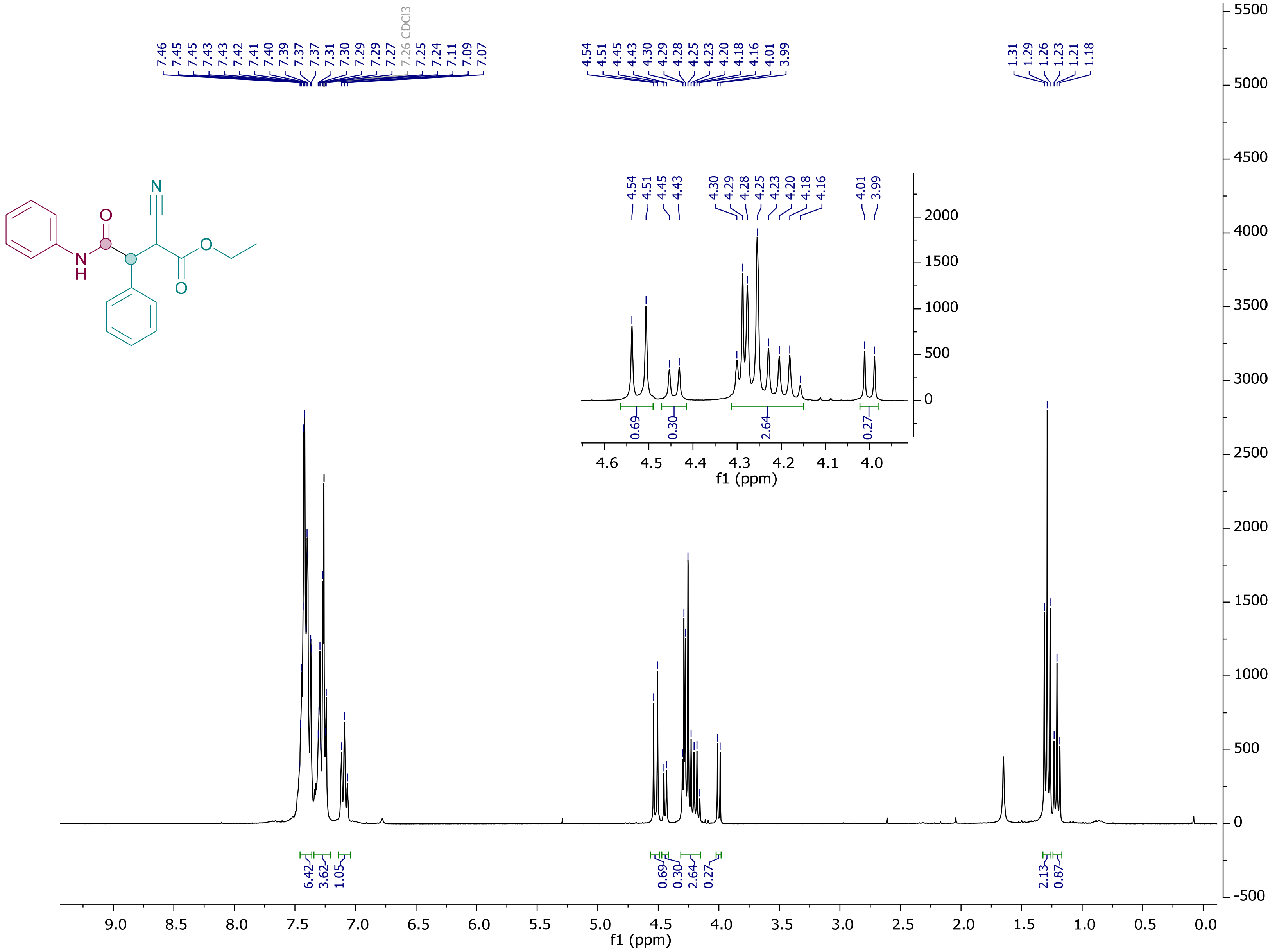

Compound 9w – ^{13}C NMR (75 MHz, Chloroform-*d*): (70:30 mixture of diastereomers)

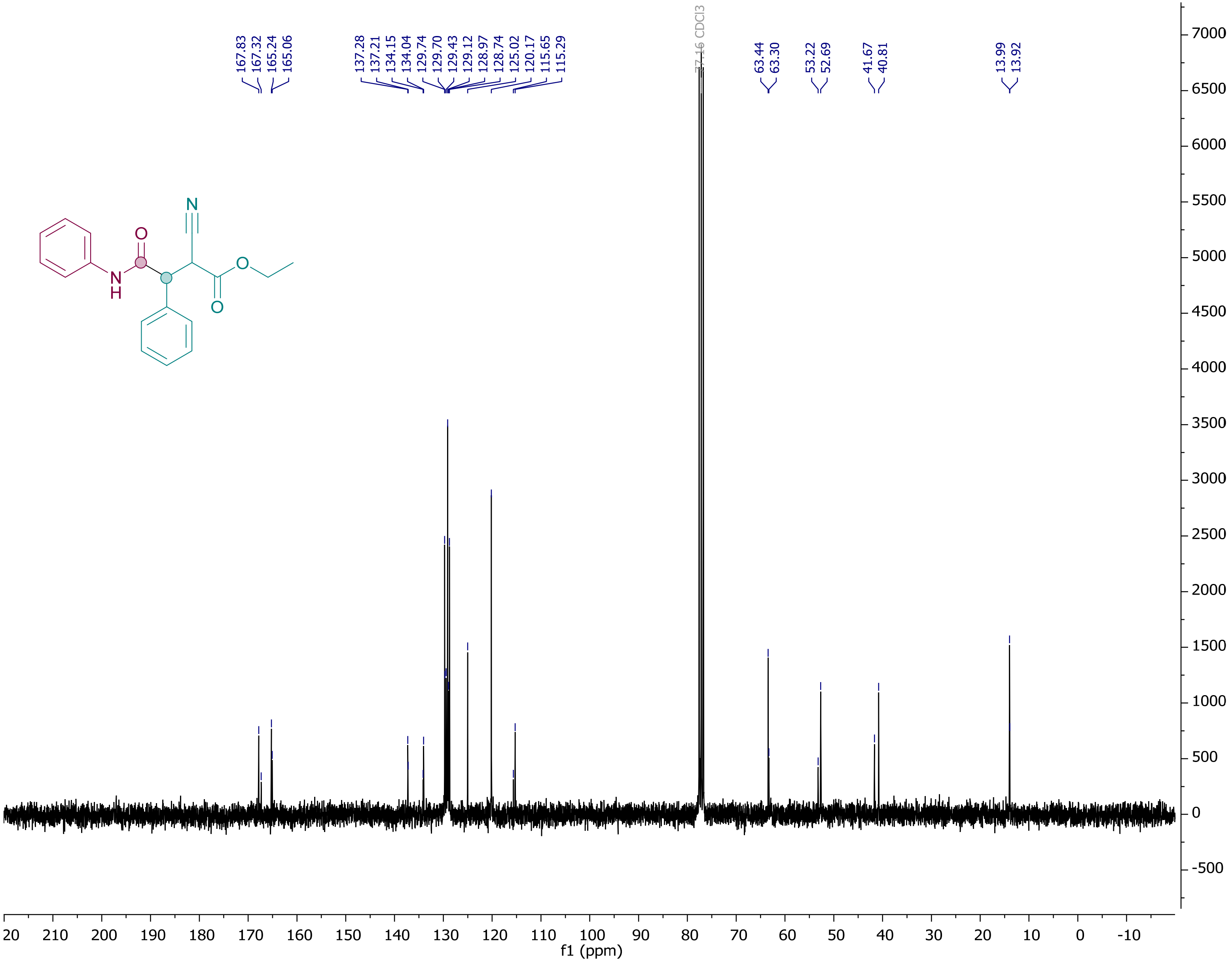

Ethyl 4-(cyclohexylcarbamoyl)-2-oxochromane-3-carboxylate (9x)

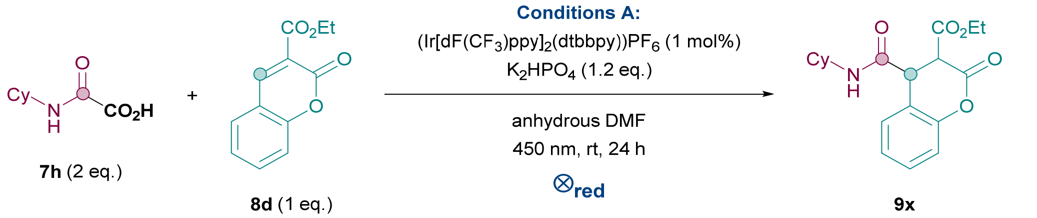

Chemical Formula: $C_{19}H_{23}NO_5$
Molecular Weight: 345.40

General procedure for Conditions A was followed. Ethyl 3-coumarincarboxylate **8d** (26.2 mg, 0.12 mmol, 1.0 eq.), *N*-(cyclohexyl)-oxoacetic acid **7h** (41.5 mg, 0.24 mmol, 2.0 eq.), $(Ir[dF(CF_3)ppy]_2(dtbbpy))PF_6$ (1.3 mg, 1.2 µmol, 1 mol%), and K_2HPO_4 (25.4, 0.14 mmol, 1.2 eq.) in anhydrous DMF (0.3 mL) was irradiated with 450 nm for 24 h. The crude was then purified *via* column flash chromatography eluting with 75:25 hexane:EtOAc. The solvent was then removed *in vacuo* to afford a 90:10 diastereomeric mixture of ethyl 4-(cyclohexylcarbamoyl)-2-oxochromane-3-carboxylate **9x** as an off-white foam (26.0 mg, 0.075 mmol, 63%).

Characterisation:

^{1}H NMR (400 MHz, Chloroform-*d*): (90:10 mixture of diastereomers) δ ppm 7.40 – 7.05 (m, 4H, 4 x ArH major + minor), 5.87 (d, *J* = 8.3 Hz, 0.1H, NH minor), 5.75 (d, *J* = 8.2 Hz, 0.9H, NH major), 4.35 – 4.08 (m, 4H, OCH_2 major + minor, 2 x CH major + minor), 3.82 – 3.70 (m, 1H, NCH major + minor), 2.01 – 0.98 (m, 13H, 5 x CH_2 major + minor, CH_3 major + minor). **^{13}C NMR (101 MHz, Chloroform-*d*):** (90:10 mixture of diastereomers) 169.3 (C), 167.7 (C), 167.0 (C), 166.8 (C), 163.7 (C), 163.2 (C), 151.8 (C), 151.2 (C), 130.1 (CH), 129.8 (CH), 127.8 (CH), 127.4 (CH), 125.3 (CH), 124.5 (CH), 121.6 (C), 119.8 (C), 117.8 (CH), 117.7 (CH), 62.6 (CH_2), 62.3 (CH_2), 49.1 (CH plus one overlapping CH), 49.0 (CH), 48.9 (CH), 46.0 (CH plus one overlapping CH), 33.0 (CH_2 plus one overlapping CH_2), 32.9 (CH_2 plus one overlapping CH_2), 25.6 (CH_2), 25.5 (CH_2), 24.93 (CH_2), 24.91 (CH_2), 24.78 (CH_2), 24.76 (CH_2), 14.2 (CH_3), 14.0 (CH_3). **IR:** ν_{max}/cm^{-1} 3277, 3092, 2933, 2854, 1772, 1736, 1637, 1562, 1489, 1458, 1456, 1362, 1263, 1208, 1159. **HRMS (ESI-TOF):** *m/z* $[M + H]^+$ calcd for $C_{19}H_{24}NO_5$, 346.16490; found, 346.1643. **TLC:** R_f = 0.26 (80:20 hexane:EtOAc). **M.p.** 175-179 °C.

NOESY analysis suggests that the major diastereomer is *anti* due to nOe correlation between NH and H_b:

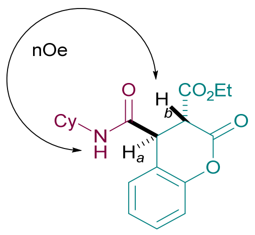

Note that under Conditions B, competitive oxidative rearomatisation occurred:

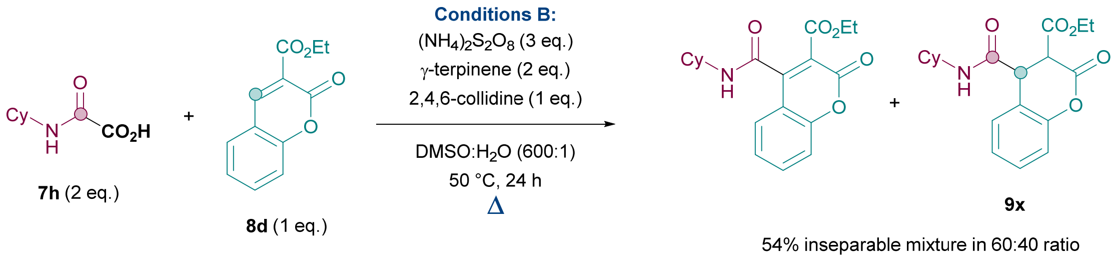

Compound 9x – ¹H NMR (400 MHz, Chloroform-*d*): (90:10 mixture of diastereomers)

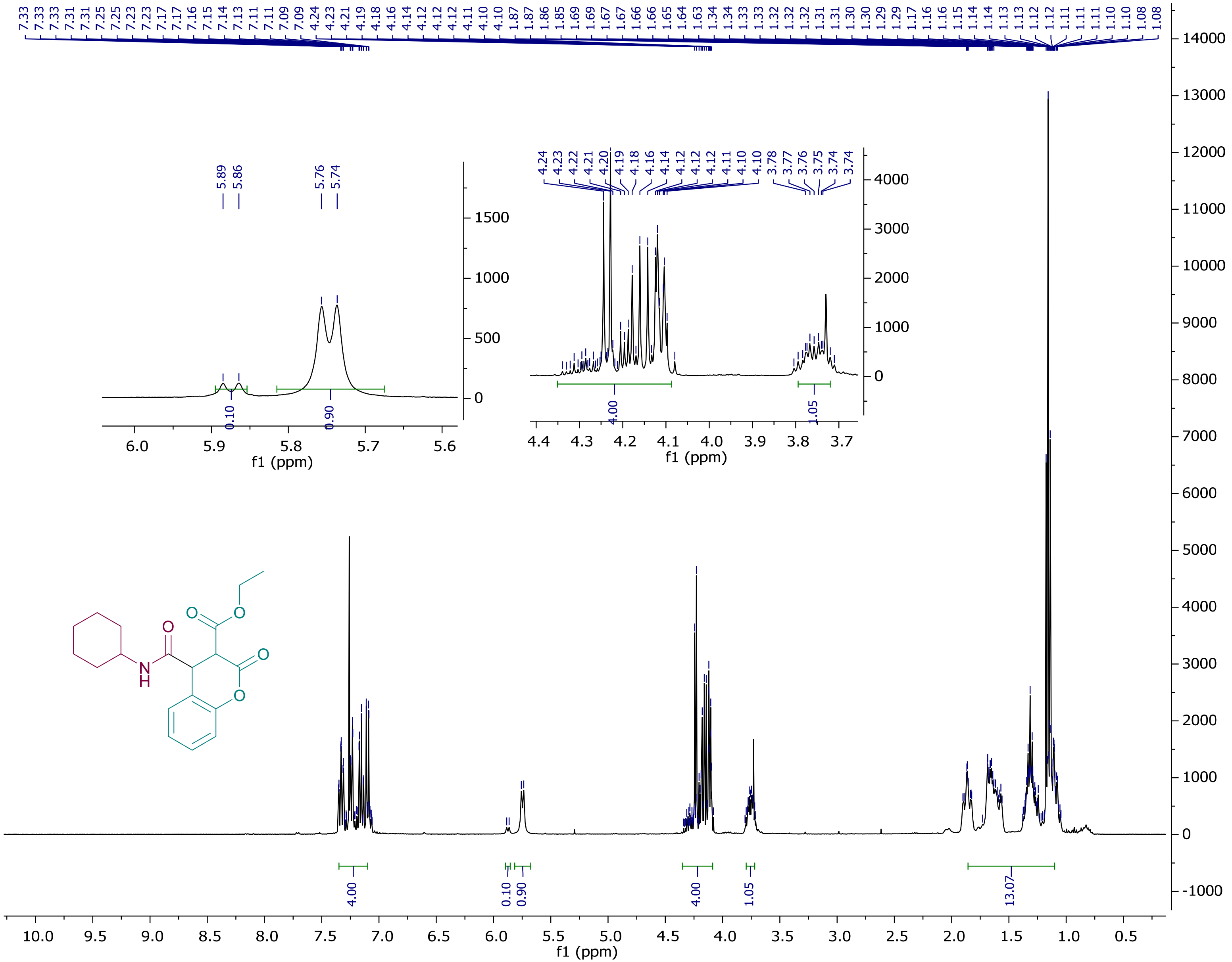

Compound 9x – ^{13}C NMR (101 MHz, Chloroform-*d*): (90:10 mixture of diastereomers)

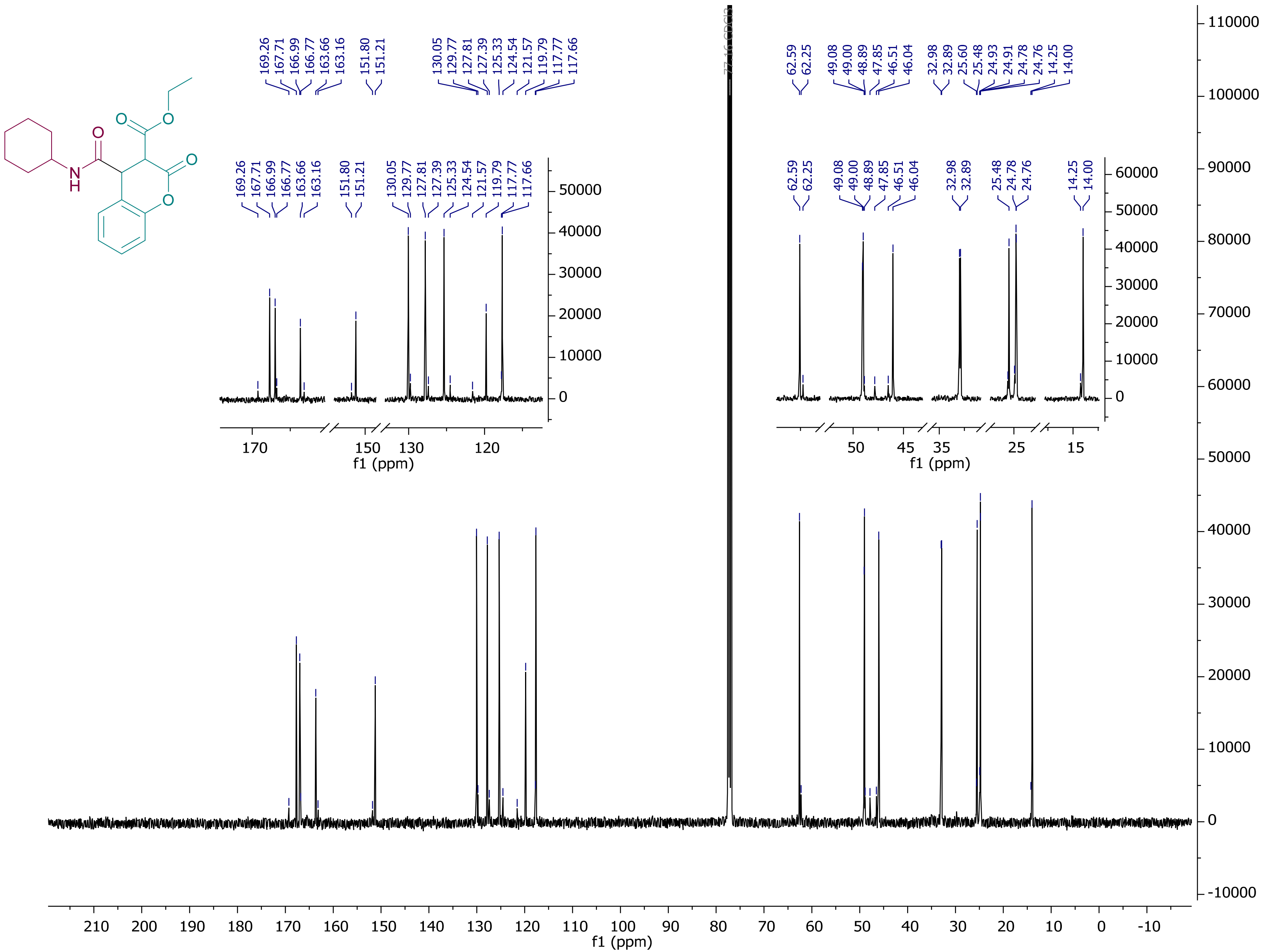

Compound 9x – NOESY (400 MHz, Chloroform-*d*): (90:10 mixture of diastereomers). Key signals for major diastereomer annotated.

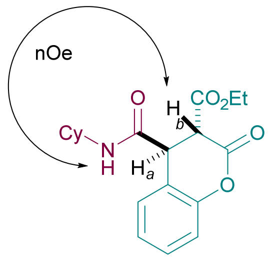

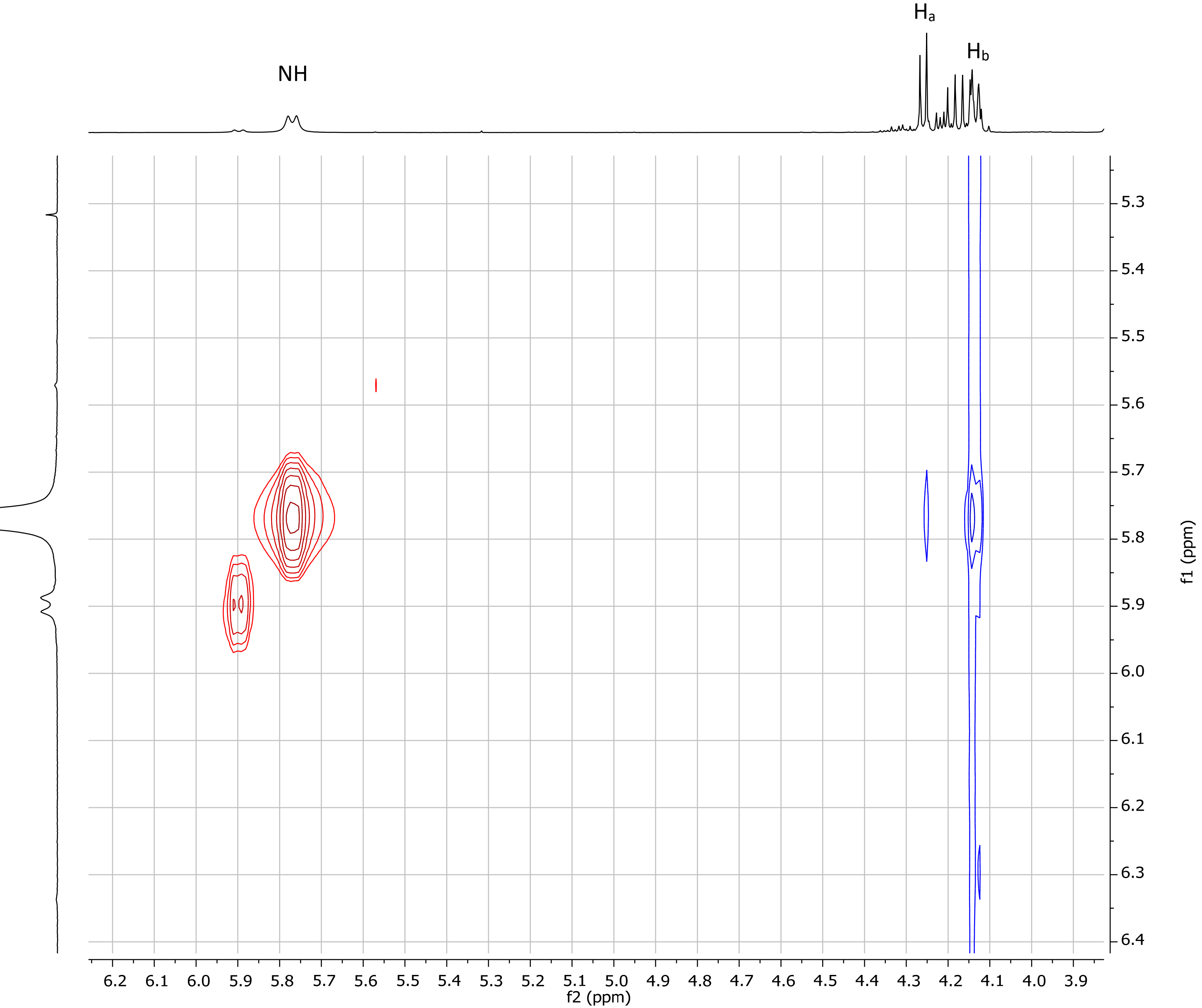

3-Oxo-*N*-phenylcyclopentane-1-carboxamide (9y)

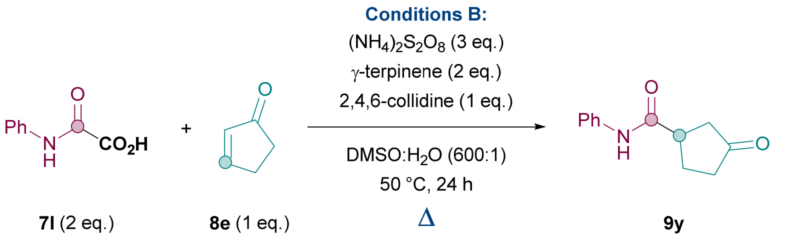

Chemical Formula: $C_{12}H_{13}NO_2$
Molecular Weight: 203.24

General procedure for Conditions B was followed. Cyclopent-2-en-1-one **8e** (9.9 mg, 0.12 mmol, 1.0 eq.), *N*-(phenyl)-oxoacetic acid **7l** (39.7 mg, 0.24 mmol, 2.0 eq.), $(NH_4)_2S_2O_8$ (82.5 mg, 0.36 mmol, 3.0 eq.), γ-terpinene (38.5 µL, 0.24 mmol, 2 eq.), and 2,4,6-collidine (15.9 µL, 0.12 mmol, 1.0 eq.) in DMSO:H_2O (600:1) (0.4 mL) was heated at 50 °C for 24 h followed by work-up X. The crude was then purified *via* column flash chromatography eluting with 35:65 hexane:EtOAc. The solvent was then removed *in vacuo* to afford 3-Oxo-*N*-phenylcyclopentane-1-carboxamide **9y** as a light brown solid (15.7 mg, 0.077 mmol, 64%).

Characterisation:

^{1}H NMR (300 MHz, Chloroform-*d*): δ ppm 7.64 (br s, 1H, NH), 7.51 (d, *J* = 7.8 Hz, 2H, ArH), 7.31 (t, *J* = 7.9 Hz, 2H, ArH), 7.12 (t, *J* = 7.4 Hz, 1H, ArH), 3.05 (p, *J* = 7.5 Hz, 1H, CH), 2.65 (ddd, *J* = 18.3, 8.5, 1.4 Hz, 1H, C<u>H</u>H), 2.56 – 2.36 (m, 2H, CH_2), 2.36 – 2.12 (m, 3H, CH_2, CH<u>H</u>). **^{13}C NMR (75 MHz, Chloroform-*d*):** δ ppm 217.1 (C), 172.2 (C), 137.7 (C), 129.2 (CH), 124.8 (CH), 120.2 (CH), 43.6 (CH), 41.8 (CH_2), 37.6 (CH_2), 27.4 (CH_2). **IR:** ν_{max}/cm^{-1} 3335, 3134, 3051, 2961, 2899, 1728, 1679, 1600, 1541, 1505, 1489, 1441, 1402, 1312, 1248, 1234, 1220, 1194. **HRMS (ESI-TOF):** *m/z* $[M + H]^+$ calcd for $C_{12}H_{14}NO_2$, 204.10191; found, 204.1020. **TLC:** R_f = 0.39 (30:70 hexane:EtOAc). **m.p.** 97-101 °C

Compound 9y – ^{1}H NMR (300 MHz, Chloroform-*d*):

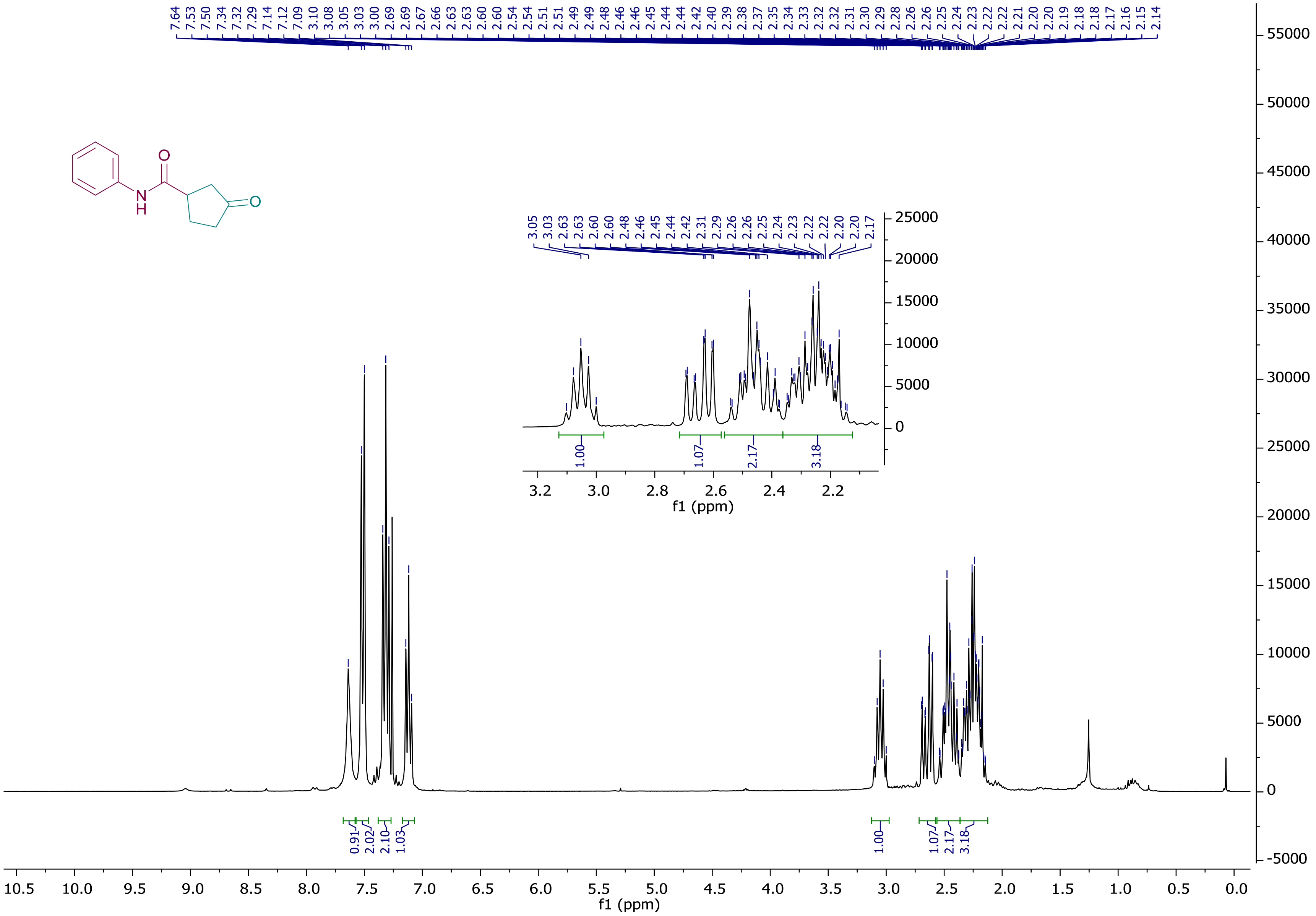

Compound 9y – ^{13}C NMR (75 MHz, Chloroform-*d*):

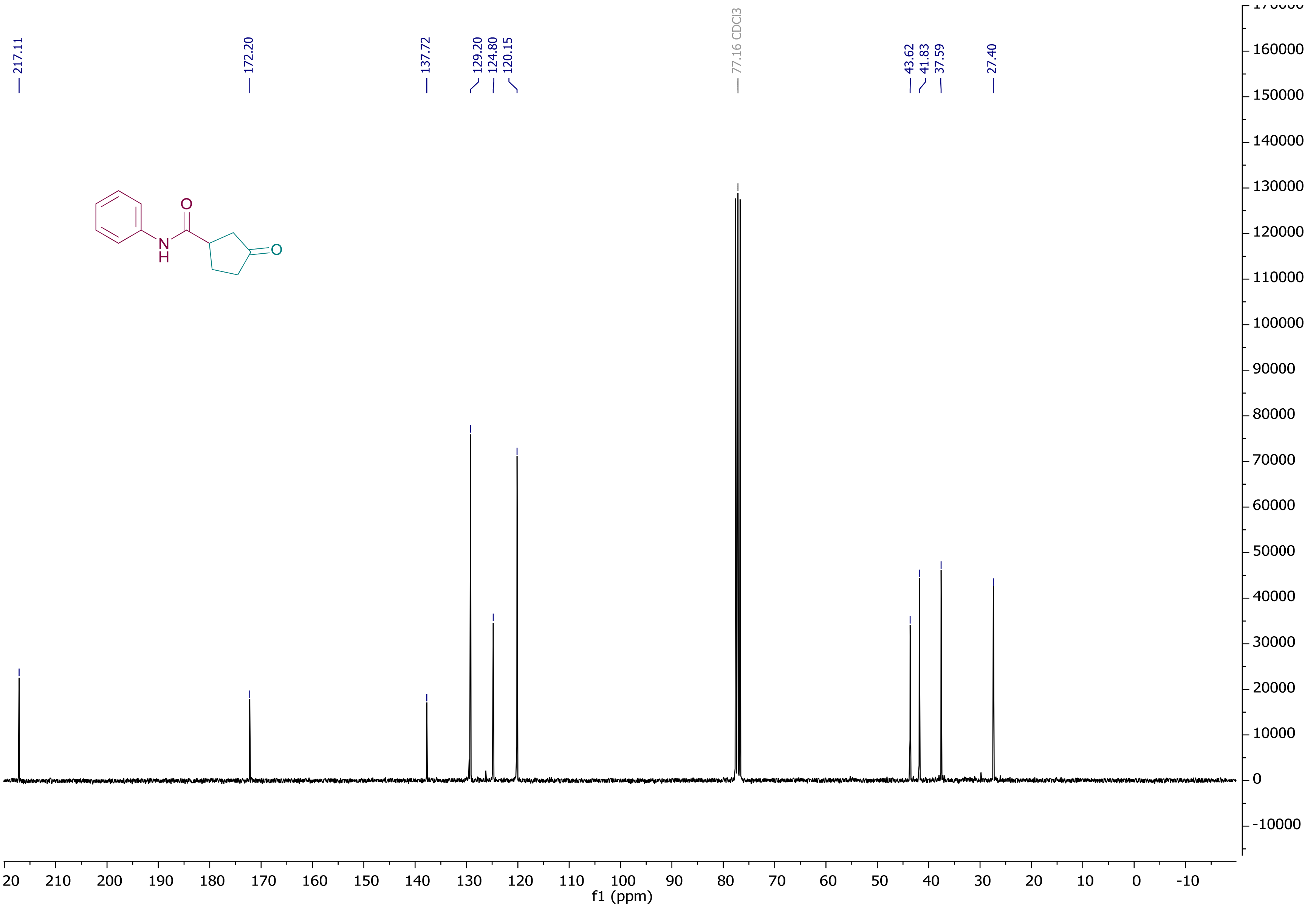

3-Oxo-*N*-phenylcyclohexane-1-carboxamide (9z)

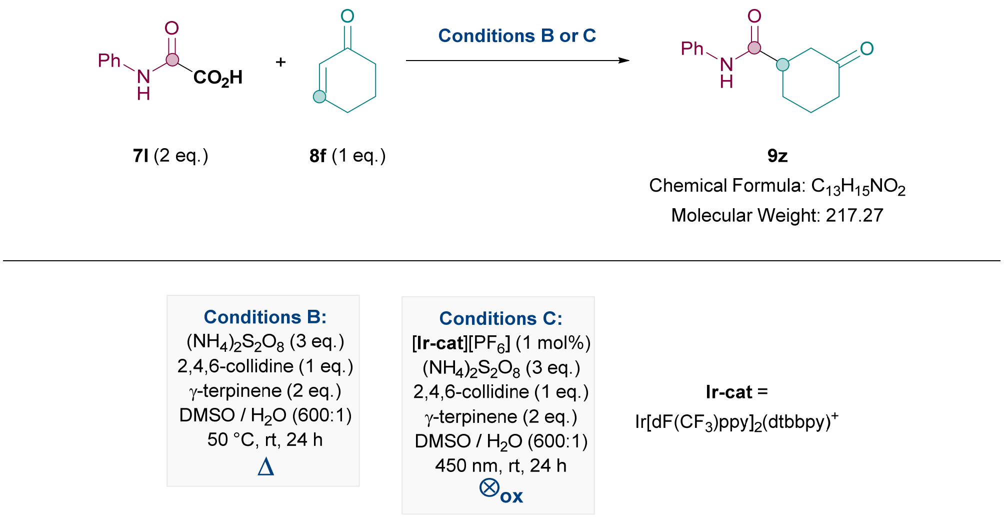

Using Conditions B: General procedure for Conditions B was followed. Cyclohex-2-en-1-one **8f** (11.5 mg, 0.12 mmol, 1.0 eq.), *N*-(phenyl)-oxoacetic acid **7l** (40.0 mg, 0.24 mmol, 2.0 eq.), $(NH_4)_2S_2O_8$ (82.2 mg, 0.36 mmol, 3.0 eq.), γ-terpinene (38.5 µL, 0.24 mmol, 2 eq.), and 2,4,6-collidine (15.9 µL, 0.12 mmol, 1.0 eq.) in DMSO:H_2O (600:1) (0.4 mL) was heated at 50 ºC for 24 h followed by work-up X. The crude was then purified *via* column flash chromatography eluting with 55% 45:55 hexane:EtOAc. The solvent was then removed *in vacuo* to afford 3-oxo-*N*-phenylcyclohexane-1-carboxamide **9z** as a pale-yellow solid (19.6 mg, 0.090 mmol, 75%).

Using Conditions C: General procedure for Conditions C was followed. Cyclohex-2-en-1-one **8f** (11.7 mg, 0.12 mmol, 1.0 eq.), *N*-(phenyl)-oxoacetic acid **7l** (40.0 mg, 0.24 mmol, 2.0 eq.), $(NH_4)_2S_2O_8$ (82.3 mg, 0.36 mmol, 3.0 eq.), Ir[dF(CF_3)ppy](dtbppy)PF_6 (1.4 mg, 1 µmol, 1 mol%), γ-terpinene (38.5 µL, 0.24 mmol, 2.0 eq.), and 2,4,6-collidine (15.9 µL, 0.12 mmol, 1.0 eq.) in DMSO:H_2O (600:1) (0.4 mL) was irradiated with 450 nm for 24 h. The crude was then purified *via* column flash chromatography eluting with 70:30 hexane:EtOAc. The solvent was then removed *in vacuo* to afford diethyl 2-(1-oxo-1-((4-butyl)phenylamino)propan-2-yl)malonate **9m** as an off-white solid (18.3 mg, 0.084 mmol, 69%).

Characterisation:

^{1}H NMR (300 MHz, Chloroform-*d*): δ ppm 7.62 (br s, 1H, NH), 7.52 (d, *J* = 7.7 Hz, 2H, ArH), 7.31 (t, *J* = 7.9 Hz, 2H, ArH), 7.11 (t, *J* = 7.4 Hz, 1H, ArH), 2.82 – 2.64 (m, 2H, CH, C<u>H</u>HCO), 2.59 – 2.46 (m, 1H, CH<u>H</u>CO), 2.44 – 2.34 (m, 2H, CH_2), 2.23 – 1.90 (m, 3H, C<u>H</u>H, CH_2), 1.83 – 1.61 (m, 1H, CH<u>H</u>). **^{13}C NMR (75 MHz, Chloroform-*d*):** δ ppm 210.4 (C), 171.6 (C), 137.8 (C), 129.2 (CH), 124.7 (CH), 120.1 (CH), 46.5 (CH), 44.0 (CH_2), 41.1 (CH_2), 28.5 (CH_2), 24.9 (CH_2). **IR:** ν_{max}/cm^{-1} 3323, 2953, 2922, 2860, 1699, 1688, 1677, 1645, 1598, 1543, 1533, 1490 1440, 1408, 1348, 1311, 1298, 1235, 1181. **HRMS (ESI-TOF):** *m*/*z* [M + H]$^{+}$ calcd for $C_{13}H_{16}NO_2$, 218.11756; found, 218.1186. **TLC:** R_f = 0.32 (40:60 hexane:EtOAc). **m.p.** 123-127 °C

Compound 9z– ^{1}H NMR (300 MHz, Chloroform-*d*):

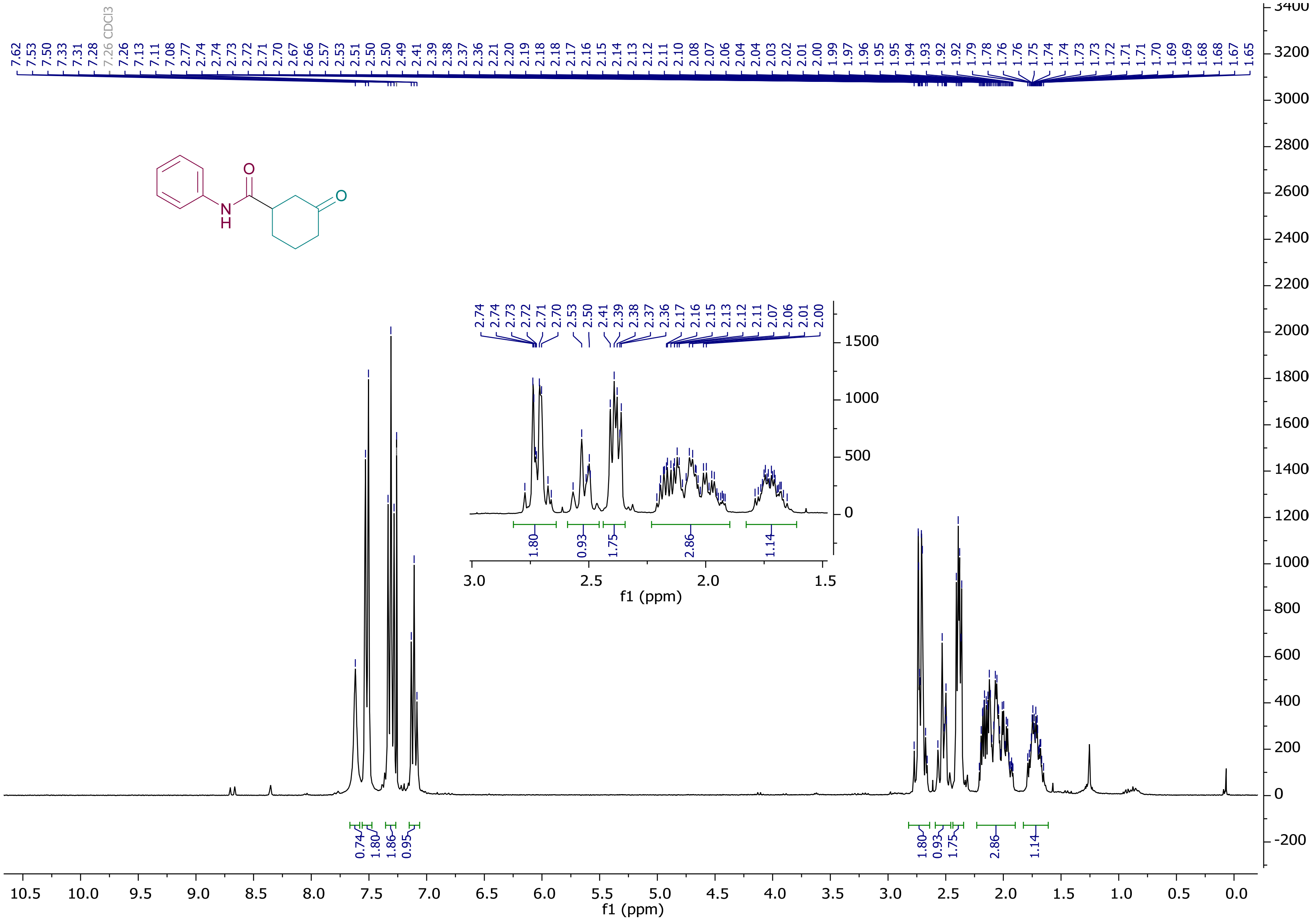

Compound 9z – ^{13}C NMR (75 MHz, Chloroform-*d*):

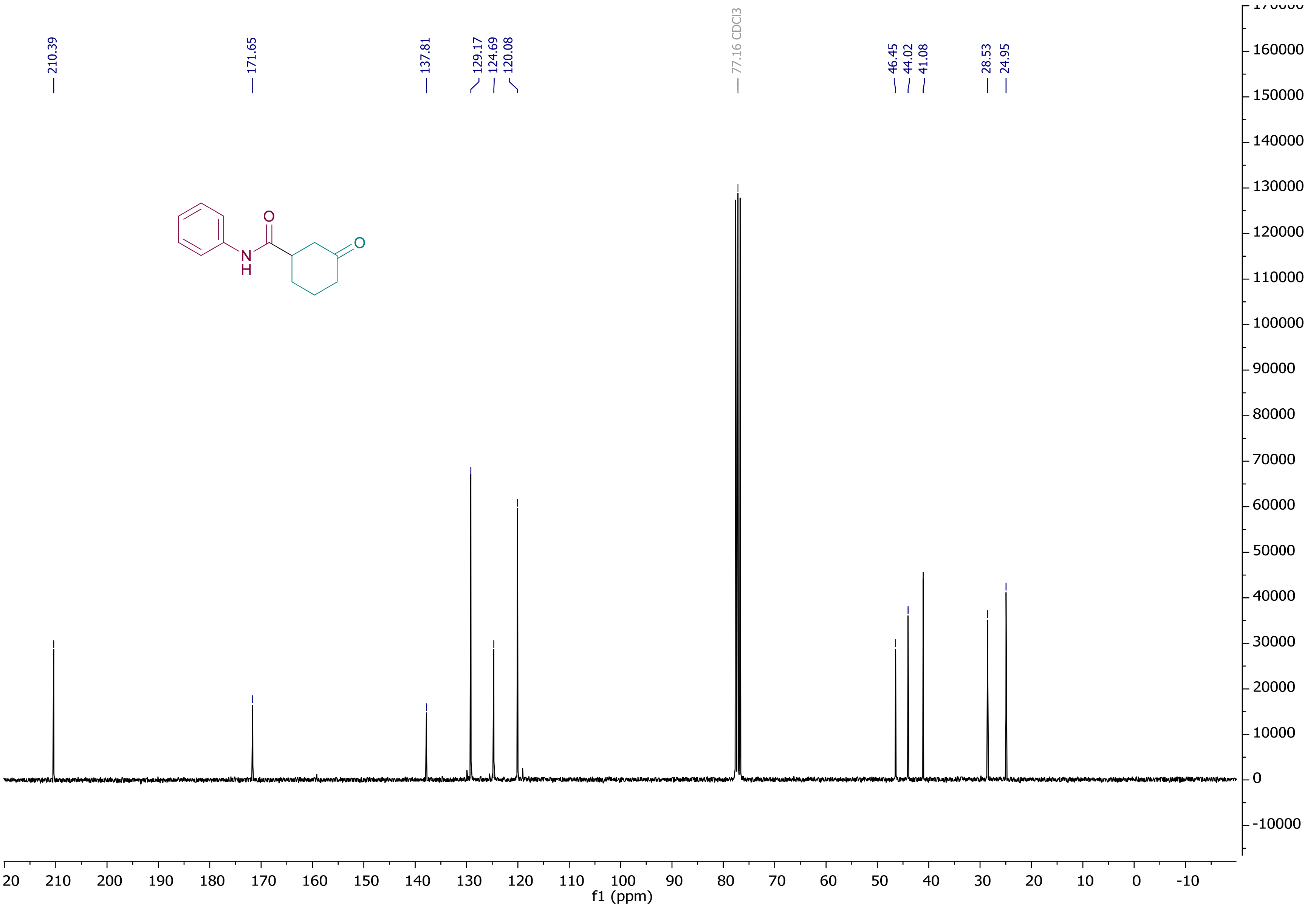

N-(Adamantan-1-yl)-3-oxocycloheptane-1-carboxamide (9aa)

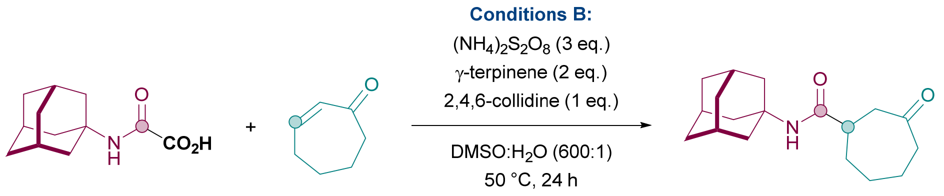

7k (2 eq.) **8g** (1 eq.) **9aa**

Chemical Formula: $C_{18}H_{27}NO_2$

Molecular Weight: 289.42

General procedure for Conditions B was followed. Cyclohept-2-en-1-one **8g** (13.3 mg, 0.12 mmol, 1.0 eq.), *N*-(1-adamantyl)-oxoacetic acid **7k** (53.7 mg, 0.24 mmol, 2.0 eq, $(NH_4)_2S_2O_8$ (82.3 mg, 0.36 mmol, 3.0 eq.), γ-terpinene (38.5 µL, 0.24 mmol, 2.0 eq.), and 2,4,6-collidine (15.9 µL, 0.12 mmol, 1.0 eq.) in DMSO:H_2O (600:1) (0.4 mL) was heated at 50 ºC for 24 h followed by work-up Y. The crude was then purified *via* column flash chromatography eluting with 70:30→60:40 hexane:EtOAc. The solvent was then removed *in vacuo* to afford *N*-(adamantan-1-yl)-3-oxocycloheptane-1-carboxamide **9aa** as a yellow solid (25.6 mg, 0.089 mmol, 74%).

Characterisation:

^{1}H NMR (400 MHz, Chloroform-*d*): δ ppm 5.17 (br s, 1H, NH), 2.85 (dd, *J* = 14.1, 10.8 Hz, 1H, C<u>H</u>H), 2.63 – 2.47 (m, 2H, CH<u>H</u>, C<u>H</u>H), 2.47 – 2.33 (m, 1H, CH<u>H</u>), 2.24 (tt, *J* = 10.5, 2.5 Hz, 1H, CH), 2.05 (br s, 3H, 3 x CH), 2.01 – 1.89 (m, 8H, 3 x CH_2, C<u>H</u>H, C<u>H</u>H), 1.88 – 1.68 (m, 2H, C<u>H</u>H, CH<u>H</u>), 1.65 (m, 7H, 3 x CH_2, CH<u>H</u>), 1.47 – 1.31 (m, 1H, CH<u>H</u>). **^{13}C NMR (101 MHz, Chloroform-*d*):** δ ppm 213.4 (C), 173.5 (C), 52.0 (C), 46.5 (CH_2), 44.3 (CH), 44.1 (CH_2), 41.7 (CH_2), 36.4 (CH_2), 34.4 (CH_2), 29.5 (CH), 28.0 (CH_2), 23.7 (CH_2). **IR:** ν_{max}/cm^{-1} 3305, 2904, 2849, 1702, 1656, 1640, 1541, 1451, 1358, 1344, 1309, 1291, 1265, 1217, 1094. **HRMS (ESI-TOF):** *m/z* $[M + H]^+$ calcd for $C_{18}H_{28}NO_2$, 290.21146; found, 290.2117. **TLC:** R_f = 0.29 (60:40 hexane:EtOAc). **m.p.** 147-151 °C

Compound 9aa – ^{1}H NMR (400 MHz, Chloroform-*d*):

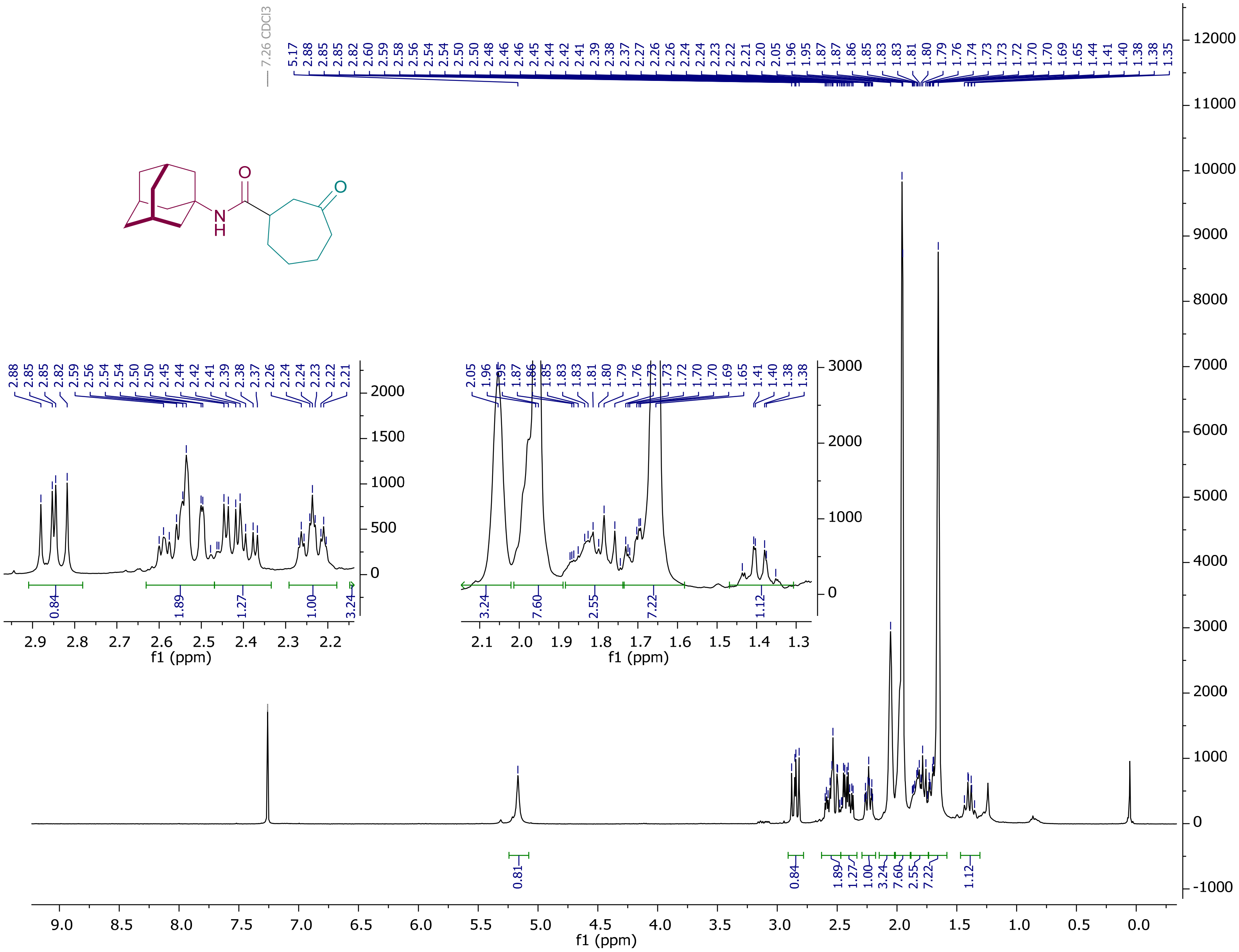

Compound 9aa – ^{13}C NMR (101 MHz, Chloroform-*d*):

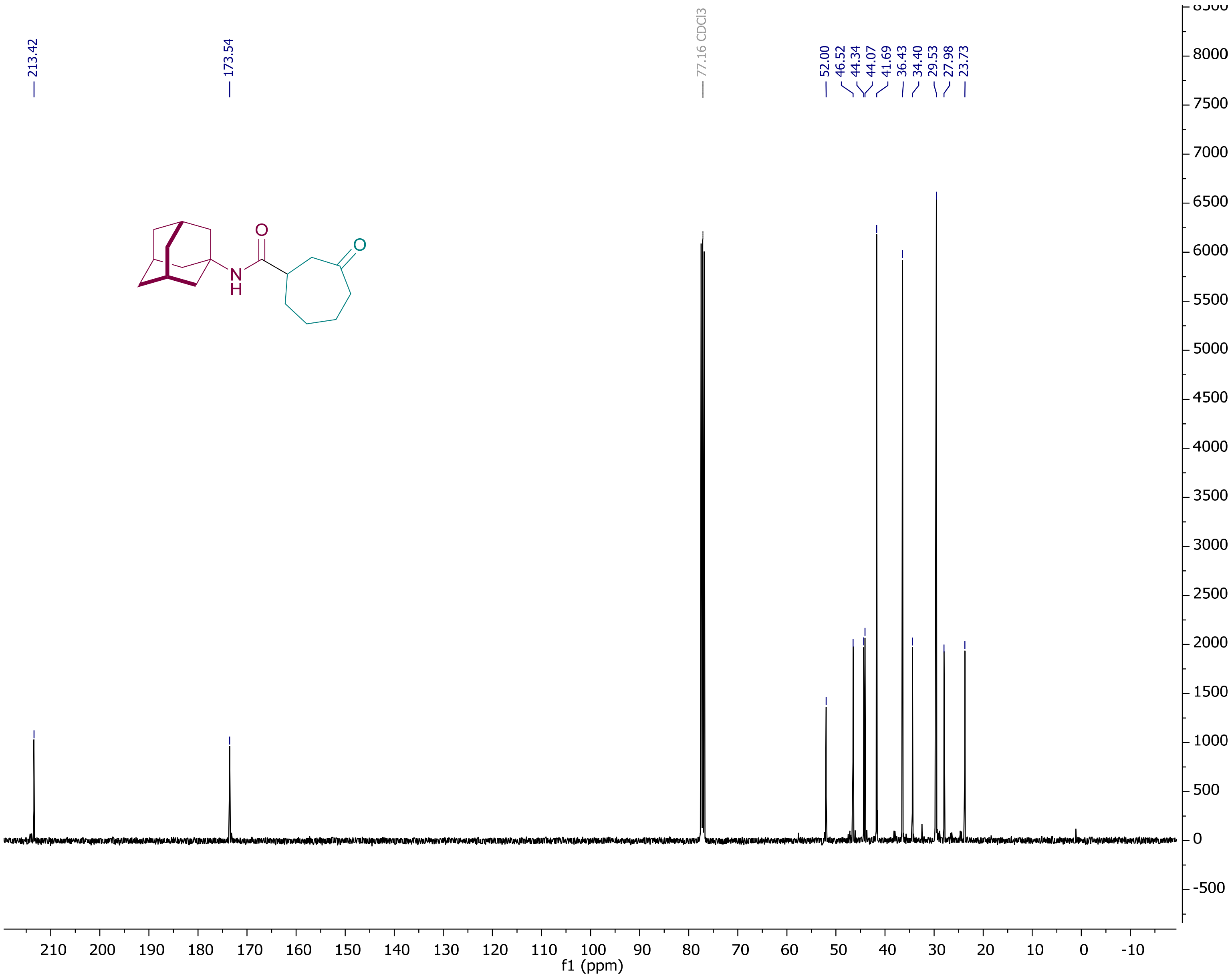

5-Oxo-*N*-phenyltetrahydrofuran-3-carboxamide (9ab)

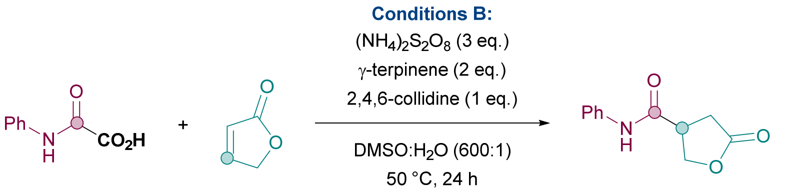

7l (2 eq.) **8h** (1 eq.) **9ab**

Chemical Formula: $C_{11}H_{11}NO_3$

Molecular Weight: 205.21

General procedure for Conditions B was followed. Cyclopent-2-en-1-one **8h** (9.9 mg, 0.12 mmol, 1.0 eq.), *N*-(phenyl)-oxoacetic acid **7l** (39.7 mg, 0.24 mmol, 2.0 eq.), $(NH_4)_2S_2O_8$ (82.5 mg, 0.36 mmol, 3.0 eq.), γ-terpinene (38.5 µL, 0.24 mmol, 2 eq.), and 2,4,6-collidine (15.9 µL, 0.12 mmol, 1.0 eq.) in DMSO:H_2O (600:1) (0.4 mL) was heated at 50 °C for 24 h followed by work-up X. The crude was then purified *via* column flash chromatography eluting with 35:65 hexane:EtOAc. The solvent was then removed *in vacuo* to afford 5-oxo-*N*-phenyltetrahydrofuran-3-carboxamide **9ab** as an off-white solid (14.0 mg, 0.068 mmol, 57%).

Characterisation:

^{1}H NMR (400 MHz, Acetone-*d$_6$*): δ ppm 9.43 (br s, 1H, NH), 77.65 (d, *J* = 7.6 Hz, 1H, ArH), 7.31 (d, *J* = 8.0 Hz, 1H, ArH), 7.08 (t, *J* = 7.5 Hz, 1H, ArH), 4.56 (dd, *J* = 9.0, 8.1 Hz, 1H, OC<u>H</u>H), 4.44 (dd, *J* = 9.0, 6.0 Hz, 1H, OCH<u>H</u>), 3.68 (dddd, *J* = 9.1, 8.1, 6.8, 6.0 Hz, 1H, CH) 2.90 – 2.67 (m, 2H, CH_2CO). **^{13}C NMR (101 MHz, Acetone-*d$_6$*):** δ ppm 176.3 (C), 170.7 (C), 139.9 (C), 129.6 (CH plus one overlapping CH), 124.6 (CH), 120.3 (CH plus one overlapping CH), 70.7 (CH_2), 42.6 (CH), 31.9 (CH_2). **IR:** $ν_{max}/cm^{-1}$ 3314, 2936, 1758, 1564, 1599, 1541, 1521, 1469, 1443, 1380, 1523, 1178. **HRMS (ESI-TOF):** *m/z* $[M + H]^+$ calcd for $C_{11}H_{12}NO_3$, 206.08117; found, 206.0820. **TLC:** R_f = 0.33 (30:70 hexane:EtOAc). **m.p.** 130-134 °C

Compound 9ab – ^{1}H NMR (400 MHz, Acetone-d_6):

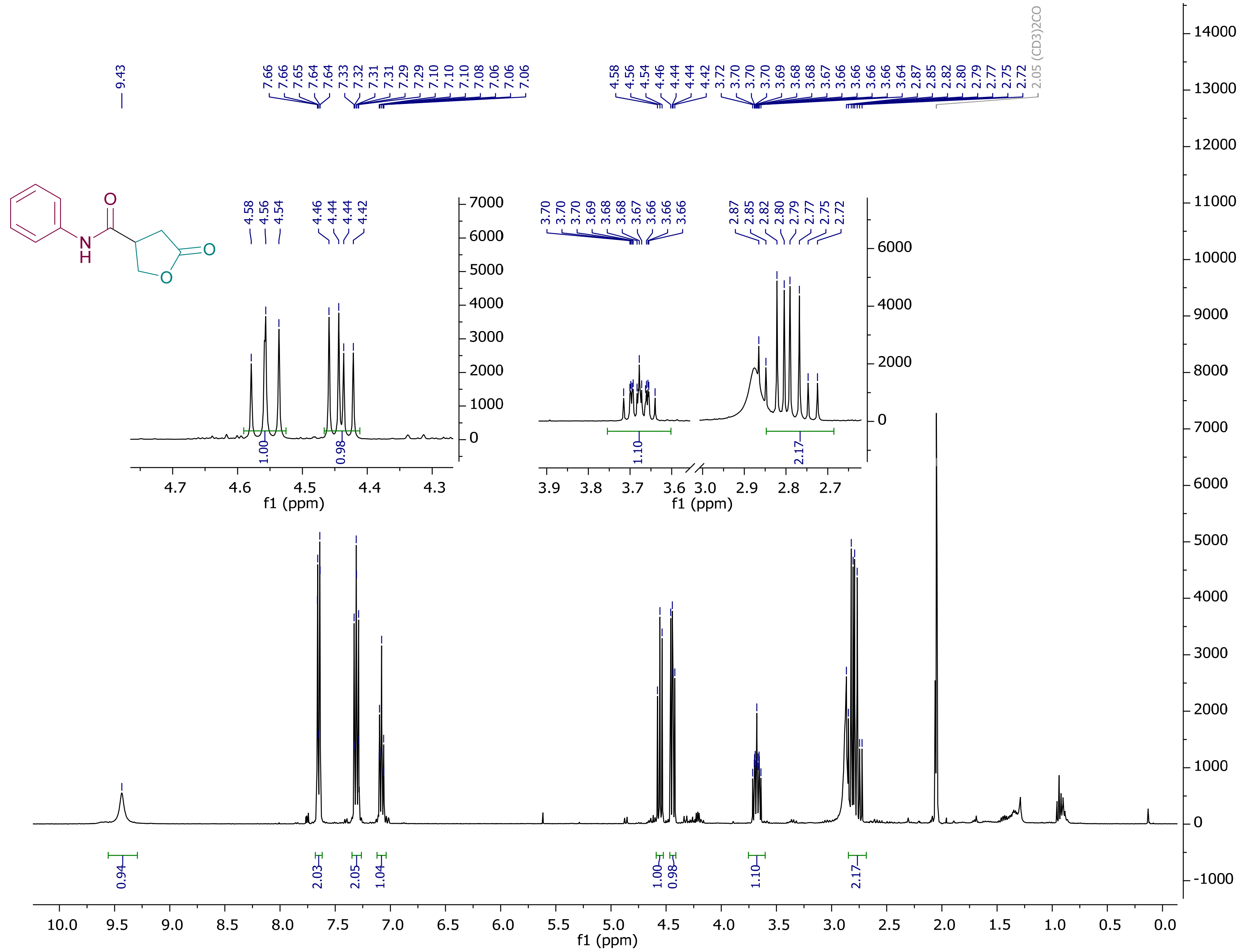

Compound 9ab – ^{13}C NMR (101 MHz, Acetone-d_6):

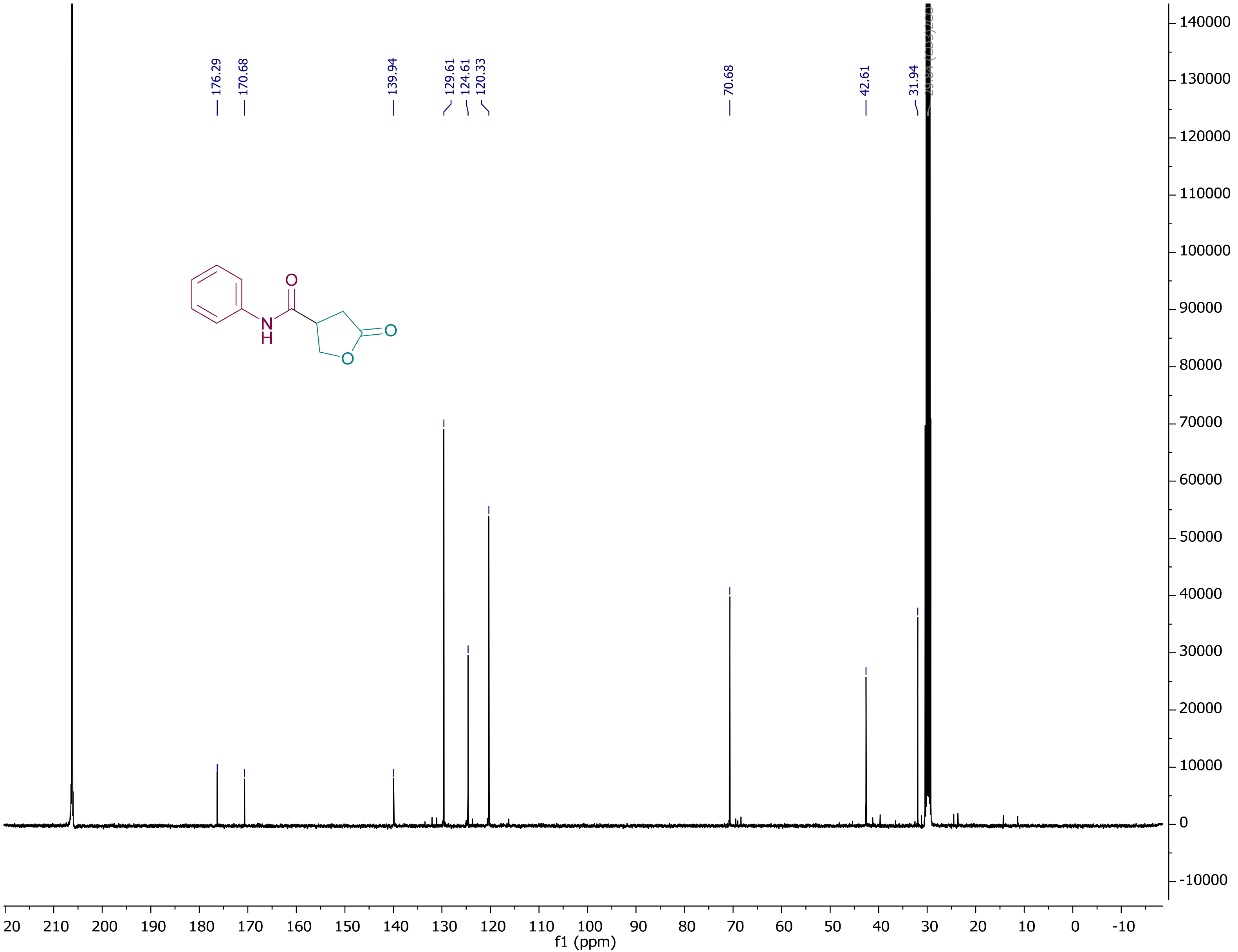

***N*-(Cyclohexyl)-2-oxotetrahydro-2H-pyran-4-carboxamide (9ac)**

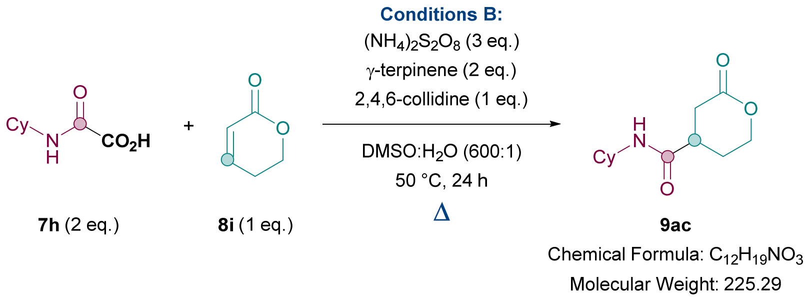

General procedure for conditions B was followed. 5,6-Dihydro-2H-pyran-2-one **8i** (11.9 mg, 0.12 mmol, 1.0 eq.), *N*-(cyclohexyl)-oxoacetic acid **7h** (41.4 mg, 0.24 mmol, 2.0 eq), $(NH_4)_2S_2O_8$ (82.2 mg, 0.36 mmol, 3.0 eq.), γ-terpinene (38.5 µL, 0.24 mmol, 2.0 eq.), and 2,4,6-collidine (15.9 µL, 0.12 mmol, 1.0 eq.) in DMSO:H_2O (600:1) (0.4 mL) was heated at 50 °C for 24 h followed by work-up B. The crude was then purified *via* column flash chromatography eluting with 20:80 hexane:EtOAc. The solvent was then removed *in vacuo* to afford methyl *N*-(cyclohexyl)-2-oxotetrahydro-2H-pyran-4-carboxamide **9ac** as an off-white solid (12.9 mg, 0.056 mmol, 47%).

Characterisation:

^{1}H NMR (400 MHz, Chloroform-*d*): δ ppm 5.58 (br d, *J* = 6.7 Hz, 1H, NH), 4.45 (dt, *J* = 11.2, 5.6 Hz, 1H, OC<u>H</u>H), 4.28 (dt, *J* = 11.7, 5.8 Hz, 1H, OCH<u>H</u>), 3.81 – 3.66 (m, 1H, NCH), 2.90 – 2.78 (m, 1H, C<u>H</u>HCO), 2.74 – 2.57 (m, 2H, CH, CH<u>H</u>CO), 2.10 – 1.98 (m, 2H, CH_2), 1.95 – 1.83 (m, 2H, 2 x C<u>H</u>H), 1.76 – 1.65 (m, 2H, 2 x C<u>H</u>H), 1.66 – 1.56 (m, 1H, C<u>H</u>H), 1.42 – 1.22 (m, 2H, 2 x CH<u>H</u>), 1.23 – 1.02 (m, 3H, 2 x CH<u>H</u>, CH<u>H</u>). **^{13}C NMR (101 MHz, Chloroform-*d*):** δ ppm 171.6 (C), 170.8 (C), 67.5 (CH_2), 48.7 (CH), 38.0 (CH), 33.19 (CH_2), 33.16 (CH_2), 32.6 (CH_2), 26.4 (CH_2), 25.5 (CH_2), 24.9 (CH_2 plus one overlapping CH_2). **IR:** ν_{max}/cm^{-1} 3309, 3075, 2930, 2853, 2359, 1711, 1634, 1547, 1456, 1444, 1471, 1422, 1403, 1302, 1257, 1219, 1170. **HRMS (ESI-TOF):** *m*/*z* $[M + H]^+$ calcd for $C_{12}H_{20}NO_3$, 226.14377; found, 226.1434. **TLC:** R_f = 0.23 (20:80 hexane:EtOAc). **m.p.** 100-104 °C

Compound 9ac – ^{1}H NMR (400 MHz, Chloroform-*d*):

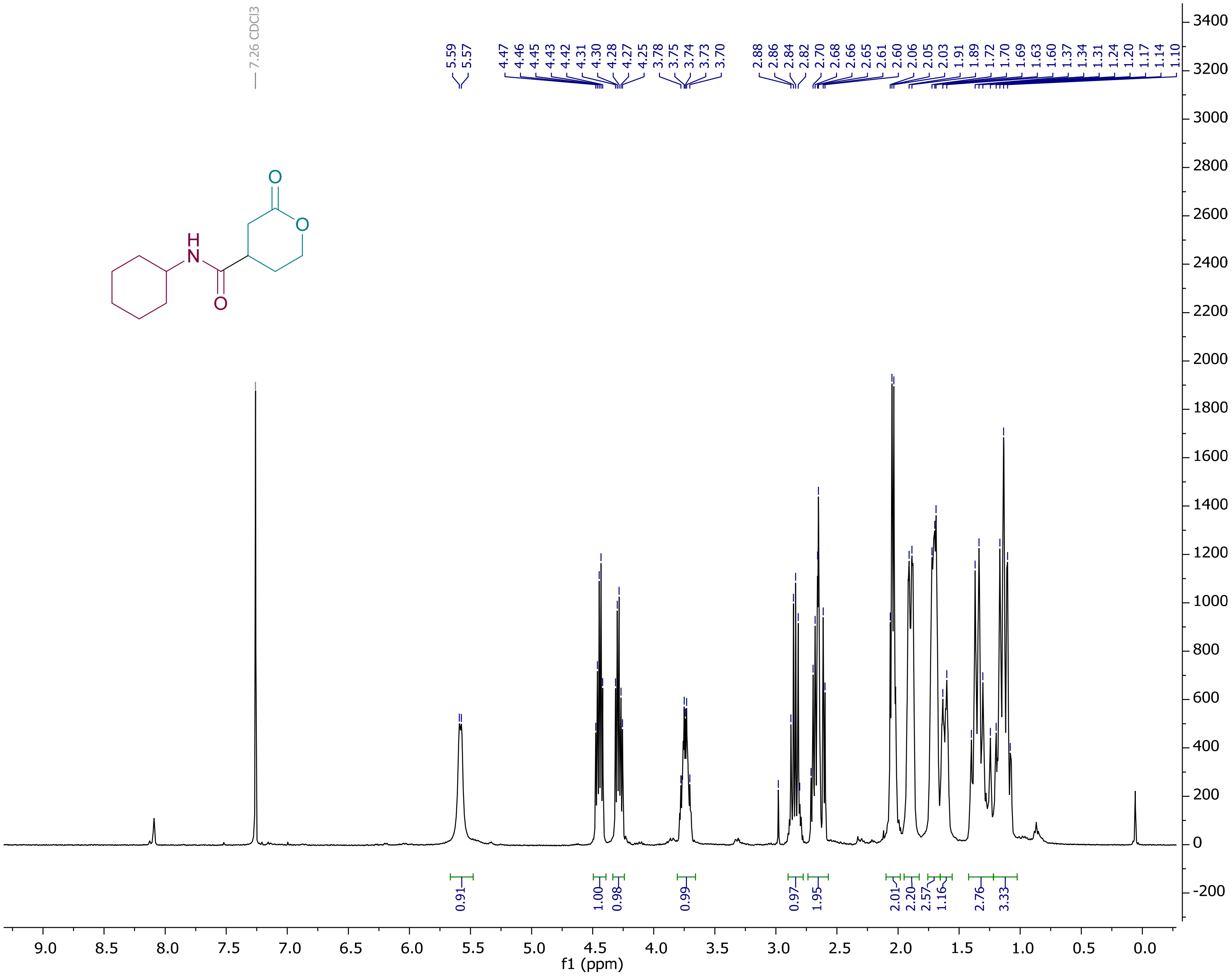

Compound 9ac – ^{13}C NMR (101 MHz, Chloroform-*d*):

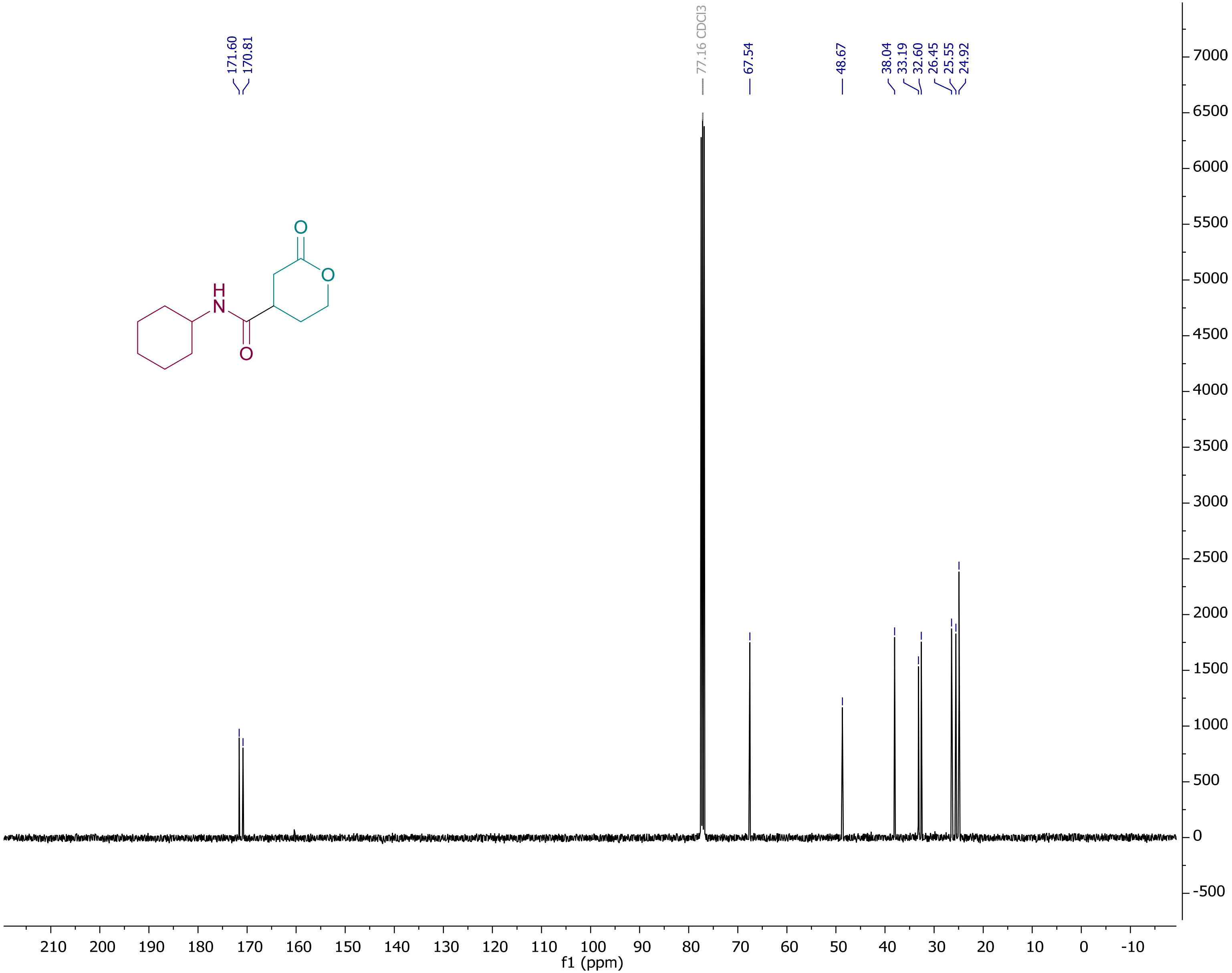

***t*-Butyl 2-oxo-4-(phenylcarbamoyl)pyrrolidine-1-carboxylate (9ad)**

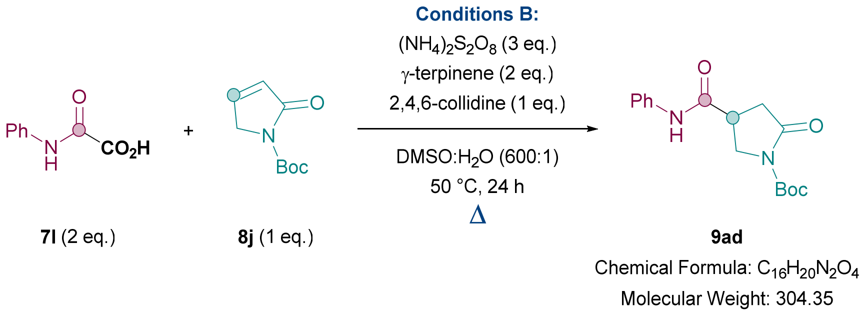

General procedure for Conditions B was followed. *t*-Butyl 2-oxo-2,5-dihydro-1H-pyrrole-1-carboxylate **8j** (22.1 mg, 0.12 mmol, 1.0 eq.), *N*-(phenyl)-oxoacetic acid **7l** (39.8 mg, 0.24 mmol, 2.0 eq.), $(NH_4)_2S_2O_8$ (82.4 mg, 0.36 mmol, 3.0 eq.), γ-terpinene (38.5 µL, 0.24 mmol, 2.0 eq.), and 2,4,6-collidine (15.9 µL, 0.12 mmol, 1.0 eq.) in DMSO:H_2O (600:1) (0.4 mL) was heated at 50 °C for 24 h followed by work-up X. The crude was then purified *via* column flash chromatography eluting with 70:30→25:75 hexane:EtOAc. The solvent was then removed *in vacuo* to afford *t*-butyl 2-oxo-4-(phenylcarbamoyl)pyrrolidine-1-carboxylate **9ad** as an off-white solid (19.8 mg, 0.064 mmol, 54%).

Characterisation:

^{1}H NMR (400 MHz, Chloroform-*d*): δ ppm 9.42 (br s, 1H, NH), 7.66 (d, *J* = 7.6 Hz, 2H, ArH), 7.30 (d, *J* = 8.0 Hz, 2H, ArH), 7.07 (t, *J* = 7.4 Hz, 1H, ArH), 4.03 (dd, *J* = 10.8, 8.6 Hz, 1H, C<u>H</u>HNBoc), 3.91 (dd, *J* = 10.8, 6.4 Hz, 1H, CH<u>H</u>NBoc), 3.41 (tt, *J* = 8.7, 6.4 Hz, 1H, CHCON), 2.88 – 2.65 (m, 2H, CH_2CO), 1.49 (s, 9H, 3 x CH_3). **^{13}C NMR (101 MHz, Chloroform-*d*):** the ^{13}C NMR spectra shows two rotamers at room temperature: δ ppm 172.0 (2 x C), 171.4 (C), 171.3 (C), 150.7 (2 x C), 140.1 (C), 140.0 (C), 129.6 (2 x CH), 124.5 (2 x CH), 120.3 (CH), 120.2 (CH), 82.5 (2 x C), 49.7 (2 x CH_2), 37.7 (CH), 37.6 (CH), 36.7 (2 x CH_2), 28.2 (2 x CH_3). **IR:** ν_{max}/cm^{-1} 3323, 2972, 2922, 1774, 1668, 1620, 1544, 1493, 1446, 1362, 1299, 1254, 1150. **HRMS (ESI-TOF):** *m/z* $[M + H]^+$ calcd for $C_{16}H_{27}NO_4$, 305.14958; found, 305.1499. **TLC:** R_f = 0.26 (30:70 hexane:EtOAc). **m.p.** 155-158 °C.

Compound 9ad – ^{1}H NMR (400 MHz, Acetone-d_6):

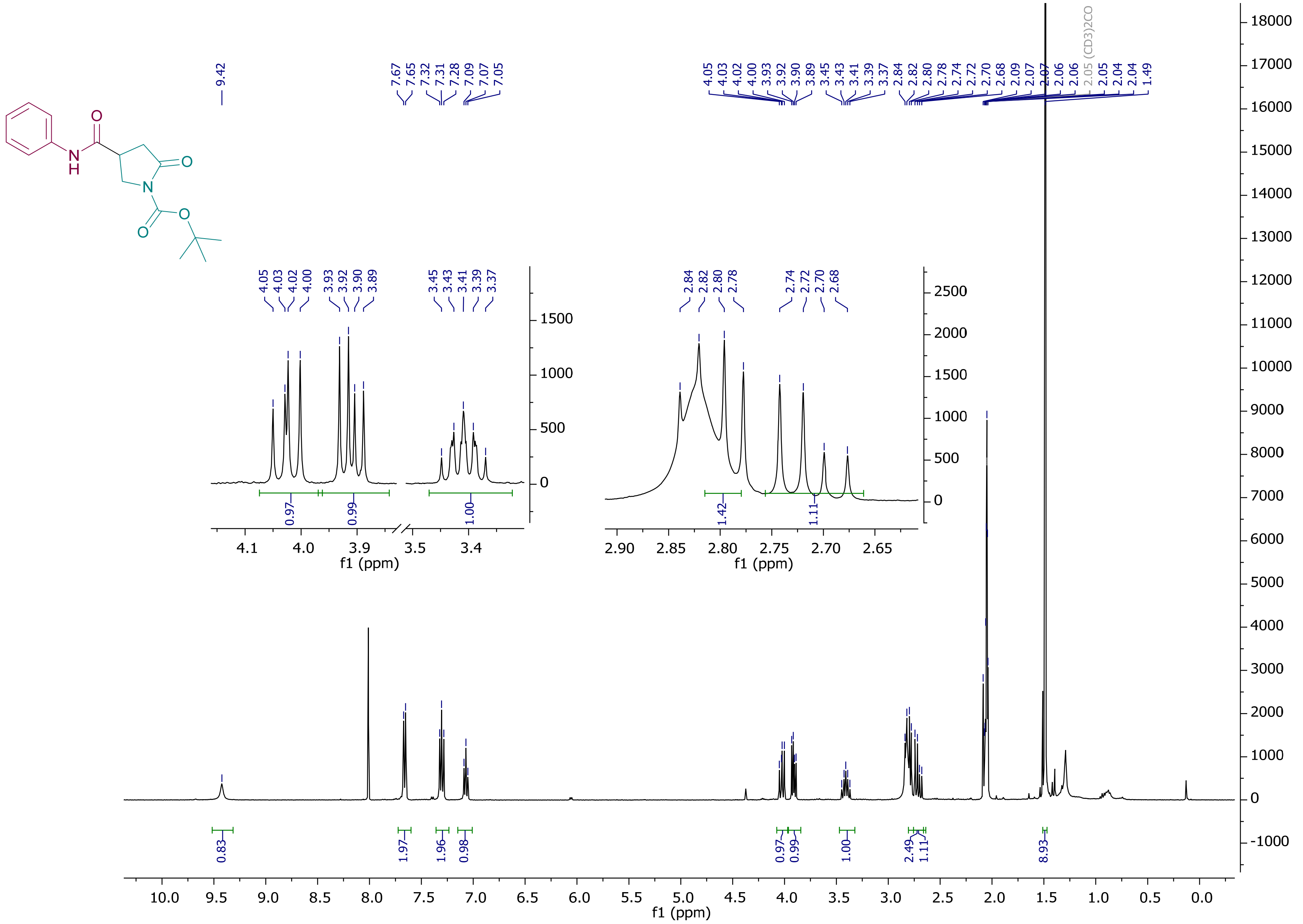

Compound 9ad – ^{13}C NMR (101 MHz, Acetone-d_6):

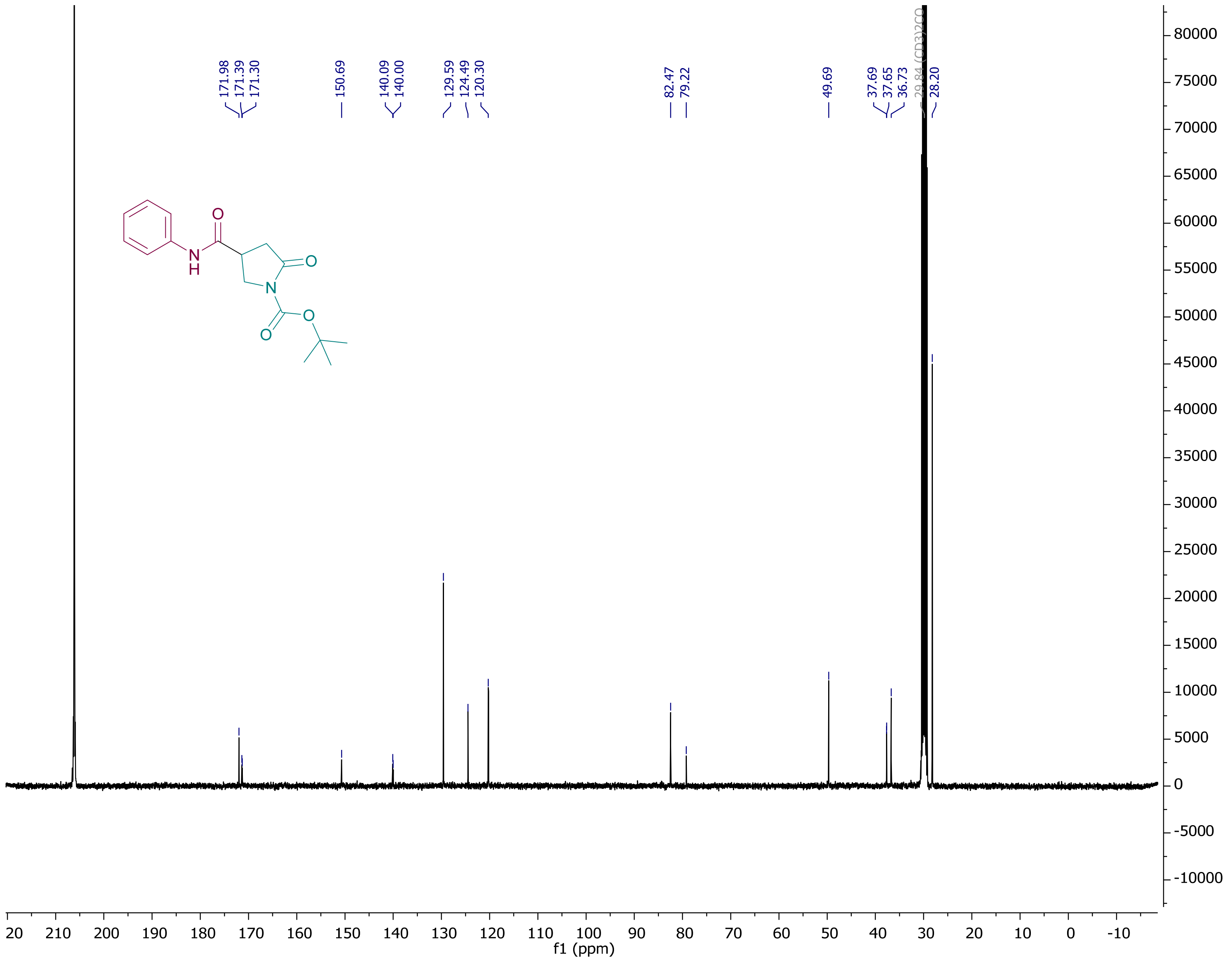

2-Methyl-3-oxo-*N*-phenylcyclohexane-1-carboxamide (9ae)

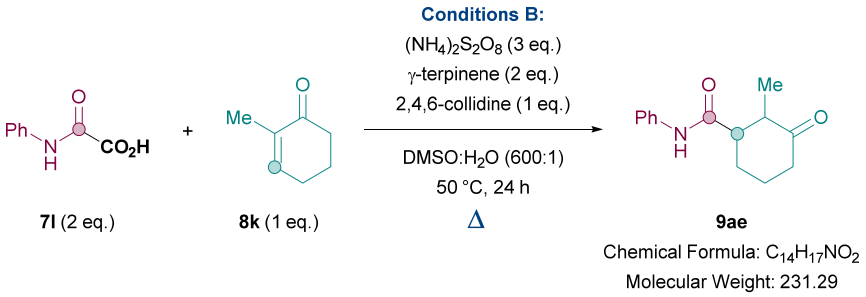

General procedure for Conditions B was followed. 2-Methyl-2-cyclohexen-1-one **8k** (13.2 mg, 0.12 mmol, 1.0 eq.), *N*-(phenyl)-oxoacetic acid **7l** (39.8 mg, 0.24 mmol, 2.0 eq.), $(NH_4)_2S_2O_8$ (82.4 mg, 0.36 mmol, 3.0 eq.), γ-terpinene (38.5 µL, 0.24 mmol, 2.0 eq.), and 2,4,6-collidine (15.9 µL, 0.12 mmol, 1.0 eq.) in DMSO:H_2O (600:1) (0.4 mL) was heated at 50 ºC for 24 h followed by work-up Y. Analysis of the crude ^{1}H NMR spectra using dibromomethane as the internal standard determined the yield to be 59% with a 60:40 mixture of diastereomers. The crude was then purified *via* column flash chromatography eluting with 70:30→50:50 hexane:EtOAc to separate out the diastereomers but the samples were impure after the first purification. For characterisation purposes, both diastereomers were purified further, prioritising pure samples for characterisation rather than isolated yields. The major diastereomer was purified *via* flash column chromatography eluting with 75:25 hexane:EtOAc to give a white solid for characterisation. The minor diastereomer was re-purified *via* flash column chromatography eluting with 65:35 hexane:EtOAc to give a white solid for characterisation.

Characterisation of Major Diastereomer:

^{1}H NMR (400 MHz, Chloroform-*d*): δ ppm 7.54 (d, *J* = 7.6 Hz, 2H, ArH), 7.38 – 7.30 (m, 3H, ArH, NH), 7.13 (t, *J* = 7.4 Hz, 1H, ArH), 2.87 (td, *J* = 12.6, 6.6 Hz, 1H, CH), 2.52 – 2.35 (m, 2H, CH_2), 2.29 – 2.14 (m, 2H, C<u>H</u>H), 2.12 – 2.03 (m, 2H, CH_2), 1.79 – 1.65 (m, 1H, CH<u>H</u>), 1.06 (d, *J* = 6.5 Hz, 3H, CH_3).**^{13}C NMR (101 MHz, Chloroform-*d*):** δ ppm 211.9 (C), 171.5 (C), 137.6 (C), 129.2 (CH), 124.9 (CH), 120.1 (CH), 55.2 (CH), 46.9 (CH), 41.4 (CH_2), 29.7 (CH_2), 26.2 (CH_2), 12.6 (CH_3). **IR:** ν_{max}/cm^{-1} 3313, 2192, 3064. 2974, 2933, 2898, 2874, 1703, 1660, 1596, 1524, 1498, 1439, 1382, 1329, 1310, 1265, 1246, 1770. **HRMS (ESI-TOF):** *m*/*z*

[M + H]$^+$ calcd for $C_{14}H_{18}NO_2$, 232.13321; found 232.1324. **TLC:** R_f = 0.22 (70:30 hexane:EtOAc). **m.p.** 120-124 °C.

Characterisation of Minor Diastereomer:

^{1}H NMR (400 MHz, Chloroform-*d*): δ ppm 7.51 (d, J = 7.7 Hz, 2H, ArH), 7.31 (t, J = 7.9 Hz, 2H, ArH), 7.17 (br s, 1H, NH), 7.11 (t, J = 7.4 Hz, 1H, ArH), 2.96 (q, J = 4.5 Hz, 1H, CH), 2.63 – 2.48 (m, 2H, CH, C$\underline{H}$H), 2.35 – 2.00 (m, 4H, CH$\underline{H}$, C$\underline{H}$H, CH_2), 1.95 – 1.86 (m, 1H, CH$\underline{H}$), 1.16 (d, J = 6.7 Hz, 2H, CH_3).**^{13}C NMR (101 MHz, Chloroform-*d*):** δ ppm 210.4 (C), 171.0 (C), 137.7 (C), 129.2 (CH), 124.6 (CH), 119.8 (CH), 51.3 (CH), 46.4 (CH), 39.9 (CH_2), 27.8 (CH_2), 23.0 (CH_2), 12.8 (CH_3). **IR:** ν_{max}/cm^{-1} 3302, 3269, 3199, 3134, 3084, 2932, 2868, 1695, 1677, 1538, 1493, 1440, 1393, 1373, 1306, 1245, 1185. **HRMS (ESI-TOF):** *m/z* [M + H]$^+$ calcd for $C_{14}H_{18}NO_2$, 232.13321; found 232.1331. **TLC:** R_f = 0.19 (60:40 hexane:EtOAc). **m.p.** 176-180 °C.

Assignment of diastereomers by coupling constants:

The major diastereomer is assigned as *anti*, based on the multiplicities and J values of the CH peaks. For example, the CH peak at δ 2.87 ppm is a td with J = 12.6, 6.6 Hz. The large 12.6 Hz is only consistent with a $^3J_{axial\text{-}axial}$, indicating the *anti* isomer, where the major conformer will be with both substituents equatorial.

The minor diastereomer is assigned as *syn*, based on the multiplicities and J values of the CH peaks. For example, the CH peak at δ 2.96 ppm is a q with J = 4.5 Hz, which is consistent with $^3J_{axial\text{-}equatorial}$ or $^3J_{equatorial\text{-}equatorial}$, indicating the *syn* isomer.

Compound 9ae (Major Diastereomer) – ^{1}H NMR (400 MHz, Chloroform-*d*):

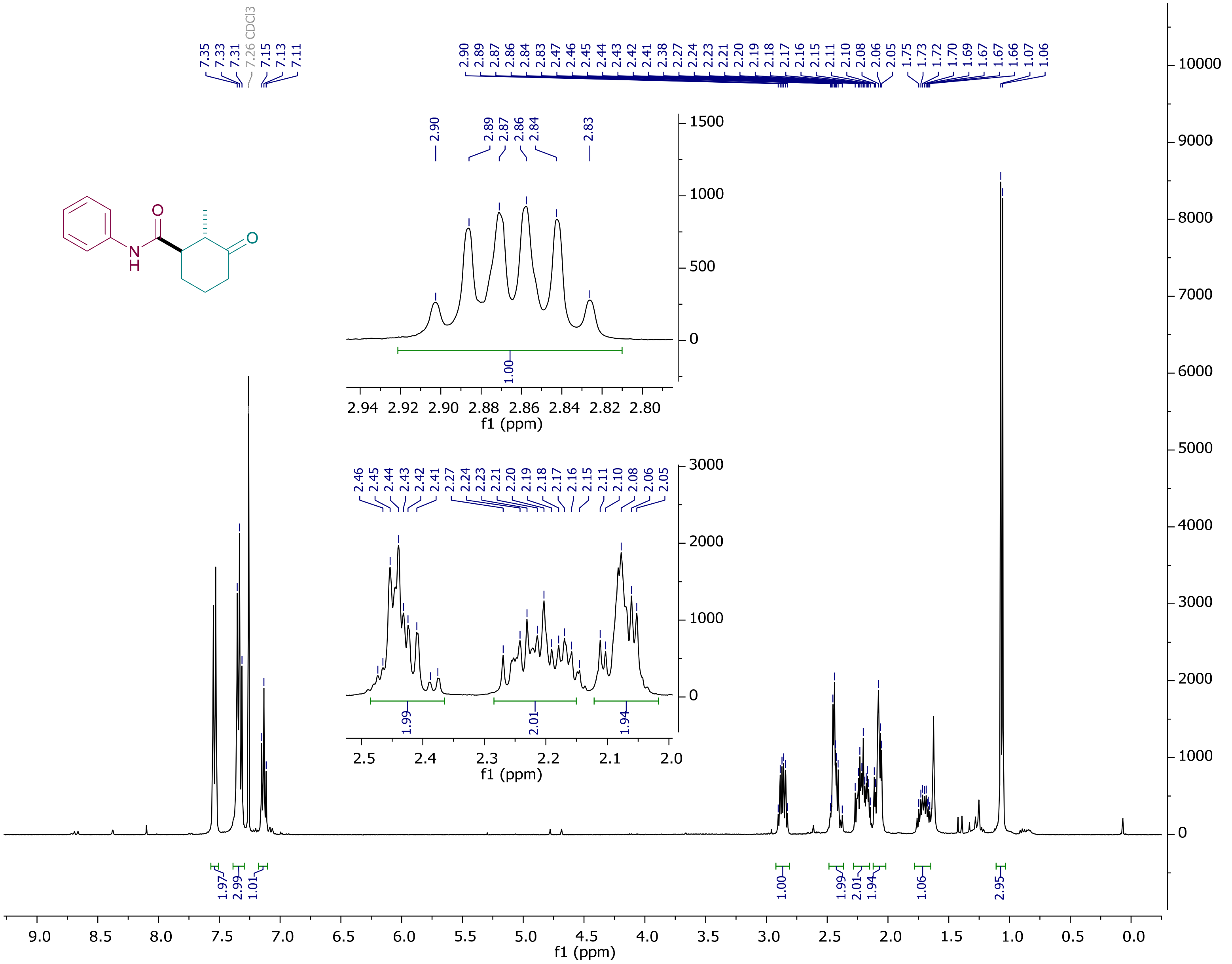

Compound 9ae (Major Diastereomer) – ^{13}C NMR 101 MHz, Chloroform-*d*):

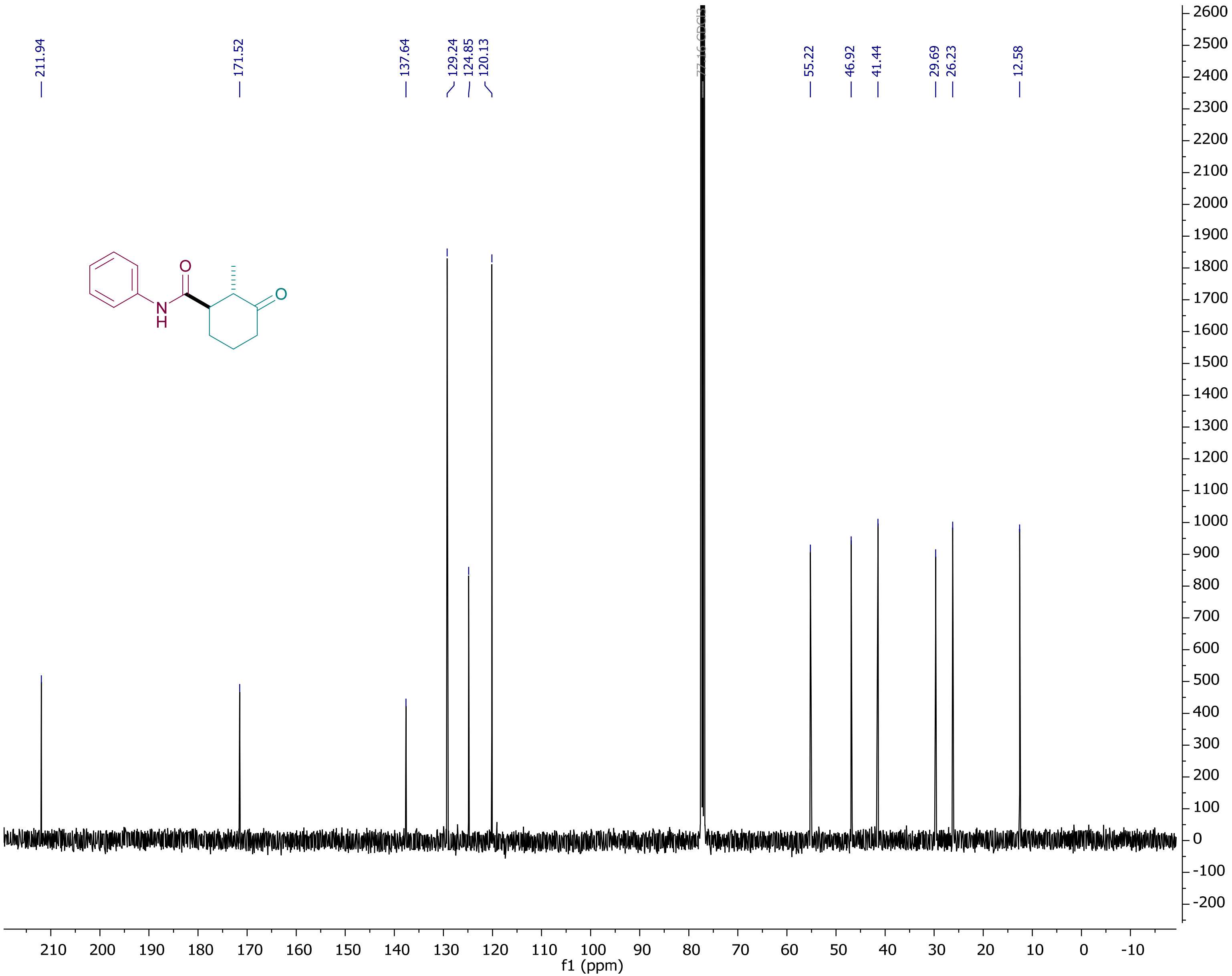

Compound 9ae (Minor Diastereomer) – ^{1}H NMR (400 MHz, Chloroform-*d*):

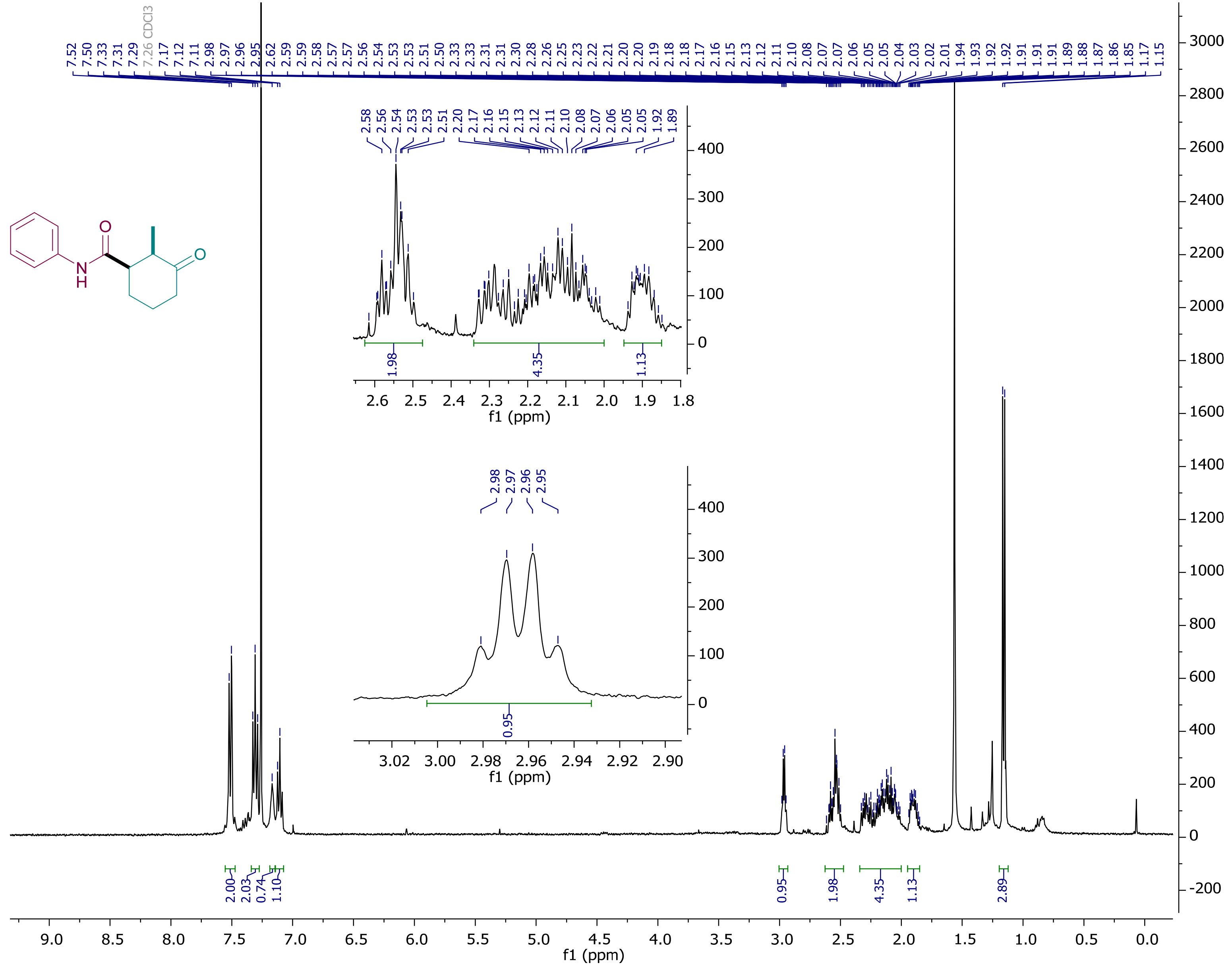

Compound 9ae (Minor Diastereomer) – ^{13}C NMR (101 MHz, Chloroform-d):

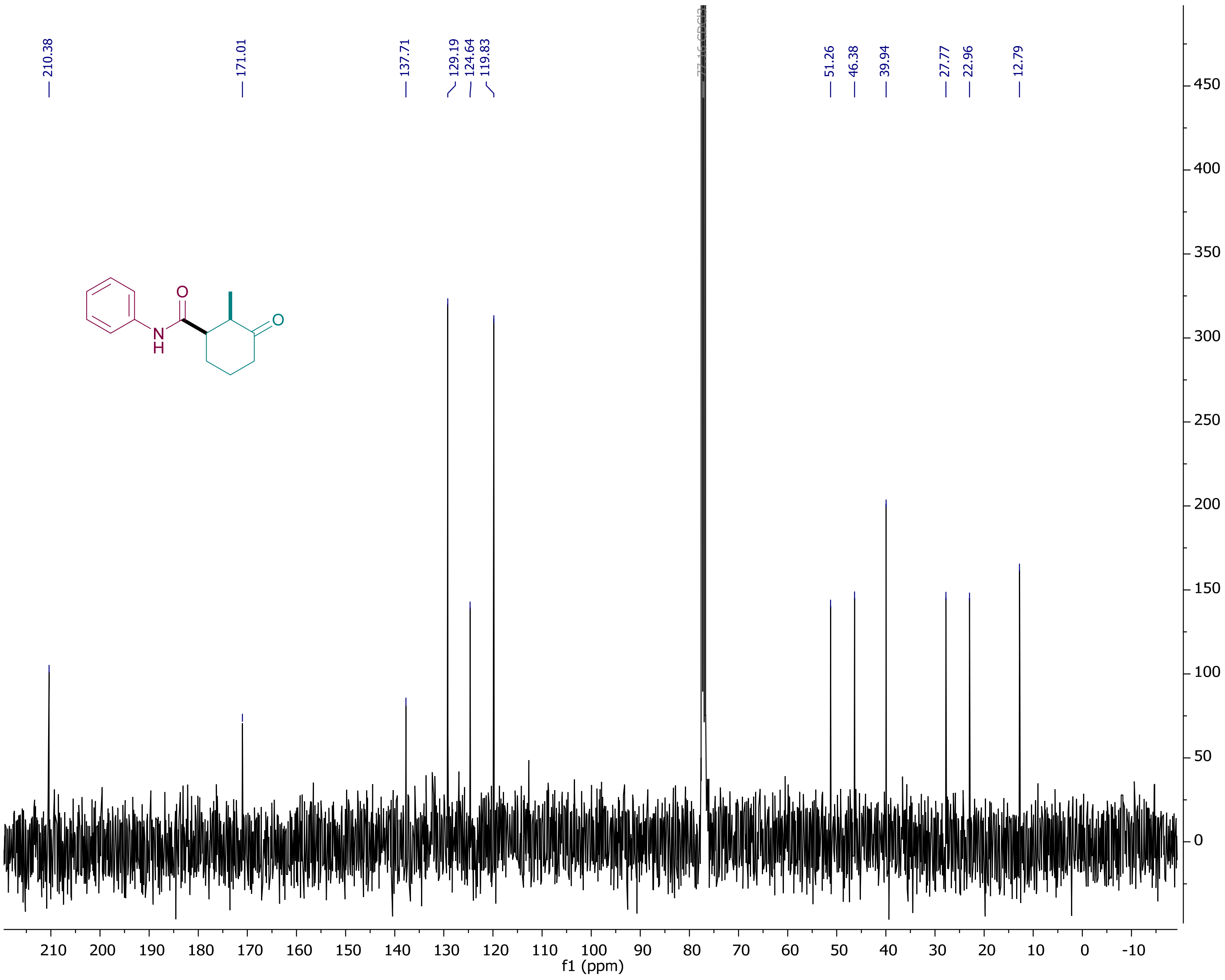

(5*R*)-2-Methyl-3-oxo-5-(prop-1-en-2-yl)cyclohexane-1-carboxamide (9af)

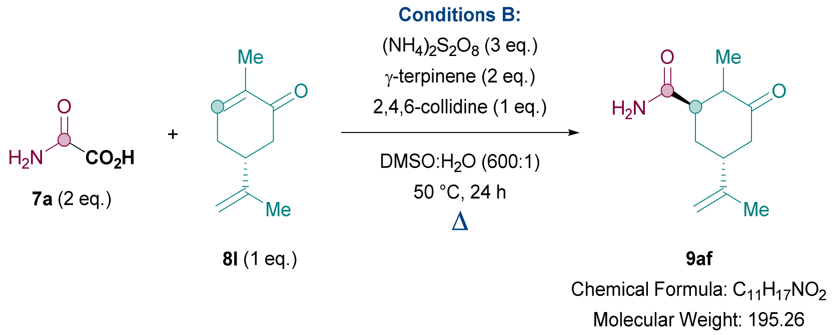

General procedure for conditions B was followed. *R*-(-)-carvone **8l** (18.0 mg, 0.12 mmol, 1.0 eq.), oxamic acid **7a** (21.5 mg, 0.24 mmol, 2.0 eq), $(NH_4)_2S_2O_8$ (82.4 mg, 0.36 mmol, 3.0 eq.), γ-terpinene (38.5 µL, 0.24 mmol, 2.0 eq.), and 2,4,6-collidine (15.9 µL, 0.12 mmol, 1.0 eq.) in DMSO:H_2O (600:1) (0.4 mL) was heated at 50 °C for 24 h followed by work-up B. The crude was then purified *via* column flash chromatography eluting with 25:75→20:80 hexane:EtOAc. The solvent was then removed *in vacuo* to afford a 50:50 diastereomeric mixture of methyl (5*R*)-2-methyl-3-oxo-5-(prop-1-en-2-yl)cyclohexane-1-carboxamide **9af** as a white solid (7.3 mg, 0.037 mmol, 31%). Data consistent with literature data.[8]

Characterisation:

^{1}H NMR (300 MHz, Chloroform-*d*): (50:50 mixture of diastereomers) δ ppm 5.76 – 5.43 (br m, 2H, NH, both diastereomers), 4.91 – 4.87 (m, 0.5H, =C<u>H</u>H), 4.79 – 4.77 (m, 0.5H, =C<u>H</u>’H’), 4.74 (s, 0.5H, =CH’<u>H</u>’), 4.68 (s, 0.5 H, =CH<u>H</u>), 2.99 – 2.93 (m, 0.5H, CH), 2.93 – 2.76 (m, 1H, CH, CH’), 2.72 – 2.64 (m, 0.5H, CH), 2.63 – 2.49 (m, 1.5H, C<u>H</u>H, CH'_2), 2.47 – 2.32 (m, 0.5H, CH’), 2.28 (td, *J* = 10.2 Hz, 4.0 Hz, 0.5H, CH’), 2.23 – 2.07 (m, 2H, CH<u>H</u>, C<u>H</u>H, CH'_2), 2.01 – 1.89 (m, 0.5H, CH<u>H</u>), 1.75 (s, 1.5H, =CH_3), 1.73 (s, 1.5H, =CH'_3), 1.11 (d, *J* = 6.8 Hz, 1.5H, CH_3), 1.05 (d, *J* = 6.6 Hz, 1.5H, CH'_3). **^{13}C NMR (101 MHz, Chloroform-*d*):** (50:50 mixture of diastereomers) δ ppm 211.5 (C), 209.6 (C), 175.9 (C), 175.2 (C), 147.4 (C), 146.3 (C), 113.0 (C), 110.2 (C), 48.8 (CH_2), 47.6 (CH_2), 46.3 (CH), 45.5 (CH), 45.4 (CH), 44.2 (CH), 41.1 (CH_2), 40.8 (CH_2), 33.5 (CH), 31.3 (CH), 22.3 (CH_3), 20.8 (CH_3), 13.0 (CH_3), 12.4 (CH_3). **IR:** ν_{max}/cm^{-1} 3328, 2952, 2872, 1720, 1678, 1597, 1538, 1489, 1439, 1372, 1355, 1308, 1298, 1244, 1215, 1193, 1180. **HRMS (ESI-TOF):** *m/z* $[M + H]^+$ calcd for $C_{11}H_{18}NO_2$, 196.13321; found 196.1334. **TLC:** R_f = 0.34 (20:80 hexane:EtOAc). **m.p.** 55-59 °C.

Compound 9af – ^{1}H NMR (300 MHz, Chloroform-*d*): (50:50 mixture of diastereomers)

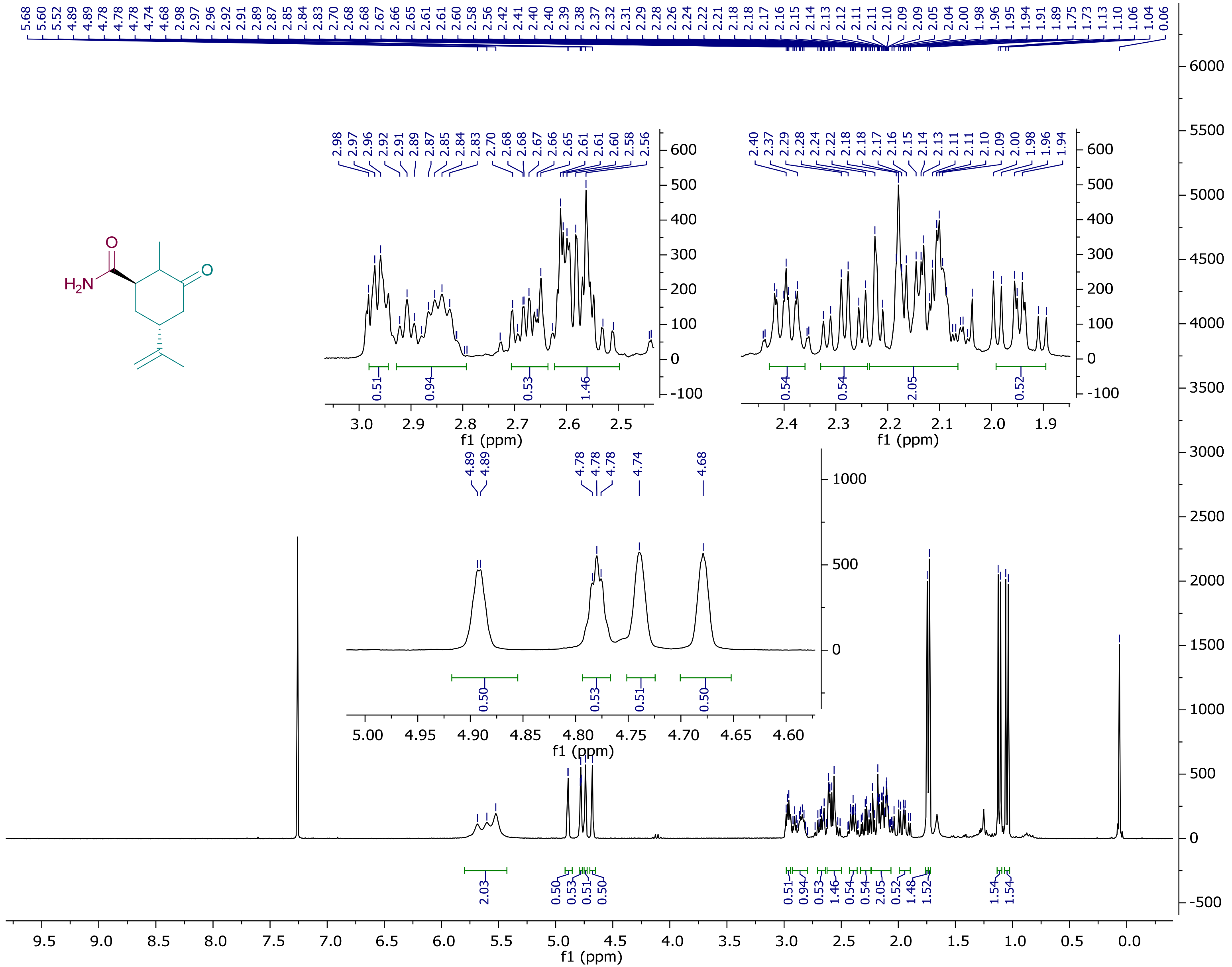

Compound 9af – ^{13}C NMR (101 MHz, Chloroform-*d*): (50:50 mixture of diastereomers)

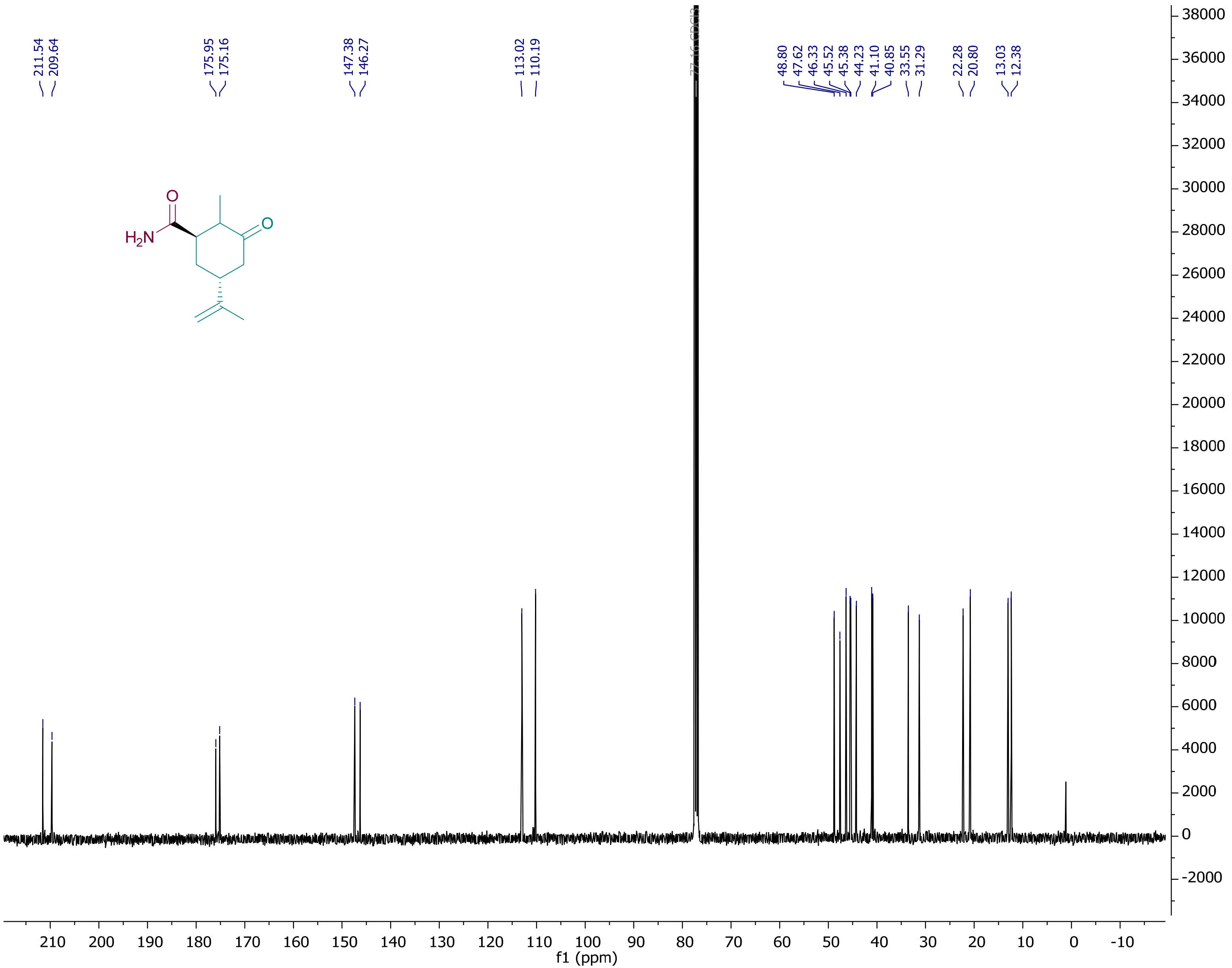

Methyl 2-(phenylcarbamoyl)cyclopentane-1-carboxylate (9ag)

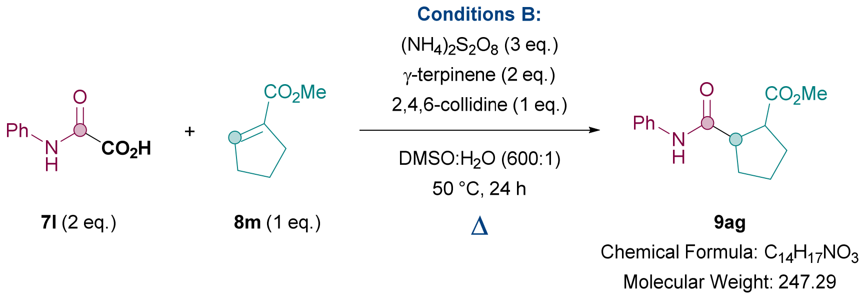

General procedure for Conditions B was followed. Methyl cyclopent-1-enecarboxylate **8m** (15.1 mg, 0.12 mmol, 1.0 eq.), *N*-(phenyl)-oxoacetic acid **7l** (39.7 mg, 0.24 mmol, 2.0 eq.), $(NH_4)_2S_2O_8$ (82.2 mg, 0.36 mmol, 3.0 eq.), γ-terpinene (38.5 µL, 0.24 mmol, 2.0 eq.), and 2,4,6-collidine (15.9 µL, 0.12 mmol, 1.0 eq.) in DMSO:H_2O (600:1) (0.4 mL) was heated at 50 °C for 24 h followed by work-up Y. The crude was then purified *via* column flash chromatography eluting with 80:20→60:40 hexane:EtOAc. The solvent was then removed *in vacuo* to afford methyl 2-(phenylcarbamoyl)cyclopentane-1-carboxylate **9ag** as 71:29 mixture of diastereomers that were separable *via* column chromatography. The major diastereomer was isolated as a pale-yellow solid (12.2 mg, 0.021 mmol, 41%) and the minor diastereomer was isolated as a pale-yellow oil (5.1 mg, 0.049 mmol, 17%).

Characterisation for Major Diastereomer:

^{1}H NMR (300 MHz, Chloroform-*d*): δ ppm 7.48 (d, *J* = 8.0 Hz, 2H, ArH), 7.36 (br s, 1H, NH), 7.29 (t, *J* = 7.4 Hz, 2H, ArH), 7.08 (t, *J* = 7.4 Hz, 1H, ArH), 3.62 (s, 3H, OCH_3), 3.14 – 2.99 (m, 2H, CH, CH), 2.24 – 1.88 (m, 4H, 2 x CH_2, C<u>H</u>H), 1.78 – 1.58 (m, 1H, CH<u>H</u>). **^{13}C NMR (101 MHz, Chloroform-*d*):** δ ppm 174.6 (C), 172.1 (C), 138.1 (C), 129.1 (CH), 124.3 (CH), 120.0 (CH), 52.0 (CH_3), 49.6 (CH), 47.8 (CH), 29.7 (CH_2), 28.4 (CH_2), 24.4 (CH_2). **IR:** ν_{max}/cm^{-1} 3328, 2951, 2872, 1720, 1679, 1597, 1537, 1488, 1439, 1355, 1298, 1244, 1193. **HRMS (ESI-TOF):** *m*/*z* $[M + H]^+$ calcd for $C_{14}H_{18}NO_3$, 248.12812; found 248.1275. **TLC:** R_f = 0.20 (70:30 hexane:EtOAc). **m.p.** 79-83 °C.

Characterisation of Minor Diastereomer:

^{1}H NMR (400 MHz, Chloroform-*d*): δ ppm 7.90 (br s, 1H, NH), 7.53 (d, *J* = 7.6 Hz, 2H, ArH), 7.36 – 7.27 (t, *J* = 8.0 Hz, 2H, ArH), 7.08 (t, *J* = 7.4 Hz, 1H, ArH), 3.74 (s, 3H, OCH_3), 3.10 (q, *J* = 8.6 Hz, 1H, CH), 3.04 (q, *J* = 8.3 Hz, 1H, CH), 2.22 – 2.06 (m, 2H, C$\underline{H}$H, C$\underline{H}$H), 2.04 – 1.91 (m, 1H, CH$\underline{H}$), 1.92 – 1.69 (m, 3H, CH$\underline{H}$, CH_2). **^{13}C NMR (101 MHz, Chloroform-*d*):** δ ppm 176.6 (C), 172.4 (C), 138.3 (C), 129.1 (CH), 124.2 (CH), 119.7 (CH), 52.4 (CH_3), 49.2 (CH), 48.5 (CH), 30.8 (CH_2), 29.5 (CH_2), 25.5 (CH_2). **IR:** ν_{max}/cm^{-1} 3306, 3139, 2952, 2872, 1731, 1660, 1598, 1541, 1500, 1441, 1386, 1312, 1247, 1202, 1174. **HRMS (ESI-TOF):** *m*/*z* $[M + H]^+$ calcd for $C_{14}H_{18}NO_3$, 248.12812; found 248.1278. **TLC:** R_f = 0.18 (80:20 hexane:EtOAc).

Compound 9ag (Major Diastereomer) – ^{1}H NMR (300 MHz, Chloroform-*d*):

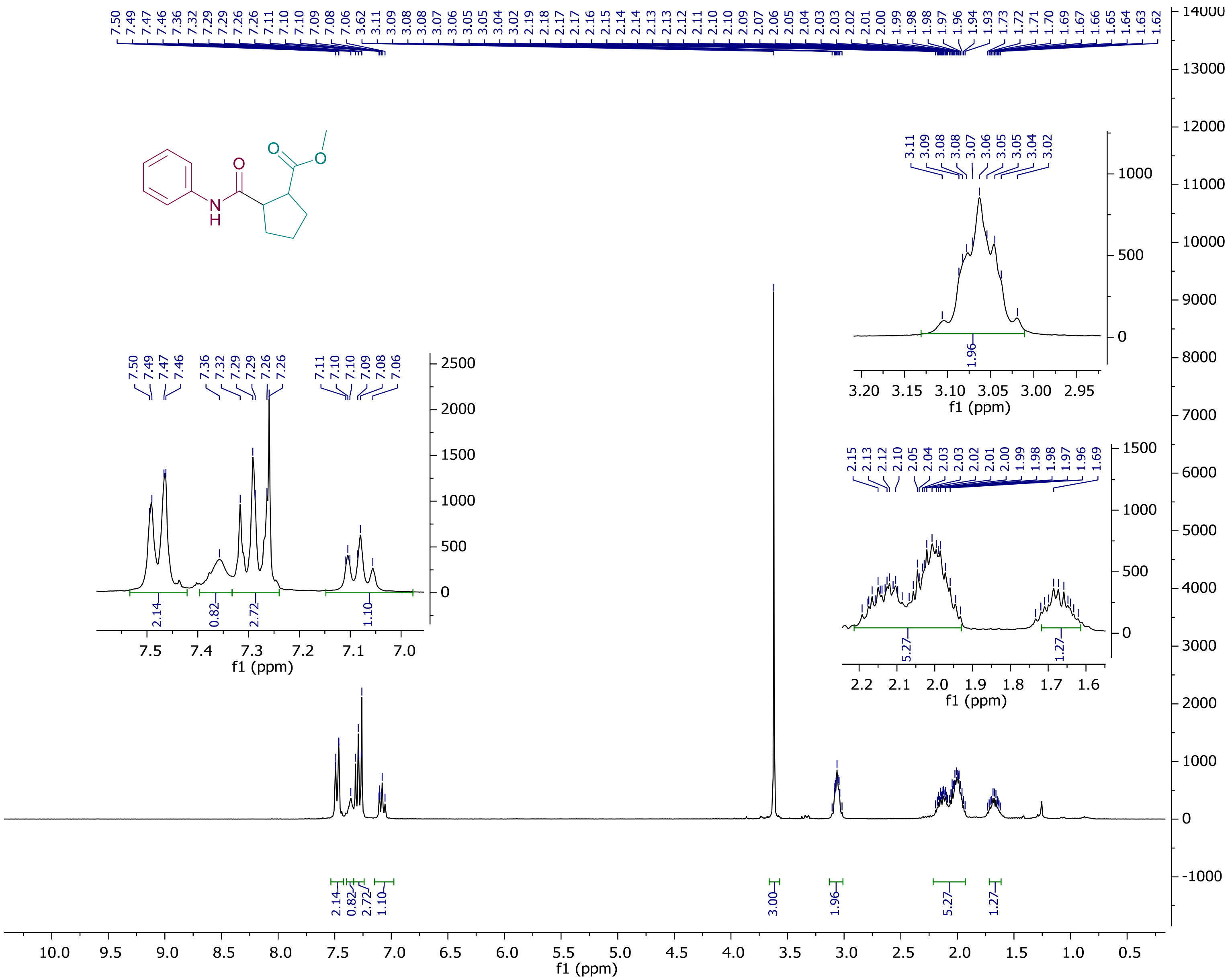

Compound 9ag (Major Diastereomer) – ^{13}C NMR (101 MHz, Chloroform-*d*):

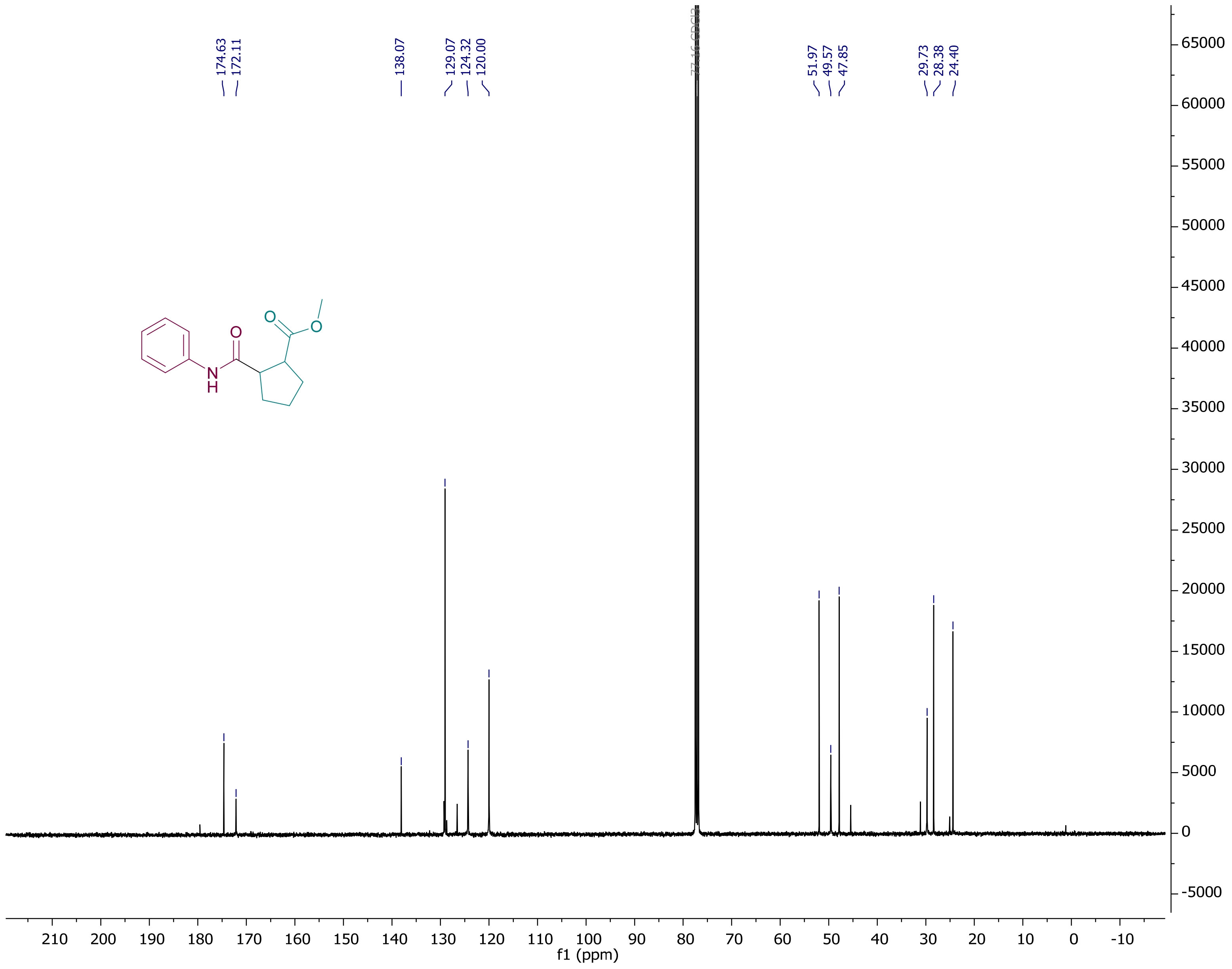

Compound 9ag (Minor Diastereomer) – ^{1}H NMR (400 MHz, Chloroform-d):

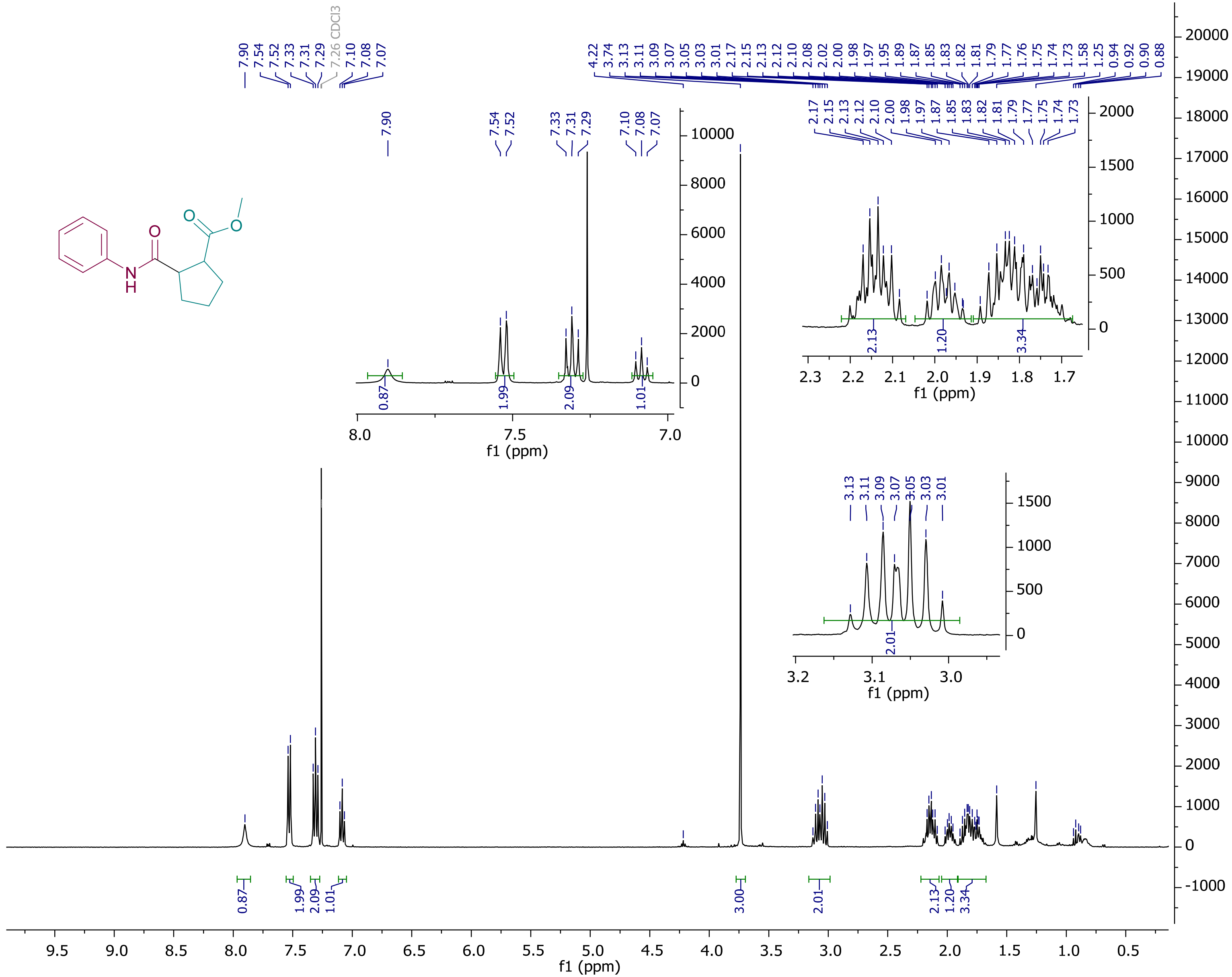

Compound 9ag (Minor Diastereomer) – ^{13}C NMR (101 MHz, Chloroform-*d*):

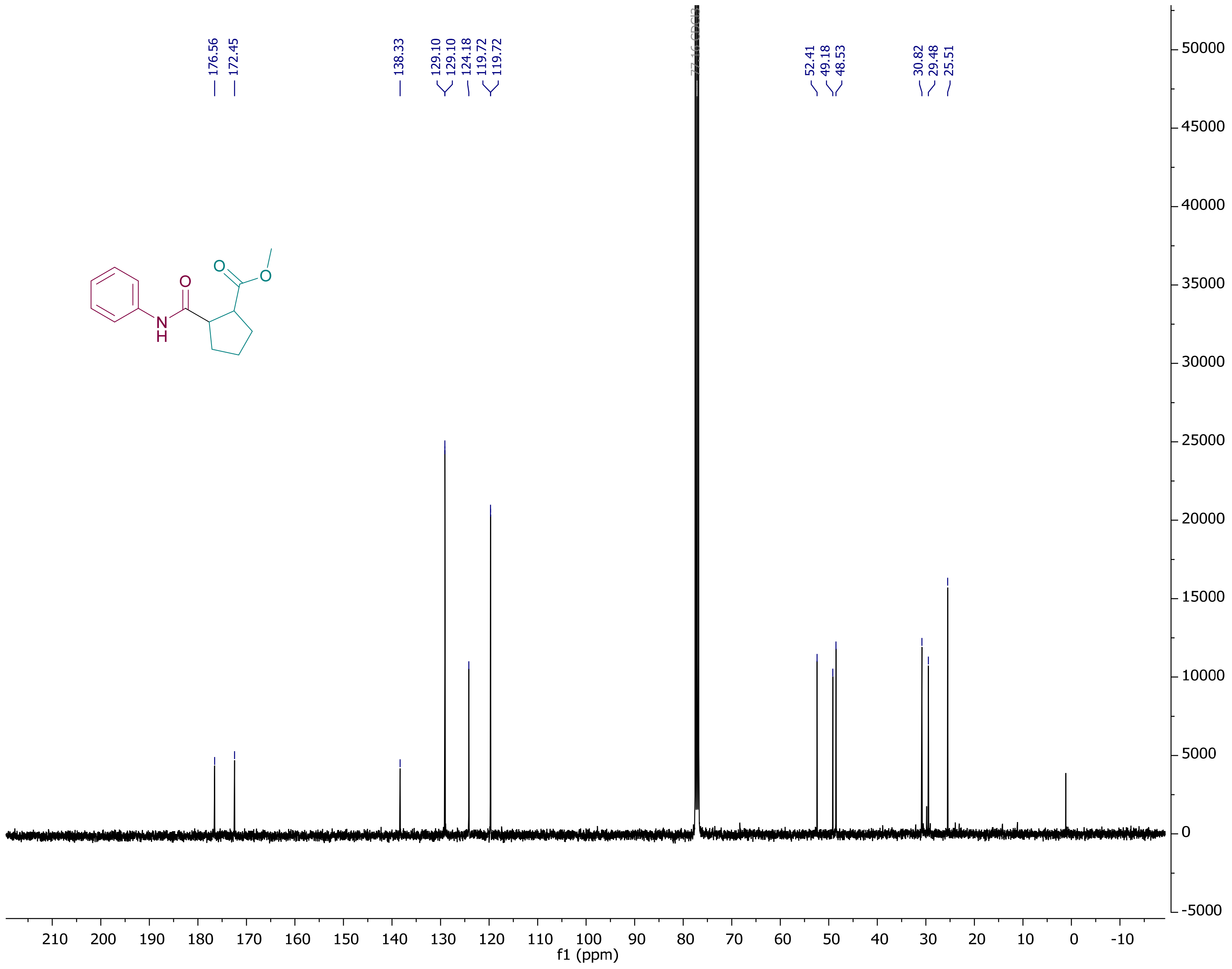

Methyl 2-(phenylcarbamoyl)cyclohexane-1-carboxylate (9ah)

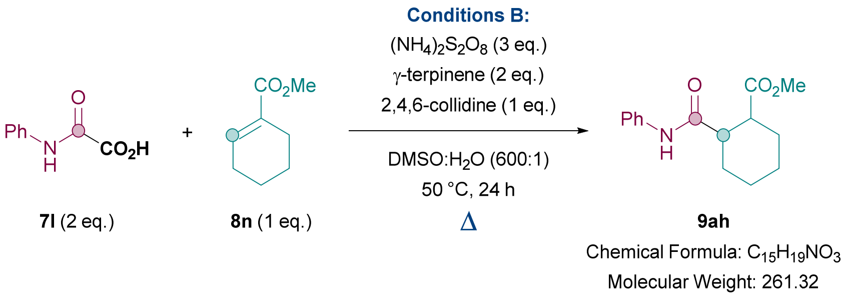

General procedure for Conditions B was followed. Methyl cyclohex-1-enecarboxylate **8n** (17.0 mg, 0.12 mmol, 1.0 eq.), *N* *N*-(phenyl)-oxoacetic acid **7l** (39.8 mg, 0.24 mmol, 2.0 eq.), $(NH_4)_2S_2O_8$ (82.2 mg, 0.36 mmol, 3.0 eq.), γ-terpinene (38.5 µL, 0.24 mmol, 2.0 eq.), and 2,4,6-collidine (15.9 µL, 0.12 mmol, 1.0 eq.) in DMSO:H_2O (600:1) (0.4 mL) was heated at 50 °C for 24 h followed by work-up Y. The crude was then purified *via* column flash chromatography eluting with 75:25→50:50 hexane:EtOAc. The solvent was then removed *in vacuo* to afford a 81:19 diastereomeric mixture of methyl 2-(phenylcarbamoyl)cyclohexane-1-carboxylate **9ah** as a pale yellow solid (17.4 mg, 0.071 mmol, 55%).

Characterisation:

^{1}H NMR (300 MHz, Chloroform-*d*): (81:19 mixture of diastereomers) δ ppm 7.59 (br s, 0.8H, NH, major), 7.49 (d, *J* = 7.3 Hz, 2H, ArH), 7.43 (br s, 0.2H, NH minor), 7.30 (t, *J* = 8.0 Hz, 2H, ArH), 7.08 (t, *J* = 7.4 Hz, 1H, ArH), 3.67 (s, 2.4H, OCH_3 major), 3.64 (s, 0.6H, OCH_3 minor), 2.95 (dt, *J* = 8.0, 4.4 Hz, 0.8H, CH major), 2.80 (dt, *J* = 8.3, 4.1 Hz, 0.8H, CH major), 2.76 (td, *J* = 11.7 Hz, 3.8 Hz, 0.2 H, CH minor), 2.51 (td, *J* = 11.8, 3.7 Hz, 0.2H, CH minor), 2.25 – 2.07 (m, 1.8H, 2 x C<u>H</u>H major, C<u>H</u>H minor), 2.03 – 1.94 (m, 0.2H, C<u>H</u>H minor)1.90 – 1.72 (m, 2.8H, C<u>H</u>H major, 2 x CH<u>H</u> major, 2 x C<u>H</u>H minor), 1.68 – 1.61 (m, 0.2H, CH<u>H</u> minor), 1.61 – 1.39 (m, 2.4H, CH<u>H</u> major, CH_2 major), 1.39 – 1.28 (m, 0.6H, 3 x CH<u>H</u> minor). **^{13}C NMR (101 MHz, Chloroform-*d*):** (81:19 mixture of diastereomers) δ ppm 176.2 (C), 174.9 (C), 173.2 (C), 172.2 (C), 138.1 (CH plus one overlapping CH), 129.0 (CH plus one overlapping CH), 124.3 (CH), 124.2 (CH), 120.2 (CH), 119.9 (CH), 52.0 (CH_3), 51.9 (CH_3), 47.8 (CH), 45.3 (CH), 45.2 (CH), 42.9 (CH), 29.6 (CH_2), 29.2 (CH_2), 27.0 (CH_2 plus one overlapping CH_2), 25.3 (CH_2), 25.2 (CH_2), 23.9 (CH_2), 23.6 (CH_2). **IR:** ν_{max}/cm^{-1} 3318, 2933, 2856, 1732, 1662, 1598, 1538, 1500, 1439, 1385, 1308, 1247, 1195. **HRMS (ESI-TOF):** *m*/*z*

$[M + H]^+$ calcd for $C_{15}H_{20}NO_3$, 262.14377; found 262.1441. **TLC:** R_f = 0.23 (60:40 hexane:EtOAc). **M.p.** 73-77 °C.

Assignment of diastereomers by coupling constants:

The major diastereomer is assigned as *syn*, based on the multiplicities and *J* values of the CH peaks. For example, the CH peak at δ 2.80 ppm is a dt with *J* = 8.3, 4.1 Hz. The *syn* isomer is expected to ring flip more readily than the *anti* isomer, giving a large *J* value of 8.3 Hz due to the averaging of the $^3J_{axial-axial}$ (approx. 12 Hz) and the $^3J_{axial-equatorial}$ (approx. 4 Hz).

The minor diastereomer is assigned as *anti*, based on the multiplicities and *J* values of the CH peaks. For example, the CH peak at δ 2.51 ppm is a td with *J* = 11.8, 3.7 Hz, The large 11.8 Hz is only consistent with a $^3J_{axial-axial}$, indicating the *anti* isomer. (The most stable conformer for the *anti* isomer is expected to be the one with both substituents equatorial.)

Compound 9ah – ^{1}H NMR (300 MHz, Chloroform-*d*): (81:19 mixture of diastereomers)

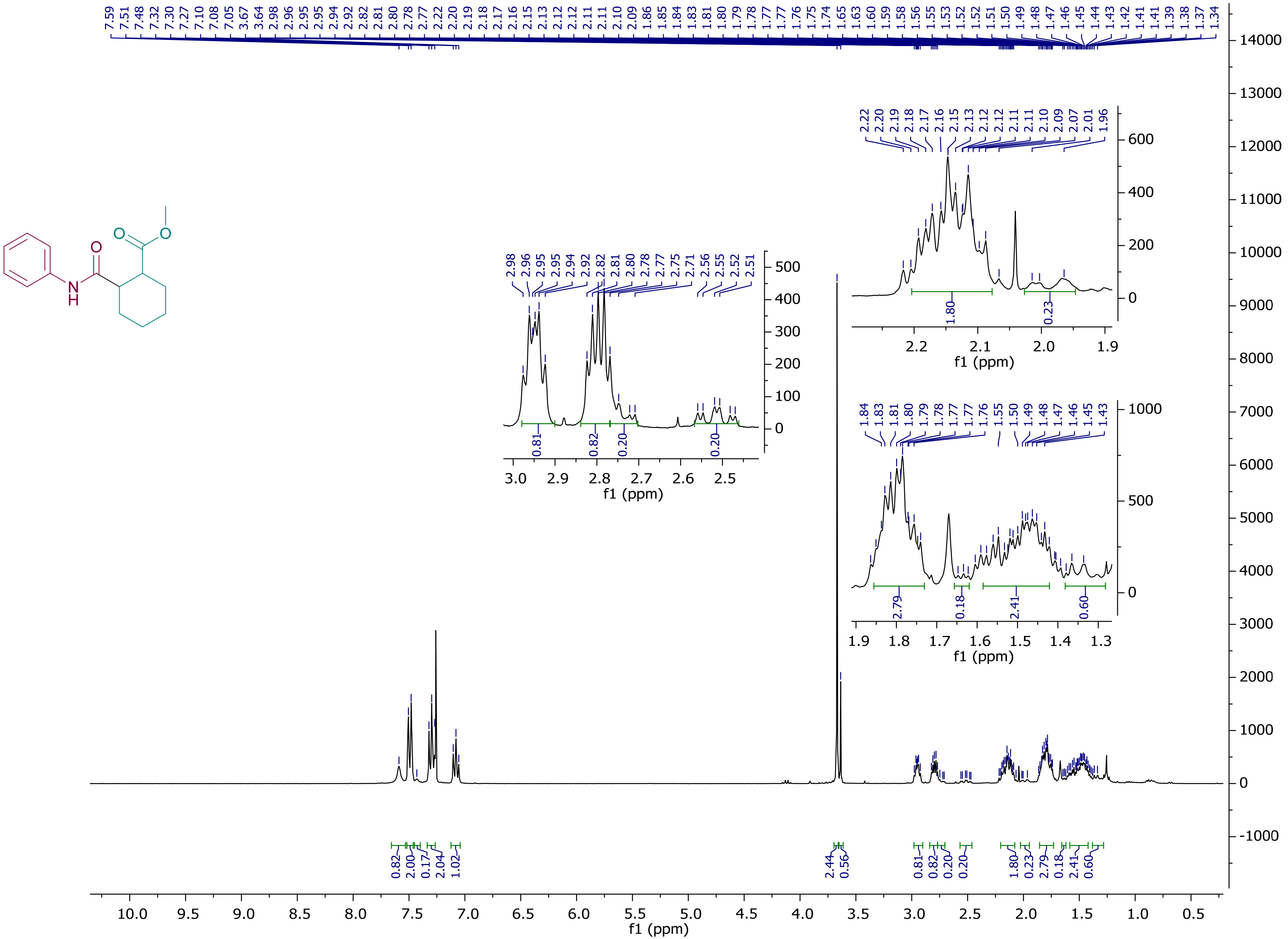

Compound 9ah – ^{13}C NMR (101 MHz, Chloroform-*d*): (81:19 mixture of diastereomers)

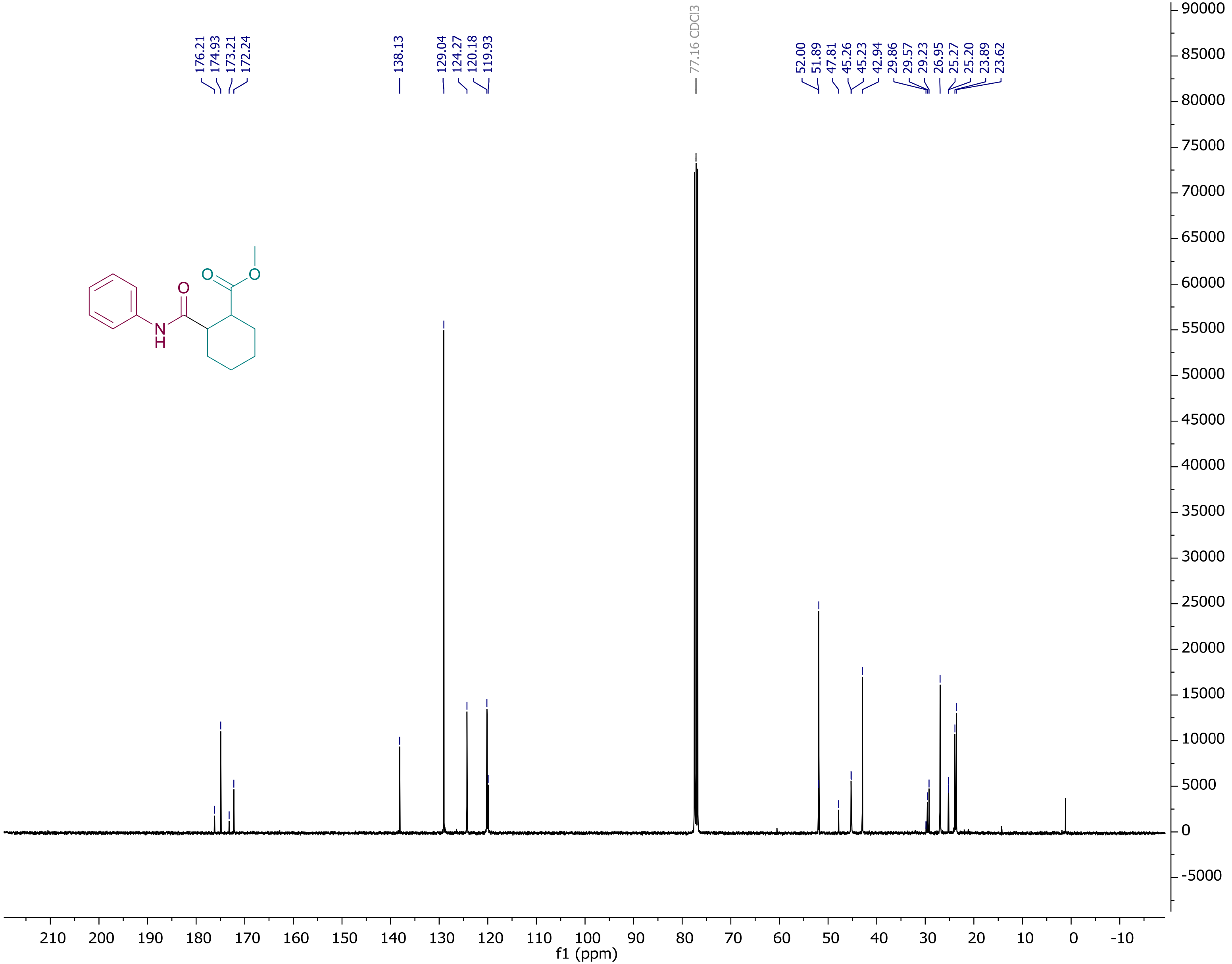

Diethyl 2-(phenylcarbamoyl)succinate (9ai)

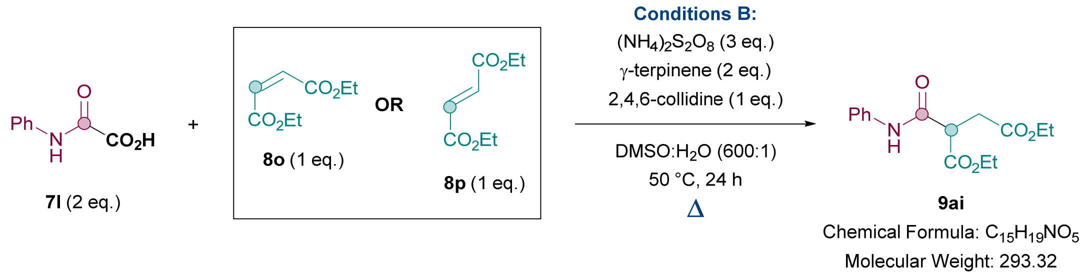

Using diethyl maleate 8o: General procedure for Conditions B was followed. Diethyl maleate **8o** (20.6 mg, 0.12 mmol, 1.0 eq.), *N*-(phenyl)-oxoacetic acid **7l** (39.8 mg, 0.24 mmol, 2.0 eq.), $(NH_4)_2S_2O_8$ (82.3 mg, 0.36 mmol, 3.0 eq.), γ-terpinene (38.5 μL, 0.24 mmol, 2.0 eq.), and 2,4,6-collidine (15.9 μL, 0.12 mmol, 1.0 eq.) in DMSO:H_2O (600:1) (0.4 mL) was heated at 50 °C for 24 h followed by work-up Y. The crude was then purified *via* column flash chromatography eluting with 70:30 hexane:EtOAc. The solvent was then removed *in vacuo* to afford diethyl 2-(phenylcarbamoyl)succinate **9ai** as off-white solid (31.3 mg, 0.11 mmol, 89%).

Using diethyl fumarate 8p: General procedure for Conditions B was followed. Diethyl fumarate **8p** (20.7 mg, 0.12 mmol, 1.0 eq.), *N*-phenyl-oxoacetic acid **7l** (39.7 mg, 0.24 mmol, 2.0 eq.), $(NH_4)_2S_2O_8$ (82.2 mg, 0.36 mmol, 3.0 eq.), γ-terpinene (38.5 μL, 0.24 mmol, 2.0 eq.), and 2,4,6-collidine (15.9 μL, 0.12 mmol, 1.0 eq.) in DMSO:H_2O (600:1) (0.4 mL) was heated at 50 °C for 24 h followed by work-up Y. The crude was then purified *via* column flash chromatography eluting with 70:30 hexane:EtOAc. The solvent was then removed *in vacuo* to afford diethyl 2-(phenylcarbamoyl)succinate **9ai** as an off-white solid (27.3 mg, 0.092 mmol, 77%).

Characterisation:

^{1}H NMR (300 MHz, Chloroform-*d*): δ ppm 8.61 (br s, 1H, NH), 7.51 (d, *J* = 7.6 Hz, 2H, ArH), 7.31 (t, *J* = 7.6 Hz, 2H, ArH), 7.11 (t, *J* = 7.4 Hz, 1H, ArH), 4.25 (q, *J* = 7.1 Hz, 2H, OCH_2), 4.16 (q, *J* = 7.2 Hz, 3H, OCH_2), 3.83 (t, *J* = 6.7 Hz, 1H, CH), 3.07 (d, *J* = 6.7 Hz, 2H, CH_2), 1.29 (t, *J* = 7.1 Hz, 3H, CH_3), 1.25 (t, *J* = 7.2 Hz, 3H, CH_3). **^{13}C NMR (101 MHz, Chloroform-*d*):** δ ppm 171.8 (C), 169.9 (C), 164.9 (C), 137.7 (C), 129.1 (CH), 124.7 (CH), 120.1 (CH), 62.3 (CH_2), 61.2 (CH_2), 49.0 (CH), 32.8 (CH_2), 14.2 (CH_3), 14.1 (CH_3). **IR:**

ν_{max}/cm^{-1} 3259, 3199, 3141, 3084, 2983, 1728, 1648, 1598, 1549, 1500, 1447, 1323, 1288. **HRMS (ESI-TOF):** *m/z* [M + H]$^+$ calcd for $C_{15}H_{20}NO_5$, 294.1334; found, 294.13360. **TLC:** R_f = 0.28 (70:30 hexane:EtOAc). **m.p.** 85-88 °C.

Compound 9ai – ¹H NMR (300 MHz, Chloroform-*d*):

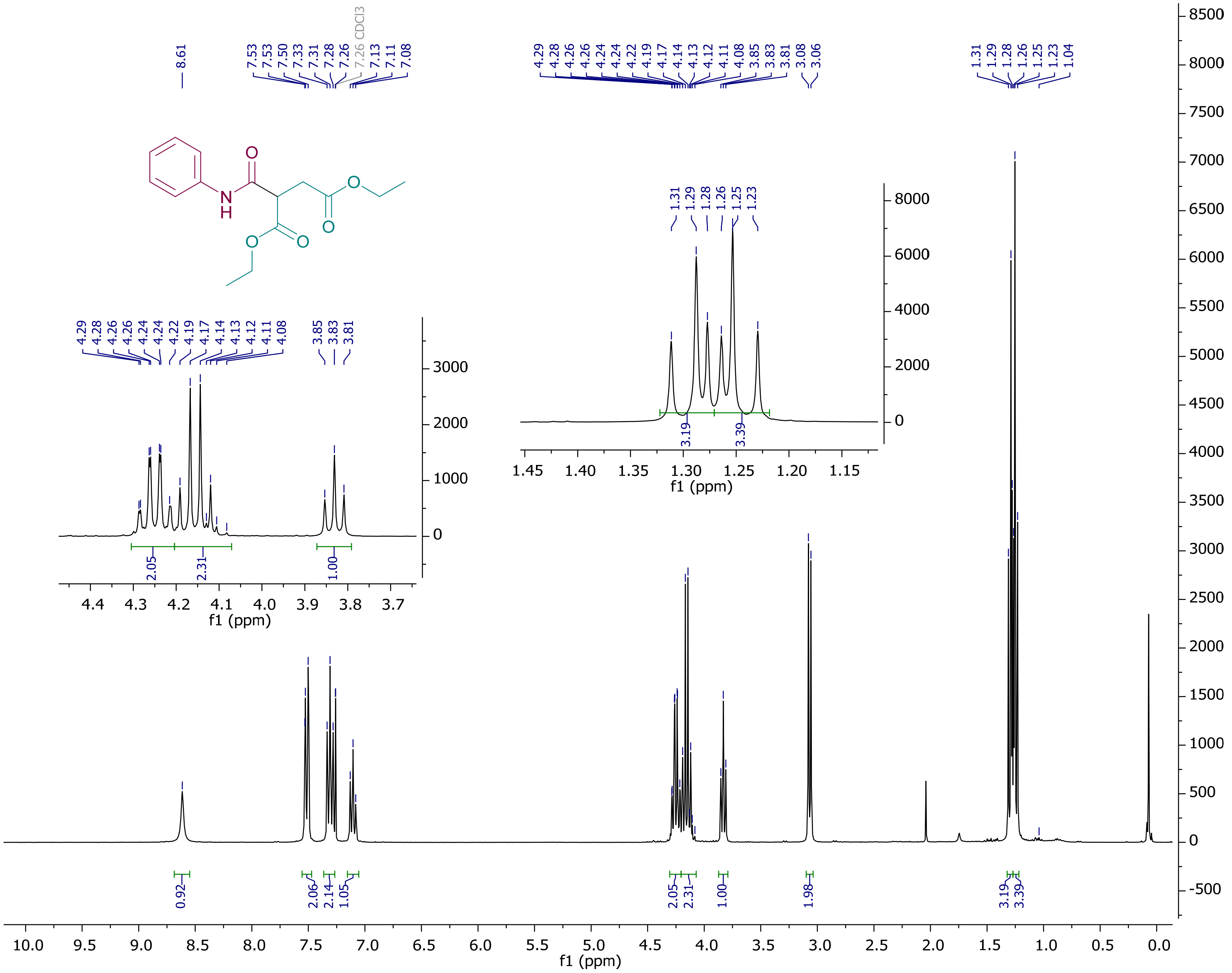

Compound 9ai – ^{13}C NMR (101 MHz, Chloroform-*d*):

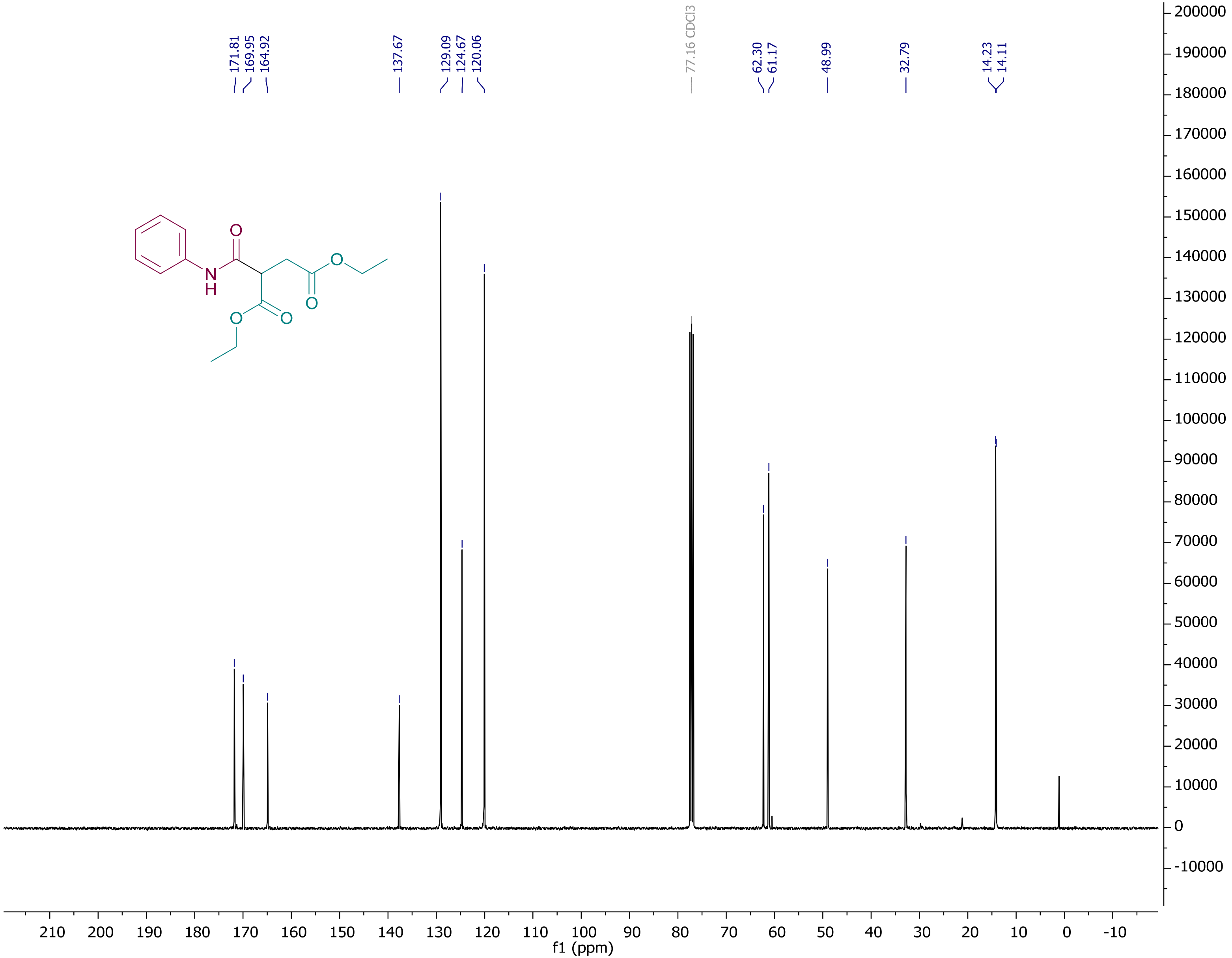

Diethyl (3-oxo-3-(phenylamino)propyl)phosphonate (9aj)

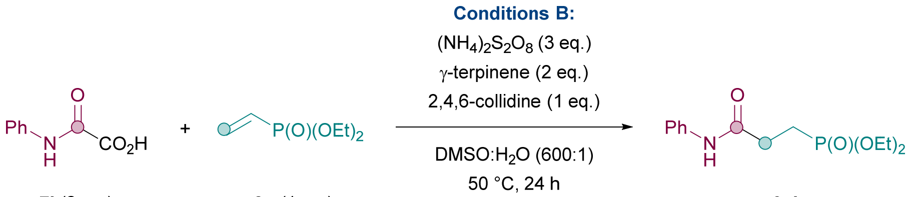

7l (2 eq.) **8q** (1 eq.) **9aj**

Chemical Formula: $C_{13}H_{20}NO_4P$

Molecular Weight: 285.28

General procedure for Conditons B was followed. Diethyl vinylphosphonate **8q** (18.8 mg, 0.12 mmol, 1.0 eq.), *N*-(phenyl)-oxoacetic acid **7l** (40.3 mg, 0.24 mmol, 2.0 eq.), $(NH_4)_2S_2O_8$ (82.6 mg, 0.36 mmol, 3.0 eq.), γ-terpinene (38.5 µL, 0.24 mmol, 2 eq.), and 2,4,6-collidine (15.9 µL, 0.12 mmol, 1.0 eq.) in DMSO:H_2O (600:1) (0.4 mL) was heated at 50 ºC for 24 h followed by work-up X. The crude was then purified *via* column flash chromatography eluting with 95:5 CH_2Cl_2:MeOH. The solvent was then removed *in vacuo* to afford diethyl (3-oxo-3-(phenylamino)propyl)phosphonate **9aj** as a red/brown oil (21.6 mg, 0.077 mmol, 64%).

Characterisation:

^{1}H NMR (300 MHz, Chloroform-*d*): δ ppm 9.23 (br s, 1H, NH), 7.60 (d, J = 7.4 Hz, 2H, ArH), 7.28 (t, 2H, J = 8.0 Hz), 7.06 (t, J = 7.4 Hz, 1H, ArH), 4.19 – 4.01 (m, 4H, 2 x OCH_2), 2.81 – 2.65 (m, 2H, CH_2), 2.27 – 2.10 (m, 2H, CH_2), 1.31 (t, J = 7.1 Hz, 6H, 2 x CH_3). **^{13}C NMR (75 MHz, Chloroform-*d*):** δ ppm 169.6 (d, J_{C-P} = 16.0 Hz, C), 138.8 (C), 128.9 (CH), 123.9 (CH), 119.8 (CH), 62.2 (d, J_{C-P} = 6.6 Hz, CH_2), 30.0 (d, J_{C-P} = 3.5 Hz, CH_2), 21.0 (d, J_{C-P} = 143.4 Hz, CH_2), 16.5 (d, J_{C-P} = 6.0 Hz, CH_3). **^{31}P NMR (121 MHz, Chloroform-*d*):** δ ppm 31.52 **IR:** ν_{max}/cm^{-1} 3263, 3197, 3134, 3083, 2982, 2929, 1690, 1599, 1549, 1499, 1443, 1310, 1218. **HRMS (ESI-TOF):** *m*/*z* $[M + H]^+$ calcd for $C_{13}H_{21}NO_4P$, 286.12027; found, 286.1211. **TLC:** R_f = 0.16 (95:5 CH_2Cl_2:MeOH).

Compound 9aj – ¹H NMR (300 MHz, Chloroform-*d*):

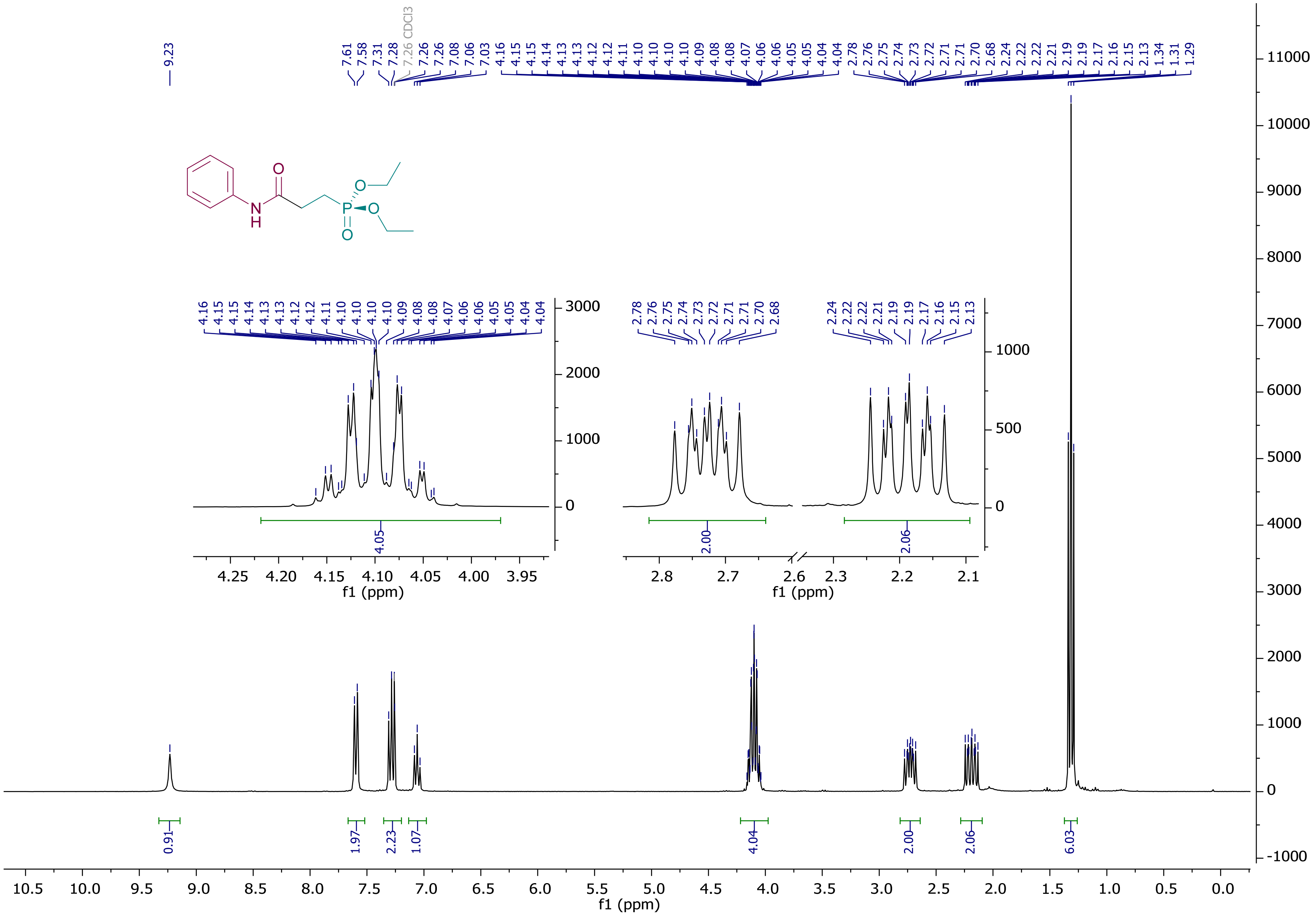

Compound 9aj – ^{13}C NMR (75 MHz, Chloroform-*d*):

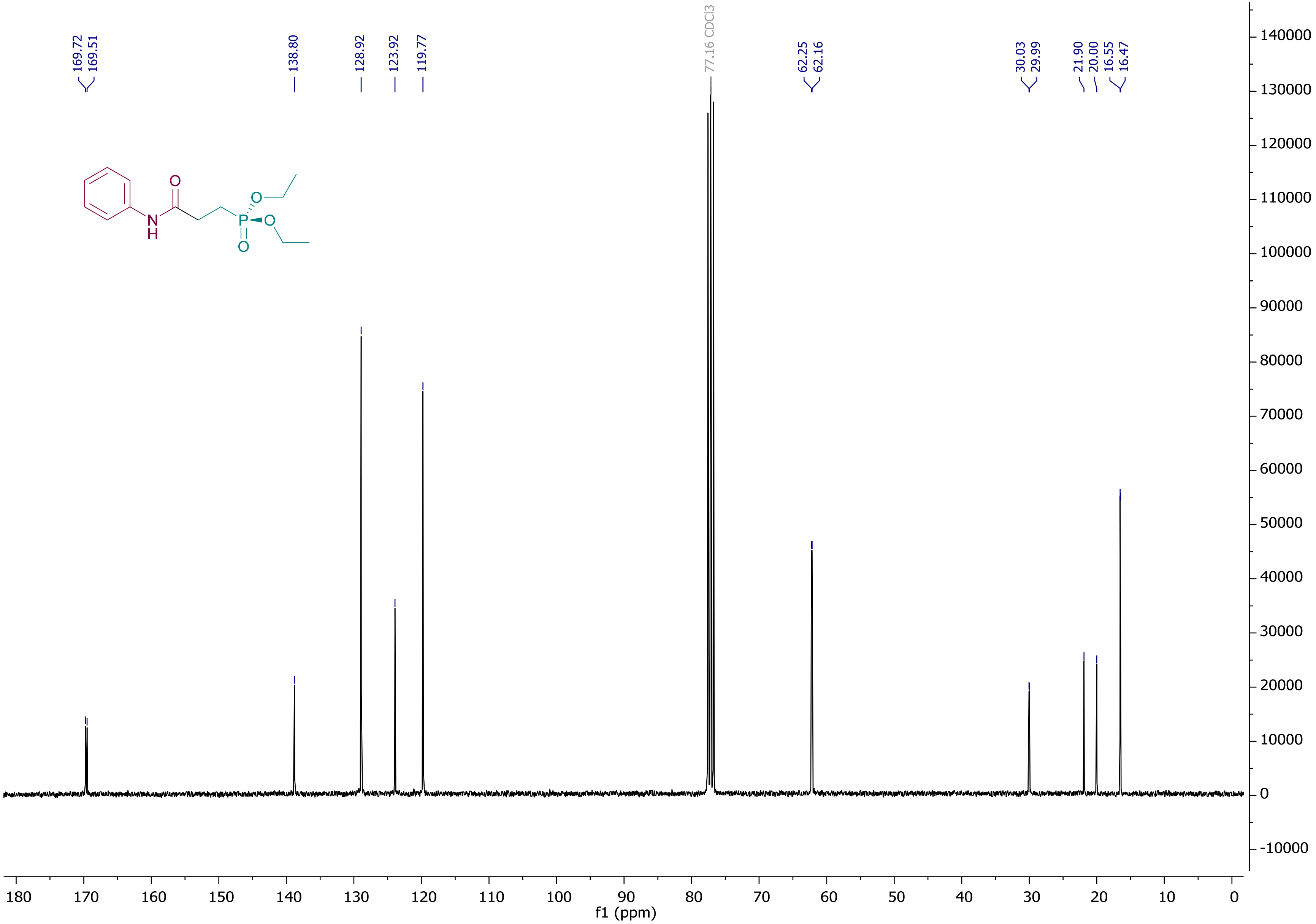

Compound 9aj – ^{31}P NMR (121 MHz, Chloroform-*d*):

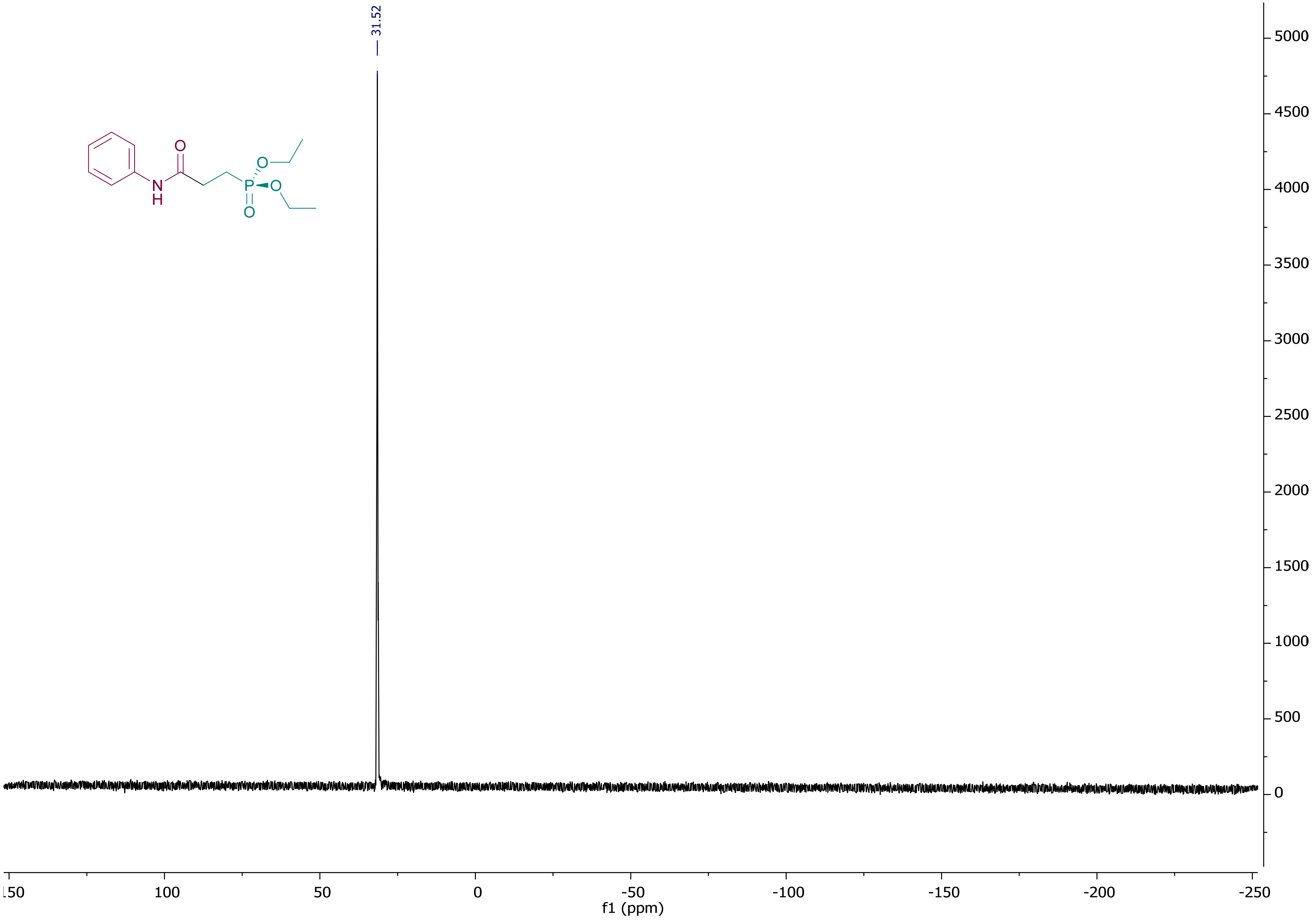

3-(Methylsulfonyl)-*N*-phenylpropanamide (9ak)

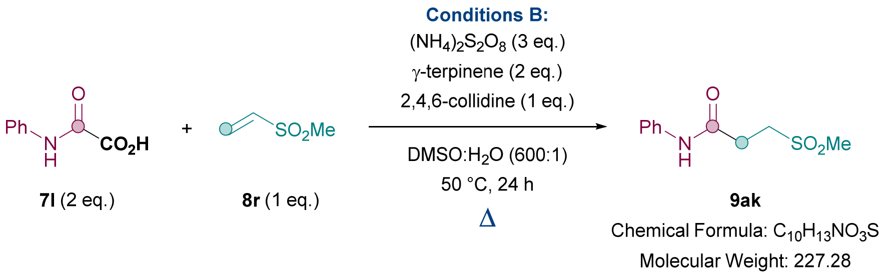

General procedure for Conditions B was followed. Methyl vinyl sulfone **8r** (15.3 mg, 0.14 mmol, 1.0 eq.), *N*-(phenyl)-oxoacetic acid **7l** (47.8 mg, 0.29 mmol, 2.0 eq.), $(NH_4)_2S_2O_8$ (98.7 mg, 0.43 mmol, 3.0 eq.), γ-terpinene (46.0 µL, 0.29 mmol, 2 eq.), and 2,4,6-collidine (19.1 µL, 0.14 mmol, 1.0 eq.) in DMSO:H_2O (600:1) (0.4 mL) was heated at 50 °C for 24 h followed by work-up X. The crude was then purified *via* column flash chromatography eluting with 20:80 hexane:EtOAc. The solvent was then removed *in vacuo* to afford 3-(methylsulfonyl)-*N*-phenylpropanamide **9ak** as a white solid (20.6 mg, 0.077 mmol, 76%).

Characterisation:

^{1}H NMR (300 MHz, Acetone-*d*$_6$): δ ppm 9.35 (s, 1H, NH), 7.64 (d, *J* = 7.5 Hz, 2H, ArH), 7.30 (t, *J* = 8.0 Hz, 2H, ArH), 7.06 (t, *J* = 7.4 Hz, 1H, ArH), 3.45 (t, *J* = 7.4 Hz, 2H, C<u>H</u>$_2$SO$_2$Me), 2.98 (s, 3H, CH_3), 2.91 (t, *J* = 5.4 Hz, 2H, CH_2). **^{13}C NMR (75 MHz, Acetone-*d*$_6$):** 168.6 (C), 140.0 (C), 129.6 (CH), 124.3 (CH), 120.0 (CH), 50.7 (CH_2), 41.1 (CH_3), 30.3 (CH_2). **IR:** ν_{max}/cm^{-1} 3323, 2927, 1671, 1652, 1539, 1490, 1429, 1362, 1298, 1269, 1249. **HRMS (ESI-TOF):** *m*/*z* $[M + H]^+$ calcd for $C_{10}H_{14}NO_3S$, 228.06889; found, 228.0696. **TLC:** R_f = 0.30 (20:80 hexane:EtOAc). **m.p.** 167-170 °C.

Compound 9ak – ^{1}H NMR (300 MHz, Acetone-d_6):

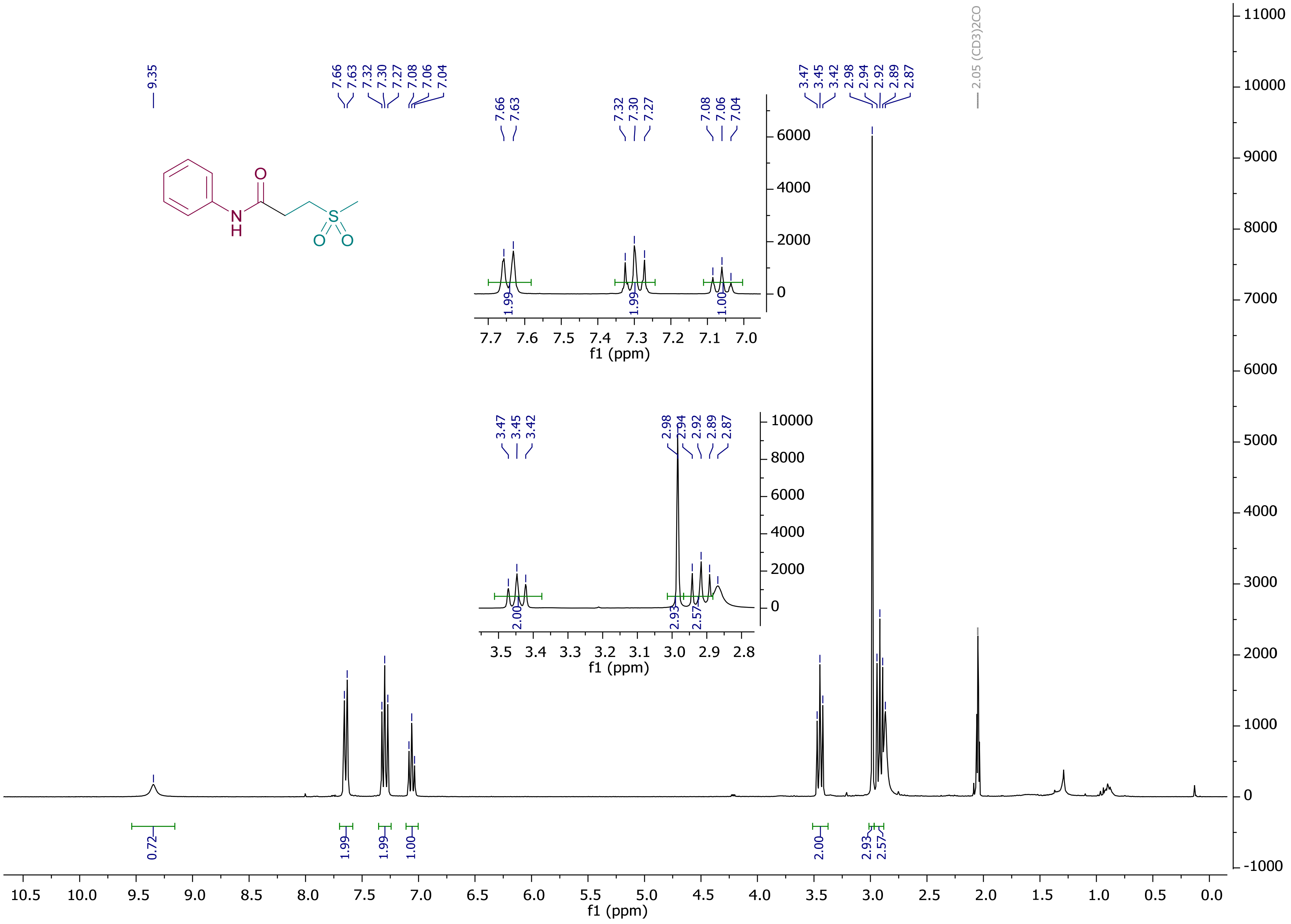

Compound 9ak – ^{13}C NMR (75 MHz, Acetone-d_6):

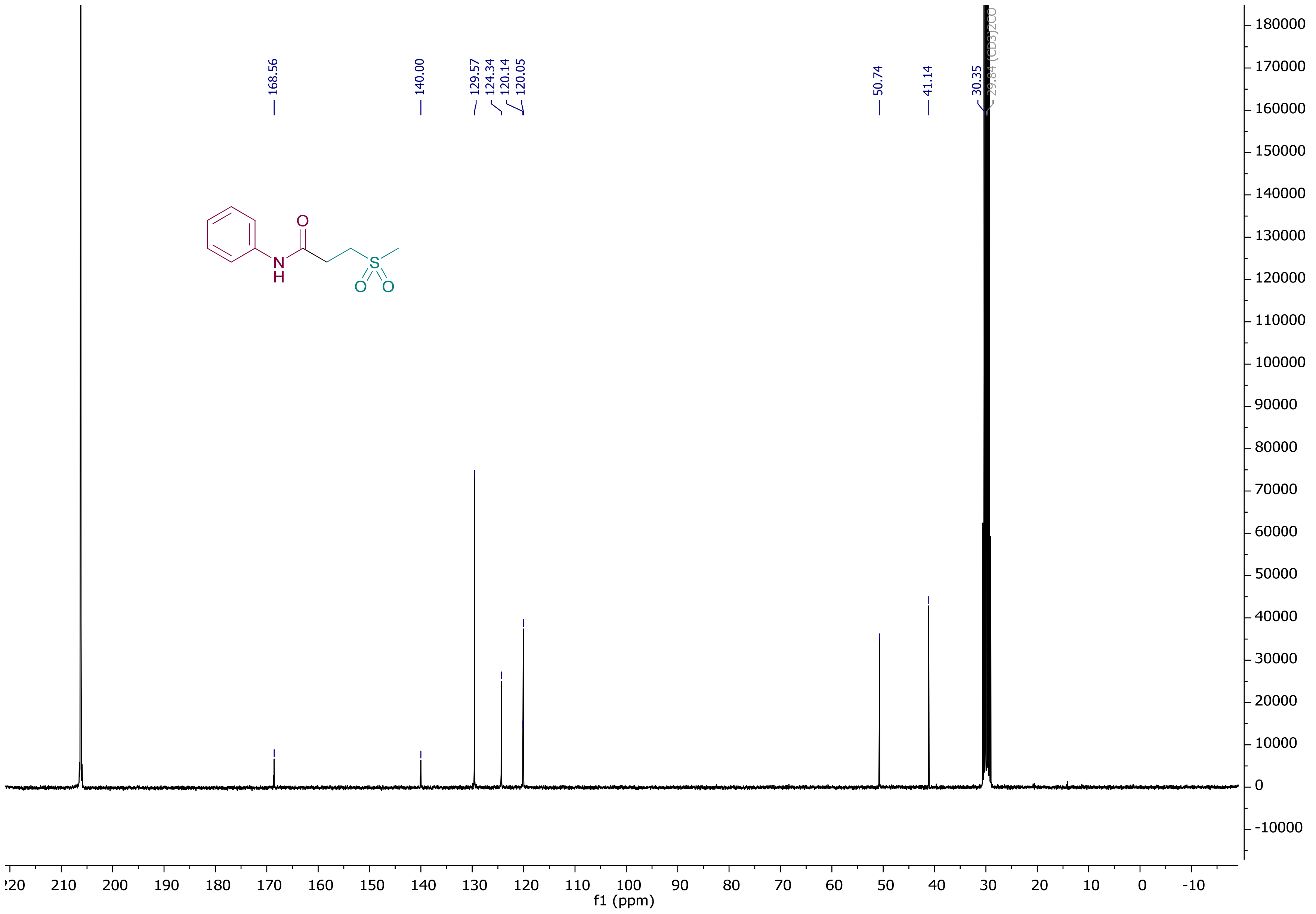

N-Phenyl-3-(phenylsulfonyl)propenamide (9al)

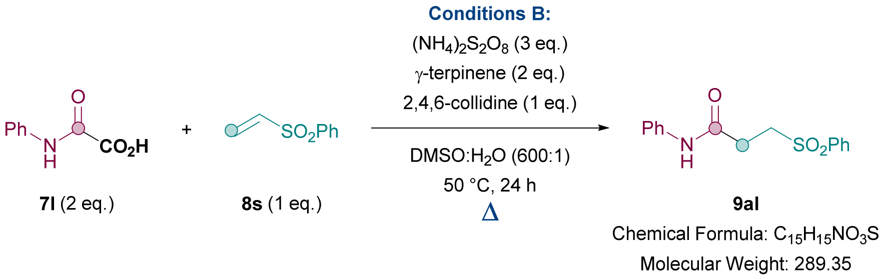

General procedure for Conditions B was followed. Phenyl vinyl sulfone **8s** (20.3 mg, 0.12 mmol, 1.0 eq.), *N*-(phenyl)-oxoacetic acid **7l** (40.0 mg, 0.24 mmol, 2.0 eq.), $(NH_4)_2S_2O_8$ (82.5 mg, 0.36 mmol, 3.0 eq.), γ-terpinene (38.5 µL, 0.24 mmol, 2 eq.), and 2,4,6-collidine (15.9 µL, 0.12 mmol, 1.0 eq.) in DMSO:H_2O (600:1) (0.4 mL) was heated at 50 °C for 24 h followed by work-up X. The crude was then purified *via* column flash chromatography eluting with 60:40 hexane:EtOAc. The solvent was then removed *in vacuo* to afford *N*-phenyl-3-(phenylsulfonyl)propenamide **9al** as a yellow solid (21.3 mg, 61%).

Characterisation:

^{1}H NMR (300 MHz, Chloroform-*d*): δ ppm 8.01 (br s, 1H, NH), 7.94 (d, *J* = 7.1 Hz, 1H, ArH), 7.66 (t, *J* = 7.4 Hz, 1H, ArH), 7.61 – 7.50 (d, *J* = 7.5 Hz, 2H, ArH), 7.43 (d, *J* = 7.4 Hz, 1H, ArH), 7.26 (t, *J* = 15.8 Hz, 1H, ArH), 7.08 (t, *J* = 7.4 Hz, 1H, ArH), 3.56 (t, *J* = 7.6 Hz, 2H, CH_2), 2.92 (dd, *J* = 8.3, 6.9 Hz, 2H, CH_2). **^{13}C NMR (101 MHz, Chloroform-*d*):** δ ppm 167.2 (C), 138.8 (C), 137.7 (C), 134.3 (CH), 129.6 (CH), 129.1 (CH), 128.1 (CH), 124.6 (CH), 120.0 (CH), 52.1 (CH_2), 30.0 (CH_2). **IR:** ν_{max}/cm^{-1} 3338, 3058, 2999, 1688, 1604, 1542, 1521, 1436, 1359, 1299, 1256, 1173, 1144. **HRMS (ESI-TOF):** *m*/*z* $[M + H]^+$ calcd for $C_{15}H_{16}NO_3S$, 290.08454; found, 290.0846. **TLC:** R_f = 0.24 (60:40 hexane:EtOAc). **m.p.** 107-111 °C.

Compound 9al – ^{1}H NMR (300 MHz, Chloroform-*d*):

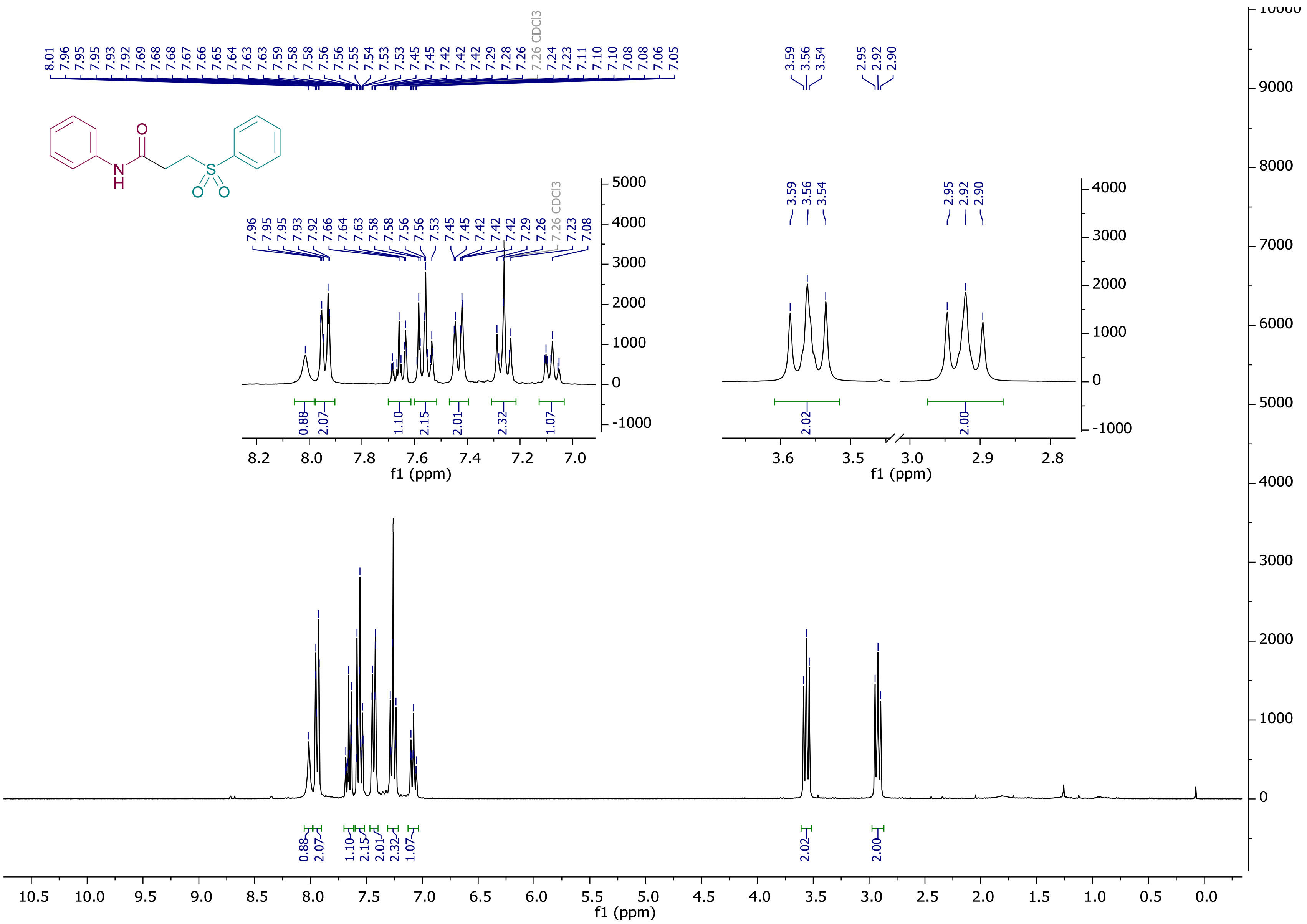

Compound 9al – ^{13}C NMR (101 MHz, Chloroform-*d*):

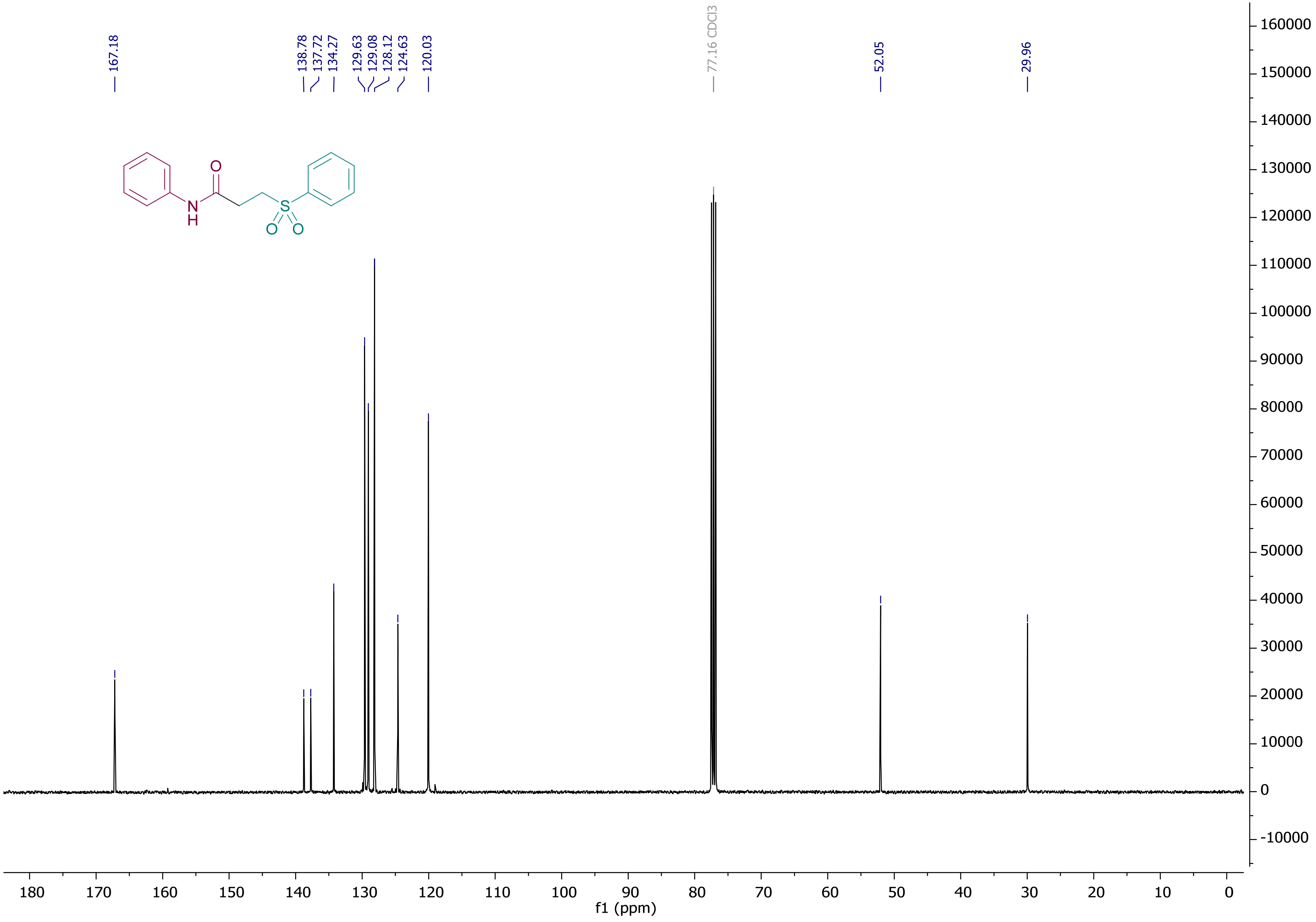

4-Oxo-*N*,2-diphenylpentanamide (9am)

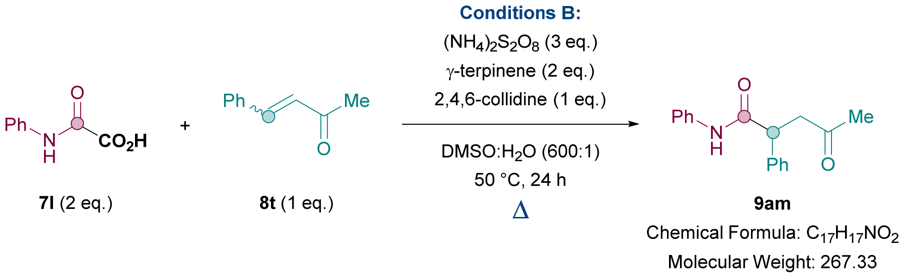

General procedure for conditions B was followed. 4-Phenylbut-3-en-2-one **8t** (18.0 mg, 0.12 mmol, 1.0 eq.), *N*-(phenyl)-oxoacetic acid **7l** (40.0 mg, 0.24 mmol, 2.0 eq.), $(NH_4)_2S_2O_8$ (82.5 mg, 0.36 mmol, 3.0 eq.), γ-terpinene (38.5 µL, 0.24 mmol, 2 eq.), and 2,4,6-collidine (15.9 µL, 0.12 mmol, 1.0 eq.) in DMSO:H_2O (600:1) (0.4 mL) was heated at 30 ºC for 24 h followed by work-up X. The crude was then purified *via* column flash chromatography eluting with 60:40 hexane:EtOAc. The solvent was then removed *in vacuo* to afford 4-oxo-*N*,2-diphenylpentanamide **9am** as a yellow solid (10.8 mg, 34%).

Characterisation:

^{1}H NMR (400 MHz, Chloroform-*d*): δ ppm 8.15 (s, 1H, NH), 7.45 (d, *J* = 7.4 Hz, 2H, ArH), 7.37 – 7.23 (m, 5H, ArH), 7.20 (d, *J* = 7.4 Hz, 2H, ArH), 7.12 (t, *J* = 7.4 Hz, 1H, ArH), 3.80 (dd, *J* = 8.4, 6.8 Hz, 1H, CH), 3.32 (dd, *J* = 13.6, 6.9 Hz, 1H, C<u>H</u>H), 3.21 (dd, *J* = 13.7, 8.4 Hz, 1H, CH<u>H</u>), 2.13 (s, 3H, CH_3). **^{13}C NMR (101 MHz, Chloroform-*d*):** δ ppm 207.9 (C), 166.2 (C), 137.5 (CH), 137.4 (CH), 129.1 (CH), 129.0 (CH), 128.9 (CH), 127.2 (CH), 124.8 (CH), 120.2 (CH), 63.6 (CH), 37.6 (CH_3), 30.8 (CH_2). **IR:** ν_{max}/cm^{-1} 3289, 3029, 2970, 1736, 1718, 1652, 1598, 1525, 1495, 1443, 1361, 1272, 1217, 1207. **HRMS (ESI-TOF):** *m*/*z* [M + H]$^+$ calcd for $C_{17}H_{18}NO_2$, 268.13321; found, 268.1329. **TLC:** R_f = 0.34 (65:35 hexane:EtOAc). **m.p.** 84-88 °C.

Compound 9am – ^{1}H NMR (400 MHz, Chloroform-*d*):

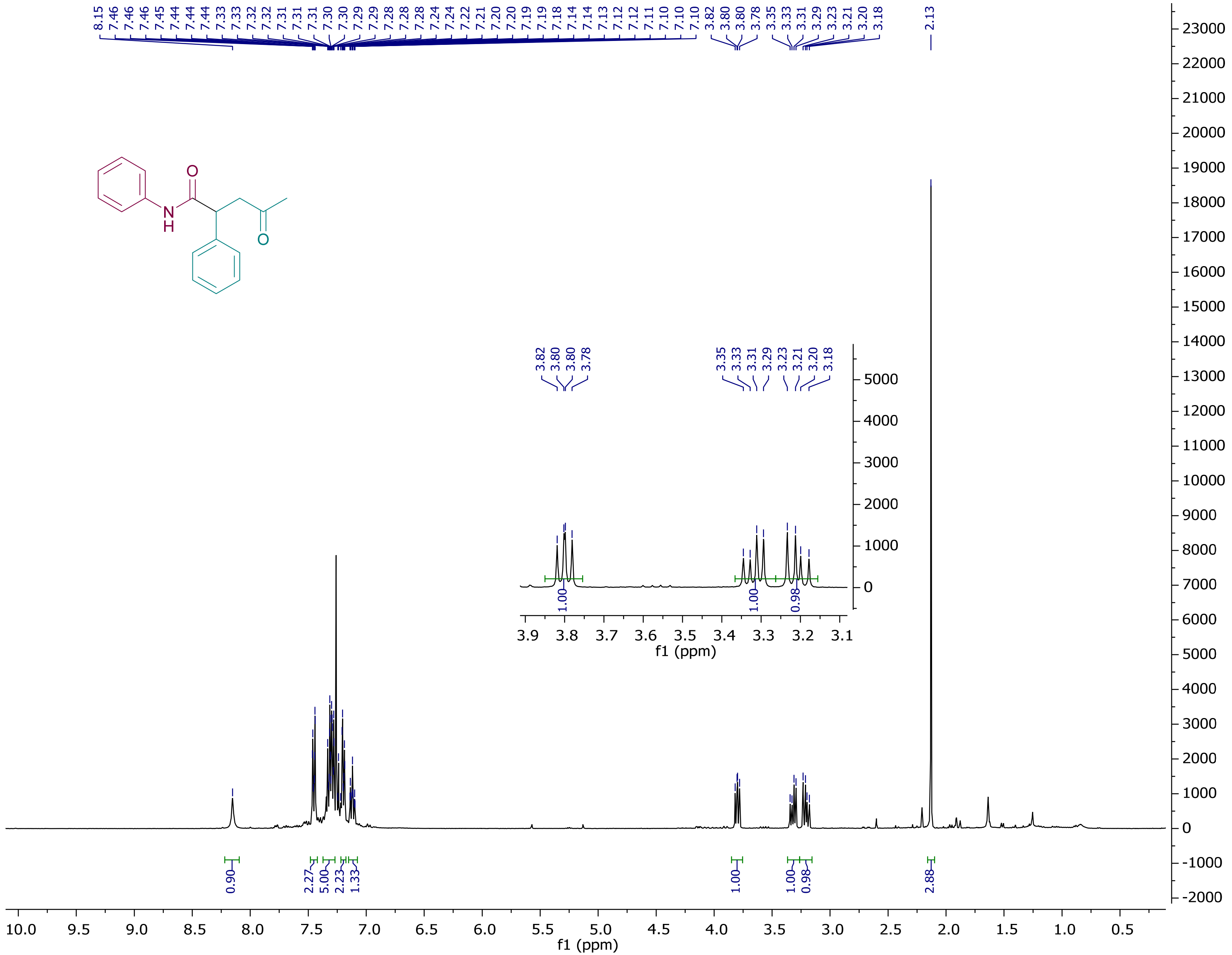

Compound 9am – ^{13}C NMR (101 MHz, Chloroform-*d*):

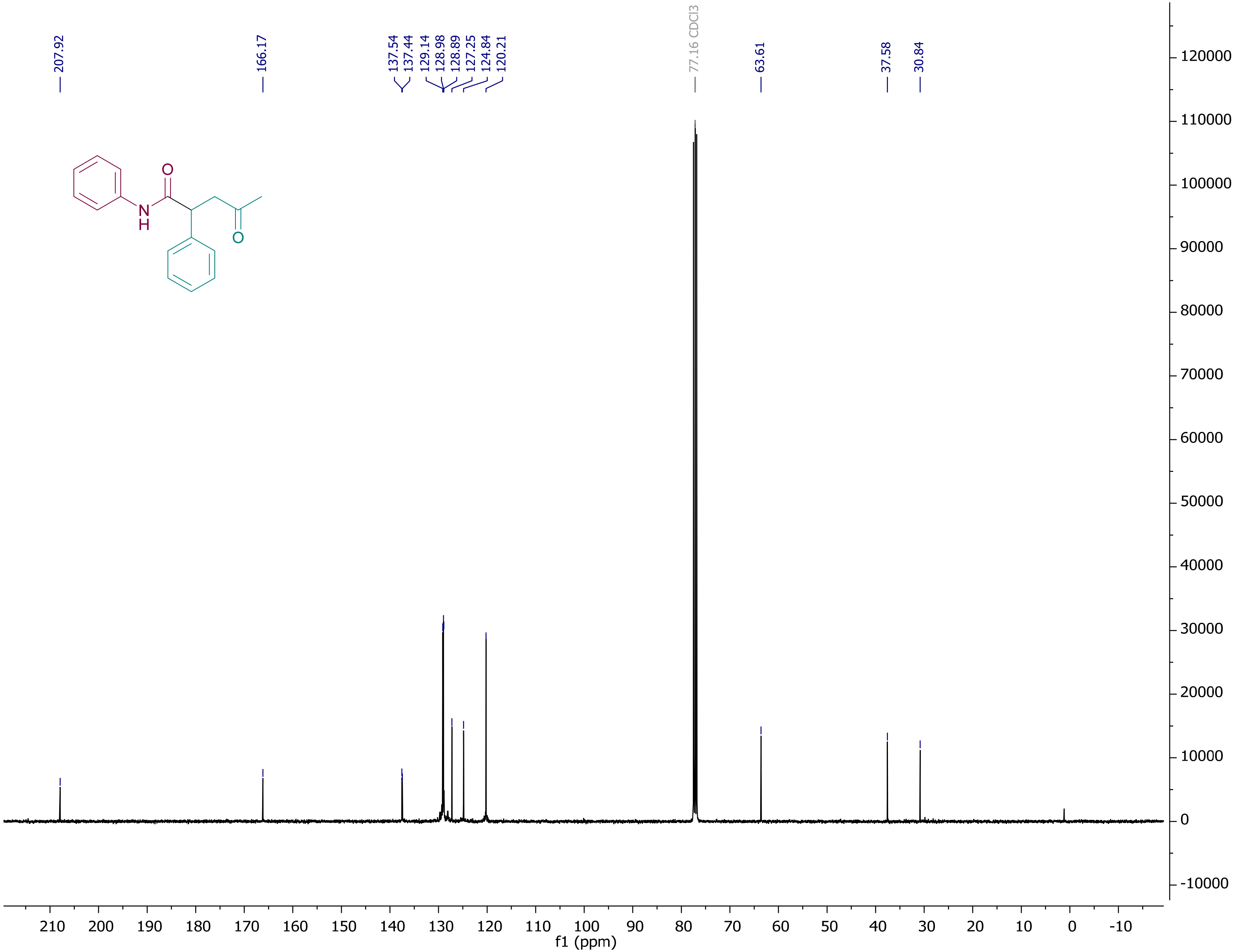

Diethyl 2-(1-(((*R*)-1-methoxy-1-oxopropan-2-yl)amino)-1-oxopropan-2-yl)malonate (9an)

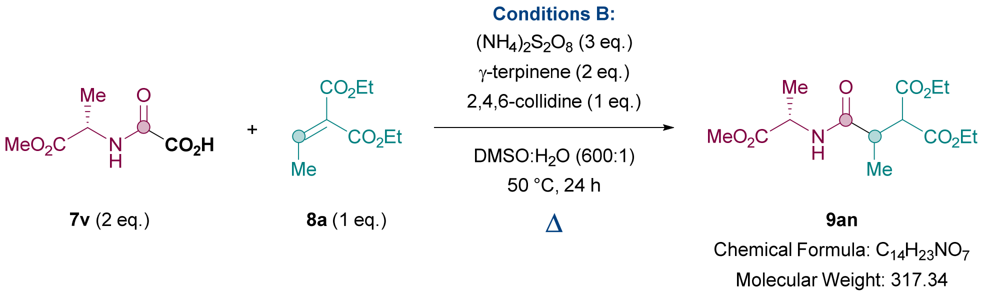

General procedure for conditions B was followed. Diethyl 2-ethylidenemalonate **8a** (22.4 mg, 0.12 mmol, 1.0 eq.), (*R*)-2-((1-methoxy-1-oxopropan-2-yl)amino)-2-oxoacetic acid **7v** (42.3 mg, 0.24 mmol, 2.0 eq.), $(NH_4)_2S_2O_8$ (82.6 mg, 0.36 mmol, 3.0 eq.), γ-terpinene (38.5 µL, 0.24 mmol, 2.0 eq.), and 2,4,6-collidine (15.9 µL, 0.12 mmol, 1.0 eq.) in DMSO:H_2O (600:1) (0.4 mL) was heated at 50 °C for 24 h followed by work-up Y. The crude was then purified *via* column flash chromatography eluting with 70:30→50:50 hexane:EtOAc. The solvent was then removed *in vacuo* to afford a 50:50 diastereomeric mixture of diethyl 2-(1-(((*R*)-1-methoxy-1-oxopropan-2-yl)amino)-1-oxopropan-2-yl)malonate **9an** as a colourless oil (30.1 mg, 79%).

Characterisation:

^{1}H NMR (300 MHz, Chloroform-*d*): (50:50 mixture of diastereomers) δ ppm 6.42 (br d, *J* = 7.1 Hz, 0.5H, NH), 6.29 (br d, J = 7.6 Hz, 0.5H, NH’), 4.62 – 4.45 (m, 1H, C<u>H</u>NH), 4.27 – 4.05 (m, 4H, 2 x OCH_2), 3.73 (m, 3.5H, OCH_3 and C<u>H</u>(CO_2Et)$_2$), 3.70 (d, *J* = 2.4 Hz, 0.5H, C<u>H</u>’(CO_2Et)$_2$), 3.07 – 2.88 (m, 1H, COC<u>H</u>Me), 1.38 (d, *J* = 7.1, 3H, CH_3), 1.38 (d, *J* = 7.1, 3H, CH_3), 1.31 – 1.14 (m, 9H, 3 x CH_3). **^{13}C NMR (101 MHz, Chloroform-*d*):** (50:50 mixture of diastereomers) δ ppm 173.5 (C), 173.43 (C), 173.41 (C), 173.3 (C), 168.5 (C), 168.42 (C), 168.39 (C), 168.3 (C), 61.74 (CH_2), 61.72 (CH_2), 61.70 (CH_2 plus one overlapping CH_2), 54.9 (CH), 54.8 (CH), 52.51 (CH_3), 52.50 (CH_3), 48.3 (CH), 48.2 (CH), 40.5 (CH), 40.4 (CH), 18.5 (CH_3), 18.3 (CH_3), 16.1 (CH_3), 16.0 (CH_3), 14.2 (2 x CH_3), 14.1 (CH_3), 14.0 (CH_3). **IR:** ν_{max}/cm^{-1} 3323, 2982, 2940, 1729, 1670, 1652, 1533, 1456, 1368, 1277, 1183. **HRMS (ESI-TOF):** *m/z* $[M + H]^+$ calcd for $C_{14}H_{24}NO_7$, 318.15473; found, 318.1546. **TLC:** R_f = 0.39 (50:50 hexane:EtOAc).

Compound 9an – ^{1}H NMR (300 MHz, Chloroform-*d*): (50:50 mixture of diastereomers)

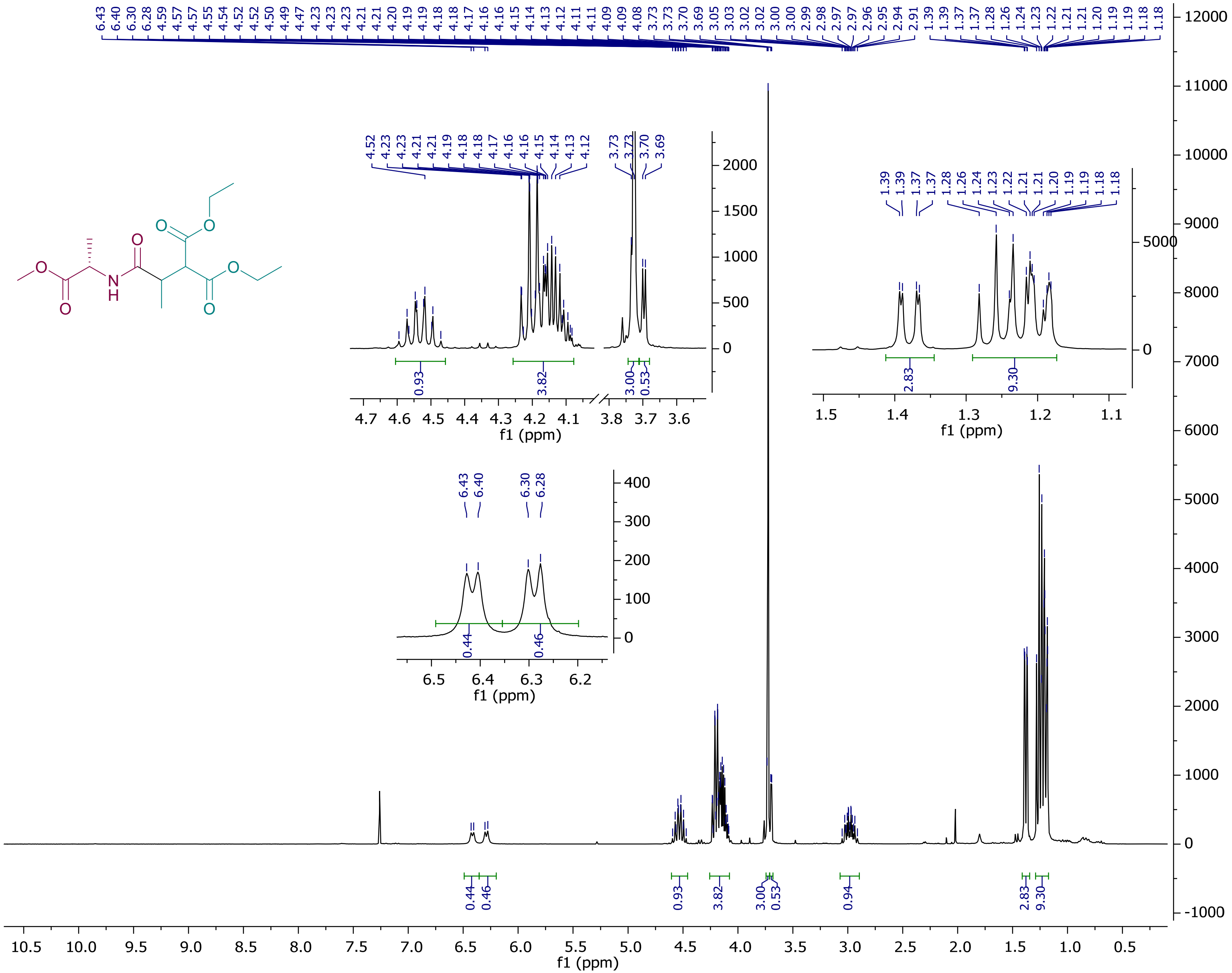

Compound 9an – ^{13}C NMR (101 MHz, Chloroform-*d*): (50:50 mixture of diastereomers)

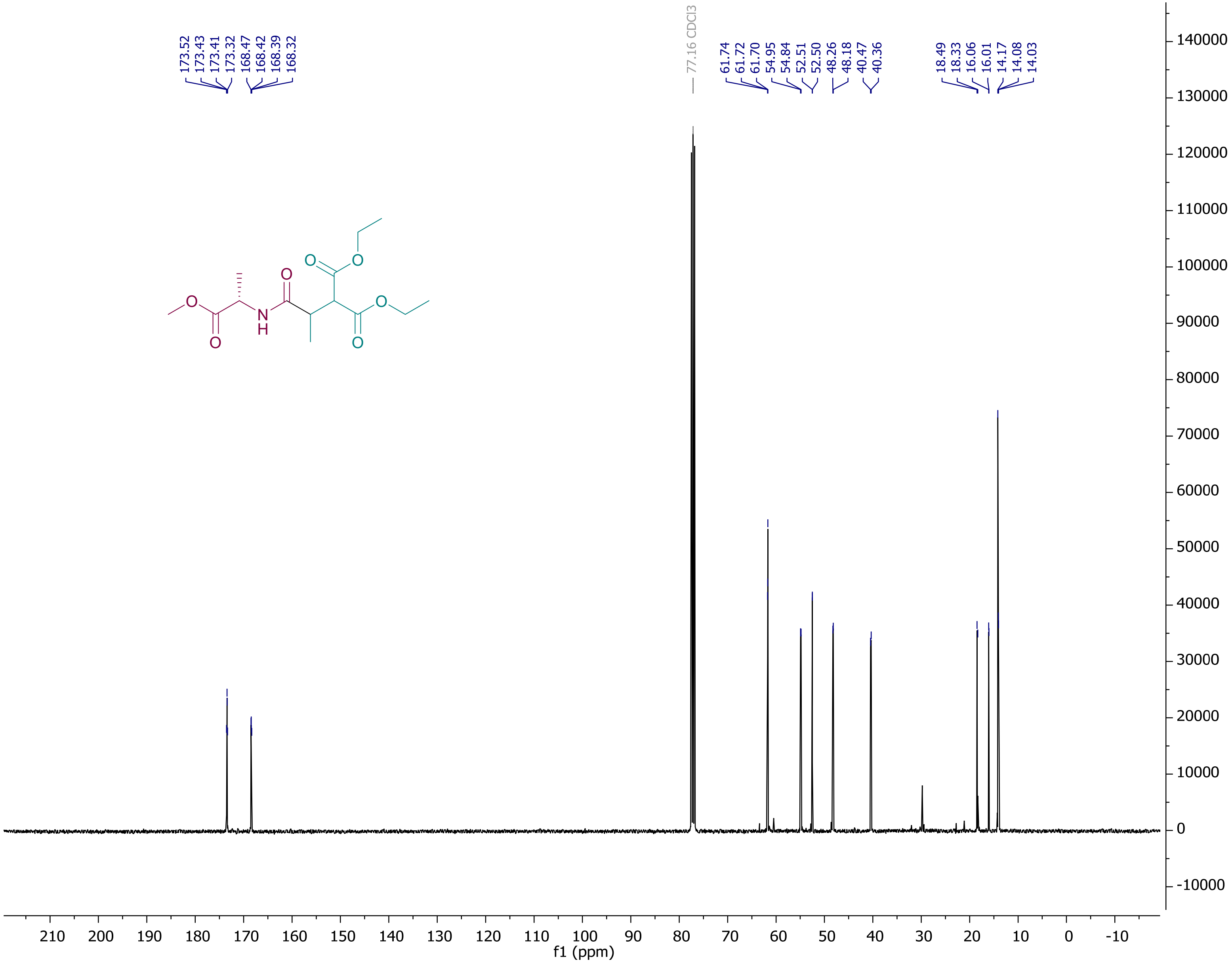

(+)-Methyl (3-(phenylsulfonyl)propanoyl)-D-valinate (9ao)

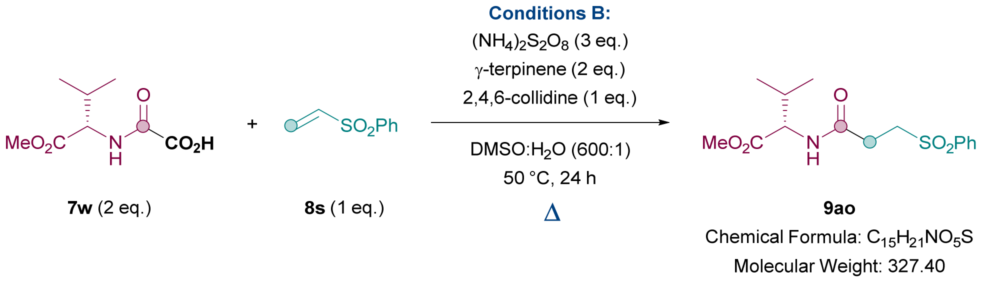

General procedure for conditions B was followed. Phenyl vinyl sulfone **8s** (40.8 mg, 0.24 mmol, 1.0 eq.), (*S*)-2-((1-methoxy-3-methyl-1-oxobutan-2-yl)amino)-2-oxoacetic acid **7w** (99.0 mg, 0.48 mmol, 2.0 eq.), $(NH_4)_2S_2O_8$ (167.0 mg, 0.72 mmol, 3.0 eq.), γ-terpinene (77.8 µL, 0.48 mmol, 2.0 eq.), and 2,4,6-collidine (32.3 µL, 0.24 mmol, 1.0 eq.) in DMSO:H_2O (600:1) (0.4 mL) was heated at 50 °C for 24 h followed by work-up Y. The crude was then purified *via* column flash chromatography eluting with 70:30→50:50 hexane:EtOAc. The solvent was then removed *in vacuo* to afford (+)-methyl (3-(phenylsulfonyl)propanoyl)-*D*-valinate **9ao** as a colourless oil (51.1 mg, 0.16 mmol, 64%).

Characterisation:

^{1}H NMR (300 MHz, Chloroform-*d*): δ ppm 7.96 – 7.85 (m, 2H, ArH), 7.69 – 7.60 (m, 1H, ArH), 7.60 – 7.51 (m, 2H, ArH), 6.39 (br d, *J* = 8.7 Hz, 1H, NH), 4.45 (dd, *J* = 8.7, 5.0 Hz, 1H, C<u>H</u>NH), 3.71 (s, 3H, OCH_3), 3.55 – 3.35 (m, 2H, C<u>H</u>$_2SO_2$Ph), 2.85 – 2.64 (m, 2H, CH_2), 2.16-2.05 (m, 1H, C<u>H</u>$(Me)_2$), 0.88 (t, *J* = 6.7 Hz, 6H, 2 x CH_3). **^{13}C NMR (101 MHz, Chloroform-*d*):** δ ppm 172.4 (C), 168.9 (C), 138.9 (C), 134.0 (CH), 129.5 (CH), 128.1 (CH), 57.5 (CH), 52.3 (CH_3), 51.9 (CH_2), 31.2 (CH), 29.0 (CH_2), 19.0 (CH_3), 17.9 (CH_3). **IR:** ν_{max}/cm^{-1} 3362, 2964, 2933, 2876, 1739, 1656, 1533, 1467, 1447, 1307, 1288, 1206, 1147. **HRMS (ESI-TOF):** *m/z* $[M + H]^+$ calcd for $C_{15}H_{22}NO_5S$, 328.12132; found, 328.1216. **TLC:** R_f = 0.30 (50:50% hexane:EtOAc). **$[\alpha]_D^{20.7}$** = +8.0 (*c* = 1, $CHCl_3$).

No racemisation of stereocentre observed by CSP-HPLC analysis (CHIRALPAK IA, 10% 2-isopropanol in hexane, flow rate: 1.0 mL min^{-1}, detection: UV 210 nm, 25 °C). t_R of major isomer: 26.1 min.

CSP-HPLC traces:

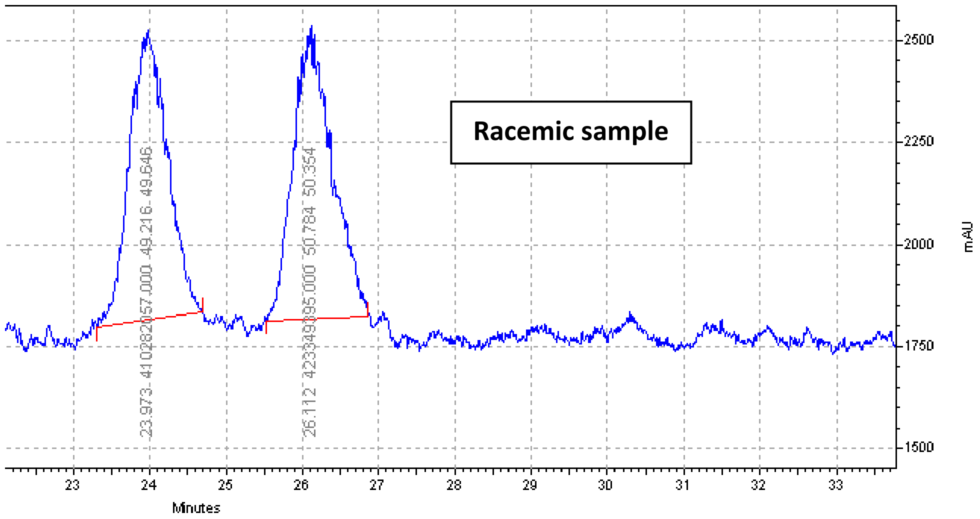

VWD: Signal A, 210 nm Results

Retention Time	Area	Area %	Height	Height %
23.973	410282057	49.22	11930385	49.65
26.112	423349395	50.78	12100622	50.35
Totals	833631452	100.00	24031007	100.00

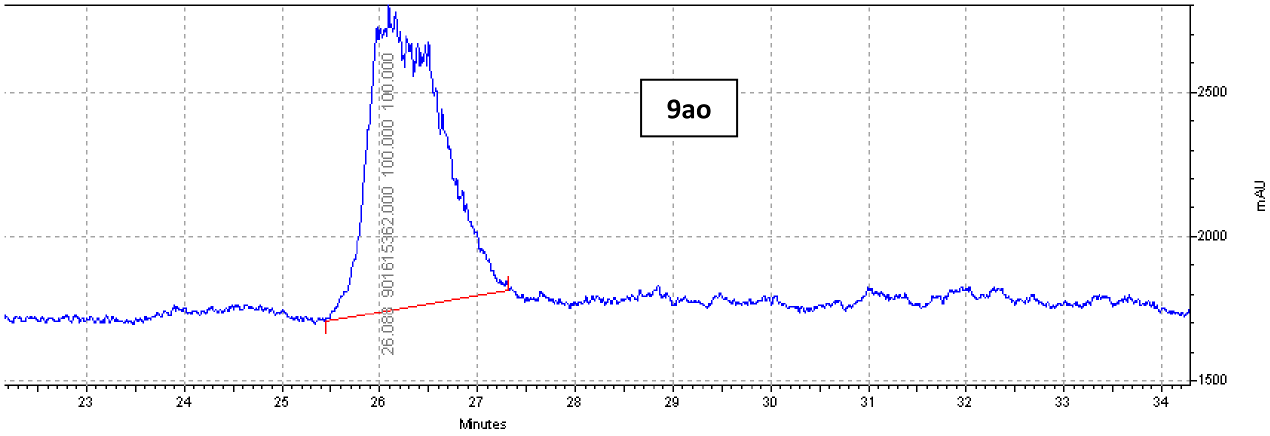

VWD: Signal A, 210 nm Results

Retention Time	Area	Area %	Height	Height %
26.088	901615362	100.00	17738959	100.00
Totals	901615362	100.00	17738959	100.00

Compound 9ao – ^{1}H NMR (300 MHz, Chloroform-*d*):

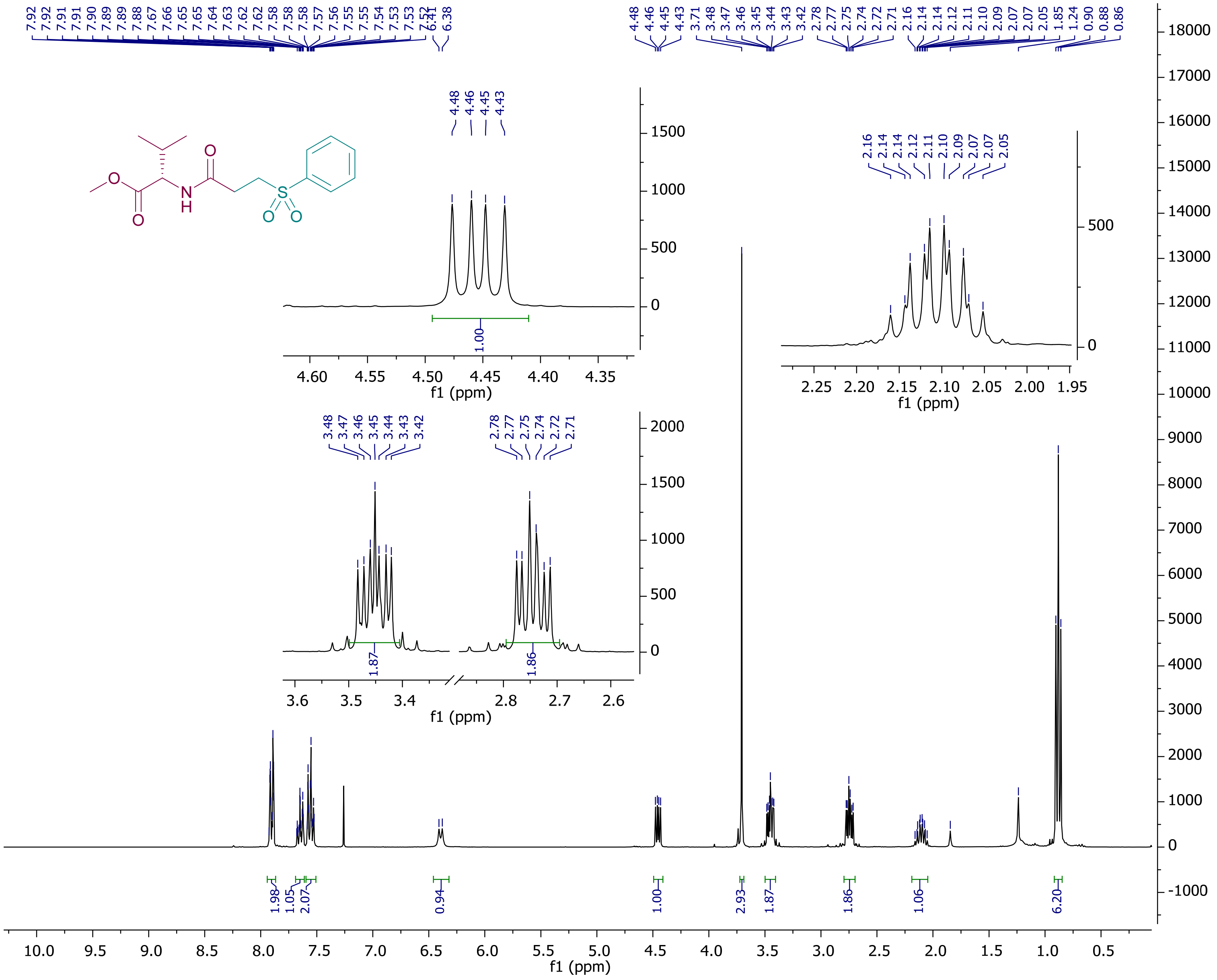

Compound 9ao – ^{13}C NMR (101 MHz, Chloroform-*d*):

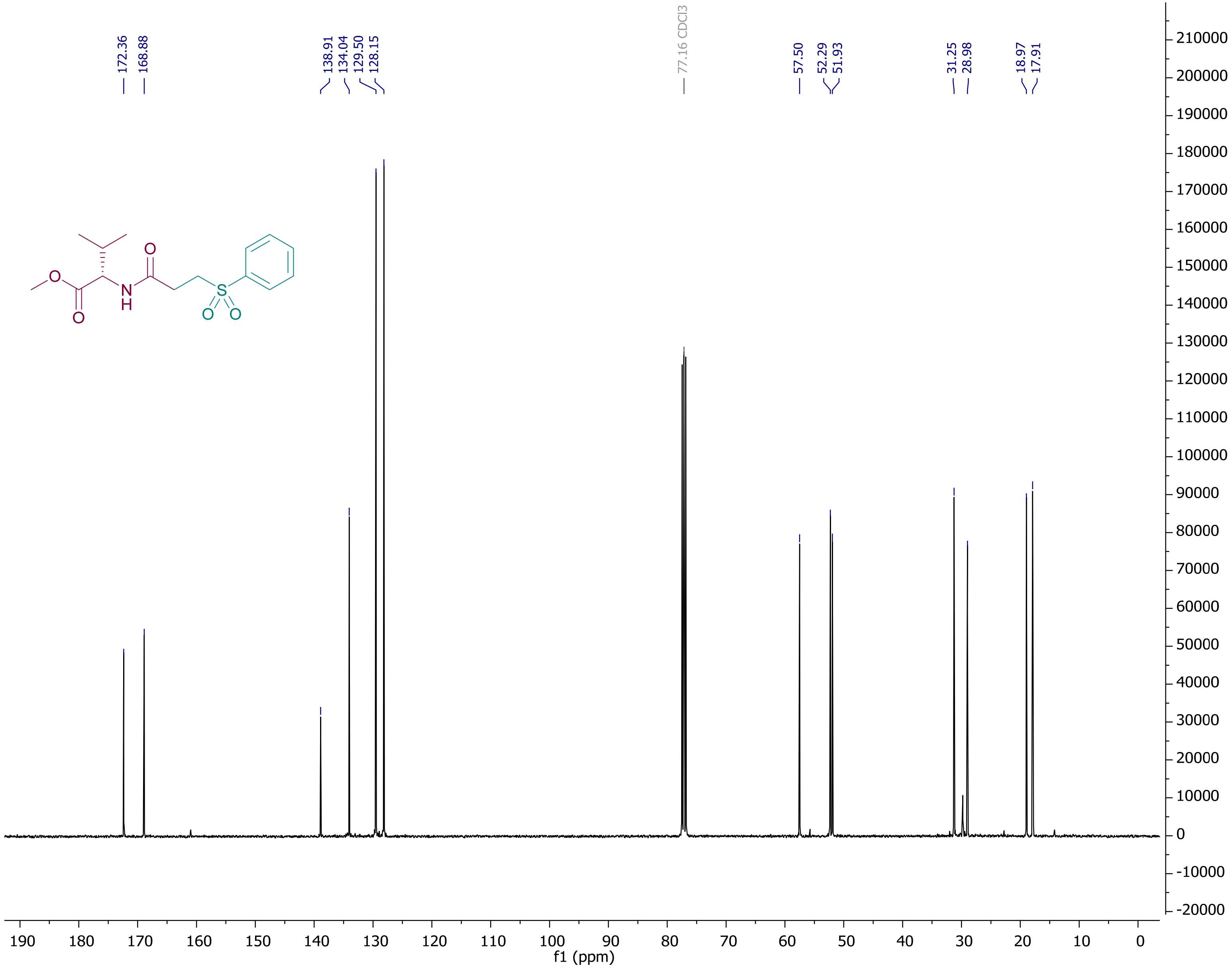

(+)-*N*-(((1*R*,4a*S*,10a*R*)-7-isopropyl-1,4a-dimethyl-1,2,3,4,4a,9,10,10a-octahydrophenanthren-1-yl)methyl)-3-(phenylsulfonyl)propenamide (9ap)

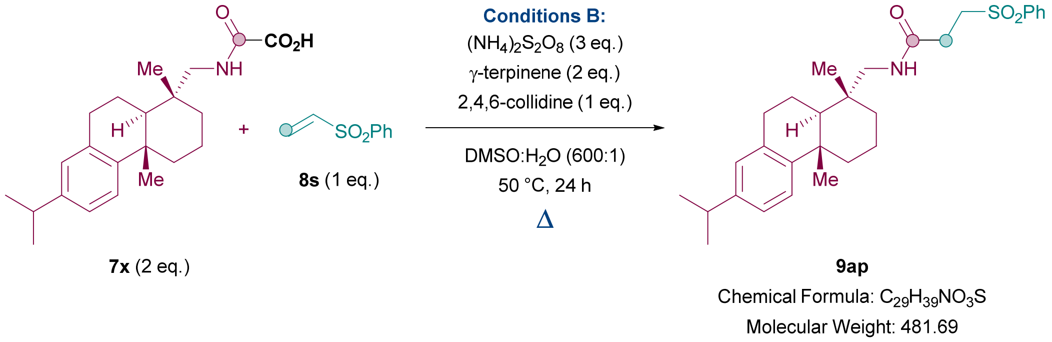

General procedure for conditions B was followed. Phenyl vinyl sulfone **8s** (19.1 mg, 0.11 mmol, 1.0 eq.), 2-((((1*R*,4a*S*,10a*R*)-7-isopropyl-1,4a-dimethyl-1,2,3,4,4a,9,10,10a-octahydrophenanthren-1-yl)methyl)amino)-2-oxoacetic acid **7x** (81.6 mg, 0.22 mmol, 2.0 eq), $(NH_4)_2S_2O_8$ (78.1 mg, 0.33 mmol, 3.0 eq.), γ-terpinene (36.5 µL, 0.22 mmol, 2.0 eq.), and 2,4,6-collidine (15.1 µL, 0.11 mmol, 1.0 eq.) in DMSO:H_2O (600:1) (0.4 mL) was heated at 50 °C for 24 h followed by work-up Y. The crude was then purified *via* column flash chromatography eluting with 65:35 hexane:EtOAc. The solvent was then removed *in vacuo* to afford methyl (+)-*N*-(((1*R*,4a*S*,10a*R*)-7-isopropyl-1,4a-dimethyl-1,2,3,4,4a,9,10,10a-octahydrophenanthren-1-yl)methyl)-3-(phenylsulfonyl)propenamide **9ap** as a colourless oil (35.3 mg, 65%).

Characterisation:

^{1}H NMR (400 MHz, Chloroform-*d*): δ ppm 7.88 (d, *J* = 7.7 Hz, 2H, ArH), 7.65 (t, *J* = 7.4 Hz, 1H, ArH), 7.54 (t, *J* = 7.7 Hz, 2H, ArH), 7.16 (d, *J* = 8.2 Hz, 1H, ArH), 7.00 (d, *J* = 8.1 Hz, 1H, ArH), 6.88 (s, 1H, ArH), 5.78 (br t, *J* = 5.9 Hz, 1H, NH), 3.44 (td, *J* = 7.3, 4.2 Hz, 2H, SCH_2), 3.17 (dd, *J* = 13.7, 6.2 Hz, 1H, C$\underline{H}$HNH), 3.07 (dd, *J* = 13.7, 6.6 Hz, 1H, CH$\underline{H}$NH), 2.94 – 2.70 (m, 3H, C$\underline{H}$(Me)$_2$, CH_2), 2.65 (t, *J* = 7.7 Hz, 2H, CH_2CO), 2.27 (d, *J* = 12.8 Hz, 1H, C$\underline{H}$H), 1.93 – 1.54 (m, 4H, 2 x CH_2), 1.43 – 1.28 (m, 3H, CH$\underline{H}$, C$\underline{H}$H, CH), 1.27 – 1.14 (m, 10H, CH$\underline{H}$, 3 x CH_3), 0.91 (s, 3H, CH_3). **^{13}C NMR (101 MHz, Chloroform-*d*):** δ ppm 169.0 (C), 147.2 (C), 145.8 (C), 138.9 (C), 134.8 (C), 134.1 (CH), 129.5 (CH), 128.1 (CH), 127.1 (CH), 124.3 (CH), 124.0 (CH) 52.2 (CH_2), 50.3 (CH_2), 45.4 (CH), 38.4 (CH_2), 37.55 (C), 37.48 (C), 36.2 (CH_2), 33.6 (CH), 30.3 (CH_2), 29.3 (CH_2), 25.4 (CH_3), 24.1 (CH_3), 19.1 (CH_2),

18.8 (CH_3), 18.6 (CH_2). **IR:** ν_{max}/cm^{-1} 3338, 2958, 2926, 2865, 2358, 2337, 1671, 1652, 1544, 1498, 1447, 1382, 1306, 1288, 1255. **HRMS (ESI-TOF):** *m*/*z* $[M + H]^+$ calcd for $C_{29}H_{40}NO_3S$, 482.27234; found, 482.2725. **TLC:** R_f = 0.33 (60:40 hexane:EtOAc). $[\alpha]_D^{21.5}$ = +20.0 (*c* = 1, $CHCl_3$).

Compound 9ap – ^{1}H NMR (400 MHz, Chloroform-*d*):

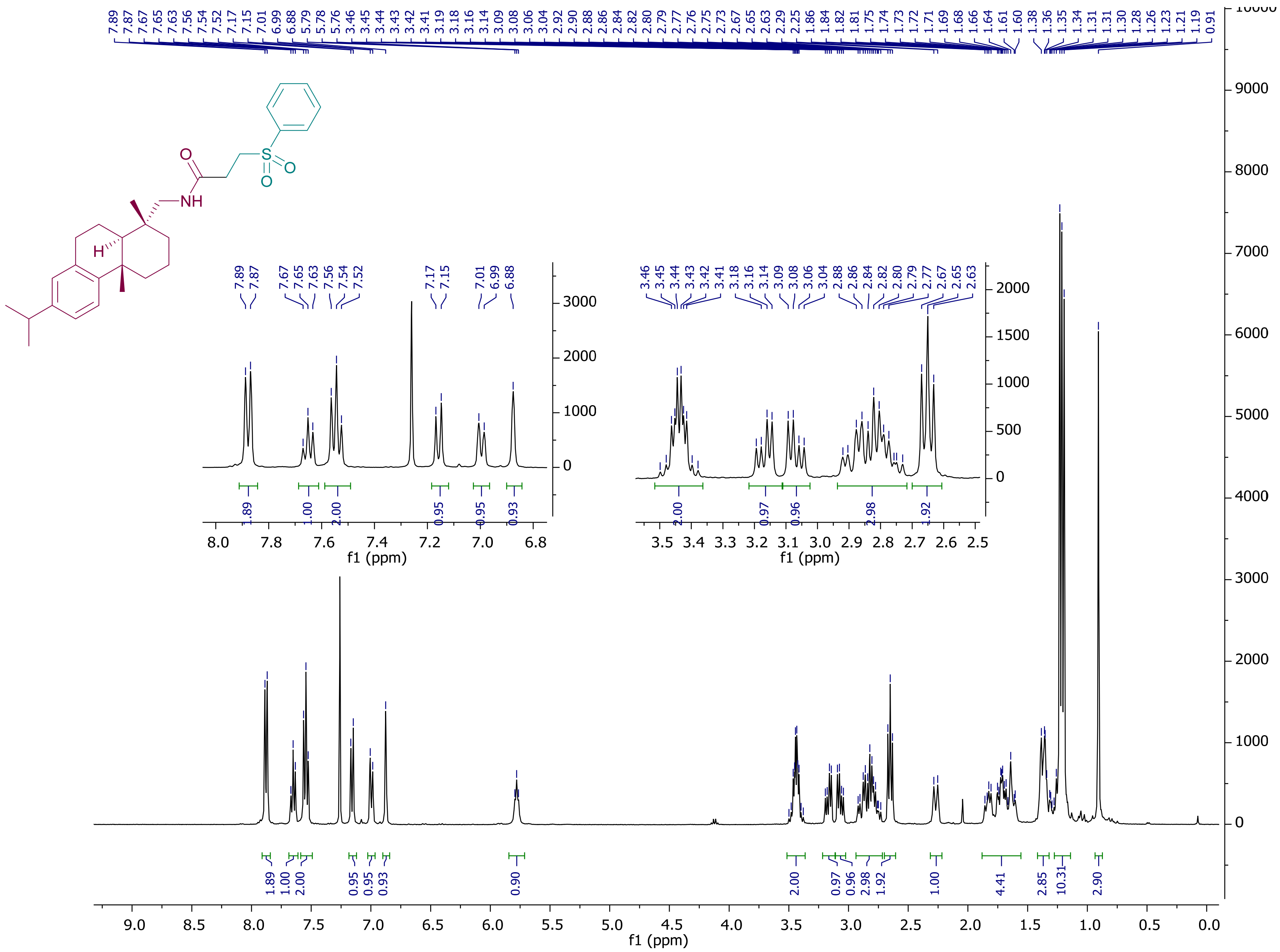

Compound 9ap – ^{13}C NMR (101 MHz, Chloroform-*d*):

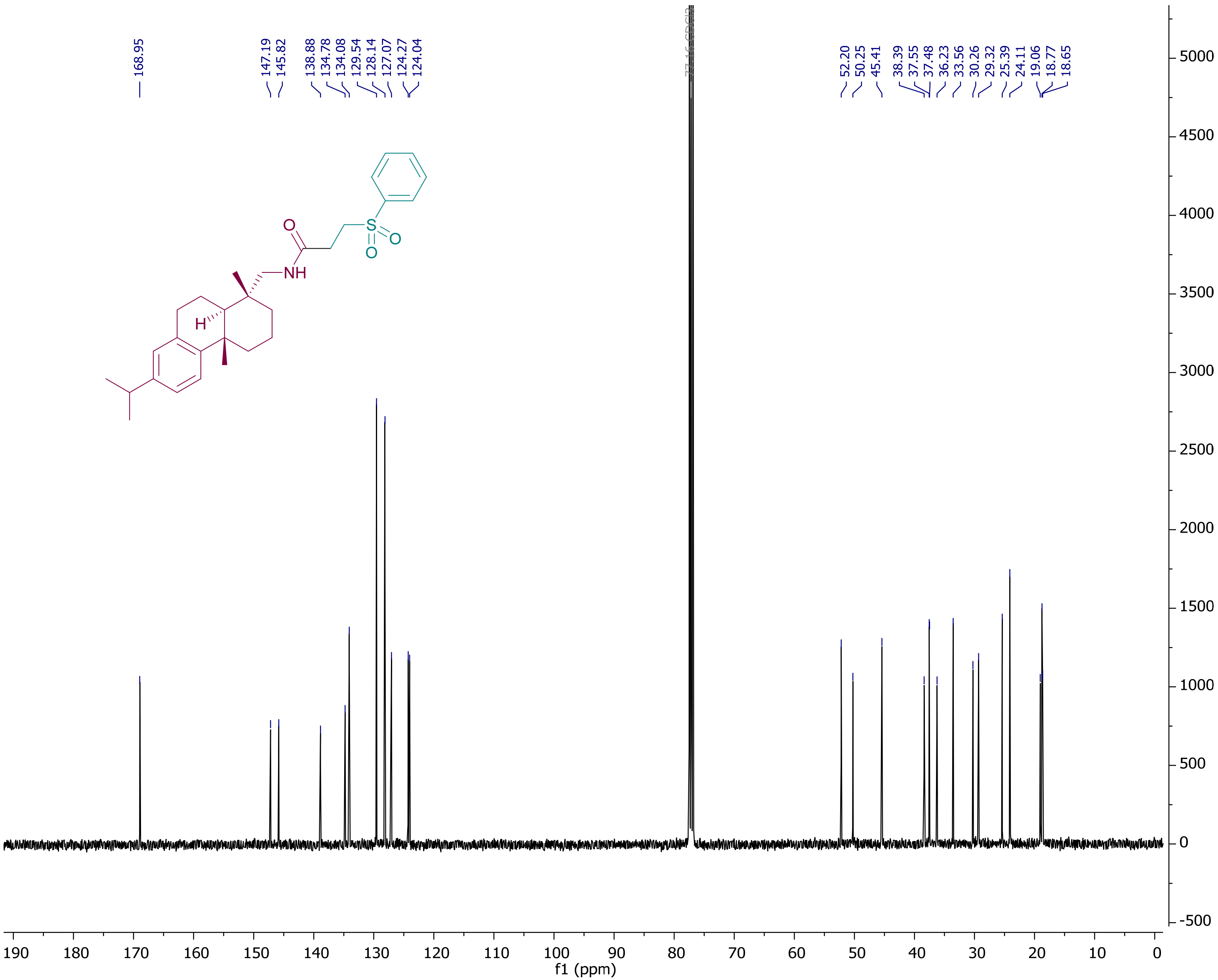

N-cyclohexyl-2-isopropyl-5-oxocyclohexane-1-carboxamide (9aq)

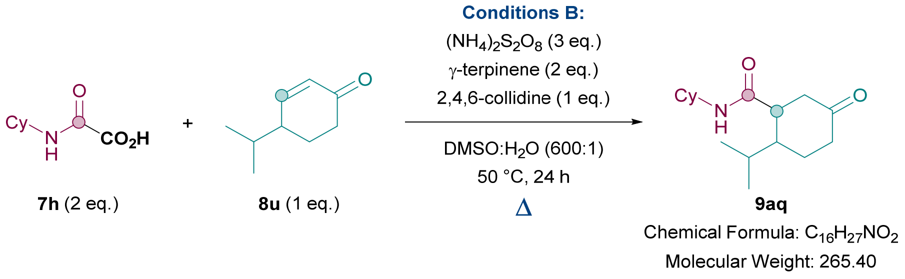

General procedure for conditions B was followed. (±)-Cryptone **8u** (16.6 mg, 0.12 mmol, 1.0 eq.), *N*-(cyclohexyl)-oxoacetic acid **7h** (41.4 mg, 0.24 mmol, 2.0 eq), $(NH_4)_2S_2O_8$ (82.4 mg, 0.36 mmol, 3.0 eq.), γ-terpinene (38.5 µL, 0.24 mmol, 2.0 eq.), and 2,4,6-collidine (15.9 µL, 0.12 mmol, 1.0 eq.) in DMSO:H_2O (600:1) (0.4 mL) was heated at 50 ºC for 24 h followed by work-up Y. The crude was then purified *via* column flash chromatography eluting with 65:35→40:60 hexane:EtOAc. The solvent was then removed *in vacuo* to afford cyclohexyl-2-isopropyl-5-oxocyclohexane-1-carboxamide **9aq** as 79:21 mixture of diastereomers that were separable *via* column chromatography. The major diastereomer was isolated as an off-white solid (17.0 mg, 0.064 mmol, 53%) and the minor diastereomer was isolated as a yellow solid (4.6 mg, 0.017 mmol, 14%).

Characterisation for Major Diastereomer:

^{1}H NMR (400 MHz, Chloroform-*d*): δ ppm 5.55 (br d, *J* = 8.4 Hz, 1H, NH), 3.83 – 3.71 (m, 1H, C<u>H</u>NH), 2.70 (dd, *J* = 13.9, 11.9 Hz, 1H, C<u>H</u>H), 2.44 – 2.23 (m, 4H, CH<u>H</u>, CH, CH_2), 2.08 – 1.94 (m, 2H, CH, C<u>H</u>H), 1.94 – 1.78 (m, 3H, 2 x C<u>H</u>H, CH), 1.69 (dt, *J* = 13.2, 3.9 Hz, 2H, 2 x C<u>H</u>H), 1.60 (dt, *J* = 12.8, 3.7 Hz, 1H, C<u>H</u>H), 1.49 – 1.26 (m, 3H, 3 x CH<u>H</u>), 1.22 – 1.04 (m, 3H, 3 x CH<u>H</u>), 0.97 (d, *J* = 6.9 Hz, 3H, CH_3), 0.80 (d, *J* = 7.0 Hz, 3H, CH_3). **^{13}C NMR (101 MHz, Chloroform-*d*):** δ ppm 211.0 (C), 172.0 (C), 49.5 (CH), 48.3 (CH), 44.3 (CH_2), 43.7 (CH), 40.7 (CH_2), 33.4 (CH_2), 33.1 (CH_2), 28.2 (CH), 25.6 (CH_2), 24.91 (CH_2), 24.88 (CH_2), 24.3 (CH_2), 21.6 (CH_3), 16.3 (CH_3). **IR:** ν_{max}/cm^{-1} 3261, 2930, 2853, 1739, 1713, 1634, 1553, 1449, 1371, 1202. **HRMS (ESI-TOF):** *m/z* [M + H]$^+$ calcd for $C_{16}H_{28}NO_2$, 266.21146; found, 266.2116. **TLC:** R_f = 0.29 (60:40 hexane:EtOAc). **m.p.** 151-155 °C

Characterisation for Minor Diastereomer:

^{1}H NMR (400 MHz, Chloroform-*d*): δ ppm 5.30 (br d, *J* = 7.6 Hz, 1H, NH), 3.82 – 3.69 (m, 1H, CH), 2.86 – 2.81 (m, 1H, CH), 2.56 – 2.48 (m, 1H, C<u>H</u>H), 2.44 (dt, *J* = 15.0, 2.4 Hz, 1H, C<u>H</u>H), 2.33 (dd, *J* = 15.0, 5.4 Hz, 1H, CH<u>H</u>), 2.28 – 2.20 (m, 1H, CH<u>H</u>), 2.08 – 1.97 (m, 2H, CH_2), 1.93 – 1.80 (m, 2H, 2 x C<u>H</u>H), 1.76 – 1.53 (m, 5H, 3 x C<u>H</u>H, 2 x CH), 1.45 – 1.21 (m, 2H, 2 x CH_2), 1.21 – 1.06 (m, 3H, 3 x CH<u>H</u>), 1.04 (d, *J* = 6.1 Hz, 3H, CH_3), 0.96 (d, *J* = 6.1 Hz, 3H, CH_3). **^{13}C NMR (101 MHz, Chloroform-*d*):** δ ppm 209.7 (C), 172.1 (C), 48.3 (CH), 45.9 (CH), 45.7 (CH), 44.1 (CH_2), 40.1 (CH_2), 33.5 (CH_2), 33.2 (CH_2), 30.5 (CH), 25.64, (CH_2) 25.57 (CH_2), 25.0 (CH_2), 24.9 (CH_2), 22.1 (CH_3), 21.1 (CH_3). **IR:** ν_{max}/cm^{-1} 3307, 2930, 2854, 1706, 1634, 1538, 1447, 1368, 1255, 1203. **HRMS (ESI-TOF):** *m*/*z* $[M + H]^+$ calcd for $C_{16}H_{28}NO_2$, 266.21146; found, 266.2109. **TLC:** R_f = 0.35 (40:60 hexane:EtOAc). **m.p.** 138-142 °C

Assignment of diastereomers by coupling constants:

The major diastereomer is assigned as *anti*, based on the multiplicities and *J* values of the C<u>H</u>H peaks. For example, the C<u>H</u>H peak at δ 2.70 ppm is a dd with *J* = 13.9, 11.9 Hz. The large 13.9 and 11.9 Hz are consistent with $^3J_{axial\text{-}axial}$ and ^2J (H_a), indicating the *anti* diastereomer (major conformer with both substituents equatorial).

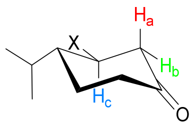

X=CONHCy

Compound 9aq (Major Diastereomer)– ^{1}H NMR (400 MHz, Chloroform-*d*):

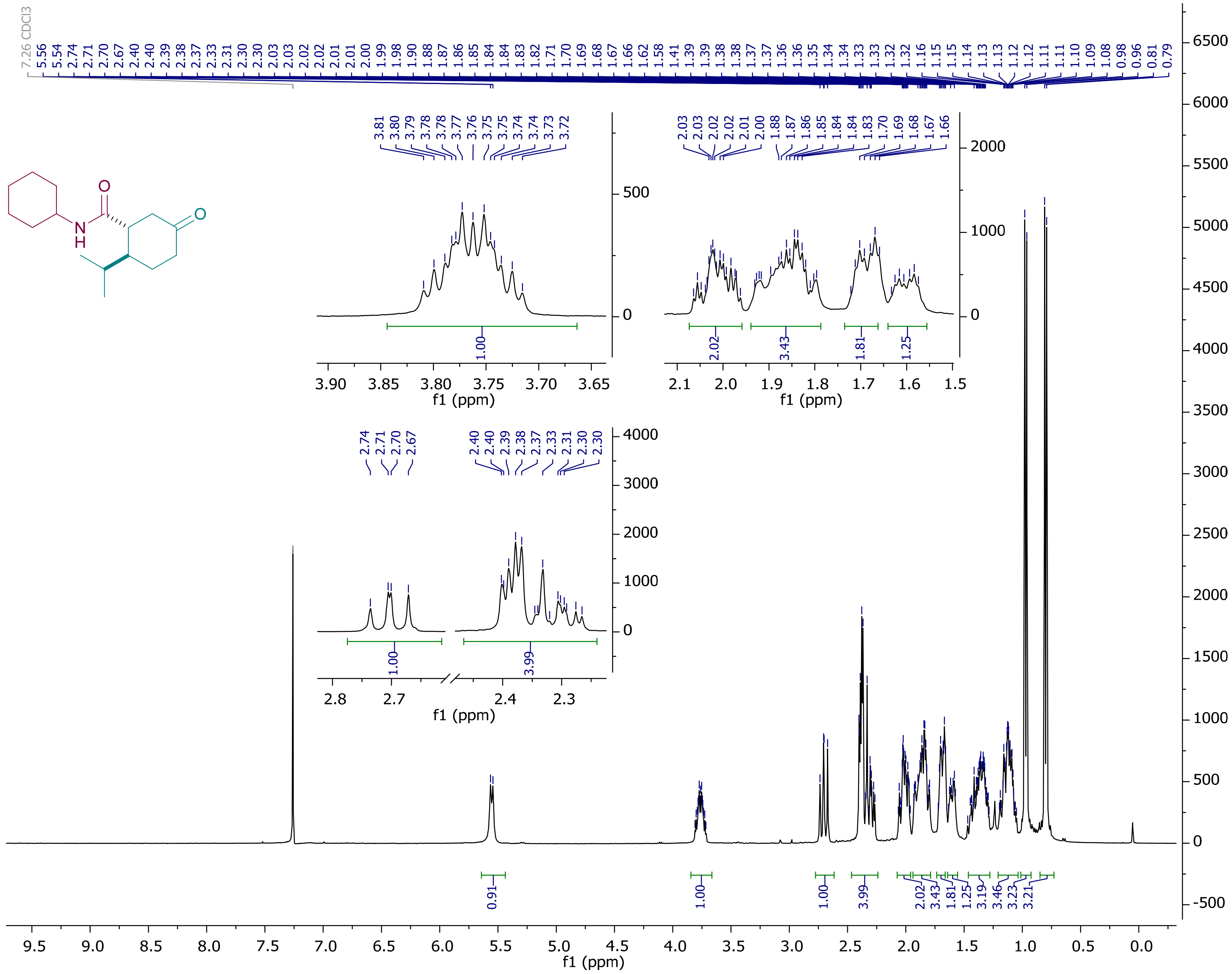

Compound 9aq (Major Diastereomer)– ^{13}C NMR (101 MHz, Chloroform-*d*):

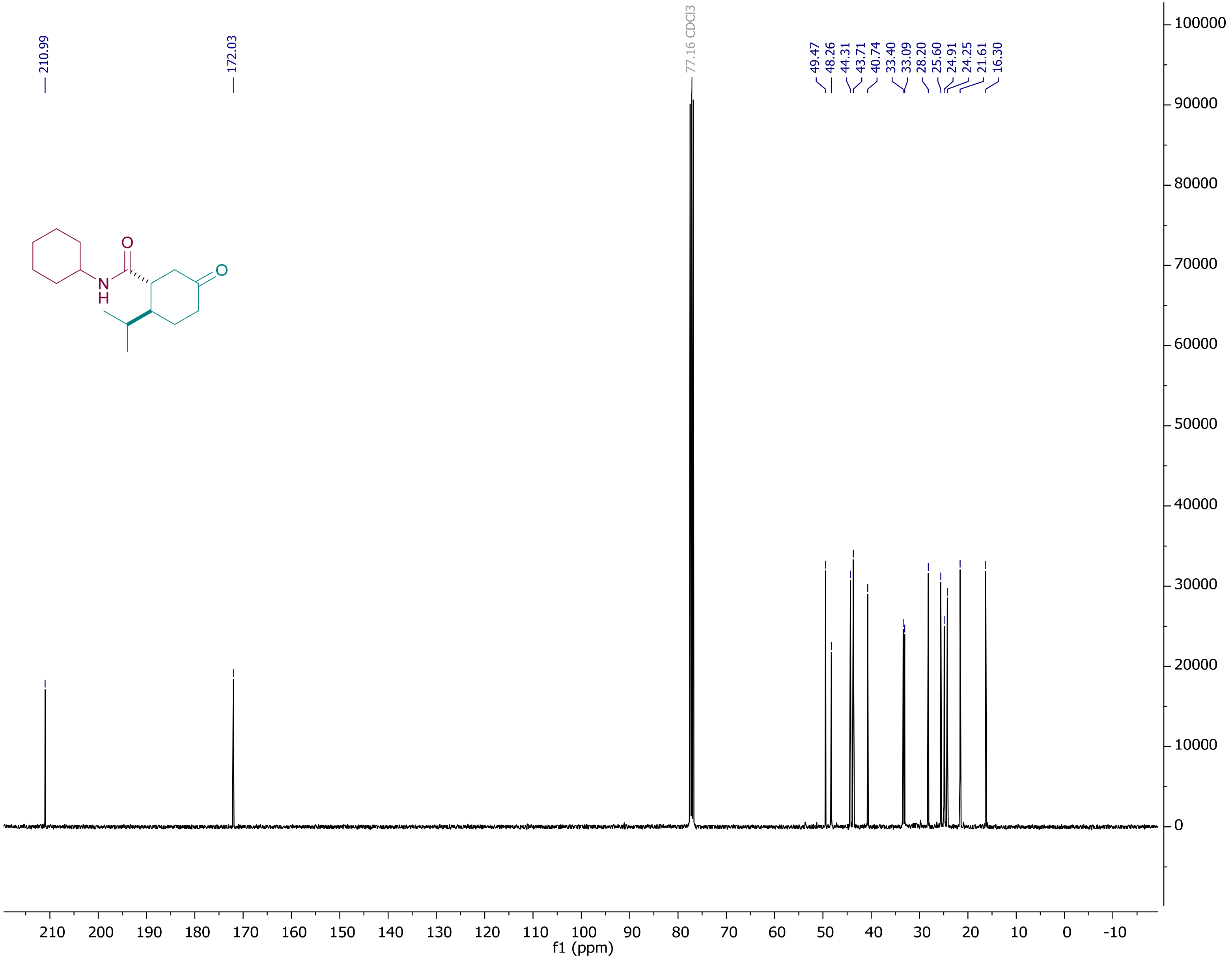

Compound 9aq (Minor Diastereomer)– ^{1}H NMR (400 MHz, Chloroform-*d*):

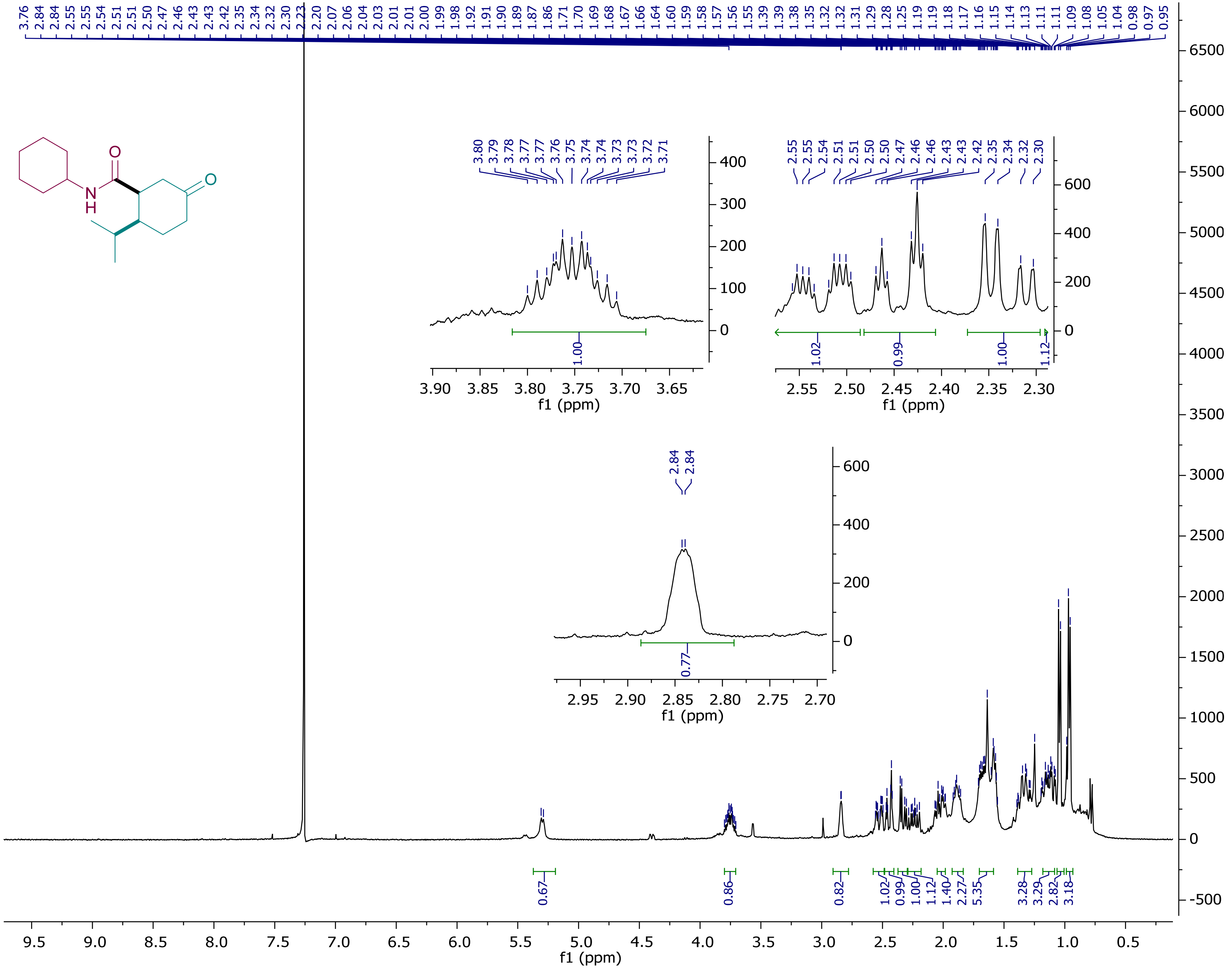

Compound 9aq (Minor Diastereomer)– ^{13}C NMR (101 MHz, Chloroform-*d*):

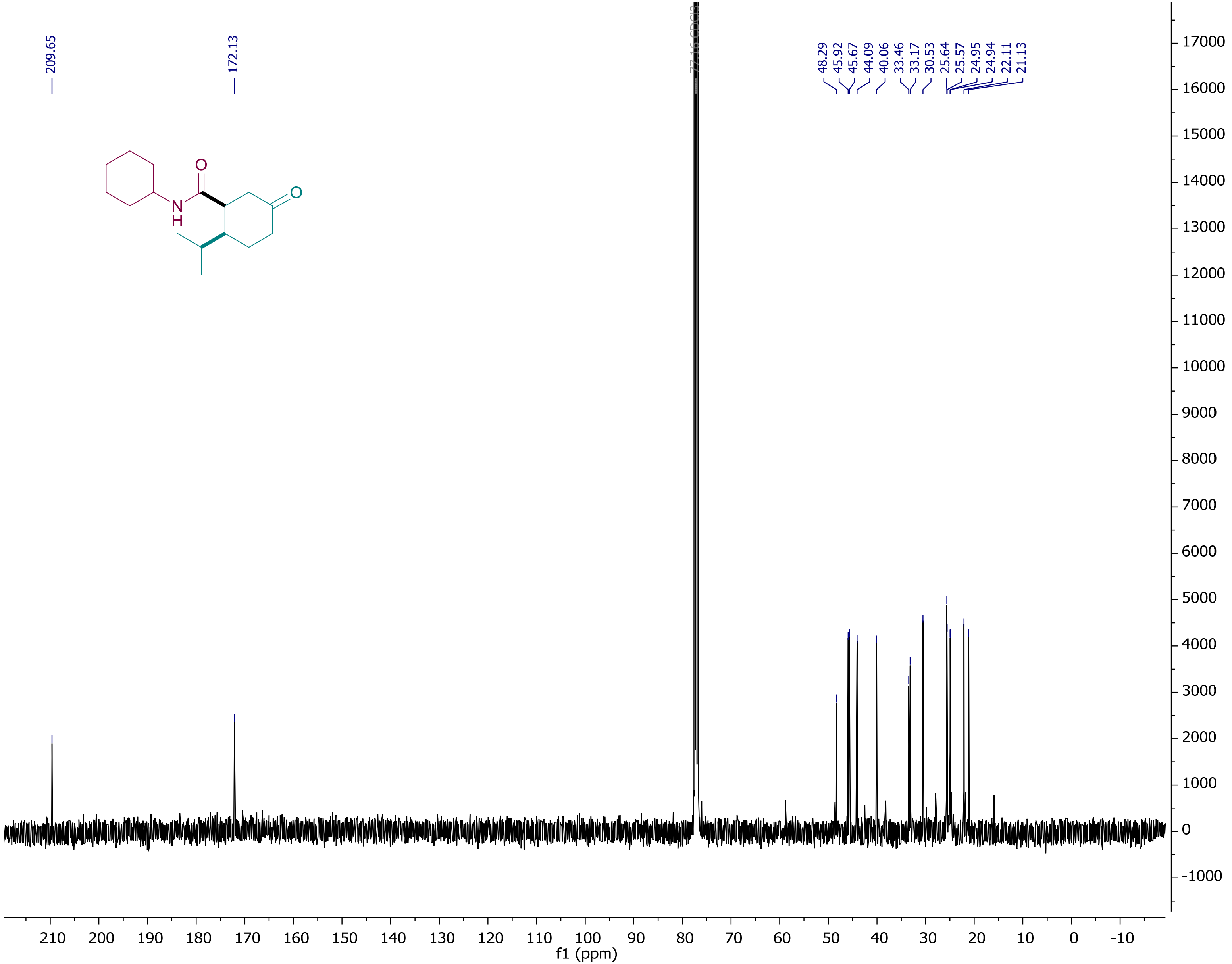

(-)-(3a*R*,4*S*,6a*R*)-2,2-Dimethyl-6-oxo-*N*-phenyltetrahydro-4H-cyclopenta[d][1,3]dioxole-4-carboxamide (9ar)

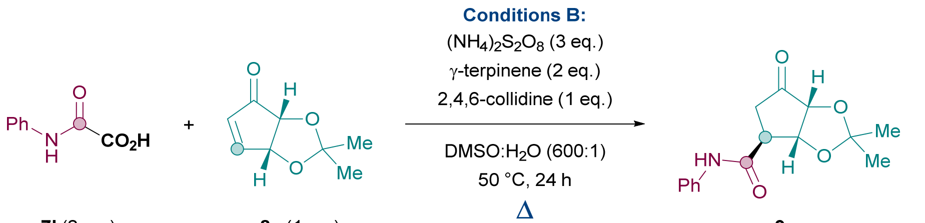

7l (2 eq.) **8v** (1 eq.) **9ar**

Chemical Formula: $C_{15}H_{17}NO_4$
Molecular Weight: 275.30

General procedure for Conditions B was followed. (3a*R*,6a*R*)-2,2-Dimethyl-3aH-cyclopenta[d][1,3]dioxol-4(6aH)-one **8v** (18.5 mg, 0.12 mmol, 1.0 eq.), *N*-phenyl-oxoacetic acid **7l** (39.7 mg, 0.24 mmol, 2.0 eq), $(NH_4)_2S_2O_8$ (82.5 mg, 0.36 mmol, 3.0 eq.), γ-terpinene (38.5 μL, 0.24 mmol, 2.0 eq.), and 2,4,6-collidine (15.9 μL, 0.12 mmol, 1.0 eq.) in DMSO:H_2O (600:1) (0.4 mL) was heated at 50 °C for 24 h followed by work-up Y. The crude was then purified *via* column flash chromatography eluting with 70:30→60:40 hexane:EtOAc. The solvent was then removed *in vacuo* to afford (-)-(3a*R*,4*S*,6a*R*)-2,2-dimethyl-6-oxo-N-phenyltetrahydro-4H-cyclopenta[d][1,3]dioxole-4-carboxamide **9ar** as a yellow solid (17.0 mg, 0.061 mmol, 51%). Product was isolated as a single enantiomer.

Characterisation:

^{1}H NMR (300 MHz, Chloroform-*d*): δ ppm 7.74 (br s, 1H, NH), 7.51 (d, *J* = 7.5 Hz, 2H, ArH), 7.32 (t, *J* = 7.9 Hz, 2H, ArH), 7.14 (t, *J* = 7.4 Hz, 1H, ArH), 4.87 (d, *J* = 5.5 Hz, 1H, CH), 4.51 (d, *J* = 5.5 Hz, 1H, CH), 3.21 (dd, *J* = 8.7, 2.1 Hz, 1H, C<u>H</u>CONH), 2.80 (dd, *J* = 18.2, 8.3 Hz, 1H, C<u>H</u>H), 2.57 (d, *J* = 18.2 Hz, 1H, CH<u>H</u>), 1.45 (s, 3H, CH_3), 1.35 (s, 3H, CH_3). **^{13}C NMR (101 MHz, Chloroform-*d*):** δ ppm 211.1 (C), 171.1 (C), 137.2 (C), 129.3 (CH), 125.2 (CH), 120.2 (CH), 112.5 (C), 79.6 (CH), 78.7 (CH), 45.7 (CH), 37.6 (CH_2), 26.9 (CH_3), 25.0 (CH_3). **IR:** ν_{max}/cm^{-1} 3328, 2952, 2872, 1720, 1679, 1597, 1537, 1489, 1439, 1355, 1308, 1244, 1214. **HRMS (ESI-TOF):** *m/z* $[M + H]^+$ calcd for $C_{15}H_{18}NO_4$, 276.12303; found, 276.1235. **TLC:** R_f = 0.33 (60:40 hexane:EtOAc). **m.p.** 139-143 °C **$[\alpha]_D^{21.3}$** = -68.0 (*c* = 1, $CHCl_3$).

X-ray Crystallography Data for Compound 9ar

The X-ray crystallography data was solely to help prove the stereochemistry at position C4 (see Figure 3 below). The configuration of C3 and C7 are known to be *R* from the starting material used, so C4 was determined to be *anti* to C3 and (*S*)-configuration. The crystals were grown by slow evaporation and solvent diffusion from $CHCl_3$/hexane.

Experimental:

Single crystals of C15H17NO4 **[Compound 9ar]** were coated in NVH oil and a suitable crystal was selected and mounted in a LithoLoop and the goniometer head placed in the Coldstream on a Bruker D8 Venture diffractometer. The crystal was kept at 100.0 K during data collection. Using Olex2[9], the structure was solved with the SHELXD[10] structure solution program using dual space direct methods and refined with the SHELXL[11] refinement package using Least Squares minimisation. The crystals were of poor quality and not single and treated as a twin (twin law -1 0 0 0 -1 0 0 0 -1). Anisotropic displacement parameters were restrained to more isotropic approximations with ISOR and RIGU (rigid-bond) restraints were applied to all non-H atoms. Connectivity and elemental composition had been determined by full characterisation for the compound (^{1}H NMR, ^{13}C NMR, IR, HRMS). Data were deposited with the Cambridge Crystallographic Data Centre and given the number 2258061.

Crystal data: for $C_{15}H_{17}NO_4$ (M =275.29 g/mol): monoclinic, space group P2$_1$ (no. 4), a = 5.20570(10) Å, b = 20.7249(5) Å, c = 12.8023(3) Å, β = 99.7432(15)°, V = 1361.29(5) Å^3, Z = 4, T = 100.0 K, μ(CuKα) = 0.808 mm^{-1}, $Dcalc$ = 1.343 g/cm^3, 37605 reflections measured (7.006° ≤ 2Θ ≤ 144.588°), 5348 unique (R_{int} = 0.0549, R_{sigma} = 0.0332) which were used in all calculations. The final R_1 was 0.1991 (I > 2σ(I)) and wR_2 was 0.5539 (all data).

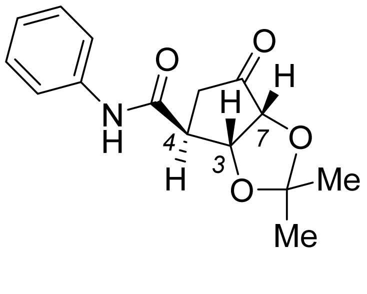

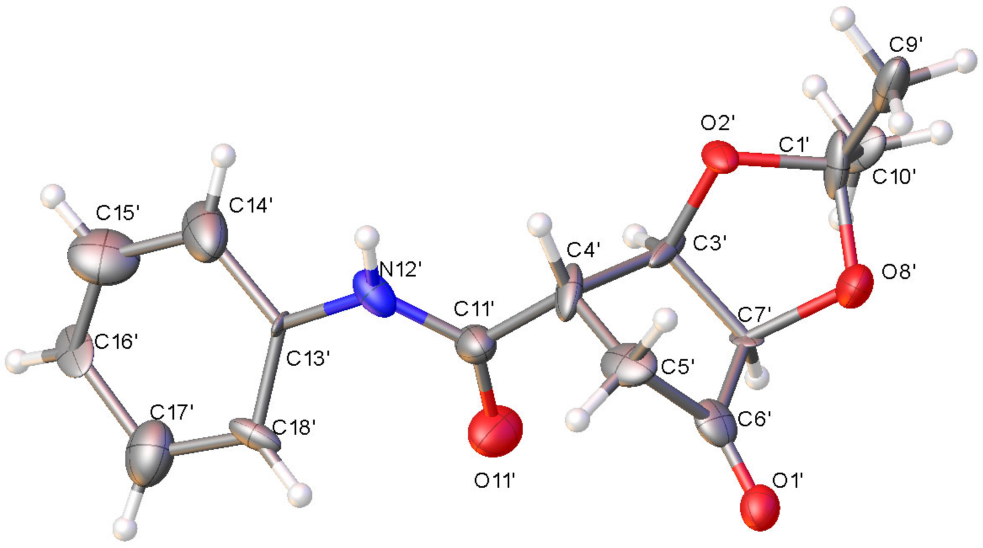

Figure 3. Crystal structure and ChemDraw structure of product **9ar**.

Table: Crystal data and structure refinement for cu_8v00967_0m_a.

Identification code	cu_8v00967_0m_a
Empirical formula	$C_{15}H_{17}NO_4$
Formula weight	275.29
Temperature/K	100.0
Crystal system	monoclinic
Space group	$P2_1$
a/Å	5.20570(10)
b/Å	20.7249(5)
c/Å	12.8023(3)
α/°	90
β/°	99.7432(15)
γ/°	90
Volume/Å^3	1361.29(5)
Z	4
ρ_{calc}g/cm^3	1.343
μ/mm^{-1}	0.808
F(000)	584.0
Crystal size/mm^3	0.22 × 0.2 × 0.04
Radiation	CuKα (λ = 1.54178)
2Θ range for data collection/°	7.006 to 144.588
Index ranges	-6 ≤ h ≤ 6, -25 ≤ k ≤ 25, -15 ≤ l ≤ 15
Reflections collected	37605
Independent reflections	5348 [R_{int} = 0.0549, R_{sigma} = 0.0332]
Data/restraints/parameters	5348/415/366
Goodness-of-fit on F^2	1.026
Final R indexes [I>=2σ (I)]	R_1 = 0.1991, wR_2 = 0.5464
Final R indexes [all data]	R_1 = 0.2050, wR_2 = 0.5539

Largest diff. peak/hole / e $Å^{-3}$	1.95/-1.86
Flack parameter	-0.2(11)

Compound 9ar – ^{1}H NMR (300 MHz, Chloroform-*d*):

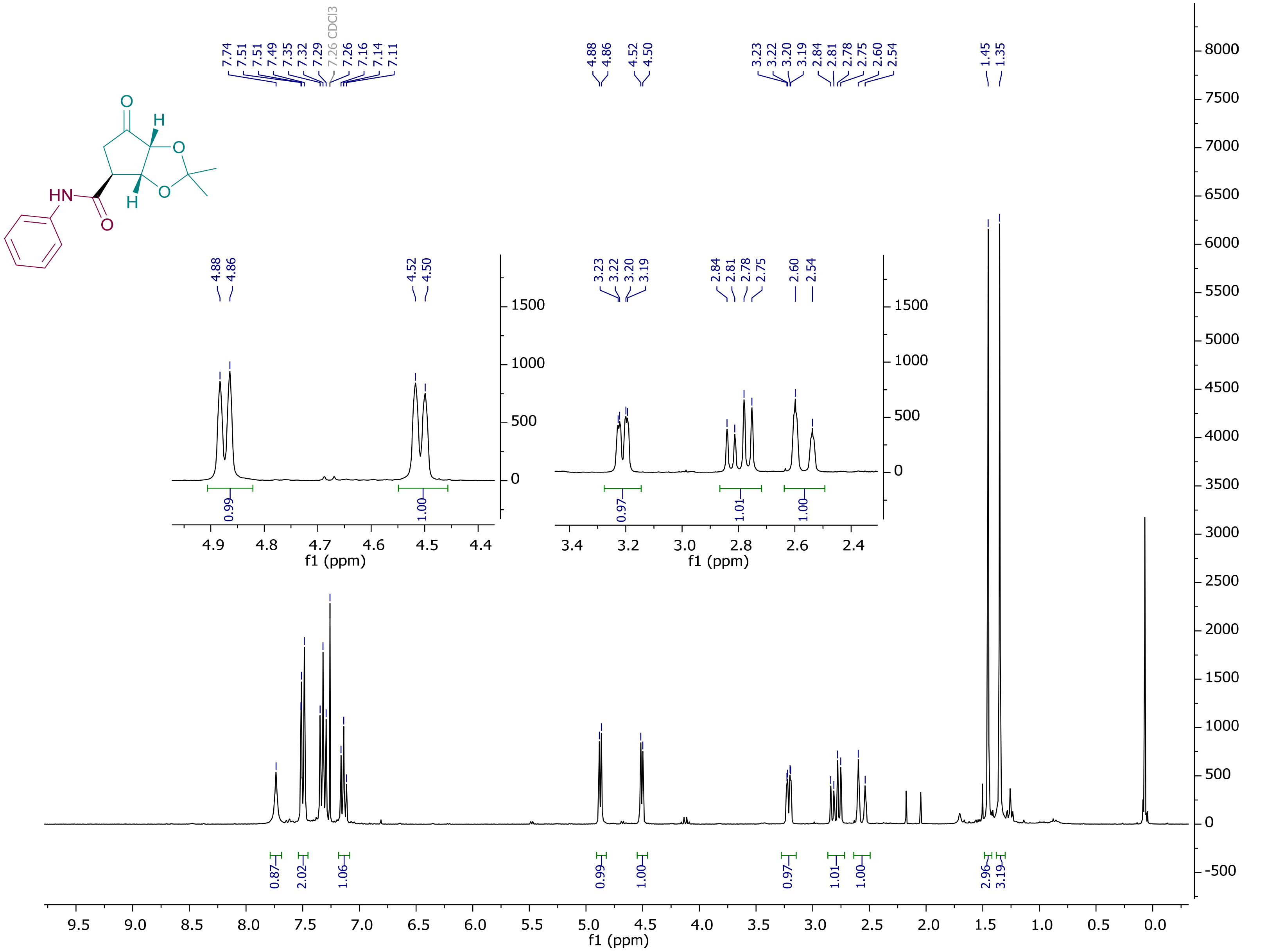

Compound 9ar – ^{13}C NMR (101 MHz, Chloroform-*d*):

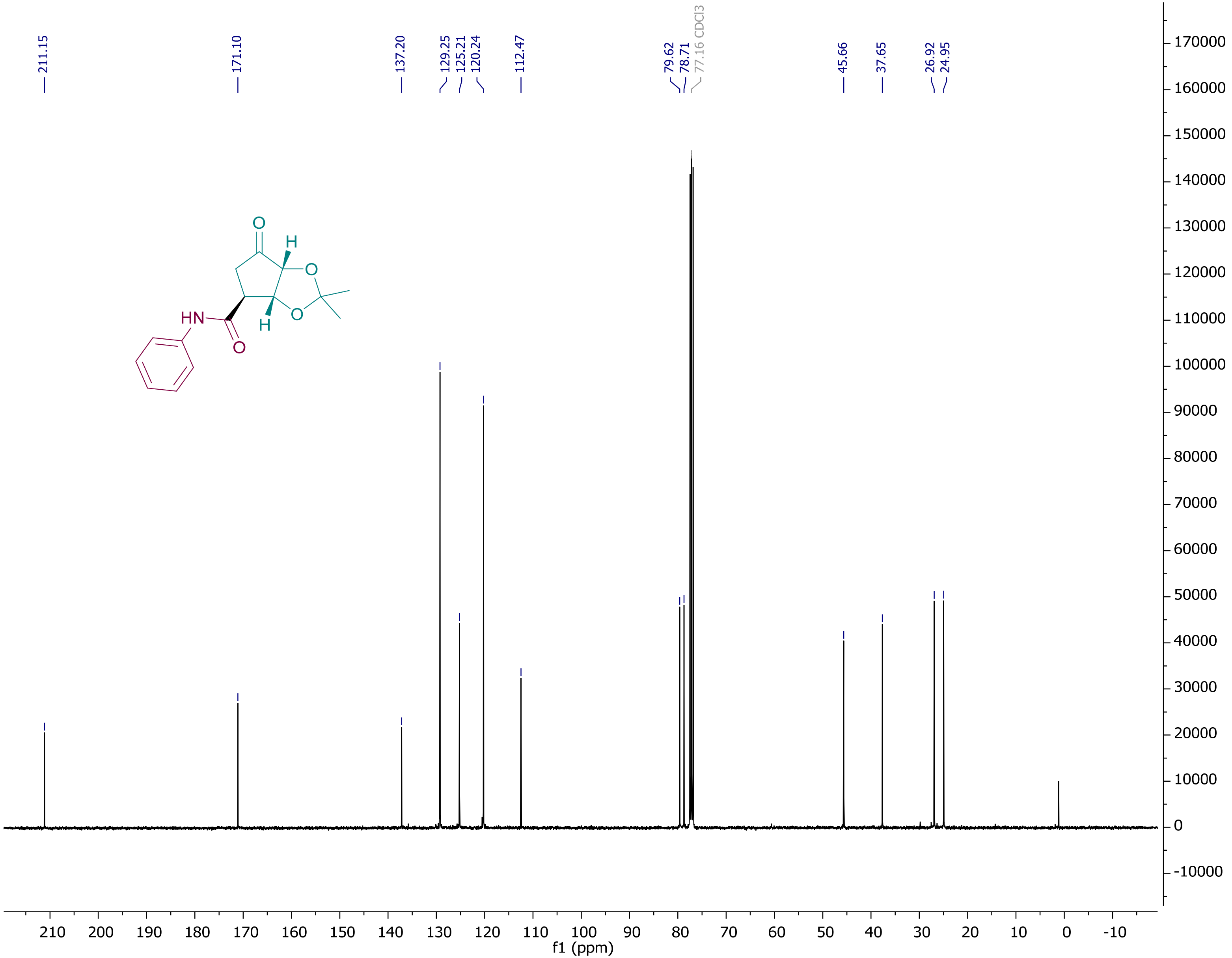

(-)-(3a*R*,4*S*,6a*R*)-*N*-(((1*R*,4aS,10a*R*)-7-Isopropyl-1,4a-dimethyl-1,2,3,4,4a,9,10,10a-octahydrophenanthren-1-yl)methyl)-2,2-dimethyl-6-oxotetrahydro-4*H*-cyclopenta[d][1,3]dioxole-4-carboxamide (9as)

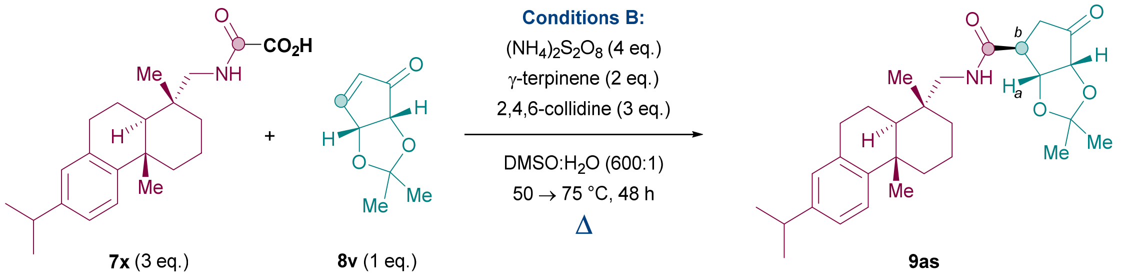

9as
Chemical Formula: $C_{29}H_{41}NO_4$
Molecular Weight: 467.65

General procedure for Conditions B was followed. (3a*R*,6a*R*)-2,2-Dimethyl-3aH-cyclopenta[d][1,3]dioxol-4(6aH)-one **8v** (18.5 mg, 0.12 mmol, 1.0 eq.), 2-((((1*R*,4a*S*,10a*R*)-7-isopropyl-1,4a-dimethyl-1,2,3,4,4a,9,10,10a-octahydrophenanthren-1-yl)methyl)amino)-2-oxoacetic acid **7x** (128.7 mg, 0.36 mmol, 3.0 eq), $(NH_4)_2S_2O_8$ (109.53 mg, 0.48 mmol, 4.0 eq.), γ-terpinene (38.5 µL, 0.24 mmol, 2.0 eq.), and 2,4,6-collidine (47.6 µL, 0.36 mmol, 3.0 eq.) in DMSO:H_2O (600:1) (0.4 mL) was heated at 50 °C for 24 h then 75 °C for 24 h followed by work-up Y. The crude was then purified *via* column flash chromatography eluting with 65:35 hexane:EtOAc. The solvent was then removed *in vacuo* to afford methyl (-)-(3a*R*,4*S*,6a*R*)-*N*-(((1*R*,4aS,10a*R*)-7-isopropyl-1,4a-dimethyl-1,2,3,4,4a,9,10,10a-octahydrophenanthren-1-yl)methyl)-2,2-dimethyl-6-oxotetrahydro-4*H*-cyclopenta[d][1,3]dioxole-4-carboxamide **9as** as a brown oil (19.9 mg, 35%). Product was isolated as a single enantiomer. The *anti* stereochemistry between H_a and H_b was assigned by analogy with compound **9ar**.

Characterisation:

^{1}H NMR (400 MHz, Chloroform-*d*): δ ppm 7.17 (d, J = 8.2 Hz, 1H, ArH), 7.00 (dd, J = 8.2, 1.6 Hz, 2H, ArH), 6.90 (s, 1H, ArH), 5.74 (t, *J* = 6.2 Hz, 1H, NH), 4.71 (d, *J* = 5.3 Hz, 1H, CH), 4.46 (d, *J* = 5.4 Hz, 1H, CH), 3.22 (dd, *J* = 13.7, 6.5 Hz, 1H, C<u>H</u>HCO), 3.14 (dd, *J* = 13.8, 6.5 Hz, 1H, CH<u>H</u>CO), 3.02 – 2.88 (m, 2H, CH, C<u>H</u>H), 2.88 – 2.71 (m, 2H, CH, CH<u>H</u>), 2.68 (dd, *J* = 18.0, 8.1 Hz, 1H, C<u>H</u>H), 2.38 (d, *J* = 18.1 Hz, 1H, CH<u>H</u>), 2.31 (d, *J* = 12.8 Hz, 1H, C<u>H</u>H), 1.88 – 1.62 (m, 4H, 2 x CH_2), 1.50 – 1.36 (m, 6H, CH, C<u>H</u>H, CH<u>H</u>, CH_3), 1.32 (s, 3H, CH_3), 1.31 – 1.16 (m, 10H, CH<u>H</u>, 3 x CH_3), 0.95 (s, 3H, CH_3). **^{13}C NMR (101 MHz,**

Chloroform-*d*): δ ppm 211.0 (C), 172.8 (C), 147.1 (C), 145.9 (C), 134.6 (C), 127.1 (CH), 124.3 (CH), 124.1 (CH), 112.2 (C), 79.8 (CH), 78.7 (C), 50.1 (CH_2), 45.6 (CH), 45.0 (CH), 38.5 (CH_2), 37.7 (CH_2), 37.6 (C), 37.5 (C), 36.5 (CH_2), 33.6 (CH), 30.2 (CH_2), 26.9 (CH_3), 25.4 (CH_3), 24.9 (CH_3), 24.1 (CH_3 plus one overlapping CH_3), 19.1 (CH_2), 18.8 (CH_3), 18.7 (CH_2). **IR:** ν_{max}/cm^{-1} 3323, 2958, 2925, 2868, 2357, 2323, 1755, 1650, 1634, 1537, 1498, 1460, 1382, 1211, 1153. **HRMS (ESI-TOF):** *m*/*z* $[M + H]^+$ calcd for $C_{29}H_{42}NO_4$, 468.31084; found, 468.3094. **TLC:** R_f = 0.19 (60:40 hexane:EtOAc). $[\alpha]_D^{21.3}$ = -40.0 (*c* = 1, $CHCl_3$).

Compound 9as – ^{1}H NMR (400 MHz, Chloroform-*d*):

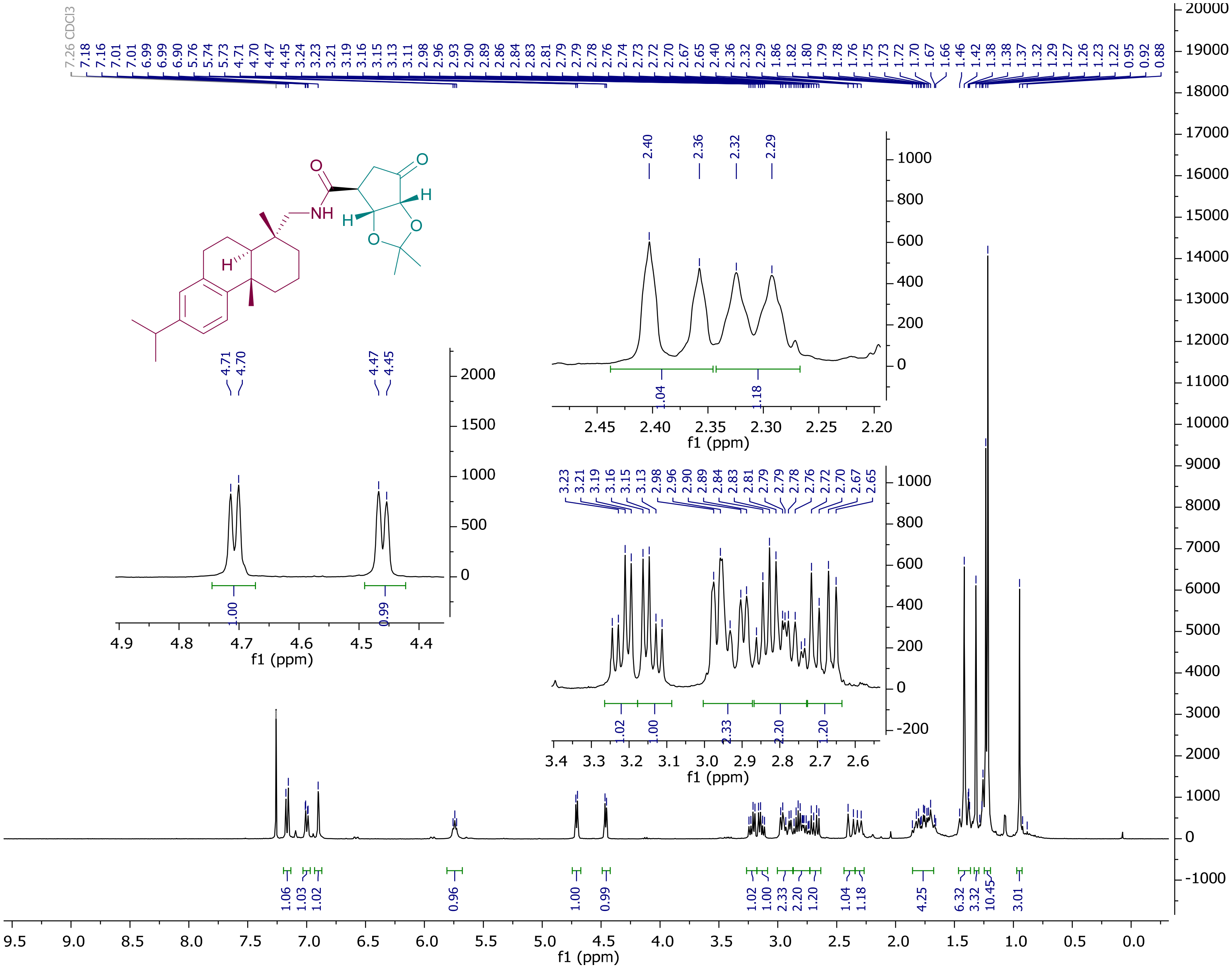

Compound 9as – ^{13}C NMR (101 MHz, Chloroform-*d*):

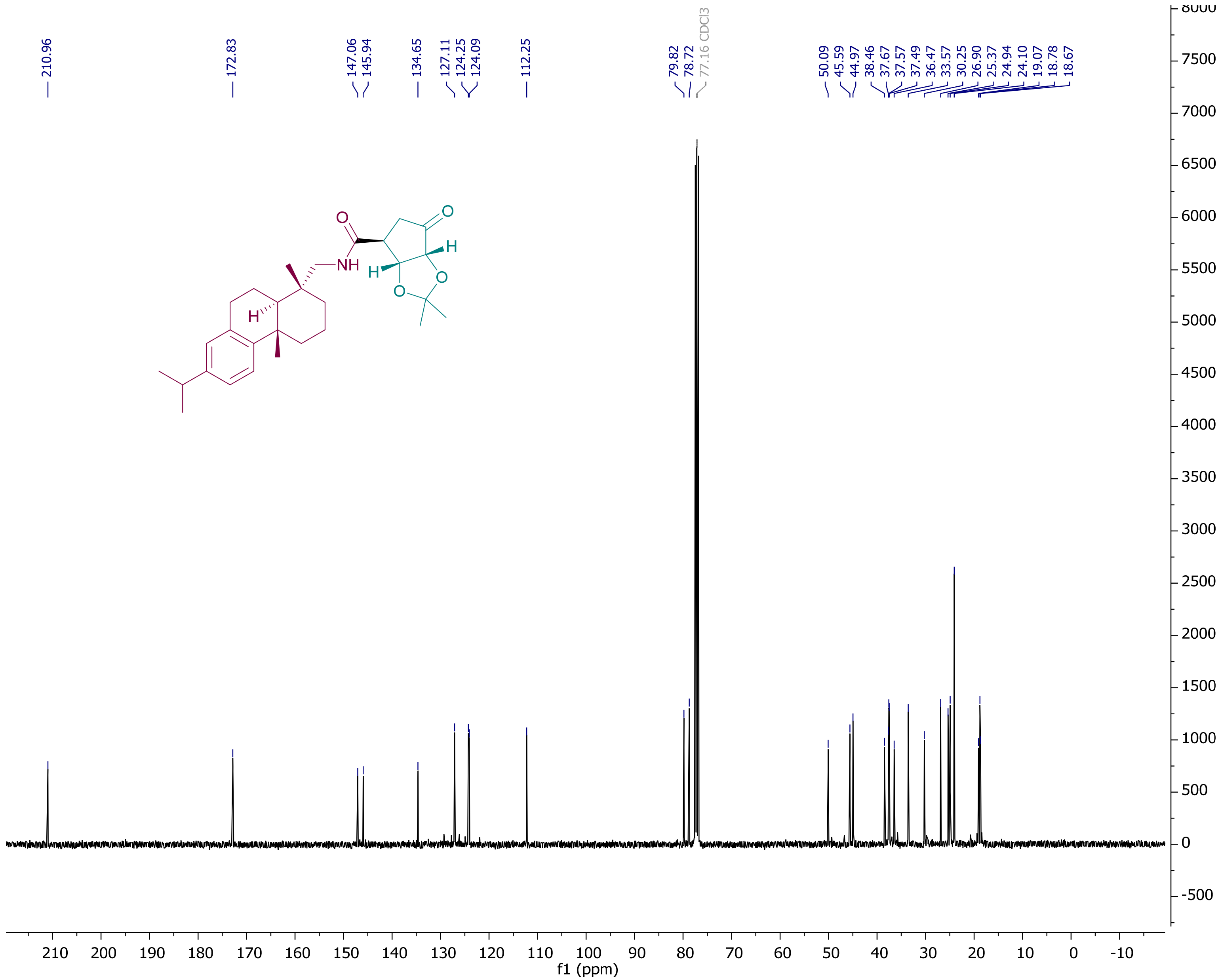

6. References

1. H. E. Gottlieb, V. Kotlyar and A. Nudelman, *J. Org. Chem.*, 1997, **62**, 7512-7515.
2. (a) D. T. Mooney, B. D. T. Donkin, N. Demirel, P. R. Moore and A.-L. Lee, *J. Org. Chem.*, 2021, **86**, 17282-17293; (b) D. T. Mooney, P. R. Moore and A.-L. Lee, *Org. Lett.*, 2022, **24**, 8008-8013; (c) D. R. Sutherland, M. Veguillas, C. L. Oates and A.-L. Lee, *Org. Lett.*, 2018, **20**, 6863-6867; (d) M. T. Westwood, C. J. C. Lamb, D. R. Sutherland and A.-L. Lee, *Org. Lett.*, 2019, **21**, 7119-7123.
3. E. B. McLean, D. T. Mooney, D. J. Burns and A.-L. Lee, *Org. Lett.*, 2022, **24**, 686-691.
4. V. A. Pistritto, S. Liu and D. A. Nicewicz, *J. Am. Chem. Soc.*, 2022, **144**, 15118-15131.
5. J. N. Demas, W. D. Bowman, E. F. Zalewski and R. A. Velapoldi, *J. Phys. Chem.*, 1981, **85**, 2766-2771.
6. Y. Seki, K. Tanabe, D. Sasaki, Y. Sohma, K. Oisaki and M. Kanai, *Angew. Chem. Int. Ed.*, 2014, **53**, 6501-6505.
7. K. Chen, Y. Ishihara, M. M. Galán and P. S. Baran, *Tetrahedron*, 2010, **66**, 4738-4744.
8. W. Cocker, D. H. Grayson and P. V. R. Shannon, *J. Chem. Soc., Perkin Trans. 1*, 1995, 1153-1162.
9. O. V. Dolomanov, L. J. Bourhis, R. J. Gildea, J. A. K. Howard and H. Puschmann, *J. Appl. Cryst.*, 2009, **42**, 339-341.
10. G. M. Sheldrick, *Acta Cryst., Sect. A: Found. Cryst.*, 2008, **64**, 112-122.
11. G. M. Sheldrick, *Acta Cryst., Sect. C: Struct. Chem.*, 2015, **71**, 3-8.
